# rAAV-CRISPRa therapy corrects *Rai1* haploinsufficiency and rescues selective disease features in Smith-Magenis syndrome mice

**DOI:** 10.1016/j.jbc.2022.102728

**Published:** 2022-11-19

**Authors:** Hao-Cheng Chang, Yu-Ju Lee, Sehrish Javed, Minza Haque, Ya-Ting Chang, Yu Cheng Lin, Cameron Oram, Wei-Hsiang Huang

**Affiliations:** 1 Department of Neurology and Neurosurgery, Centre for Research in Neuroscience, McGill University, Québec, Canada; 2 Brain Repair and Integrative Neuroscience Program, The Research Institute of the McGill University Health Centre, Montréal, Québec, Canada

**Keywords:** CRISPR activation, gene therapy, mouse models, neurodevelopmental disorders, retinoic acid induced 1, Smith-Magenis syndrome, ALT, alanine aminotransferase, AST, aspartate aminotransferase, Bdnf, brain-derived neurotrophic factor, Cas9, CRISPR associated protein 9, CRF, corticotropin releasing factor receptor, CRISPR, clustered regularly interspaced short palindromic repeats, CLAMS, Comprehensive Lab Animal Monitoring System metabolic cages, EE, energy expenditure, GFAP, glial fibrillary acidic protein, HDL, high-density lipoprotein, LDL, low-density lipoproteins, LPS, lipopolysaccharide, MC4R, melanocortin-4 receptor, PVH, paraventricular nucleus of the hypothalamus, PTLS, Potocki-Lupski Syndrome, qRT-PCR, quantitative real-time polymerase chain reaction, rAAV, recombinant adeno-associated virus, RAI1, retinoic acid induced 1, RER, respiratory exchange rate, sgRNA, single guide RNA, SMS, Smith-Magenis Syndrome, VLDL, very low-density lipoproteins

## Abstract

Haploinsufficiency in *retinoic acid induced 1* (*RAI1*) causes Smith-Magenis syndrome (SMS), a severe neurodevelopmental disorder characterized by neurocognitive deficits and obesity. Currently, curative treatments for SMS do not exist. Here, we take a recombinant adeno-associated virus (rAAV)-clustered regularly interspaced short palindromic repeats activation (CRISPRa) approach to increase expression of the remaining intact *Rai1* allele. Building upon our previous work that found the paraventricular nucleus of hypothalamus plays a central role in SMS pathogenesis, we performed paraventricular nucleus of hypothalamus–specific rAAV-CRISPRa therapy by increasing endogenous *Rai1* expression in SMS (*Rai1*^*±*^) mice. We found that rAAV-CRISPRa therapy rescues excessive repetitive behavior, delays the onset of obesity, and partially reduces hyperphagia in SMS mice. Our work provides evidence that rAAV-CRISPRa therapy during early adolescence can boost the expression of healthy *Rai1* allele and modify disease progression in a mouse model of Smith-Magenis syndrome.

Smith-Magenis syndrome (SMS) is a syndromic autism spectrum disorder associated with stereotyped behaviors, social interaction deficits, and hyperphagic obesity (1, 2). SMS is caused by loss-of-function mutations in *retinoic acid induced 1* (*RAI1*) or 17p11.2 chromosomal deletions that contain more than 70 genes including *RAI1* (1, 3). *Rai1* heterozygous mice (hereafter, SMS mice) exhibit three hallmark disease features: increased repetitive rearing, impaired social dominance, and hyperphagic obesity (4, 5, 6, 7). 17p11.2 duplication in human causes Potocki-Lupski syndrome (PTLS), a distinct neurodevelopmental disorder (8). *RAI1* is the only gene located within a 125 kilobase interval shared among PTLS-causing duplications (9). Collectively, clinical evidence suggests that brain function and energy homeostasis are highly sensitive to *RAI1* dosage (10).

Our previous cell type–specific study found that Rai1 loss from *Vglut2*^*Cre*^-lineage subcortical excitatory neurons (but not *Emx1*^*Cre*^ -lineage cortical excitatory neurons) recapitulates SMS-like obesity and neurobehavioral features in mice (11). More recently, we found that homozygous *Rai1* loss in *Emx1*^*Cre*^-lineage cortical excitatory neurons (but not GABAergic neurons) underlies increased seizure susceptibility in mice (12). Within subcortical structures, deleting *Rai1* from one region, the paraventricular nucleus of hypothalamus (PVH) was sufficient to induce SMS-like obesity in mice (11, 13). PVH neurons regulate energy homeostasis (14), social interaction (15, 16), and repetitive rearing behavior (17). Therefore, we considered PVH an attractive brain region for therapeutically modifying disease progression in SMS mice.

Mouse models of SMS have shown that *Rai1* haploinsufficiency does not result in irreparable damage of brain function. By engineering a Cre-dependent reactivatable *Rai1* allele, we reported that correcting *Rai1* haploinsufficiency during early adolescence (3–4 weeks of age) partially rescues SMS-like neurobehavioral defects in mice (5). By contrast, normalizing *Rai1* expression levels at 7 to 8 weeks of age is not therapeutically effective. More recently, we found that PVH-specific overexpression of *brain-derived neurotrophic factor* (*Bdnf*), one of Rai1’s direct target genes, during early adolescence was sufficient to fully rescue obesity in SMS mice (13). Building on these studies, we hypothesize that obesity and neurobehavioral features in SMS mice can be partially mitigated using gene therapy that enhances expression of the remaining *Rai1* allele in the PVH during early adolescence.

Recombinant adeno-associated virus (rAAV) is the leading platform for gene therapy due to its long-lasting transgene expression, low immunogenicity, and lack of genomic integration (18, 19). However, the development of *Rai1* gene transfer therapy is limited by the size of *Rai1* coding sequence, which exceeds rAAV’s payload capacity. The advent of clustered regularly interspaced short palindromic repeats activation (CRISPRa) system and the discovery of the smaller *Staphylococcus aureus* CRISPR-associated protein 9 (saCas9) hold great promise for rAAV-mediated, gene size-independent therapy in treating SMS and other disorders associated with genetic forms of obesity, epilepsy, blindness, and muscular dystrophy (20, 21, 22, 23, 24, 25, 26, 27). A common strategy to express Cas9 and single-guide RNAs (sgRNAs) is using dual rAAV systems that could result in low co-infection efficiency (28). To overcome this challenge, we adapted an “all-in-one” single vector rAAV-CRISPRa system that encodes a sgRNA and a nuclease-deficient saCas9 (sadCas9) fused with the VP64 transcriptional activator (25). In SMS mice, we show that PVH-specific rAAV-CRISPRa gene therapy enhances the expression of the remaining wildtype (WT) *Rai1* allele, fully reverses repetitive behavior and partially rescues hyperphagic obesity and delays its onset. We increased the expression levels of *Bdnf*, a key Rai1 direct target gene, using rAAV8-CRISPRa gene therapy *in vitro* and *in vivo*. These findings highlight the interventional potential of rAAV-CRISPRa in reversing *Rai1* haploinsufficiency and ameliorating selective disease features in SMS.

## Results

### Identification of sgRNAs that target Rai1 proximal promoter and stimulate Rai1 expression *in vitro*

**Figure 1:**
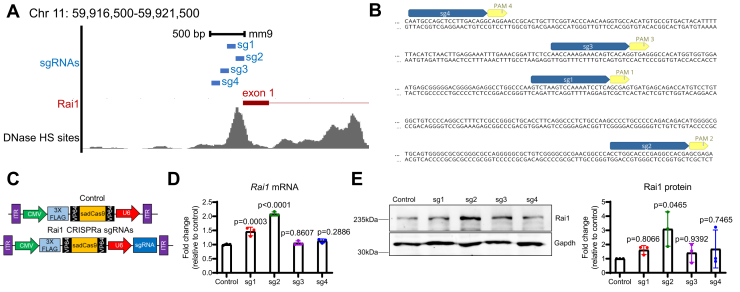
**sadCas9-2 × VP64-sgRNA upregulates *Rai1* expression *in vitro*.***A*, schematic representation of the mouse *Rai1* promoter. DNase hypersensitive sites (HS) with open chromatin configurations are shown in gray. *B*, positions of four sgRNAs within *Rai1*’s proximal promoter, with sequences of each sgRNA (*blue*) immediately followed by *Sa* protospacer adjacent motif (PAM) sequences (*yellow*). *C*, schematic representation of the sadCas9-2 × VP64 constructs. *D*, qRT-PCR showing that sadCas9-2 × VP64-sg1 and sadCas9-2 × VP64-sg2 vectors significantly increase *Rai1* mRNA expression (one-way ANOVA with Dunnett’s post-hoc test, F (4, 10) = 76.13291, *p* <0.0001). *p*-values for the post-hoc tests are indicated in the figure. *E*, *left*: representative images of Western blotting data showing that sadCas9-2 × VP64-sg2 significantly increases Rai1 protein levels. Molecular weights are indicated on the right. *Right*: quantification of Western blotting data (one-way ANOVA with Dunnett’s post-hoc test, F (4, 10) = 2.464, *p* =0.1128). Data in *D* and *E* are means ±SD. *p*-values represent comparisons between experimental groups to cells expressing no guide sadCas9-2 × VP64.

To enhance expression of the endogenous *Rai1* allele, we focused on a 500-nucleotide promoter region immediately upstream of the *Rai1* transcription start site with an open chromatin configuration in the mouse brain (Fig. 1*A*). We screened for the ability of four different sgRNAs to activate *Rai1* transcription (Fig. 1, *A* and *B*, see Table S1 for sgRNA properties). Each sgRNA was separately cloned into an “all-in-one” rAAV vector encoding the sadCas9 protein fused with 3 × FLAG tags and 2 × VP64 transcriptional activators (Fig. 1*C*) (25). We individually lipofected either a no-guide control or the sadCas9-2 × VP64-sg1 to -sg4 plasmids into mouse neuro2a cells. By detecting the expression of FLAG-sadCas9, we found that all plasmids showed similarly high transfection efficiencies (90–92%, Fig. S1*A*–*B*). Forty-eight hours after transfection, we measured *Rai1* mRNA levels using quantitative reverse transcription polymerase chain reaction (qRT-PCR). We found that only sg1 and sg2 consistently increase *Rai1* mRNA expression to 1.5- and 2-fold compared to control plasmid transfected cells, respectively (Fig. 1*D*). To validate the anti-RAI1 antibody (Abcam ab86599), we generated human *RAI1* knockout 293A cells using two different pairs of sgRNAs (Fig. S1*C*). We subsequently confirmed that *RAI1* mRNA levels were significantly reduced in four different clones (two clones per guide-pair, Fig. S1*D*). Consistent with these results, Western blotting assays showed a near-complete loss of RAI1 protein in all four *RAI1* knockout clones (Fig. S1*E*). Importantly, we found that the same anti-RAI1 antibody reliably detects overexpressed human RAI1 and mouse Rai1 proteins (Fig. S1*F*). We then performed Western blotting assays and confirmed that sg2 is the only guide RNA that significantly increases mouse Rai1 protein abundance 72 h after transfection (Fig. 1*E*). Therefore, we proceeded with sg2 because of its ability to reliably increase mouse *Rai1* mRNA and protein levels and its minimal predicted off-target activity (Table S2).

### rAAV8-sg2 treatment increases the expression of Rai1 and its downstream target genes in mouse neurons

**Figure 2:**
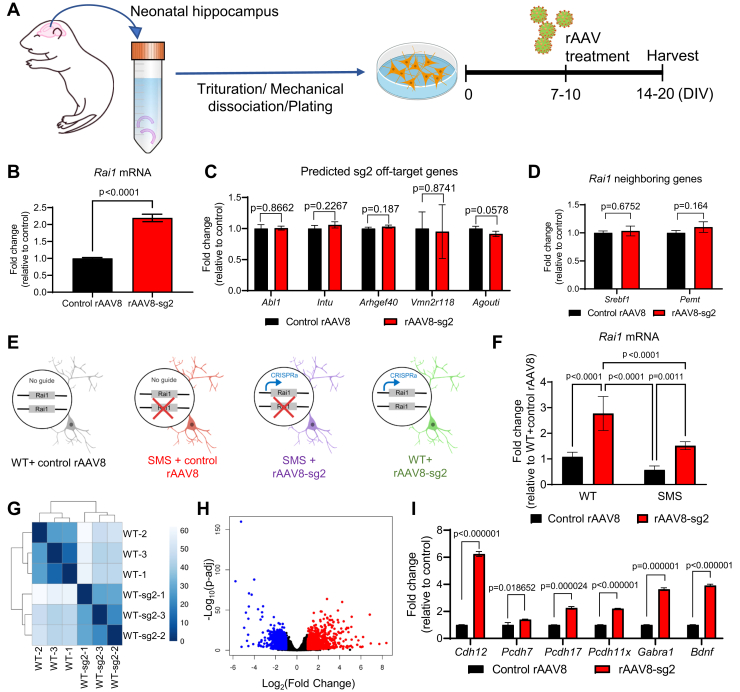
**Transcriptional profiling uncovers that rAAV8–sg2 particles upregulate *Rai1* and its downstream target genes in primary mouse neurons.***A*, schematic representation of the experimental procedures. *B*, rAAV8–sg2 treatment induces a significant increase of *Rai1* mRNA expression in neurons (unpaired *t* test). *C*, primary neurons treated with control rAAV8 and rAAV8–sg2 show similar expression of predicted sg2 off-target genes (unpaired *t* tests). *D*, *Rai1* neighboring genes do not show differential expression in primary neurons treated with control rAAV8 or rAAV8–sg2 (unpaired *t* tests). *E*, schematic representation of four different experimental groups of primary neuronal cultures. *F*, qRT-PCR analysis showing that *Rai1* mRNA levels in WT and SMS mice were increased by rAAV8-sg2 treatment. Two-way ANOVA with Tukey’s post-hoc test. Genotype: F (1, 20) = 35.84, *p* < 0.0001; Treatment: F (1, 20) = 79.93, *p* < 0.0001; Genotype and treatment interaction: F (1, 20) = 6.425, *p* = 0.0197. *p*-values for the post-hoc tests are indicated in the figure. *G*, sample similarity assessment found that samples were clustered based on their rAAV8 treatment. *H*, a volcano plot showing global transcriptional changes by comparing WT neurons treated with control rAAV8 or rAAV8-sg2. The log_2_ fold change of each gene is represented on the x-axis, and the log_10_ of its adjusted *p*-value is on the y-axis. Genes upregulated by rAAV8-sg2 treatment with an adjusted *p*-value less than 0.05 and a log_2_ fold change greater than one are indicated by *red dots*. Genes downregulated by rAAV8-sg2 treatment with an adjusted *p*-value less than 0.05 and a log_2_ fold change less than -1 are indicated by *blue dots*. *I*, qRT-PCR analysis confirming the expression of genes previously shown to be downregulated in *Rai1*-deficient brains are upregulated by rAAV8-sg2 treatment (unpaired *t* tests). Data are shown as means ±SD.

Next, we packaged the sadCas9-2 × VP64 vectors that express sadCas9 without guide RNA (hereafter, control rAAV8) and sadCas9-2 × VP64-sg2 (hereafter, rAAV8-sg2) into rAAV particles with capsid 8, a serotype with efficient brain transduction (29). We then tested if rAAV8-sg2 viral particles can stimulate endogenous *Rai1* expression in primary hippocampal neurons isolated from WT neonatal mice. We treated dissociated primary hippocampal neurons with control rAAV8 particles at 10 days *in vitro* (DIV) (Fig. 2*A*). Immunostaining experiments found that the primary hippocampal cultures on average contained 79.78 ± 1.56% of NeuN^+^ neurons and 10.93 ± 1.04% of glial fibrillary acidic protein-expressing (GFAP^+^) glial cells (Fig. S1*G*–*H*). The majority of NeuN^+^ neurons (80.37 ± 2.86%) and a subset of GFAP^+^ glial cells (44.59 ± 4.51%) were targeted by rAAV8 (Fig. S1*I*). We also confirmed that control rAAV8 and rAAV8-sg2 viruses showed similar infection efficiencies in WT and SMS neurons (Fig. S1*J*), as indicated by >80% colocalization of NeuN and FLAG signals across groups (Fig. S1*J*–*K*).

Next, we analyzed gene expression and found that at DIV20, rAAV8-sg2 treatment increased *Rai1* mRNA levels to 2 folds compared to neurons treated with control rAAV8 (Fig. 2*B*). To determine the specificity of rAAV8-sg2, we compiled the top 50 predicted off-target binding sites for sg2 and found that most predicted sites show low likelihood scores (Table S2). Only five out of the 50 putative off-target sites have the potential to modulate gene expression because they are proximal to the *Abl1*, *Intu*, *Arhgef40*, *Vmn2r118*, and *Agouti* genes. However, qRT-PCR found that the mRNA levels of these genes did not differ in neurons treated with control rAAV8 or rAAV8-sg2 (Fig. 2*C* and Table S3). Furthermore, rAAV8-sg2 treatment did not alter expression of Rai1’s neighboring genes *Srebf1* and *Pemt* (Fig. 2*D*). Importantly, rAAV8-sg2 treatment significantly increases *Rai1* expression in WT and SMS neurons (Fig. 2, *E* and *F*).

To globally determine the effects of rAAV8-sg2 treatment on the neuronal transcriptome, we performed RNA-sequencing using WT neurons treated with either control rAAV8 or rAAV8-sg2 (n = 3 biological replicates per group). Sample similarity assessment based on gene expression profile showed that samples clustered according to rAAV treatment (Fig. 2*G*), suggesting that rAAV8-sg2 induced a distinct transcriptomic profile. Rai1 is a transcription regulator with hundreds of downstream targets including other transcriptional factors, cell adhesion molecules, and neurotransmitter receptors that could mediate secondary changes of gene expression (11). As a result, rAAV8-sg2 treatment induces 2356 differentially expressed genes, including 1192 and 1164 genes that were upregulated and downregulated by rAAV8-sg2 treatment, respectively (Fig. 2*H* and Tables S4 and S5). While this feature of Rai1 prevented us from systemically analyzing the off-target effects of rAAV8-sg2, RNA-sequencing experiment found that the expression levels of *Abl1*, *Intu*, *Arhgef40*, *Vmn2r118*, and *Agouti*, as well as *Rai1* neighboring genes *Srebf1* and *Pemt* remained unchanged after rAAV8-sg2 treatment (Fig. 2, *C* and *D*). Our previous work found that the four biological processes most sensitive to *Rai1* dosage are “homophilic cell adhesion”, “cell adhesion”, “biological adhesion”, and “cell-cell adhesion” (11). Similarly, the only category of genes significantly enriched by rAAV8-sg2 treatment involves “cell adhesion” (*p* = 5.12 × 10^−8^, Fisher exact test). Guided by RNA-sequencing, we performed qRT-PCR and confirmed that many cell surface molecules known to be underexpressed in *Rai1*-deficient brains—including *Cdh12*, *Pcdh7*, *Pcdh11x*, *Pcdh17*, and *Gabra1*—were upregulated by rAAV8-sg2 treatment (Fig. 2*I*) (11). Moreover, both RNA-sequencing and qRT-PCR found that rAAV8-sg2 treatment significantly increased the expression of *Bdnf*, a key Rai1 direct target gene (Fig. 2*I*). Conversely, many genes known to be overexpressed in *Rai1*-deficient brains including *Hspg2*, *Igf2*, *Nos1*, *Slc16a1*, and *Ttr* (11) were downregulated by rAAV8-sg2 treatment (Table S5). Together, these data suggest that while we cannot completely rule out potential off-target effects, rAAV8-sg2 treatment activates *Rai1* and its downstream targets including *Bdnf* and cell adhesion molecules, consistent with the known function of *Rai1*.

### rAAV8-sg2 treatment increases Rai1 levels with minimal immunogenicity *in vivo*

**Figure 3:**
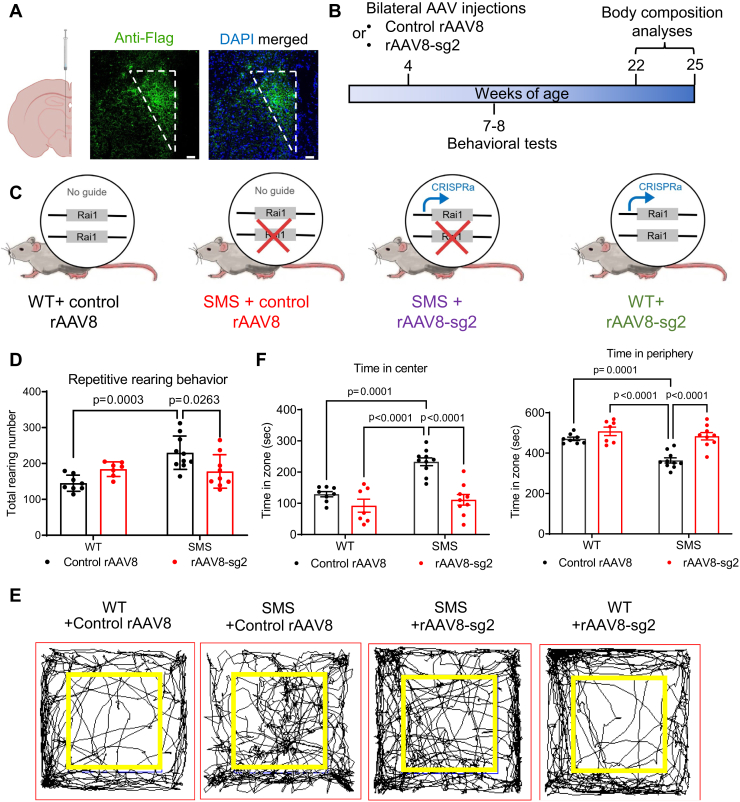
**PVH-specific administration of rAAV8-sg2 particles rescues selective SMS-like neurobehavioral features in mice.***A*, *left*: schematic depicting stereotaxic delivery of rAAV-CRISPRa particles into the PVH. *Right*: immunostaining confirming the expression of FLAG-tagged sadCas9 (in *green*) in the PVH. Scale bars represent 50 μm. *B*, schematic depicting the experimental procedures. *C*, schematic representation of the four different experimental and control groups. *D*, SMS mice treated with control rAAV8 show increased repetitive rearing behavior, which was reversed by rAAV8-sg2 treatment. Two-way ANOVA with Tukey’s post-hoc test. Genotype: F (1, 30) = 8.996, *p* = 0.0054; Treatment: F (1, 30) = 0.2457, *p* = 0.6237; Genotype and treatment interaction: F (1, 30) = 12.15, *p* = 0.0015. (*E*) Trace maps of mouse activity in the open field test. *F*, quantification of time spent in the center (*left*) and peripheral (*right*) of the open field for each group. Two-way ANOVA with Tukey’s post-hoc test. Time in center: Genotype: F (1, 30) = 16.59, *p* = 0.0003; Treatment: F (1, 30) = 27.72, *p* < 0.0001; Genotype and treatment interaction: F (1, 30) = 7.910, *p* =0.0086. Time in periphery: Genotype: F (1, 30) = 17.68, *p* = 0.0002; Treatment: F (1, 30) = 25.17, *p* < 0.0001; Genotype and treatment interaction: F (1, 30) = 7.028, *p* = 0.0127. The data are shown as means ±SD. *p*-values for the post-hoc tests are indicated in the figure. CRISPRa, clustered regularly interspaced short palindromic repeats activation; PVH, paraventricular nucleus of hypothalamus; rAAV, recombinant adeno-associated virus; SMS, Smith-Magenis syndrome.

To test if rAAV particles can be stably expressed in PVH neurons *in vivo*, we stereotaxically injected rAAV8 into the PVH of 4-week-old WT mice. Immunostaining confirmed that FLAG-sadCas9 is continuously expressed in the PVH even 5 months after injections (Fig. 3*A*). We determined the distribution and variability of rAAV8 expression by performing immunostaining using an anti-FLAG antibody in six mice (Fig. S2*A*–*F*). In all mice, we found that rAAV8 was expressed in the PVH but not in nearby hypothalamic nuclei such as the arcuate nucleus (Fig. S2*A*–*F*). To identify the proportion of specific PVH cell types that received rAAV8, we measured the co-expression of FLAG, DAPI, and cell type-specific markers, including NeuN (neuron), GFAP (astrocyte), vasopressin (AVP), corticotropin releasing factor receptor (CRF), melanocortin-4 receptor (MC4R), and oxytocin (Fig. S3*A*). We found that 60% of DAPI^+^ cells within the PVH express FLAG-sadCas9 (Fig. S3*B*), among which 71% are NeuN^+^ neurons and 26% are GFAP^+^ glial cells (Fig. S3*C*). We also found that rAAV8 targets 30.9% of AVP^+^ PVH neurons, 33.4% of CRF^+^ PVH neurons, 44.7% of MC4R^+^ PVH neurons, and 19% of oxytocin^+^ PVH neurons (Fig. S3*D*). These data suggest that rAAV8 targets multiple PVH neuronal subtypes.

We then performed qRT-PCR using dissected hypothalamic tissues and found that endogenous *Rai1* mRNA levels in the PVH of SMS mice were significantly elevated (Fig. S4*A*). Consistent with our previous results that showed *Bdnf* as an Rai1 direct target gene (11), qRT-PCR found that *Bdnf* mRNA levels in SMS mice treated with control rAAV8 were 42% lower than WT mice treated with control rAAV8 (Fig. S4*B*). By contrast, *Bdnf* mRNA levels in SMS mice treated with rAAV8-sg2 increased to 81% of the levels in WT mice treated with control rAAV8 (Fig. S4*B*); this suggests that *Bdnf* expression in PVH was partially rescued by rAAV8-sg2. Furthermore, we found the expression levels of predicted sg2 off-target genes and *Rai1* surrounding genes did not differ among hypothalamic tissues dissected from WT + control rAAV8, SMS + control rAAV8, and SMS+rAAV8-sg2 groups (Fig. S4*C*–*H*). This is consistent with our data in primary neurons and suggests that rAAV8-sg2 does not significantly increase the expression of predicted sg2 off-target genes *in vivo*.

Prolonged rAAV expressions have been shown to trigger host immune responses (30, 31). We performed qRT-PCR using the liver tissues of 6-month-old mice with early adolescent PVH-specific CRISPRa gene therapy and found that sustained control rAAV8-sg2 expression for 5 months did not trigger overexpression of proinflammatory cytokines including interleukin-6 and tumor necrosis factor alpha (Fig. S5*B*). By contrast, intraperitoneal injections of lipopolysaccharide (LPS) purified from *Escherichia coli* induced dramatic overexpression of proinflammatory cytokines in the liver (Fig. S5*B*). Next, we measured serum levels of aspartate aminotransferase (AST) and alanine aminotransferase (ALT). Mice with PVH-specific rAAV8-sg2 treatment showed similar AST and ALT levels to saline-injected control mice (Fig. S5*C* and *D*). By contrast, mice with intraperitoneal LPS injections showed dramatically increased AST and ALT levels (Fig. S5*C* and *D*). These data suggest that PVH-specific rAAV8-sg2 injections did not significantly induce hepatic inflammation. To further study if rAAV8-sg2 expression induces local inflammations in the PVH, we performed immunofluorescence staining using anti-Iba1 and anti-CD68 antibodies to identify activated microglia. We found that mice injected with either saline or rAAV8-sg2 showed similar number of Iba1^+^/CD68^+^ microglial cells (Fig. S5*E* and *F*). By contrast, mice that received PVH-specific LPS injections showed significantly increased Iba1^+^/CD68^+^ microglial cells (Fig. S5*E* and *F*). Together, these data suggest that chronic rAAV8-sg2 expression in the PVH does not elicit hepatic or local inflammation.

### PVH-specific rAAV8-sg2 treatment rescues stereotypical rearing behavior in SMS mice

Two core neurobehavioral features in SMS mice are increased repetitive rearing in the open field test and reduced social dominance in tube test (4, 5, 32). To test if PVH-specific rAAV-CRISPRa therapy could modify these neurobehavioral features in SMS mice, we bilaterally injected 4-week-old WT and SMS mice with either control rAAV8 or rAAV8-sg2 in the PVH (Fig. 3, *B* and *C*) and monitored their neurobehavioral functions at 7 to 8 weeks of age. While all groups of mice show similar travel distance (Fig. S6*A*) and movement speed (Fig. S6*B*) in the open field, SMS mice treated with control rAAV8 show increased repetitive rearing behavior compared to control rAAV8 treated WT mice (Fig. 3*D*). This is consistent with previous findings (4, 5). WT and SMS mice did not show different self-grooming behaviors (Fig. S6*C*), suggesting that *Rai1* haploinsufficiency affects neural circuits mediating repetitive rearing but not repetitive self-grooming. Intriguingly, we found that rAAV8-sg2 treatment normalizes excessive repetitive rearing in the SMS mice (Fig. 3*D*). Specifically, SMS mice treated with control rAAV8 show a significantly increased number of rearing episodes in the center of the open field (Fig. S6*D*), which was normalized by rAAV8-sg2 treatment (Fig. 3, *E* and *F*). While SMS mice spent more time in the center, this was not due to reduced anxiety because WT and SMS mice treated with control rAAV8 show similar anxiety levels in the elevated plus maze (Fig. S6*E*), consistent with our previous findings that indicate *Rai1* loss does not induce anxiety in mice (11). Treating WT mice with rAAV8-sg2 did not affect their rearing or exploratory patterns (Fig. 3, *D*–*F*). In addition, PVH-specific rAAV8-sg2 treatment did not significantly alter social dominance deficits in SMS mice (Fig. S6*F*). On the basis of these data, we conclude that PVH-specific rAAV8-sg2 treatment rescues stereotypical repetitive behaviors in SMS mice.

### PVH-specific rAAV-CRISPRa-sg2 treatment delays the onset of and partially rescues SMS-like obesity

**Figure 4:**
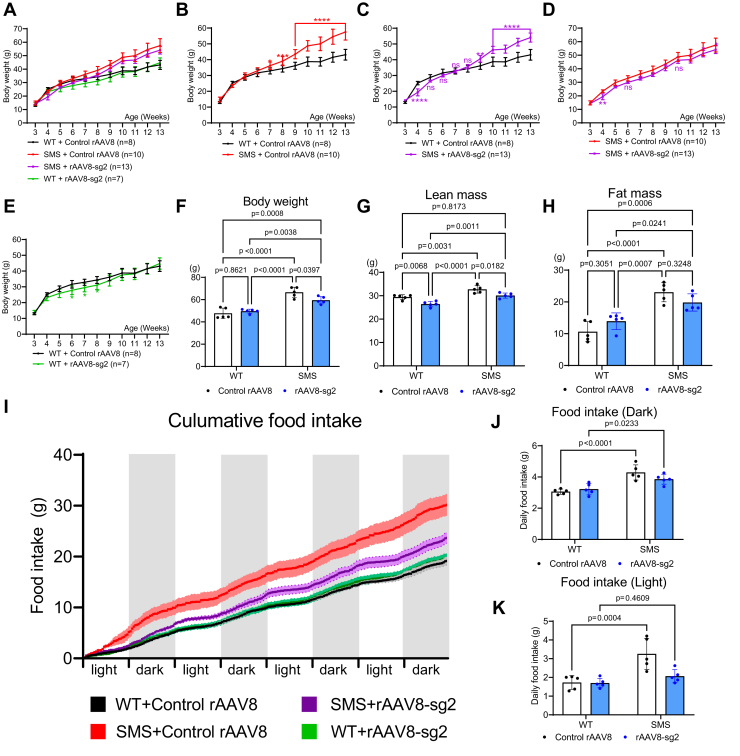
**PVH-specific administration of rAAV8-sg2 particles partially rescues SMS-like obesity in SMS.***A*, body weight of WT and SMS mice with control rAAV8 or rAAV8-sg2 treatment, which are separately illustrated in (*B*–*E*) to facilitate visualization. The data are shown as means ±SD. *F*, body weight of mice at 25 weeks of age. Genotype: F (1, 16) = 72.66, *p* < 0.0001; Treatment: F (1, 16) = 2.419, *p* = 0.1394; Genotype and treatment interaction: F (1, 16) = 7.072, *p* =0.0171. *G*, rAAV8-sg2 treatment decreases lean mass (Genotype: F (1, 16) = 40.38, *p* < 0.0001; Treatment: F (1, 16) = 26.18, *p* =0.0001; Genotype and treatment interaction: F (1, 16) = 0.1197, *p* = 0.7339) but not fat mass (*H*, Genotype: F (1, 16) = 50.44, *p* < 0.0001; Treatment: F (1, 16) = 0.0009619, *p* = 0.9756; Genotype and treatment interaction: F (1, 16) = 6.389, *p* = 0.0224) in SMS mice. SMS mice treated with rAAV8-sg2 show decreased cumulative food intake (*I*, shades represent ±SEM) due to reduced food intake in dark phase (*J*, Genotype: F (1, 16) = 34.96, *p* <0.0001; Treatment: F (1, 16) = 0.7447, *p* = 0.4009; Genotype and treatment interaction: F (1, 16) = 3.573, *p* =0.077) but not light phase (*K*, Genotype: F (1, 16) = 17.67, *p* = 0.0007; Treatment: F (1, 16) = 7.329, *p* = 0.0155; Genotype and treatment interaction: F (1, 16) = 6.621, *p* = 0.0204). The data are shown as means ±SD. Two-way ANOVA with Tukey’s post-hoc test. *p*-values for the post-hoc tests are indicated in the figure. n.s. indicates not significantly different. PVH, paraventricular nucleus of hypothalamus; SMS, Smith-Magenis syndrome.

Truncal obesity associated with excessive food intake is a debilitating feature in SMS patients and mouse models (7, 13, 33). Our body weight analyses showed that SMS mice treated with control rAAV8 become significantly overweight by 7 weeks of age when compared to WT mice treated with control rAAV8 (Fig. 4, *A* and *B*, body weight data in **A** are separately illustrated in **B–E** to facilitate visualization). Intriguingly, between 5 and 8 weeks of age, SMS mice treated with rAAV8-sg2 show similar body weight to their WT littermates treated with control rAAV8 (Fig. 4*C*). SMS mice treated with rAAV8-sg2 become significantly more obese than WT controls at 9 weeks of age (Fig. 4*C*), suggesting that rAAV8-sg2 treatment delays the onset but does not fully reverse obesity in SMS mice. Consistent with a long-lasting rAAV expression, SMS mice treated with rAAV8-sg2 continue to show a modest but significant lower weight compared to SMS mice treated with control rAAV8 even at 25 weeks of age (Fig. 4, *D* and *F*). We calculated the percent change in body weight between 3 and 25 weeks of age and found that WT mice treated with control rAAV8 gained 267% of body weight and SMS mice treated with control rAAV8 gained 350% of body weight during this period. By contrast, SMS mice treated with rAAV8-sg2 gained 314% body weight by 25 weeks of age. SMS mice treated with control rAAV8 showed increased serum levels of leptin and high-density lipoprotein (HDL), but no statistically significant change in low- and very low-density lipoproteins (LDL + VLDL) (Fig. S7*A*–*C*). By contrast, the leptin and HDL levels of SMS mice treated with rAAV8-sg2 did not differ with WT or SMS mice treated with control rAAV8, suggesting that these parameters were not rescued. Together, this suggests that PVH-specific rAAV8-sg2 treatment delays the onset of obesity and partially mitigated excessive body weight gain in SMS mice. Previous studies found that mice that carry globally expressed *Rai1* transgenes or a genomic duplication syntenic to the human PTLS critical regions show a transient underweight phenotype (34, 35). We found that WT mice treated with rAAV8-sg2 show a significantly lower body weight compared to WT mice treated with control rAAV8 during 6 weeks of age (Fig. 4*E*). This suggests that Rai1 overexpression in PVH may contribute to transient weight loss observed in PTLS mice.

Next, we measured body composition using Echo-magnetic resonance imaging and found that SMS mice treated with control rAAV8 show significantly increased lean mass and fat mass compared to WT mice treated with control rAAV8 (n = 5/group, Fig. 4, *G* and *H*). Interestingly, the excessive gain of lean mass but not fat mass in SMS mice fully normalizes to WT levels by rAAV8-sg2 treatment (Fig. 4, *G* and *H*). Comprehensive lab animal monitoring system (CLAMS) analysis found that WT and control mice treated with control rAAV8 show similar respiratory exchange ratio (RER) during light and dark cycles (Fig. S8*A* and *B*). Interestingly, rAAV8-sg2 treatment increases RER in WT but not SMS mice during dark phase (Fig. S8*A* and *B*). We also found that all groups of mice show similar energy expenditure (EE) ratio at light or dark phases regardless of treatment (Fig. S8*C* and *D*), consistent with our previous findings that Rai1 loss did not alter EE (11). Instead, SMS mice treated with control rAAV8 show increased food intake when compared to WT mice treated with control rAAV8 (Fig. 4, *I*–*K*). By contrast, SMS mice treated with rAAV8-sg2 show a partially rescued food intake in the dark phase when compared to SMS mice treated with control rAAV8 (Fig. 4, *I*–*K*). WT mice treated with rAAV8-sg2 did not show significantly altered food intake (Fig. 4, *I*–*K*). Overall, these findings indicate that PVH-specific rAAV8-sg2 treatment in SMS mice is associated with reduced food intake.

## Discussion

Genetic haploinsufficiency in more than 660 genes results in monoallelic expression that is insufficient to support normal physiology and causes genetic disorders (36, 37). Many of these disorders, including SMS, are incompatible with gene replacement therapy because the length of disease-causing genes exceeds the payload capacity of AAVs. A promising alternative strategy is transcriptional targeting of the endogenous cis-regulatory elements of haploinsufficient genes (25, 28, 37, 38), which in principle should benefit SMS patients either carrying *Rai1* point mutations or 17p11.2 deletions. Here, we show that mouse *Rai1* proximal promoter is accessible to CRISPRa-mediated *Rai1* overexpression *in vitro* and *in vivo*. Importantly, localized administration of rAAV-CRISPRa in the PVH rescues repetitive rearing behavior and partially corrects obesity in SMS mice.

A critical consideration for treating SMS is to specifically boost *Rai1* expression in brain region(s) relevant to disease. Our work shows that neurobehavioral features and obesity in SMS mice are caused by Rai1 loss in subcortical but not cortical glutamatergic neurons (11). To avoid affecting the cortex, we stereotaxically targeted PVH and found that rAAV-CRISPRa gene therapy increases *Rai1* expression levels and rescues excessive repetitive rearing, a cardinal feature of SMS mice. This finding also corroborates with the known role for CRF^+^ PVH neurons in mediating repetitive rearing behavior (39). By contrast, PVH-specific *Rai1* gene therapy did not rescue SMS-like social dominance deficits. This can be explained by our previous findings that indicate social dominance deficits in SMS mice involve multiple cell types in different brain regions and can only be rescued by globally increasing *Rai1* expression, not only in glutamatergic or GABAergic neurons (5). A future direction is to identify additional brain regions responsible SMS-like features and evaluate if simultaneously increasing *Rai1* expression levels in multiple brain regions can further modify disease progression.

We previously show that postnatal *Rai1* deletion from PVH was sufficient to induce SMS-like obesity (13), suggesting that *Rai1* expression in the postnatal brain mediates food intake. Here we show that PVH-specific *Rai1* activation delays the onset of obesity and partially rescues excessive weight gain and food intake. The PVH is a complex brain region that contains multiple neuronal subtypes defined by expression of different neuropeptides, growth factors, and their receptors (40). Each PVH neuronal subtype shows unique projection patterns that differentially regulate brain function and metabolism (14). We previously reported that deleting *Rai1* using a *Sim1-Cre* allele, which targets all PVH neuronal subtypes, increases lean and fat mass in mice (11). Here, we show that CRISPRa-mediated *Rai1* induction in the PVH of SMS mice selectively rescues lean but not fat mass. Because only subsets (19–44%) of AVP^+^, CRF^+^, MC4R^+^, and oxytocin^+^ neurons were targeted by rAAV8-sg2, the rescue effect could result from incomplete Rai1 restoration in all PVH neurons. Moreover, the neural mechanism of how each PVH subtype interacts with the connected brain regions to regulate energy metabolism is only beginning to be understood. For example, deleting *MC4R* using *Sim1-Cre* induces increased fat mass but not lean mass, while rescuing *MC4R* reexpression in PVH reduces both lean and fat mass (41). An alternative explanation is that increasing *Rai1* expression in MC4R^+^ neurons rescues lean mass, while other PVH neuronal subtypes mediating increased fat mass were not sufficiently targeted. Future work that delineates the contribution of different PVH neuronal subtypes in SMS-like features will likely further inform therapeutic efficacy.

Our recent work found that PVH-specific *Bdnf* overexpression during early adolescence fully reverses obesity in SMS mice (13), suggesting that postnatal manipulation of the Bdnf signaling could be more effective than increasing Rai1 for treating obesity. To avoid triggering systemic inflammatory toxicities associated with systemic rAAV injection (18, 42, 43, 44), we locally administered rAAV and observed minimal inflammatory responses in the liver, locally in PVH, and serum even after 5 months of rAAV treatment. Future work that miniaturizes the Cas9 protein and reduces its immunogenicity will further enable the clinical applicability of rAAV/CRISPRa therapy.

Identification of off-target effects remains an important and challenging aspect of CRISPR gene therapy. In this study, AAV8-sg2 treatment *in vivo* did not induce ectopic expression of predicted sg2 off-target genes. We showed that rAAV8 targets 60% of PVH, suggesting that microdissection unavoidably included cells not targeted by rAAV8. Due to these limitations, we focused our analysis of sg2 off-target effect on primary neuronal cultures that have higher rAAV targeting efficiency. We found that rAAV8-sg2 treatment *in vitro* induced the expression of *Rai1* and many of its downstream target genes including *Bdnf* and cell adhesion molecules. We cannot exclude potential off-target effects because many Rai1 target genes also regulate gene expression; however, mice that received rAAV8-sg2 treatment did not show noticeable behavioral and physiological abnormalities. Future work is needed to fully understand the extent of off-target effects caused by rAAV-CRISPRa therapy from molecular to organismal levels.

Mice carrying extra copies of *Rai1* or PTLS-like duplications are underweight and exhibit increased social tube dominance behavior and decreased repetitive rearing (34, 45, 46). PTLS mice also show increased respiratory exchange ratio (33). Here we show that WT mice treated with rAAV-CRISPRa in the PVH showed a transient underweight and increased respiratory exchange ratio, consistent with toxicity associated with *Rai1* overexpression. These results highlight the importance of developing strategies to prevent toxicity associated with *Rai1* overexpression. Given that *Rai1* overexpression during early development is more toxic than adulthood (46), it remains to be determined if overexpressing Rai1 at earlier timepoints or outside of PVH will induce other PTLS-like features.

Mounting evidence suggests that early rather than later interventions are more effective for treating neurodevelopmental disorders (11, 47). For example, rAAV-CRISPRa-mediated *Scn1a* activation at 4 weeks of age did not fully rescue epilepsy in mouse models of Dravet syndrome (23, 48). Realistically, early preventive treatments are not always possible because SMS is not commonly diagnosed in utero (49, 50). Our results are encouraging because rAAV-sg2 treatment was therapeutically effective for 1-month-old mice, which are equivalent to 14-year-old early adolescent human (51). Given that gRNA target sites have stringent requirements (52), future work is needed to evaluate *Rai1* promoter accessibility across therapeutic timepoints and different brain regions. Most importantly, sgRNAs that target human *RAI1* promoter could have distinct functionality, off/on-target effects, and tolerability, which should be independently evaluated to better inform clinical study design.

## Experimental procedures

### Mouse procedures

All procedures were performed in accordance with the guidelines of the Canadian Council on Animal Care and the Montreal General Hospital Facility Animal Care Committee, with the appropriate approved protocols for animal use. Mice were housed in groups on a 12-h light/12-h dark cycle with *ad libitum* access to food and water. SMS mice were generated as previously described (13). All mouse experiments were conducted using F1 hybrids of CD1 and C57BL/6J parents.

### Cell cultures

Mouse neuro2a cells (American Type Culture Collection, CCL-131) were cultured in Dulbecco’s Modified Eagle’s Medium with GlutaMAX Supplement (Gibco) supplemented with nonessential amino acids, 10% fetal bovine serum, and 1% penicillin/streptomycin. Cells were split every 2 to 3 days using 0.25% trypsin (Gibco). For transfection, Lipofectamine 3000 (Thermo Fisher Scientific) was used according to the manufacturer’s instructions. For primary hippocampal cultures, hippocampi were harvested from P0 mice in dissection media (in 100 ml: 1 ml of 100× sodium pyruvate, 0.5 ml of 20% glucose, 98.5 ml of Ca/Mg free HBSS), triturated with trypsin (in 37 °C for 17 min), treated with 1% DNAse I for another 3 min, mechanically dissociated, and seeded into 6-well-plates (5 × 10^5^ cells/well) in plating media (in 100 ml: 10 ml of heat-inactivated horse serum, 2.25 ml of 20% glucose, 1 ml of 100× sodium pyruvate, 1 ml of 100× GlutaMAX, 1 ml of Pen/Strep (10,000 U/ml), and fill volume to 100 ml with Dulbecco’s Modified Eagle’s Medium). Three hours after plating, media were changed to maintenance media (in 100 ml: 2 ml of B27 supplements, 1 ml of 100× GlutaMAX, 1 ml of Pen/Strep (10,000 U/ml), and fill with Neurobasal Medium to 100 ml). At DIV2, 1 μM Ara-C was added to inhibit the replication of non-neuronal cells. For primary neurons, 3.25 × 10^10^ genome copies of control rAAV8 and rAAV8-sg2 were administered.

### CRISPRa engineering and administrating rAAV particles

The sgRNA oligonucleotides targeting mouse *Rai1* promoter regions were designed using the Benchling gRNA Design Tool (53). The sadCas9-2 × VP64 vector (Addgene #135338) (25) carries mutations in the endonuclease catalytic residues (D10A, N580A) of a FLAG-tagged saCas9, which was fused on both N and C termini with transcriptional activators VP64 (four copies of VP16). To clone the sgRNAs into the vector, 4 μg of vectors were digested with BsaI enzyme for overnight at 37 °C, followed by PCR purification and dephosphorylation using alkaline phosphatase. The T4 PNK enzyme and T4 ligation buffer were used to anneal the sgRNA oligonucleotides (95 °C for 5 min and then ramp down to 25 °C at 5 °C/min). After T4 DNA ligase-mediated ligation and bacterial transformation (One Shot Stbl3, Invitrogen), colonies were harvested, and vectors were extracted for Sanger sequencing. Plasmids were then packaged into the rAAV8 capsid by Vector Biolabs. To administer the rAAVs into the PVH region, 4-week-old WT and SMS mice were anesthetized with isoflurane (1–2%) and ear-barred onto a stereotaxic instrument (David Kopf). An incision at the midline of the scalp was made to expose the skull, and two holes were made with a microdrill (Stoelting) above the injection sites. For PVH injections, 3.25 × 10^9^ genome copies of control rAAV8 or rAAV8-sg2 viral particles were delivered bilaterally into the PVH using the following coordinates relative to Bregma: −0.8 mm anteroposterior; ± 0.25 mm mediolateral; and −5.0 mm dorsoventral.

### RAI1 deletion editing using CRISPR/Cas9

To generate human 293A cells carrying *RAI1* deletions, we took a dual sgRNA approach to ensure robustness of knockout of exon three that encodes >95% of *RAI1*’s open reading frame. Specifically, we used two guide pair (GP) sequences: GP1 5′-GAGGCTCCGGCAGAGCCCGG-3′ and 5′-TCGCTTCTCCACCCGCACGA-3′ or GP2 5′-CAAACATGAGTGCAGCAAGG-3′ and 5′-AGGGCCCACAGAGGTCCCCA-3′. We cloned guide pair sequences into a pCLIP dual vector and cotransfected with a Lenti-Cas9-Blast vector into 293A cells followed by puromycin selection (1 μg/ml) for 3 days. We pelleted polyclonal populations for a first stage PCR test for the presence of the KO band in the heterogeneous mixture. After confirming the deletions can occur, we performed single cell sorting and screened monoclonal populations for deletion.

### Immunostaining, immunoblotting, and enzyme-linked immunosorbent assay

Immunostainings of mouse brains were done as previously described (11). Briefly, mouse brains were either (1) rapidly dissected and mounted in Optimal Cutting Temperature compound (ThermoFisher) and then mounted or (2) perfused with 4% paraformaldehyde, cryopreserved in 30% sucrose in PBS, and then mounted. Sections were prepared on a cryostat (Leica), washed in a PBS solution, and sections from freshly dissected brains were incubated for 10 min at −20 °C in precooled acetone, washed again in PBS, and then blocked for 2 h at room temperature in 10% normal donkey serum (NDS) in PBS. For immunostaining of cultured hippocampal neurons, neurons were seeded onto poly-D-lysine–coated coverslips and fixed in precooled acetone for 10 min at −20 °C. Samples were further incubated overnight at 4 °C with antibodies in 10% NDS/PBS. The next day, the slides were incubated for 2 to 3 h at room temperature with secondary antibodies in 10% NDS in PBS and coverslipped in DAPI-containing Fluoromount-G (Southern Biotech). Primary antibodies used in this work include mouse anti-FLAG (Abcam ab95045), rabbit anti-NeuN (Abcam ab177487), chicken anti-GFAP (Abcam ab4674), rabbit anti-oxytocin (Sigma AB911), rabbit anti-MC4R (Abcam ab24233), rabbit anti-CRF (Abcam ab272391), mouse anti-Iba1 (Millipore-Sigma MABN92-AF488), and rabbit anti-CD68 (Abcam ab213363).

Western blot analysis was used to quantify protein levels. Protein was extracted and separated by SDS-PAGE gel and then transferred onto nitrocellulose membranes. Membranes were cut into two portions, blocked in 5% milk in tris-buffered saline (TBS) for 1 h, and incubated at 4 °C with anti-Rai1 antibody (1:500, Abcam ab96599) or anti-Gapdh antibody (1: 10,000, Abcam ab9485) in TBS containing 0.1% Tween 20 with 2% milk overnight. The next day, membranes were washed with TBS containing 0.1% Tween 20 and incubated with secondary antibodies conjugated with horseradish peroxidase (ThermoFisher). Finally, membranes were incubated with ECL substrates and then imaged using a Bio-Rad ChemiDoc imager.

We used mouse AST and ALT ELISA kits to measure serum levels of AST and ALT, following the manufacturer’s manual (Abcam ab263882 and ab282882).

### RNA-sequencing and quantitative reverse transcription polymerase chain reaction

Total RNA was extracted by TRIzol reagent and phenol-chloroformisoamyl alcohol (ThermoFisher) and reverse-transcribed with the SuperScript III First-Strand Synthesis System (ThermoFisher). Quantitative PCR reactions were conducted using SsoFast EvaGreen Supermix on a Bio-Rad qPCR system. Primer sequences can be found in Table S3. We prepared the RNA sequencing libraries using the NEBNext Ultra II RNA Library Prep Kit for Illumina following manufacturer’s instructions (New England Biolabs). Briefly, mRNAs were initially enriched with Oligo(dT) beads. Enriched mRNAs were fragmented for 15 min at 94 °C. First strand and second strand cDNA were subsequently synthesized. cDNA fragments were end repaired and adenylated at 3′ends, and universal adapters were ligated to cDNA fragments, followed by index addition and library enrichment by PCR with limited cycles. The sequencing libraries were validated on the Agilent TapeStation (Agilent Technologies) and quantified using Qubit 2.0 Fluorometer (Thermo Fisher Scientific) as well as by quantitative PCR (KAPA Biosystems). The sequencing libraries were clustered on three flowcell lanes. After clustering, the flowcell was loaded on an Illumina HiSeq instrument according to manufacturer’s instructions. The samples were sequenced using a 2 × 150 bp paired-end configuration, yielding 58 to 104 million reads per sample (n = 3 per group). Image analysis and base calling were conducted by the Control software. Raw sequence data (.bcl files) generated from the sequencer were converted into fastq files and de-multiplexed using Illumina's bcl2fastq 2.17 software.

### RNA-sequencing data analysis

Sequence reads were trimmed to remove adapter sequences and nucleotides with poor quality using Trimmomatic v.0.36. The trimmed reads were mapped to the *Mus musculus* GRCm38 reference genome available on ENSEMBL using the STAR aligner v.2.5.2b, which detects splice junctions and incorporates them to help align the entire read sequences. On average, more than 91.86% of the trimmed sequences were mapped to the reference genome. Unique gene hit counts were calculated using featureCounts from the Subread package v.1.5.2. Only unique reads that fell within exon regions were counted. Genes with an adjusted *p*-value < 0.05 and absolute log_2_ fold change > 1 were called as differentially expressed genes (DESeq2). Significantly differentially expressed genes were clustered by their gene ontology, and the enrichment of gene ontology terms was tested using Fisher exact test (GeneSCF v1.1-p2). The goa_mouse GO list was used to cluster the set of genes based on their biological processes and determine their statistical significance.

### Behavioral tests

#### Open field

Mice were placed for 10 min in the center of a 45 cm (W) × 45 cm (D) × 40 cm (H) square arena and allowed to move freely. Mice were taken into the testing room and allowed to habituate for 1 h prior to testing. Parameters including distance moved, velocity, vertical rearing, self-grooming, and times spent in predefined zone of the arena were recorded and analyzed by experimenters blinded to mouse genotypes.

#### Tube test

Mice were housed in cages in the testing environment for 1 day before training. On each of two training days, each mouse passed through the tube for 10 trials (five times from each side, without opponents), which helped familiarize the mice with walking through the tube and knowing that the tube was safe. On the test days, two mice of different genotypes were placed at the two ends of the tube and released simultaneously to meet in the middle of the tube. The mouse that retreated first from the tube was designated as the loser. To avoid measuring social hierarchy established between cage mates, the animals used for the tube test were housed with littermates of the same genotype/treatment and encountered unfamiliar mice of differing genotype/treatment in the tube test.

#### Elevated plus maze test

The maze is shaped as a large plus (60 mm (W) × 295 (D) mm) with two arms flanked by tall covers (elevation 150 (H) mm) and the remaining arms flanked only by 18 (H) mm boarder. The testing apparatus is elevated 400 mm off the ground by means of plastic columns at the end of each arm. Mice were brought into the room in a covered cage cart and allowed to habituate for 1 h. They were then individually placed in the center square region of the maze facing away from the investigator. Mice were given 5 min to explore the novel environment. Duration of time mice spent in each arm of the maze was recorded using a ceiling-mounted camera and computer-interfaced video tracking system (SMART3) and subsequently analyzed.

### Mouse food intake and EE analyses

Chow-fed control and SMS mice with either control rAAV8 or rAAV8-sg2 injected in the PVH at 4 weeks of age were used for metabolic profiling around 22 to 25 weeks of age. The RER and EE were measured using indirect calorimetry in the metabolic cages of a CLAMS (Columbus Instruments). Animals were singly housed in the CLAMS apparatus at 21 °C (70 °F) in a light-dark cycle matching their housing conditions for 4 days. EE was normalized by lean mass. Total fat and lean mass were assessed using a nuclear echo magnetic resonance imaging whole-body composition analyzer. A fluorimetric assay kit (cat# ab65390, Abcam) was used for the measurement of serum HDL and LDL/VLDL levels. The kit was used according to the manufacturer's instructions. Fluorescence at 535/587 nm (Ex/Em) was recorded using the Ensight instrument (PerkinElmer). A fresh standard curve (0 and 10 μg/ml) was made for each microplate to be able to precisely quantify HDL and LDL/VLDL cholesterol levels of serum samples. Standards and samples were loaded and analyzed in duplicate.

## Statistics

All data were statistically analyzed using GraphPad Prism 9 software, and *p*-values less than 0.05 were considered significant. The levels of significance are indicated as follows: ∗< 0.05, ∗∗< 0.01, ∗∗∗< 0.001, and ∗∗∗∗< 0.0001. Statistical analysis was performed using Student’s *t* test or one- or two-way analysis of variance (ANOVA) with post-hoc correction for multiple comparisons.

## Data availability

RNA-sequencing data (BAM files) have been deposited at the NCBI Sequence Read Archive (SRA). Accession number: PRJNA888480. https://dataview.ncbi.nlm.nih.gov/object/PRJNA888480?reviewer=ag3c5m2t8jchofqvba43847791.

## Supporting information

This article contains supporting information.

## Conflict of interest

The authors declare no conflicts of interests with the contents of this article.
