# Supplementary material for: rAAV-CRISPRa therapy corrects Rai1 haploinsufficiency and rescues selective disease features in Smith-Magenis syndrome mice
Source: J Biol Chem. 2022 Nov 19;299(1):102728. doi: 10.1016/j.jbc.2022.102728 (PMC9762195; doi:10.1016/j.jbc.2022.102728)
Supplement: Supplemental Figures and Tables [file mmc1.docx]

**Supporting information**

**rAAV-CRISPRa therapy corrects *Rai1* haploinsufficiency and rescues selective disease features in Smith-Magenis syndrome mice**

Hao-Cheng Chang, Yu-Ju Lee, Sehrish Javed, Minza Haque, Ya-Ting Chang, Yu Cheng Lin, Cameron Oram, Wei-Hsiang Huang

**This file contains:**

**Supplemental Figures S1-S8**

**Supplemental Tables S1-S5**

**
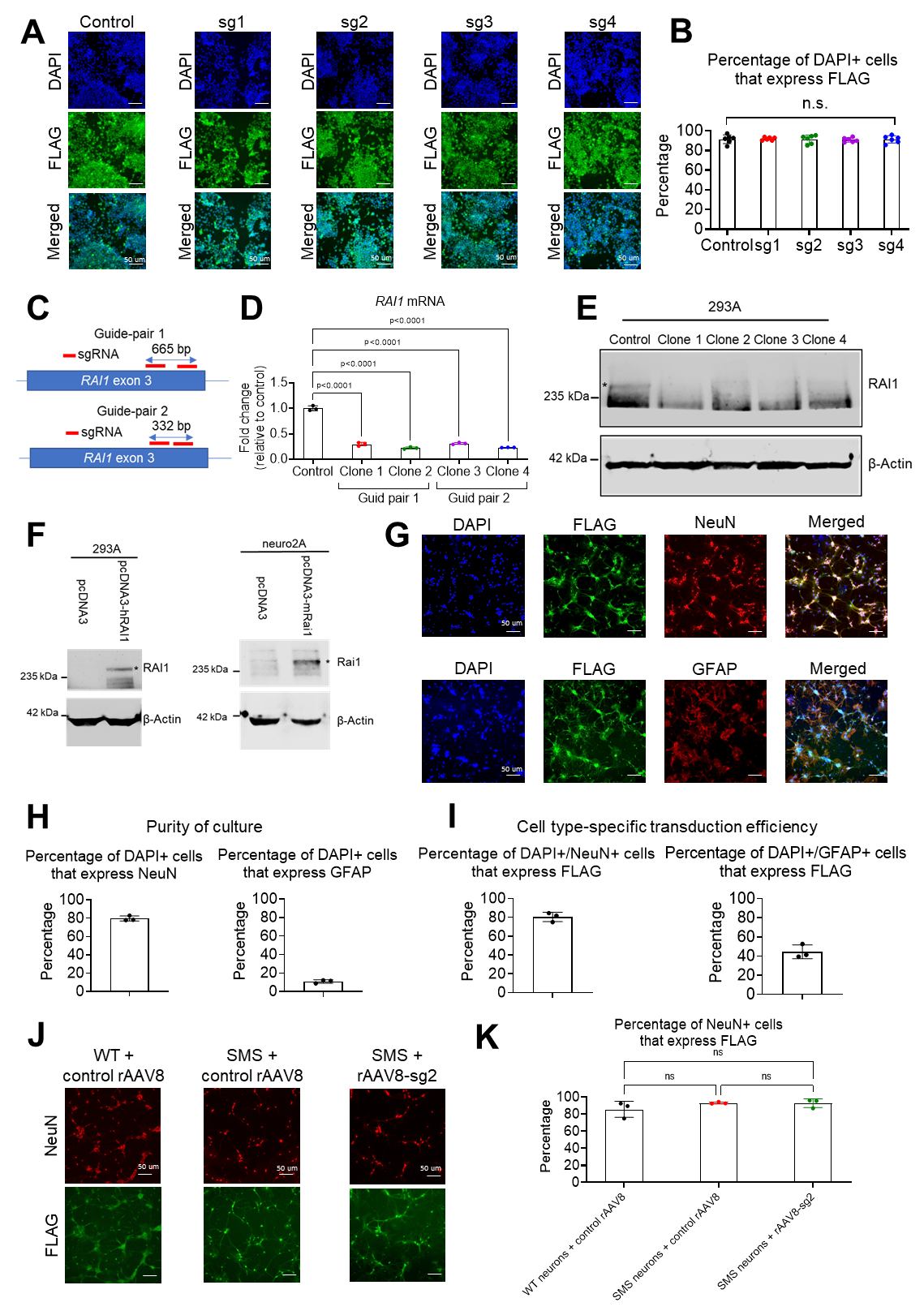
**

**Figure S1. Validation of RAI1 antibody, transduction efficiency, and neuronal purity.**

(**A**) Representative immunofluorescence staining images showing neuro2a cells transfected with control or sg1-4 plasmids. All plasmids express FLAG-tagged sadCas9, as shown by FLAG staining in green. Nuclei are indicated by DAPI staining in blue.

(**B**) Quantification showing similar transfection efficiencies in neuro2a cells transfected with control or sg1-sg4 plasmids (90-92% of DAPI^+^ cells express FLAG-sadCas9). Data obtained from 3 independent transfections per plasmid, two images per transfection. One-way ANOVA with Tukey’s post-hoc test, n.s. indicates not significantly different.

(**C**) Schematic illustration of two independent guide pairs of sgRNAs (guide-pairs 1 and 2) used to delete the exon 3 of human *RAI1* gene.

(**D**) Quantitative RT-PCR data showing significantly decreased human *RAI1* mRNA levels in four different clones carrying *RAI1* deletions when compared to a control cell line (mean±SD, one-way ANOVA with Dunnett’s post-hoc test, F (4, 10) = 386.0, p<0.0001). P-values for the post-hoc tests are indicated in the figure. Two independent clones per guide pair.

(**E**) Western blotting assays using an anti-RAI1 antibody showing near-complete loss of RAI1 protein (indicated by an asterisk) in four independent clones carrying *RAI1* deletions.

(**F**) Western blotting data using an anti-RAI1 antibody detects the overexpression of human RAI1 in 293A cells (left) and mouse Rai1 in neuro2a cells (right). Asterisks indicate human RAI1 and mouse Rai1 proteins.

(**G**) Representative images showing that rAAV8-treated cultured primary hippocampal neurons express FLAG-sadCas9 in NeuN^+^ neurons (top row) and GFAP^+^ astrocytes (bottom row).

(**H**) Quantification showing that the primary hippocampal neurons contain 79.78±1.56% of NeuN^+^ neurons and 10.93±1.04% of GFAP^+^ astrocytes (n=3 biological replicates, mean±SD).

(**I**) Quantification showing that 80.37±2.86% of NeuN^+^ neurons and 44.59±4.51% of GFAP^+^ astrocytes express FLAG after rAAV8 treatment (n=3 biological replicates, mean±SD, 4 images per replicate).

(**J**) Representative images showing that most WT and SMS primary NeuN^+^ neurons are targeted by control rAAV8 or rAAV8-sg2 with similar efficiencies.

(**K**) Quantification showing that similar percentage of WT and SMS primary NeuN^+^ neurons are targeted by control rAAV8 or rAAV8-sg2 (n=3 biological replicates, mean±SD). One-way ANOVA with Tukey’s post-hoc test. n.s. indicates not significantly different.


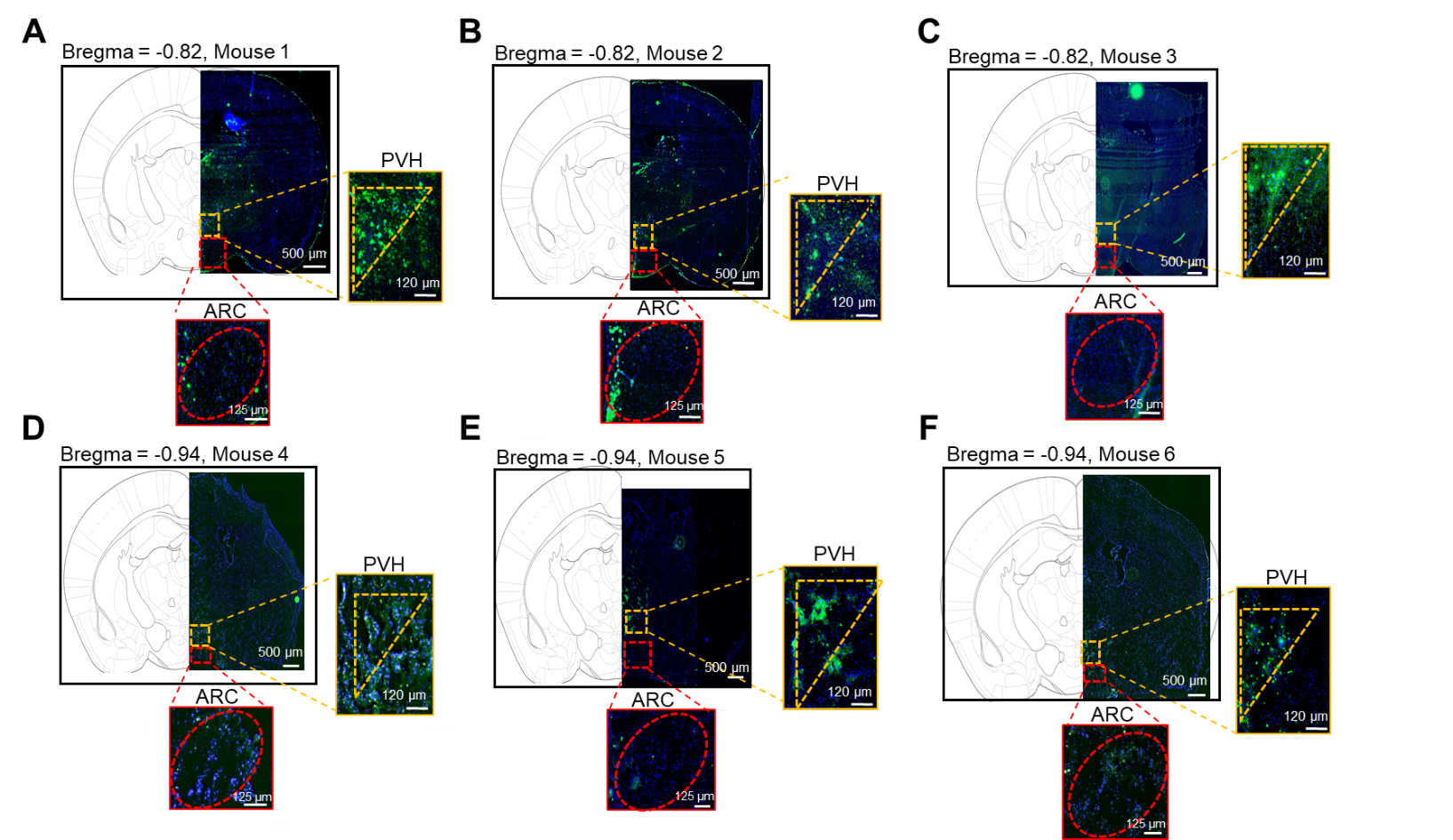


**Figure S2. Distribution of rAAV8 particles in PVH but not hypothalamic arcuate nucleus.**

(**A-F**) Immunostaining data showing FLAG-sadCas9 expression in six mice injected with rAAV8. Shown are DAPI (blue) and FLAG (green) signals detected in the PVH region (orange triangles) between Bregma -0.82 mm and -0.94 mm (anterior-posterior axis). Note the lack of FLAG signals in the nearby arcuate nucleus (ARC, red circles) that also mediates feeding.

**
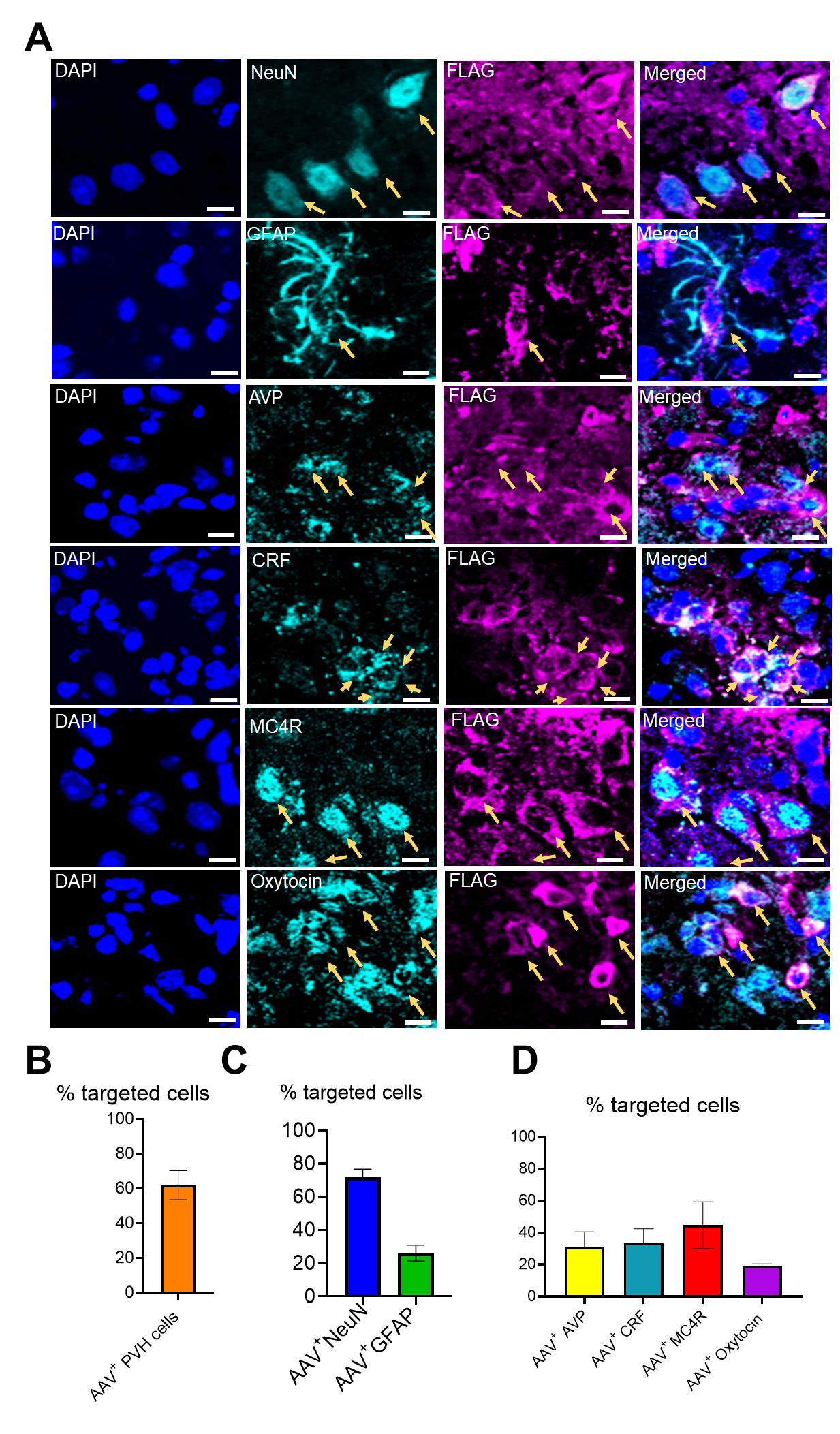
**

**Figure S3. Expression analysis of rAAV8-sg2 in PVH neuronal subtypes.**

(**A**) Representative images of rAAV8-sg2 infected PVH cells co-labeled with FLAG (magenta) and cell type-specific markers (cyan) including NeuN, glial fibrillary acidic protein (GFAP), vasopressin (AVP), corticotropin releasing factor receptor (CRF), melanocotin-4 receptor (MC4R), and oxytocin. Yellow arrows indicate co-localization of FLAG and cell type-specific markers. Scale bars = 10 µm.

(**B**) Quantification showing that 60% of DAPI^+^ cells within PVH are targeted by rAAV8 (mean±SD).

(**C**) Quantification showing that among the targeted PVH cells, 71% are NeuN^+^ neurons and 26% are GFAP^+^ astrocytes (mean±SD).

(**D**) Quantification showing that 30.9% of AVP^+^ PVH neurons, 33.4% of CRF^+^ PVH neurons, 44.7% of MC4R^+^ PVH neurons, and 19% of oxytocin^+^ PVH neurons are targeted with rAAV8-sg2 (mean±SD).

**
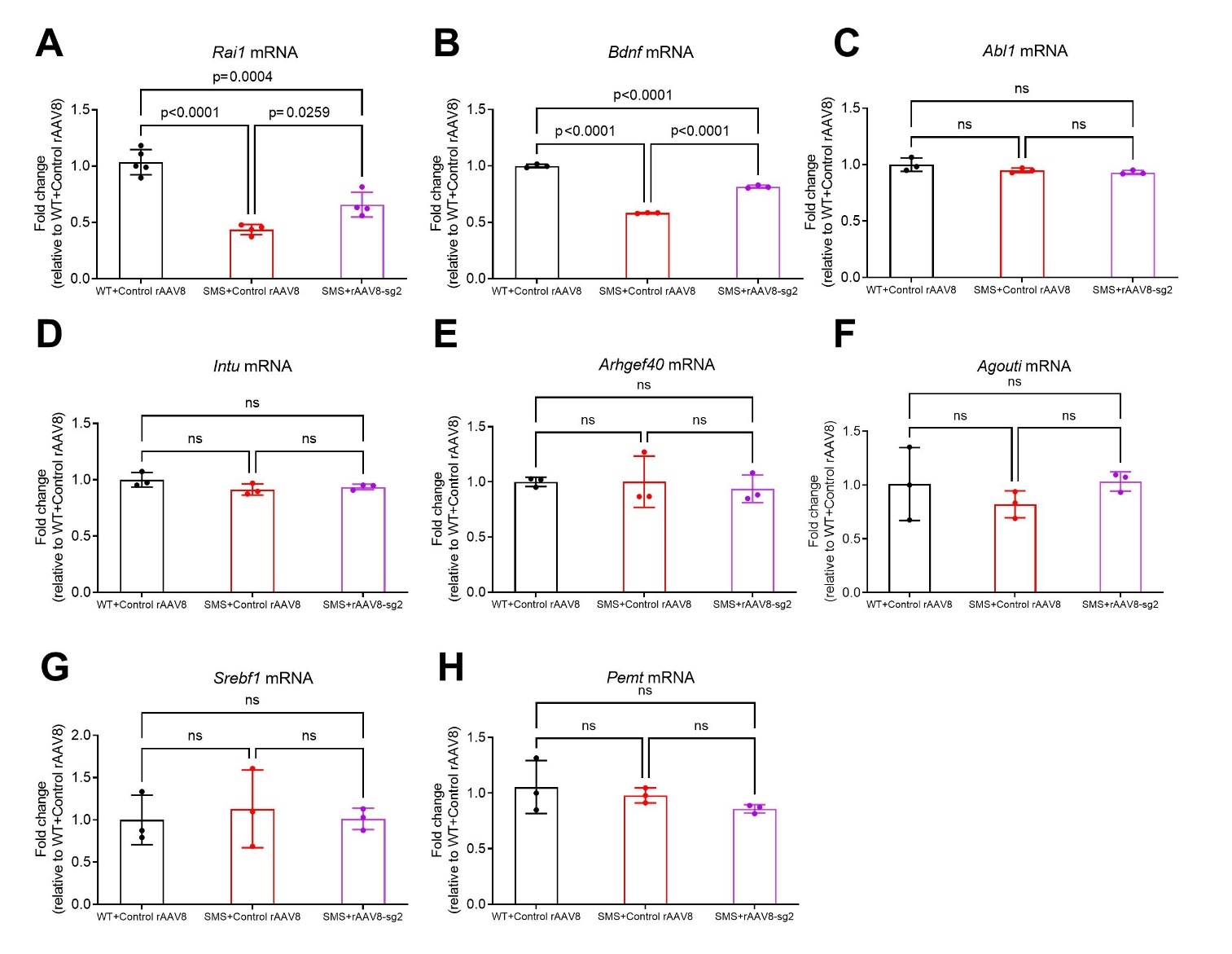
**

**Figure S4. Quantitative RT-PCR showing that rAAV8-sg2 treatment increases expression of hypothalamic *Rai1* and its downstream target *Bdnf* but not predicted sg2 off-target/Rai1 neighboring genes**

(**A**) Quantitative RT-PCR showing that rAAV8-sg2 treatment significantly increased hypothalamic *Rai1* expression in WT and SMS mice (n=2-5 biological replicates per group, mean±SD). Note that the extent of *Rai1* upregulation was not as significant as treatment in primary neurons, likely due to lower infection rate in vivo and the dissected hypothalamic tissues containing non-PVH neurons. Nevertheless, *Rai1* mRNA levels were significantly increased in SMS mice treated with rAAV8-sg2. One-way ANOVA with Tukey’s post-hoc test, F (3, 11) = 68.41, p<0.0001. P-values for the post-hoc tests are indicated in the figure.

(**B**) Quantitative RT-PCR showing that rAAV8-sg2 treatment partially rescued decreased hypothalamic *Bdnf* expression in SMS mice (n=3 biological replicates per group, mean±SD). One-way ANOVA with Tukey’s post-hoc test, F (2, 6) = 859.9, p<0.0001. P-values for the post-hoc tests are indicated in the figure.

(**C-H**) Quantitative RT-PCR showing that rAAV8-sg2 treatment did not significantly alter expression of predicted sg2 off-target genes (**C-F**) and *Rai1* neighboring genes (**G-H**) (n=3 biological replicates per group, mean±SD). One-way ANOVA with Tukey’s post-hoc tests. n.s. indicates not significantly different.

**
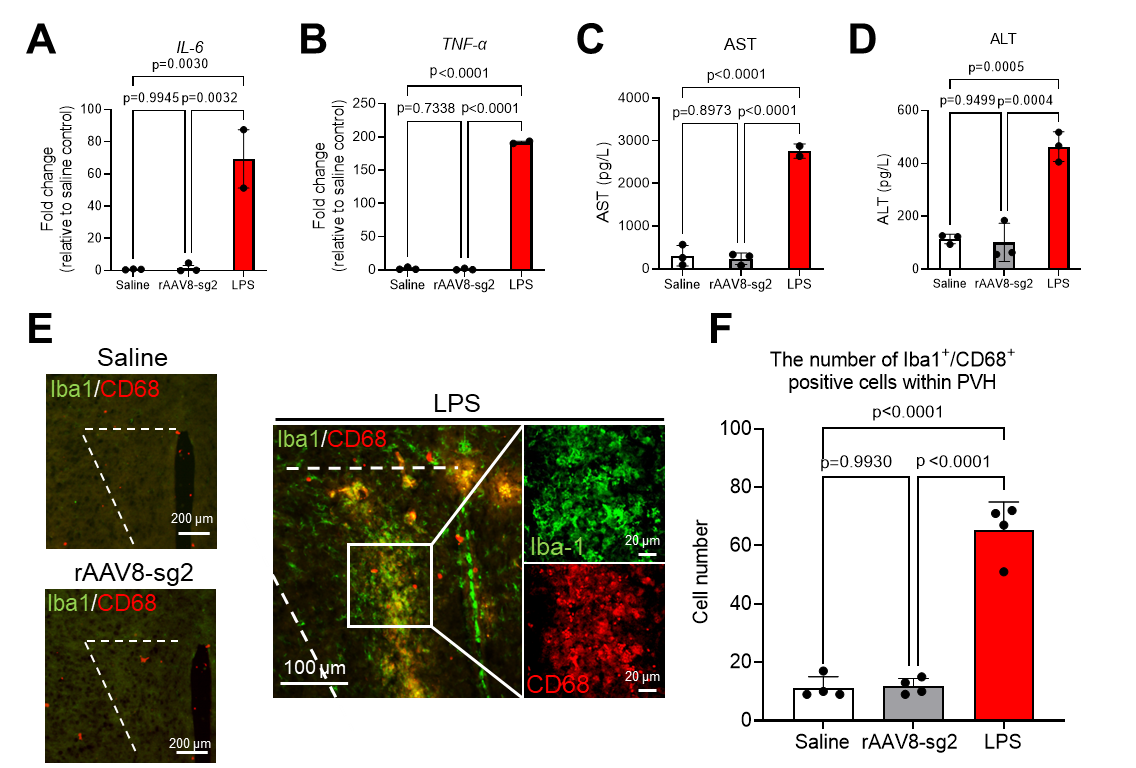
**

**Figure S5. PVH-specific rAAV8-sg2 treatment did not elicit hepatic or local inflammations.**

(**A-B**) Quantitative RT-PCR showing similar mRNA expression levels of the proinflammatory cytokines *interleukin 6* (*IL-6*, **A**, one-way ANOVA with Tukey’s post-hoc test, F (2, 5) = 26.01, p=0.0023) and *tumor necrosis factor alpha* (*TNF-α*, **B**, one-way ANOVA with Tukey’s post-hoc test, F (2, 5) = 8297, p<0.0001) in the livers of 6-month-old saline controls and WT mice with rAAV8-sg2 injected at 4 weeks of age. By contrast, lipopolysaccharide (LPS) injected mice show significantly increased expression of *IL-6* and *TNF-α* in the liver (n=2-3 biological replicates per group). P-values for the post-hoc tests are indicated in the figure.

(**C-D**) Serum levels of aminotransferases AST (**C**, one-way ANOVA with Tukey’s post-hoc test, F (2, 5) = 127.8, p<0.0001) and ALT (**D**, one-way ANOVA with Tukey’s post-hoc test, F (2, 6) = 43.52, p=0.0003) showing that PVH-specific rAAV8-sg2 expression did not significantly elicit liver damage 5 months after injection (AST: 239.07±79.41 ng/L, ALT: 101.79±41.7 ng/L) when compared to saline-treated control mice (AST: 308.72±138.51 ng/L, ALT: 115.27±10.26 ng/L). As a positive control, LPS (1 mg/kg body weight) was I.P. injected for three consecutive days to induce liver damage, as shown by significantly increased AST and ALT levels (AST: 2757.03±118.51 ng/L, ALT: 463.59±32.4 ng/L). P-values for the post-hoc tests are indicated in the figure.

(**E**) Immunostaining data showing that PVH-specific rAAV8-sg2 or saline injections did not induce local inflammation, as evidenced by the lack of activated microglial cells that co-express CD68 and Iba1. As a positive control, LPS (12 mg, concentration 5 mg/µl) was stereotaxically injected the PVH to induce local PVH inflammation (tissues harvested 48 hours after PVH-specific LPS injection).

(**F**) Quantification showing that the average number of Iba1 and CD68 double-positive cells within the PVH were similar between saline treated and rAAV8-sg2 treated mice (four mice per group, each dot indicates one mouse). By contrast, PVH-specific LPS (but not rAAV8-sg2 or saline) injections induced microglial activation, as evidenced by the significantly increased Iba1^+^/CD68^+^ microglial cells. One-way ANOVA with Tukey’s post-hoc test, F (2, 9) = 98.43, p<0.0001. P-values for the post-hoc tests are indicated in the figure.

**
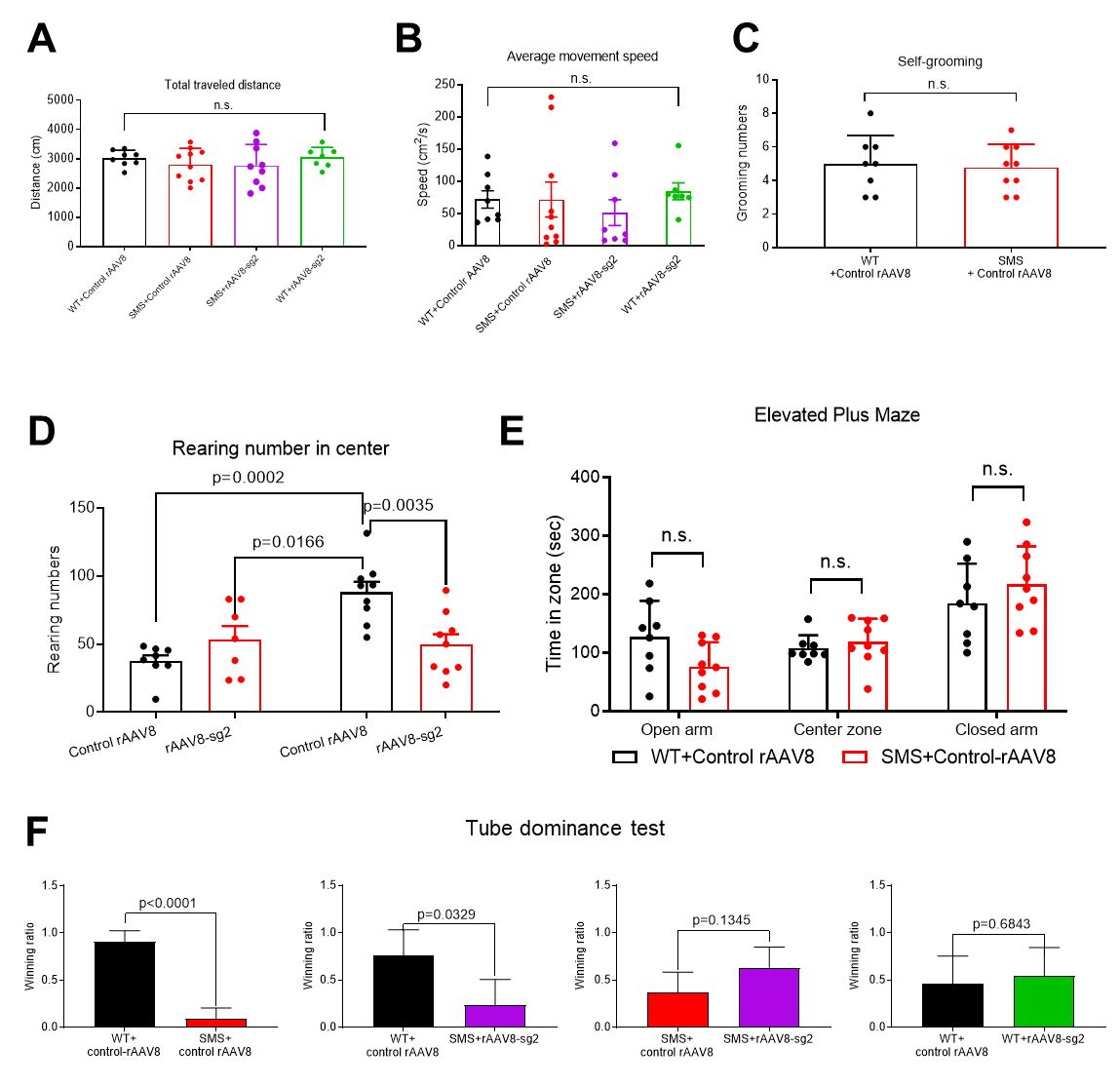
**

**Figure S6. Neurobehavioral features of WT and SMS mice treated with control rAAV8 or rAAV8-sg2 viral particles.** (**A**) All four groups of mice show similar total traveled distance in the open field. Each dot represents one mouse. One-way ANOVA with Tukey's multiple comparisons, F (3, 30) = 0.6151, p=0.6106, n.s. indicates not significantly different.

(**B**) All groups of mice show similar average movement speed in the open field. One-way ANOVA with Tukey's multiple comparisons, F (3, 29) = 0.3909, p=0.7604, n.s. indicates not significantly different.

(**C**) WT and SMS mice show similar self-grooming behavior. Unpaired t-test.

(**D**) SMS mice treated with control rAAV8 show a significantly increased number of rearing episodes in the center of open field, which is normalized by rAAV8-sg2 treatment. Two-way ANOVA with Tukey’s post-hoc test. Genotype: F (1, 29) = 9.740, p =0.0041; Treatment: F (1, 29) = 2.202, =0.1486; genotype and treatment interaction: F (1, 29) = 13.33, p =0.001. P-values for the post-hoc tests are indicated in the figure.

(**E**) WT and SMS mice show similar anxiety levels in the elevated plus maze. Unpaired t-tests.

(**F**) Winning ratios of each group of mice in the tube dominance test showing that rAAV8-sg2 treatment did not improve social dominance deficits in SMS mice (unpaired t-tests). Shown are mean±SD.


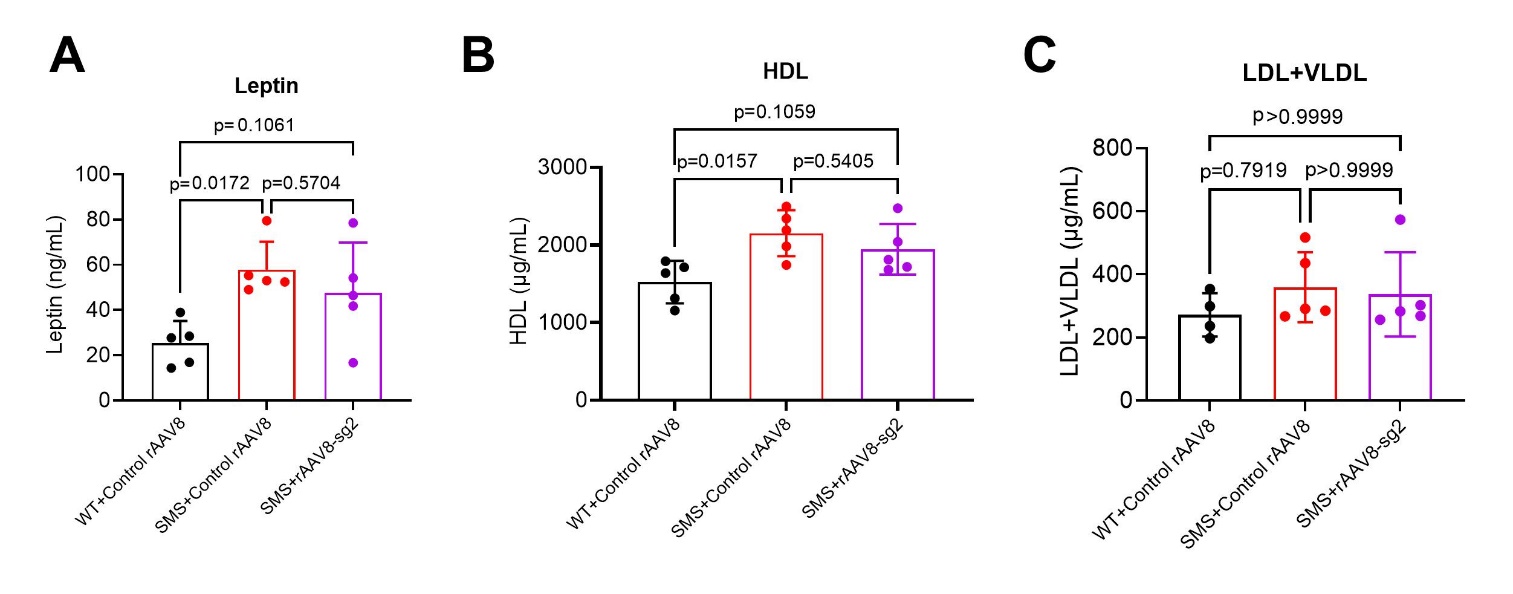
**Figure S7. Lipid profile of WT and SMS mice treated with control rAAV8 or rAAV8-sg2 viral particles.**

SMS mice treated with control rAAV8 show increased leptin (**A**, one-way ANOVA with Tukey's multiple comparisons, F (2, 12) = 5.567, p=0.0195) and HDL (**B**, one-way ANOVA with Tukey's multiple comparisons, F (2, 12) = 5.719, p=0.018) but not LDL+VLDL levels (**C**, one-way ANOVA with Tukey's multiple comparisons, F (2, 11) = 0.7288, p=0.5044) when compared to age-matched WT mice. Treatment with rAAV8-sg2 did not significantly alter leptin or HDL levels. Shown are mean±SD.

**
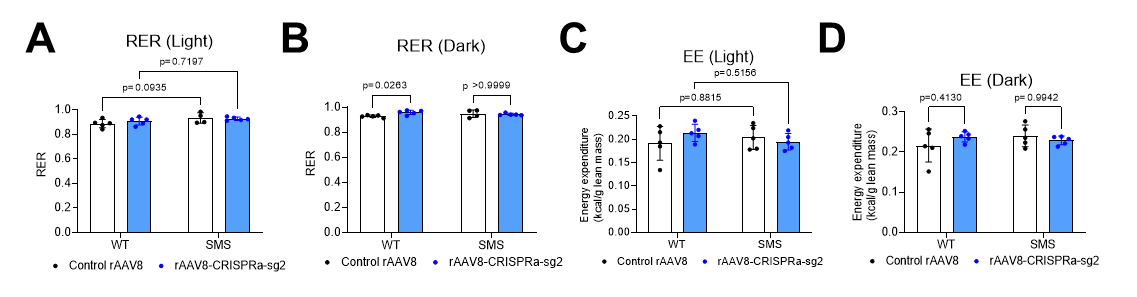
Figure S8. Energy expenditure of WT and SMS mice treated with control rAAV8 or rAAV8-sg2 viral particles.**

(**A**) Mice of all groups show similar levels of respiratory exchange ratio (RER) in the light phase (mean±SD). Two-way ANOVA with Bonferroni’s post-hoc test. Genotype: F (1, 15) = 4.95, p=0.0419; Treatment: F (1, 15) = 0.2691, p=0.6115; Genotype and treatment interaction: F (1, 15) = 0.8628, p=0.3677.

(**B**) WT mice treated with rAAV8-CRISPRa-sg2 viral particles show increased RER in the dark phase (mean±SD). Two-way ANOVA with Bonferroni’s post-hoc test. Genotype: F (1, 15) = 0.1026, p=0.7532; Treatment: F (1, 15) = 2.545, p=0.1315; Genotype and treatment interaction: F (1, 15) = 5.111, p=0.0391.

(**C-D**) No significant differences were detected in energy expenditure (EE) among different groups of mice (mean±SD). Two-way ANOVA with Bonferroni’s post-hoc test. EE (light): Genotype: F (1, 16) = 0.07331, p=0.79; Treatment: F (1, 16) = 0.3632, p=0.5552; Genotype and treatment interaction: F (1, 16) = 1.929, p=0.1839. EE (dark): Genotype: F (1, 16) = 0.4406, p=0.5163; Treatment: F (1, 16) = 0.1933, p=0.6661; Genotype and treatment interaction: F (1, 16) = 2.023, p=0.1741.

**SUPPLEMENTARY TABLES**

**Table S1.** The properties of four different sgRNAs tested in Figure 1.

**Table S2.** Properties of the top 50 predicted off-target sites for sg2. The true and top target DNA site for sg2 is marked in red.

**Table S3.** The primer sequences for qRT-PCR reactions in this study.

**Table S4.** Differentially expressed genes that are upregulated by rAAV8-sg2 treatment (ranked by adjusted p values)

| **log2FoldChange** | **pvalue** | **padj** | **WT-3** | **WT-2** | **WT-1** | **WT-sg2-3** | **WT-sg2-2** | **WT-sg2-1** | **Gene.name** |
| --- | --- | --- | --- | --- | --- | --- | --- | --- | --- |
| 2.815432603 | 6.51E-68 | 2.09E-64 | 1825.130374 | 1603.627648 | 1818.984878 | 10767.92088 | 10845.23108 | 15331.17323 | Ptgs2 |
| 4.239979961 | 3.86E-64 | 1.06E-60 | 456.2825935 | 251.6209385 | 454.2205014 | 6072.903247 | 6064.174008 | 9814.077547 | Chrna1 |
| 3.839908758 | 2.27E-53 | 3.98E-50 | 51.53088839 | 40.11348294 | 36.80027211 | 594.1676046 | 468.8311882 | 783.8720085 | Dsc1 |
| 2.228868825 | 3.67E-52 | 5.89E-49 | 8083.791 | 7781.104021 | 7862.640994 | 33838.39186 | 32170.6686 | 45224.6435 | Bdnf |
| 2.311598146 | 5.68E-52 | 8.42E-49 | 463.7779955 | 399.3114893 | 419.523102 | 1765.188114 | 2203.761847 | 2402.056485 | Lhfpl3 |
| 1.576005092 | 3.28E-50 | 3.96E-47 | 2613.084504 | 2595.52468 | 2615.973629 | 7068.042855 | 7869.727151 | 8394.928397 | Ttc39b |
| 6.827560416 | 5.06E-48 | 5.74E-45 | 4.684626217 | 7.293360535 | 2.102872692 | 339.0036026 | 563.2781245 | 732.0682932 | 1700017M07Rik |
| 2.63370688 | 5.77E-48 | 6.19E-45 | 171.4573195 | 197.8324045 | 177.6927425 | 948.6633073 | 1057.635512 | 1394.610547 | Dtl |
| 3.139353362 | 4.32E-46 | 4.39E-43 | 111.494104 | 87.52032642 | 93.57783478 | 619.6840048 | 984.460408 | 976.0910576 | Sln |
| 5.129606508 | 2.78E-45 | 2.55E-42 | 12.18002816 | 9.116700669 | 13.6686725 | 267.9222021 | 396.5069577 | 556.2083121 | Serpinb2 |
| 2.839867245 | 4.46E-44 | 3.74E-41 | 112.4310292 | 152.2489012 | 129.3266705 | 804.6779062 | 834.7067072 | 1187.395686 | Pcdh15 |
| 2.85004046 | 3.94E-43 | 3.17E-40 | 228.6097594 | 196.9207344 | 177.6927425 | 1086.269608 | 1433.72151 | 1836.305383 | Pde1c |
| 2.511018401 | 1.58E-42 | 1.17E-39 | 2090.280218 | 2427.777388 | 1940.951494 | 12238.75909 | 9320.466127 | 15267.10021 | Pcdh11x |
| 2.181788444 | 3.70E-41 | 2.55E-38 | 25208.9106 | 19019.26093 | 25041.00801 | 89728.42129 | 101462.3866 | 123095.1705 | Scg2 |
| 3.053235502 | 3.88E-41 | 2.58E-38 | 454.408743 | 308.1444826 | 455.2719378 | 3198.663025 | 2476.892176 | 4433.307429 | Grp |
| 3.295863334 | 4.17E-41 | 2.59E-38 | 2255.179061 | 1226.19624 | 2335.240124 | 15253.33952 | 17535.64784 | 24331.38714 | Nptx2 |
| 3.82327625 | 4.10E-41 | 2.59E-38 | 31.85545827 | 39.20181288 | 25.2344723 | 329.8906025 | 435.6471295 | 606.6487718 | Klhl4 |
| 3.119319744 | 5.95E-41 | 3.58E-38 | 159.2772914 | 127.6338094 | 128.2752342 | 850.2429066 | 1163.994674 | 1599.098897 | Penk |
| 1.511262323 | 6.32E-40 | 3.69E-37 | 597.7583053 | 655.4907781 | 605.6273352 | 1664.033813 | 1755.351618 | 1884.019332 | Rps6ka6 |
| 2.209258845 | 1.84E-39 | 1.05E-36 | 1573.097484 | 1276.338094 | 1449.930721 | 5836.876545 | 5792.745426 | 8257.239575 | Pcsk1 |
| 1.550263908 | 2.36E-39 | 1.30E-36 | 2625.264532 | 2881.789081 | 2690.625609 | 7592.040359 | 7417.913428 | 9005.666936 | Armcx4 |
| 4.381001836 | 2.49E-39 | 1.33E-36 | 29.04468254 | 22.79175167 | 37.85170845 | 347.2053027 | 852.5750465 | 661.1789985 | Nrsn2 |
| 3.4347741 | 1.18E-38 | 6.13E-36 | 73.08016898 | 43.76016321 | 68.34336248 | 661.6038051 | 479.0416678 | 861.5775815 | Gm42891 |
| 3.404966099 | 4.03E-38 | 2.04E-35 | 34.666234 | 49.23018361 | 42.05745383 | 311.6646024 | 514.7783464 | 509.8576195 | Scml4 |
| 3.880220803 | 5.53E-38 | 2.74E-35 | 48.72011266 | 42.84849314 | 27.33734499 | 582.3207045 | 382.8929849 | 796.1413095 | Rxfp1 |
| 2.89424779 | 8.30E-38 | 4.00E-35 | 107.746403 | 80.22696588 | 130.3781069 | 611.4823047 | 810.0313815 | 942.0096659 | Gpr83 |
| 3.020894455 | 2.18E-35 | 9.55E-33 | 154.5926652 | 140.3971903 | 119.8637434 | 718.1044055 | 1346.08156 | 1307.362185 | Arg2 |
| 3.128500774 | 4.06E-35 | 1.74E-32 | 322.3022837 | 202.3907548 | 266.0133955 | 1601.154112 | 3196.730988 | 2114.40954 | Asphd1 |
| 1.770948226 | 7.64E-35 | 3.20E-32 | 3121.834911 | 2521.679405 | 3055.474021 | 9040.09607 | 9170.712426 | 11479.97597 | Syt4 |
| 2.605531093 | 9.99E-35 | 4.01E-32 | 2267.359089 | 1406.706913 | 2261.63958 | 9488.455673 | 12129.19889 | 14506.40355 | Gng4 |
| 2.972511114 | 1.22E-34 | 4.81E-32 | 62.77399131 | 48.31851354 | 66.24048979 | 339.0036026 | 505.4187401 | 546.6655225 | Jsrp1 |
| 3.26672956 | 6.03E-34 | 2.28E-31 | 82.44942142 | 53.78853394 | 66.24048979 | 460.2065036 | 601.567423 | 890.2059506 | Arr3 |
| 1.622062081 | 6.84E-34 | 2.54E-31 | 1898.210543 | 1672.914573 | 1876.813877 | 4922.842638 | 5437.93126 | 6412.754658 | Stard4 |
| 2.609597475 | 8.68E-34 | 3.16E-31 | 171.4573195 | 196.9207344 | 165.0755063 | 770.0485059 | 1133.363235 | 1357.802644 | Cd300lb |
| 3.350180942 | 9.48E-34 | 3.38E-31 | 28.1077573 | 23.70342174 | 24.18303596 | 233.2928018 | 216.9726915 | 328.5446157 | AU023762 |
| 2.24831558 | 4.37E-33 | 1.53E-30 | 1176.778106 | 1637.35944 | 1073.516509 | 5411.299442 | 5717.868575 | 7350.674556 | Zdbf2 |
| 2.691254518 | 2.66E-32 | 9.17E-30 | 65.58476704 | 65.64024481 | 74.65198056 | 328.0680025 | 513.0765998 | 488.0455288 | Vash2 |
| 5.09672447 | 3.22E-32 | 1.09E-29 | 14.05387865 | 2.735010201 | 3.154309038 | 186.8165014 | 224.6305512 | 279.4674117 | Crisp1 |
| 2.338943906 | 6.42E-32 | 2.13E-29 | 155.5295904 | 149.513891 | 120.9151798 | 803.7666062 | 556.4711381 | 800.2310765 | Col28a1 |
| 1.318719241 | 7.41E-32 | 2.42E-29 | 10408.30253 | 9036.473703 | 9729.991945 | 24184.07959 | 22293.73133 | 26301.29158 | Nefm |
| 3.267798525 | 1.89E-31 | 5.98E-29 | 81.51249617 | 63.81690468 | 72.54910787 | 413.7302032 | 898.5222047 | 786.5985199 | Pappa2 |
| 1.444397801 | 2.09E-31 | 6.51E-29 | 7496.338872 | 6743.623485 | 7414.729111 | 18297.08154 | 18174.65369 | 22466.45339 | Inpp5f |
| 1.720267215 | 3.59E-31 | 1.08E-28 | 5316.113831 | 3863.657743 | 5111.032077 | 16314.09273 | 14198.52276 | 16574.4624 | Dio2 |
| 1.79201198 | 6.18E-31 | 1.81E-28 | 795.4495316 | 810.4746894 | 770.7028415 | 2406.743319 | 2470.08519 | 3360.425219 | Sox11 |
| 4.67558831 | 6.69E-31 | 1.93E-28 | 8.43232719 | 10.02837074 | 17.87441788 | 173.1470013 | 422.88403 | 323.0915931 | Odaph |
| 1.453053738 | 7.61E-31 | 2.16E-28 | 4782.066442 | 4208.269029 | 4621.06274 | 11691.97909 | 11390.64087 | 14188.76498 | Hdac9 |
| 4.804638012 | 8.23E-31 | 2.30E-28 | 14.05387865 | 2.735010201 | 9.462927113 | 187.7278014 | 212.718325 | 331.2711271 | Hspb3 |
| 4.697993233 | 9.50E-31 | 2.62E-28 | 2.81077573 | 12.76338094 | 6.308618075 | 165.8566013 | 174.4290265 | 234.4799747 | Sele |
| 1.872492503 | 3.97E-30 | 1.05E-27 | 1568.412857 | 1270.868073 | 1596.080373 | 4358.747934 | 5628.526879 | 6254.617 | Tmod1 |
| 2.658041146 | 4.29E-30 | 1.12E-27 | 1058.725525 | 600.7905741 | 1088.236618 | 4800.728437 | 5288.177559 | 7252.520148 | Sorcs3 |
| 4.149227224 | 4.72E-30 | 1.21E-27 | 12.18002816 | 10.02837074 | 19.97729057 | 159.4775012 | 296.1039084 | 286.2836901 | Igfn1 |
| 1.073018891 | 4.88E-30 | 1.24E-27 | 8537.262818 | 9322.738104 | 8478.782693 | 17840.52024 | 18396.73162 | 19179.64398 | Atp6ap2 |
| 1.727358517 | 6.41E-30 | 1.61E-27 | 416.9317333 | 368.314707 | 403.7515568 | 1250.30361 | 1149.529828 | 1541.842159 | Mylk3 |
| 3.514405627 | 1.01E-29 | 2.50E-27 | 40.28778546 | 18.23340134 | 33.64596307 | 245.1397019 | 385.4456048 | 421.2460011 | 1700014D04Rik |
| 4.59895106 | 2.94E-29 | 7.17E-27 | 14.05387865 | 10.9400408 | 9.462927113 | 151.2758012 | 397.357831 | 289.0102014 | Tdrd9 |
| 2.554342708 | 5.99E-29 | 1.44E-26 | 432.8594624 | 260.7376391 | 401.6486841 | 1596.597612 | 2186.744381 | 2650.169017 | Acan |
| 1.342589182 | 7.50E-29 | 1.79E-26 | 1901.021319 | 1790.520011 | 1810.573388 | 4332.320233 | 4347.962562 | 5279.889198 | Rassf8 |
| 1.996253361 | 1.06E-28 | 2.50E-26 | 4306.108419 | 3194.491914 | 4307.734709 | 14035.84271 | 13611.42018 | 19465.92767 | Fosl2 |
| 1.232077817 | 2.75E-28 | 6.17E-26 | 1260.164452 | 1259.928032 | 1239.643452 | 2897.934022 | 2707.47884 | 3232.279186 | Hs6st2 |
| 2.327009252 | 8.67E-28 | 1.88E-25 | 653.0368946 | 844.2064819 | 612.9873896 | 3473.875627 | 2568.786492 | 4553.273928 | Zfp804a |
| 1.433682137 | 9.74E-28 | 2.09E-25 | 4351.08083 | 4331.344488 | 3937.629115 | 11252.73249 | 9842.05146 | 13004.09581 | Pcdh17 |
| 1.418007129 | 1.39E-27 | 2.91E-25 | 3199.599706 | 3801.664179 | 2955.587568 | 8332.927264 | 8704.433858 | 9575.507805 | Nap1l5 |
| 2.520936372 | 1.96E-27 | 4.02E-25 | 580.8936509 | 344.6112853 | 526.7696093 | 2606.31802 | 2131.437616 | 3598.994961 | Cyp26b1 |
| 2.405843353 | 2.35E-27 | 4.73E-25 | 124.6110574 | 83.87364615 | 144.0467794 | 539.4896042 | 585.4008303 | 741.6110828 | Alkal2 |
| 2.622931369 | 2.39E-27 | 4.75E-25 | 630.5506888 | 319.0845234 | 566.7241904 | 2434.082319 | 3243.529019 | 3661.704722 | Fndc9 |
| 1.907409464 | 3.49E-27 | 6.88E-25 | 2660.867691 | 3802.575849 | 2543.424521 | 9760.934375 | 11576.98212 | 12456.06703 | Gabra1 |
| 1.736911894 | 3.77E-27 | 7.33E-25 | 742.0447927 | 602.6139142 | 720.2338969 | 2243.620617 | 1929.780644 | 2712.878777 | Gm43682 |
| 2.003390279 | 6.25E-27 | 1.17E-24 | 5436.040262 | 3688.617091 | 5451.697453 | 16243.92263 | 19176.13156 | 23022.6617 | Lgi2 |
| 1.643003884 | 8.06E-27 | 1.49E-24 | 4756.769461 | 5846.540139 | 4646.297212 | 16001.51682 | 13362.1143 | 18270.35245 | Gabrg2 |
| 1.053201998 | 1.05E-26 | 1.91E-24 | 1747.365579 | 1575.365876 | 1725.407044 | 3570.473428 | 3436.677258 | 3466.759161 | Nwd2 |
| 2.487415578 | 1.07E-26 | 1.93E-24 | 361.6531439 | 249.7975983 | 363.7969757 | 1231.16631 | 2126.332376 | 2110.319773 | Hcn4 |
| 1.095905916 | 1.39E-26 | 2.46E-24 | 2181.161967 | 2002.027467 | 2067.123856 | 4237.545033 | 4360.725662 | 4765.941812 | Hecw2 |
| 3.126913693 | 1.46E-26 | 2.56E-24 | 163.0249923 | 172.3056426 | 205.0300874 | 801.9440062 | 1848.947681 | 2069.422103 | Stac2 |
| 2.454048906 | 3.28E-26 | 5.60E-24 | 232.3574604 | 216.9774759 | 235.5217415 | 800.1214062 | 1400.537452 | 1554.11146 | Cpxm2 |
| 4.325133441 | 5.68E-26 | 9.29E-24 | 25.29698157 | 11.85171087 | 17.87441788 | 174.9696014 | 531.7958124 | 395.3441434 | Aox4 |
| 4.258237276 | 6.15E-26 | 9.96E-24 | 10.30617768 | 7.293360535 | 6.308618075 | 128.493301 | 129.3327416 | 204.4883501 | Gpr151 |
| 1.869890031 | 8.99E-26 | 1.42E-23 | 400.0670789 | 295.3811017 | 397.4429387 | 1133.657209 | 1295.880036 | 1566.380761 | Bves |
| 3.878160841 | 1.37E-25 | 2.13E-23 | 15.92772914 | 10.02837074 | 13.6686725 | 123.936801 | 217.8235648 | 241.2962531 | Hsf2bp |
| 2.595314678 | 2.03E-25 | 3.06E-23 | 76.82786996 | 139.4855202 | 78.85772594 | 527.6427041 | 542.8571653 | 719.7989922 | Olfm3 |
| 1.228368079 | 2.17E-25 | 3.24E-23 | 21652.34237 | 17840.47154 | 21125.45906 | 43859.95804 | 49422.97562 | 48745.93289 | Prkacb |
| 2.371304846 | 2.52E-25 | 3.70E-23 | 79.63864569 | 61.08189448 | 91.47496209 | 313.4872024 | 456.0680887 | 429.4255351 | Gm13383 |
| 4.827224357 | 2.79E-25 | 4.08E-23 | 6.558476704 | 5.470020401 | 9.462927113 | 107.5334008 | 276.5338225 | 220.8474181 | Krt75 |
| 2.969005856 | 8.78E-25 | 1.21E-22 | 80.57557093 | 36.46680267 | 63.08618075 | 364.5200028 | 439.901496 | 606.6487718 | Insc |
| 2.245302986 | 9.44E-25 | 1.28E-22 | 101.1879263 | 86.60865635 | 76.75485325 | 335.3584026 | 410.1209305 | 513.9473865 | Gm3294 |
| 1.352674888 | 1.54E-24 | 2.04E-22 | 13221.88903 | 15771.89216 | 12545.73848 | 31926.48445 | 35353.78561 | 38811.88884 | Psd3 |
| 2.010645248 | 1.58E-24 | 2.08E-22 | 444.1025654 | 312.7028329 | 394.2886297 | 1314.09461 | 1378.414746 | 1949.455604 | Fstl4 |
| 1.949875266 | 1.60E-24 | 2.10E-22 | 197.6912264 | 227.9175167 | 192.4128513 | 619.6840048 | 815.9874946 | 957.0054782 | Zfp57 |
| 1.779615965 | 3.31E-24 | 4.23E-22 | 1034.365469 | 711.1026522 | 996.7616559 | 2783.110221 | 2991.670522 | 3641.255887 | Celf6 |
| 3.960032477 | 3.37E-24 | 4.27E-22 | 14.05387865 | 5.470020401 | 16.82298153 | 130.315901 | 192.2973658 | 238.5697417 | Stac |
| 2.678879784 | 4.29E-24 | 5.37E-22 | 319.491508 | 198.7440746 | 369.0541574 | 1295.86861 | 1885.535233 | 2498.847638 | Epha10 |
| 4.122609448 | 4.64E-24 | 5.77E-22 | 17.80157962 | 8.205030602 | 5.257181729 | 128.493301 | 189.7447459 | 233.1167191 | Gm29593 |
| 1.516993578 | 8.85E-24 | 1.09E-21 | 1273.281406 | 1270.868073 | 1262.775051 | 3491.190327 | 3031.661567 | 4378.777202 | Htr2c |
| 1.432143078 | 9.65E-24 | 1.18E-21 | 4220.848221 | 5390.705105 | 4083.778767 | 11721.14069 | 11643.35024 | 13598.47528 | Slk |
| 1.90057314 | 1.08E-23 | 1.31E-21 | 1203.948938 | 877.0266043 | 1286.958087 | 3604.191528 | 3810.210637 | 5161.285955 | Sstr2 |
| 1.301669389 | 1.97E-23 | 2.34E-21 | 1528.125072 | 1687.501294 | 1392.101722 | 3616.949728 | 3501.343629 | 4247.904658 | Nabp1 |
| 3.471761426 | 2.22E-23 | 2.62E-21 | 55.27858936 | 47.40684348 | 88.32065305 | 351.7618027 | 981.9077881 | 779.7822415 | Hs3st2 |
| 1.950147665 | 2.36E-23 | 2.76E-21 | 430.0486867 | 294.4694316 | 446.860447 | 1318.65111 | 1393.730465 | 1814.493293 | Sult2b1 |
| 1.399649769 | 2.57E-23 | 2.99E-21 | 4944.154509 | 3897.389536 | 5042.688715 | 10848.11528 | 13050.69467 | 12731.44467 | Zcchc12 |
| 1.347790395 | 2.70E-23 | 3.12E-21 | 1701.456242 | 2123.279586 | 1720.149862 | 4246.658033 | 4776.802706 | 5094.486428 | Abca5 |
| 2.124173122 | 2.89E-23 | 3.31E-21 | 390.6978265 | 492.3018361 | 461.5805558 | 1884.568415 | 1408.195311 | 2573.826699 | Tll1 |
| 2.993343989 | 3.33E-23 | 3.77E-21 | 52.46781363 | 62.90523461 | 72.54910787 | 292.5273023 | 689.2073729 | 512.5841308 | Ptgs1 |
| 1.58297798 | 4.67E-23 | 5.17E-21 | 3459.127999 | 4460.801637 | 3318.333108 | 9896.718076 | 10713.34572 | 13065.44231 | Gad2 |
| 2.140567655 | 5.27E-23 | 5.80E-21 | 325.1130594 | 508.7118973 | 308.0708493 | 1563.790812 | 1438.82675 | 2039.430478 | Cdh12 |
| 3.639268969 | 6.84E-23 | 7.45E-21 | 34.666234 | 18.23340134 | 21.02872692 | 166.7679013 | 401.6121975 | 354.4464734 | Vgll2 |
| 1.571473463 | 7.48E-23 | 8.05E-21 | 9377.684761 | 6608.696315 | 9528.116166 | 23323.81238 | 25066.72741 | 27439.61007 | Gap43 |
| 1.293278419 | 7.55E-23 | 8.09E-21 | 1392.270912 | 1194.287788 | 1414.181885 | 3075.637524 | 3047.82816 | 3684.880068 | Mir9-3hg |
| 1.225179111 | 1.05E-22 | 1.10E-20 | 1019.374665 | 1057.537278 | 1063.002146 | 2136.998516 | 2513.479728 | 2693.793198 | Tmeff1 |
| 1.841933608 | 1.10E-22 | 1.15E-20 | 3628.711468 | 4069.695178 | 3684.232956 | 12742.708 | 10322.79487 | 17746.86227 | Homer1 |
| 1.495726819 | 1.13E-22 | 1.17E-20 | 667.0907733 | 640.904057 | 744.4169329 | 1744.228213 | 1740.886772 | 2306.628589 | Dok5 |
| 1.880285887 | 1.64E-22 | 1.69E-20 | 931.3036919 | 590.7622033 | 936.8297842 | 2971.749323 | 2648.768583 | 3431.314514 | Adcyap1 |
| 2.649076478 | 1.67E-22 | 1.71E-20 | 78.70172044 | 66.55191488 | 55.72612633 | 281.5917022 | 430.5418897 | 553.4818008 | Pappa |
| 2.018340832 | 1.73E-22 | 1.77E-20 | 545.2904916 | 388.3714485 | 563.5698814 | 1828.979114 | 1652.395948 | 2586.096 | A830036E02Rik |
| 2.507270122 | 3.19E-22 | 3.21E-20 | 49.6570379 | 53.78853394 | 48.36607191 | 212.3329016 | 282.4899356 | 372.1687971 | Ankrd34c |
| 2.869990248 | 4.03E-22 | 4.03E-20 | 112.4310292 | 61.99356455 | 133.5324159 | 521.263604 | 716.4353185 | 1011.535705 | Aloxe3 |
| 1.152840466 | 4.95E-22 | 4.89E-20 | 4306.108419 | 4171.802226 | 4297.220346 | 8697.447267 | 8898.43297 | 10816.07046 | Rapgef4 |
| 1.537007817 | 5.18E-22 | 5.09E-20 | 1745.491728 | 1180.612737 | 1640.2407 | 4651.275236 | 4415.181553 | 4181.105131 | Tceal5 |
| 1.674066242 | 5.96E-22 | 5.77E-20 | 682.0815772 | 440.3366423 | 649.7876617 | 1800.728814 | 1864.2634 | 1988.990018 | Tceal6 |
| 1.838730299 | 6.64E-22 | 6.37E-20 | 336.3561624 | 299.0277819 | 335.4081943 | 855.7107066 | 1377.563873 | 1239.199401 | Pafah1b3 |
| 1.505476836 | 6.71E-22 | 6.40E-20 | 272.6452458 | 247.9742582 | 281.7849407 | 656.1360051 | 863.6363994 | 756.6068952 | Slc1a6 |
| 1.104205242 | 6.80E-22 | 6.46E-20 | 7235.873655 | 7364.4708 | 7193.927478 | 13759.71881 | 15884.95364 | 17213.82931 | Akap6 |
| 1.366596131 | 8.12E-22 | 7.56E-20 | 11830.55505 | 14177.38121 | 11627.83455 | 31625.75544 | 28214.10775 | 37215.51645 | Tcaf1 |
| 2.500357882 | 9.50E-22 | 8.80E-20 | 42.16163595 | 43.76016321 | 57.82899902 | 219.6233017 | 244.2006371 | 350.3567064 | Ptger4 |
| 1.52824176 | 9.76E-22 | 8.96E-20 | 5128.728782 | 3994.938233 | 5375.994036 | 11622.72029 | 15075.77313 | 15122.59511 | Kcnip3 |
| 3.724897417 | 1.06E-21 | 9.71E-20 | 13.11695341 | 7.293360535 | 9.462927113 | 98.42040076 | 131.8853615 | 166.3171914 | Gm28578 |
| 1.507411033 | 1.71E-21 | 1.51E-19 | 1179.588881 | 1064.830638 | 1163.940035 | 2838.699522 | 2873.399134 | 3983.433059 | Trpc6 |
| 1.700131595 | 2.08E-21 | 1.79E-19 | 1109.319488 | 766.7145262 | 1121.882581 | 2764.884221 | 3304.791897 | 3671.247511 | Smyd4 |
| 1.90776747 | 2.62E-21 | 2.24E-19 | 775.7741015 | 534.2386592 | 763.3427871 | 1960.206315 | 2882.75874 | 2936.452707 | Rgs6 |
| 1.245392881 | 2.67E-21 | 2.27E-19 | 6678.403135 | 7970.731395 | 6335.95542 | 15833.83762 | 15553.96392 | 18371.23337 | Pak3 |
| 1.765690374 | 3.06E-21 | 2.58E-19 | 118.9895059 | 108.488738 | 141.9439067 | 391.859003 | 381.1912383 | 483.9557618 | Gm13716 |
| 1.448090663 | 3.28E-21 | 2.75E-19 | 6596.890639 | 5442.670299 | 7005.720373 | 14338.39431 | 18733.67744 | 18891.99703 | Csdc2 |
| 1.732702404 | 3.48E-21 | 2.90E-19 | 1212.381265 | 804.092999 | 1170.248653 | 3258.808825 | 3189.073128 | 4144.297228 | Bex1 |
| 1.259219671 | 7.53E-21 | 6.07E-19 | 5594.380628 | 5849.275149 | 5445.388835 | 12599.6339 | 11937.7524 | 15896.92433 | R3hdm1 |
| 1.321068698 | 8.80E-21 | 7.01E-19 | 2376.979342 | 2171.598099 | 2334.188688 | 5614.519343 | 4862.740909 | 6724.940205 | Grm7 |
| 1.503151652 | 8.79E-21 | 7.01E-19 | 37675.63789 | 45100.31821 | 35334.56984 | 99677.99477 | 99518.99203 | 135608.4942 | Meg3 |
| 2.114204536 | 1.11E-20 | 8.79E-19 | 1508.449642 | 2538.089466 | 1463.599393 | 8191.675763 | 6293.058926 | 9377.835733 | Spock3 |
| 2.414295445 | 1.50E-20 | 1.17E-18 | 56.2155146 | 53.78853394 | 73.60054421 | 246.0510019 | 305.4635147 | 428.0622794 | Olfr443-ps1 |
| 1.129337994 | 1.58E-20 | 1.23E-18 | 3299.850707 | 2828.912217 | 3338.310398 | 6261.542348 | 7431.527401 | 7015.313662 | Hmox2 |
| 1.531998222 | 1.69E-20 | 1.31E-18 | 2452.870287 | 2455.12749 | 2402.53205 | 6178.614048 | 6025.033836 | 8944.320431 | Mest |
| 1.129681981 | 1.72E-20 | 1.33E-18 | 511.5611829 | 485.9201456 | 474.197792 | 1037.970708 | 1163.143801 | 1016.988728 | Emb |
| 1.201218954 | 2.23E-20 | 1.71E-18 | 11221.55364 | 9581.652403 | 11075.83047 | 24107.53039 | 21640.26064 | 27556.85005 | Vsnl1 |
| 2.349074102 | 2.82E-20 | 2.14E-18 | 88.07097288 | 66.55191488 | 115.657998 | 368.1652028 | 415.2261703 | 593.0162151 | Dusp26 |
| 1.930276643 | 4.58E-20 | 3.45E-18 | 553.7228188 | 342.7879451 | 539.3868454 | 1448.055711 | 1947.648983 | 2076.238381 | Sema4a |
| 1.239990525 | 4.74E-20 | 3.56E-18 | 10327.72696 | 7758.312269 | 9774.152271 | 22886.38838 | 20426.91531 | 22490.99199 | Chgb |
| 1.22396409 | 4.84E-20 | 3.61E-18 | 3459.127999 | 2703.101748 | 3271.018472 | 6936.815654 | 7009.494244 | 8090.922384 | Arxes1 |
| 1.493147008 | 5.01E-20 | 3.73E-18 | 4413.854822 | 5652.354415 | 3950.246351 | 13597.5074 | 11341.29021 | 14524.12588 | Acsl4 |
| 1.353650055 | 5.44E-20 | 4.03E-18 | 327.9238352 | 297.2044418 | 358.5397939 | 742.7095057 | 849.1715533 | 922.9240866 | Abcg2 |
| 1.210360563 | 6.28E-20 | 4.64E-18 | 4637.779955 | 3462.522914 | 4371.872326 | 9765.490875 | 9423.421796 | 9669.572446 | Rai1 |
| 1.915753519 | 7.24E-20 | 5.30E-18 | 519.0565848 | 333.6712445 | 544.6440272 | 1429.829711 | 1767.263844 | 2074.875125 | Pthlh |
| 1.735770755 | 8.41E-20 | 6.14E-18 | 287.6360497 | 219.7124861 | 314.3794674 | 914.0339071 | 752.1719971 | 1071.518954 | Brinp3 |
| 2.228318693 | 9.87E-20 | 7.15E-18 | 480.6426498 | 812.2980296 | 415.3173566 | 2635.47962 | 2149.305956 | 3226.826164 | B3galt2 |
| 1.126113891 | 1.19E-19 | 8.49E-18 | 641.7937917 | 655.4907781 | 603.5244625 | 1282.19911 | 1337.572827 | 1535.025881 | Gpr137c |
| 2.634506906 | 1.58E-19 | 1.11E-17 | 173.33117 | 104.8420577 | 132.4809796 | 487.5455038 | 1078.907344 | 984.2705916 | Gm9899 |
| 1.631766743 | 1.60E-19 | 1.12E-17 | 31456.32812 | 20940.14977 | 31886.91006 | 79364.20631 | 81254.99665 | 100568.7338 | Ptprn |
| 3.441337084 | 1.66E-19 | 1.16E-17 | 42.16163595 | 26.43843194 | 63.08618075 | 228.7363018 | 581.1464638 | 616.1915615 | Gipr |
| 5.517003952 | 3.04E-19 | 2.08E-17 | 1.873850487 | 6.381690468 | 2.102872692 | 67.43620052 | 214.4200716 | 200.398583 | Lmod3 |
| 1.586564269 | 3.22E-19 | 2.19E-17 | 2565.301316 | 2869.93737 | 2593.893465 | 8355.709764 | 5984.191918 | 9779.996155 | Rnf128 |
| 1.772914015 | 3.27E-19 | 2.21E-17 | 4052.201678 | 2537.177796 | 4350.843599 | 11085.05329 | 12313.8384 | 13987.00314 | Nell1 |
| 1.309674785 | 3.32E-19 | 2.24E-17 | 530.2996877 | 439.4249722 | 539.3868454 | 1090.826108 | 1324.809728 | 1325.084508 | Ing5 |
| 1.597610911 | 3.49E-19 | 2.34E-17 | 674.5861752 | 469.5100844 | 657.1477162 | 1526.427512 | 2021.674961 | 1901.741655 | Cdh22 |
| 1.371445153 | 3.92E-19 | 2.62E-17 | 3376.678577 | 2630.168143 | 3262.606981 | 7473.571358 | 7122.660393 | 9390.105034 | Rgs7 |
| 2.341109686 | 4.05E-19 | 2.69E-17 | 80.57557093 | 133.1038298 | 96.73214382 | 361.7861028 | 645.8128346 | 567.1143575 | Nlrc3 |
| 1.984248182 | 5.03E-19 | 3.33E-17 | 112.4310292 | 93.90201689 | 88.32065305 | 324.4228025 | 348.858053 | 497.5883185 | Kctd8 |
| 6.198467812 | 5.75E-19 | 3.78E-17 | 2.81077573 | 1.823340134 | 1.051436346 | 75.63790058 | 172.7272799 | 175.859981 | Lce1g |
| 2.506248813 | 6.26E-19 | 4.09E-17 | 128.3587583 | 162.2772719 | 108.2979436 | 616.0388048 | 532.6466857 | 1124.685925 | Inhba |
| 2.607109316 | 7.57E-19 | 4.90E-17 | 110.5571787 | 99.37203729 | 94.62927113 | 339.9149026 | 843.2154402 | 673.4482995 | Clec18a |
| 1.314285424 | 8.56E-19 | 5.48E-17 | 1202.075087 | 1551.662454 | 1133.448381 | 2993.620523 | 3215.4502 | 3462.669394 | Armcx1 |
| 1.84066488 | 9.46E-19 | 6.01E-17 | 401.9409294 | 491.390166 | 360.6426666 | 1163.730109 | 1387.774352 | 1946.729092 | Fam46a |
| 3.610956267 | 9.85E-19 | 6.21E-17 | 8.43232719 | 7.293360535 | 13.6686725 | 92.04130071 | 104.6574159 | 160.8641687 | Gm19410 |
| 1.145552542 | 1.06E-18 | 6.66E-17 | 6186.517382 | 5131.790806 | 6131.976769 | 11565.30839 | 12786.92395 | 14255.56451 | Frmpd3 |
| 1.682760745 | 1.23E-18 | 7.66E-17 | 155.5295904 | 165.0122821 | 215.5444509 | 482.9890037 | 654.3215676 | 580.7469141 | Neurog2 |
| 1.849089974 | 1.73E-18 | 1.05E-16 | 288.572975 | 222.4474963 | 234.4703051 | 654.3134051 | 1094.223064 | 937.9198989 | Xdh |
| 2.482696702 | 1.85E-18 | 1.13E-16 | 65.58476704 | 73.84527542 | 46.26319922 | 336.2697026 | 241.6480172 | 466.2334381 | Gm42528 |
| 1.439347845 | 1.92E-18 | 1.15E-16 | 1770.78871 | 2478.830912 | 1755.898698 | 5420.412442 | 4883.161868 | 5988.782145 | Pcdh7 |
| 3.090550696 | 2.58E-18 | 1.52E-16 | 12.18002816 | 14.58672107 | 17.87441788 | 92.04130071 | 132.7362348 | 155.411146 | Gm35037 |
| 1.135664526 | 2.85E-18 | 1.66E-16 | 9868.633588 | 8384.629605 | 9725.786199 | 19369.68165 | 18737.08094 | 23373.01841 | 11-Sep |
| 2.377849975 | 3.01E-18 | 1.75E-16 | 95.56637482 | 83.87364615 | 148.2525248 | 382.746003 | 676.4442734 | 640.7301635 | Gm39822 |
| 1.40414533 | 3.47E-18 | 2.01E-16 | 523.741211 | 434.8666219 | 579.3414266 | 1191.069109 | 1322.257108 | 1558.201227 | Trpv2 |
| 1.269912323 | 5.96E-18 | 3.35E-16 | 2821.081908 | 2138.777977 | 2710.6029 | 5585.357743 | 5974.832312 | 6938.971345 | Pcbp3 |
| 1.570370323 | 6.72E-18 | 3.75E-16 | 10098.18027 | 6797.412019 | 10283.04746 | 23629.09788 | 25748.27693 | 31338.52127 | Iqsec3 |
| 1.463775482 | 6.81E-18 | 3.79E-16 | 8625.33379 | 6287.788451 | 8671.195544 | 21475.69597 | 18320.15302 | 25258.401 | Nr4a3 |
| 1.111504296 | 6.94E-18 | 3.85E-16 | 420.6794343 | 419.3682308 | 439.5003926 | 826.5491064 | 972.5481818 | 966.5482679 | Fosl1 |
| 3.627918597 | 1.04E-17 | 5.67E-16 | 9.369252434 | 5.470020401 | 9.462927113 | 82.01700063 | 87.63994989 | 130.872544 | 1700048F04Rik |
| 2.815391853 | 1.48E-17 | 7.88E-16 | 34.666234 | 12.76338094 | 26.28590865 | 194.1069015 | 143.7975877 | 179.949748 | Pgr15l |
| 1.67596779 | 1.52E-17 | 8.07E-16 | 3181.798126 | 3030.391302 | 3181.646383 | 6994.227554 | 10230.90056 | 12795.51769 | Igsf9b |
| 1.744025228 | 1.59E-17 | 8.42E-16 | 538.7320149 | 374.6963975 | 584.5986083 | 1302.24771 | 1831.930215 | 1882.656076 | Cckbr |
| 1.452897021 | 1.67E-17 | 8.76E-16 | 1843.868879 | 1501.5206 | 1646.549318 | 3523.085827 | 4804.881524 | 5341.235703 | Vegfa |
| 2.601205205 | 1.70E-17 | 8.88E-16 | 29.04468254 | 40.11348294 | 25.2344723 | 133.961101 | 221.227058 | 220.8474181 | Gm14005 |
| 1.604347241 | 1.80E-17 | 9.31E-16 | 8667.495426 | 6105.454438 | 8558.691855 | 18219.62104 | 25960.14438 | 26762.072 | 6430548M08Rik |
| 1.209550459 | 1.93E-17 | 9.96E-16 | 4980.694594 | 6411.77558 | 4734.617865 | 11656.43839 | 13273.62348 | 12368.81867 | Rabgap1l |
| 2.050464188 | 2.15E-17 | 1.10E-15 | 39.35086022 | 58.34688428 | 46.26319922 | 174.9696014 | 222.9288046 | 199.0353274 | Exph5 |
| 3.011840544 | 2.15E-17 | 1.10E-15 | 20.61235535 | 18.23340134 | 14.72010884 | 154.9210012 | 95.29780959 | 185.4027707 | Clca1 |
| 2.625507109 | 2.64E-17 | 1.34E-15 | 31.85545827 | 28.26177207 | 24.18303596 | 156.7436012 | 131.8853615 | 235.8432304 | Krt20 |
| 1.428745516 | 3.16E-17 | 1.58E-15 | 163.9619176 | 161.3656018 | 177.6927425 | 383.657303 | 496.0591338 | 474.4129721 | Trim36 |
| 1.193390556 | 3.62E-17 | 1.80E-15 | 1306.073789 | 1136.852573 | 1408.924703 | 2618.16492 | 2846.171188 | 3346.792662 | Cnih3 |
| 1.306590268 | 4.17E-17 | 2.06E-15 | 274.5190963 | 287.1760711 | 244.9846686 | 631.5309049 | 604.9709162 | 764.7864292 | Slco5a1 |
| 1.041627074 | 4.49E-17 | 2.21E-15 | 2567.175167 | 2342.992072 | 2471.926849 | 4440.764934 | 5105.239799 | 5654.784507 | Dennd5b |
| 1.545875954 | 6.86E-17 | 3.31E-15 | 683.0185024 | 519.6519381 | 713.9252788 | 1600.242812 | 1695.790487 | 2302.538822 | Smad7 |
| 1.403714827 | 7.22E-17 | 3.46E-15 | 625.8660626 | 514.1819177 | 614.038826 | 1336.87711 | 1420.107538 | 1888.109099 | Baalc |
| 1.308088369 | 7.71E-17 | 3.64E-15 | 2715.209355 | 2134.219627 | 2724.271572 | 5224.48294 | 6433.45302 | 7097.109002 | Ppp1r1a |
| 4.058189127 | 7.93E-17 | 3.74E-15 | 5.62155146 | 9.116700669 | 7.360054421 | 61.96840048 | 142.9467144 | 164.9539357 | Afp |
| 1.149487428 | 1.13E-16 | 5.25E-15 | 1107.445638 | 1042.950556 | 1016.738946 | 1980.254915 | 2647.066836 | 2399.329974 | Clcn5 |
| 1.731590005 | 1.17E-16 | 5.44E-15 | 589.3259781 | 378.3430777 | 542.5411545 | 1397.022911 | 1523.91408 | 2096.687216 | Gpr3 |
| 1.032556721 | 1.22E-16 | 5.64E-15 | 3238.950566 | 2649.313214 | 3327.796035 | 5921.627446 | 6377.295383 | 6553.169991 | Znrf1 |
| 3.282935718 | 1.25E-16 | 5.74E-15 | 20.61235535 | 5.470020401 | 22.08016326 | 151.2758012 | 122.5257552 | 192.219049 | Dppa1 |
| 1.214528364 | 1.27E-16 | 5.83E-15 | 3536.892794 | 3974.881492 | 3299.407253 | 8670.108267 | 6939.722634 | 9485.532931 | Alcam |
| 1.108882113 | 1.45E-16 | 6.59E-15 | 6598.764489 | 8284.345898 | 6355.932711 | 14287.36151 | 15271.47399 | 16254.09732 | Impact |
| 1.034806789 | 1.55E-16 | 7.00E-15 | 289.5099002 | 309.0561527 | 299.6593586 | 610.5710047 | 610.076156 | 620.2813285 | Lrrtm3 |
| 2.404736261 | 1.63E-16 | 7.36E-15 | 110.5571787 | 73.84527542 | 78.85772594 | 458.3839035 | 293.5512885 | 646.1831862 | Gm17501 |
| 1.384624405 | 2.07E-16 | 9.15E-15 | 524.6781363 | 433.0432818 | 565.6727541 | 1382.442111 | 1091.670444 | 1505.034256 | Ccdc63 |
| 1.199394757 | 2.38E-16 | 1.04E-14 | 3504.10041 | 2777.858694 | 3526.517504 | 6475.69785 | 7789.74506 | 8259.966086 | Slc41a2 |
| 1.335798 | 2.44E-16 | 1.07E-14 | 8205.591281 | 8956.246737 | 7882.618285 | 21683.47237 | 16382.71452 | 25156.15682 | Oxr1 |
| 1.602481614 | 2.72E-16 | 1.18E-14 | 125.5479826 | 132.1921597 | 134.5838523 | 311.6646024 | 445.8576091 | 434.8785578 | Perm1 |
| 1.392645365 | 2.81E-16 | 1.22E-14 | 420.6794343 | 540.6203496 | 402.7001205 | 1282.19911 | 1003.179621 | 1300.545906 | Elavl4 |
| 1.089535711 | 2.93E-16 | 1.27E-14 | 2745.190963 | 2439.629099 | 2738.991681 | 4765.187737 | 6072.682741 | 6025.590048 | Dnajb2 |
| 3.501700932 | 3.24E-16 | 1.40E-14 | 31.85545827 | 14.58672107 | 7.360054421 | 127.582001 | 253.5602434 | 233.1167191 | Serpina3g |
| 3.106291157 | 3.33E-16 | 1.42E-14 | 11.24310292 | 13.675051 | 11.5657998 | 74.72660058 | 110.613529 | 130.872544 | Mypn |
| 1.221572168 | 3.34E-16 | 1.42E-14 | 278.2667973 | 240.6808977 | 258.6533411 | 544.0461042 | 601.567423 | 670.7217882 | Gm5124 |
| 1.373669748 | 3.76E-16 | 1.60E-14 | 2974.737648 | 2118.721235 | 2975.564859 | 6085.661447 | 6835.916091 | 7988.678209 | Nrsn1 |
| 1.489393821 | 3.83E-16 | 1.62E-14 | 3456.317223 | 2427.777388 | 3570.677831 | 8381.226165 | 7600.000315 | 10566.59467 | Tyro3 |
| 2.084023423 | 4.82E-16 | 2.01E-14 | 47.78318741 | 43.76016321 | 62.03474441 | 166.7679013 | 251.0076235 | 231.7534634 | Opn3 |
| 1.794576112 | 5.14E-16 | 2.14E-14 | 386.9501255 | 263.4726493 | 419.523102 | 1159.173609 | 986.1621546 | 1567.744017 | Kcns2 |
| 1.918674115 | 6.46E-16 | 2.66E-14 | 229.5466846 | 151.3372311 | 207.1329601 | 559.5382043 | 687.5056263 | 978.8175689 | Rem2 |
| 1.207548591 | 7.44E-16 | 3.03E-14 | 2008.767722 | 1789.608341 | 2241.662289 | 3864.82333 | 4988.670157 | 5095.849683 | Hmga1 |
| 1.507656524 | 1.01E-15 | 4.10E-14 | 929.4298414 | 692.8692508 | 977.8358017 | 2363.912218 | 2021.674961 | 3010.068513 | Mir670hg |
| 2.506765485 | 1.03E-15 | 4.15E-14 | 84.3232719 | 91.16700669 | 126.1723615 | 616.0388048 | 309.7178812 | 789.3250312 | Myh15 |
| 5.122797036 | 1.60E-15 | 6.36E-14 | 3.747700973 | 3.646680267 | 0 | 58.32320045 | 121.6748819 | 85.88510702 | Tcap |
| 3.134719431 | 1.63E-15 | 6.48E-14 | 13.11695341 | 11.85171087 | 10.51436346 | 70.17010054 | 111.4644023 | 132.2357997 | Olfr237-ps1 |
| 3.10326637 | 1.67E-15 | 6.60E-14 | 29.98160779 | 16.4100612 | 15.77154519 | 101.1543008 | 233.1392842 | 201.7618387 | Abcb1a |
| 1.299033636 | 1.88E-15 | 7.38E-14 | 8658.126174 | 7226.80862 | 8347.35315 | 18518.52744 | 16561.39791 | 24552.23456 | Fat3 |
| 4.577858805 | 1.92E-15 | 7.50E-14 | 4.684626217 | 2.735010201 | 2.102872692 | 54.67800042 | 91.89431639 | 83.15859569 | Sh2d1b1 |
| 1.346587714 | 2.25E-15 | 8.74E-14 | 680.2077267 | 563.4121013 | 651.8905344 | 1288.57821 | 1908.508812 | 1622.274244 | Pvr |
| 1.17570685 | 2.49E-15 | 9.58E-14 | 7831.758109 | 6659.749838 | 7444.169329 | 14484.20231 | 15202.55325 | 19872.17786 | Trank1 |
| 1.263254613 | 2.78E-15 | 1.07E-13 | 1049.356273 | 1321.921597 | 1053.539219 | 2849.635122 | 2275.235204 | 3101.406642 | Idi1 |
| 2.371888329 | 3.00E-15 | 1.14E-13 | 22.48620584 | 40.11348294 | 21.02872692 | 131.227201 | 155.7098139 | 148.5948677 | Tmem196 |
| 1.194997825 | 3.11E-15 | 1.17E-13 | 185.5111982 | 192.3623841 | 221.853069 | 482.9890037 | 435.6471295 | 452.6008814 | Syna |
| 2.159581059 | 3.29E-15 | 1.24E-13 | 83.38634666 | 73.84527542 | 67.29192614 | 267.9222021 | 260.3672298 | 479.8659948 | Grm8 |
| 1.857629381 | 3.58E-15 | 1.34E-13 | 820.7465132 | 589.8505333 | 903.1838211 | 2395.807718 | 2133.990236 | 3858.013538 | Nfil3 |
| 1.686073191 | 3.88E-15 | 1.43E-13 | 389.7609012 | 255.2676187 | 427.9345928 | 920.4130071 | 1227.810172 | 1303.272418 | Cidea |
| 1.661662285 | 4.03E-15 | 1.48E-13 | 282.9514235 | 206.9491052 | 296.5050495 | 653.402105 | 798.9700286 | 1037.437563 | Rasd1 |
| 1.793965016 | 4.48E-15 | 1.63E-13 | 272.6452458 | 193.2740542 | 276.527759 | 821.9926063 | 639.8567215 | 1115.143136 | 6430573F11Rik |
| 1.357349609 | 4.76E-15 | 1.73E-13 | 4110.291043 | 2982.984459 | 4112.167549 | 8832.319668 | 8415.136936 | 11464.98016 | Map3k9 |
| 1.505902823 | 5.94E-15 | 2.14E-13 | 522.8042858 | 325.4662139 | 499.4322643 | 1119.076409 | 1330.765841 | 1376.888224 | Tecta |
| 3.498978144 | 6.09E-15 | 2.19E-13 | 9.369252434 | 8.205030602 | 9.462927113 | 71.08140055 | 82.53471009 | 154.0478904 | Asb11 |
| 2.288251801 | 6.23E-15 | 2.23E-13 | 52.46781363 | 88.43199649 | 42.05745383 | 247.8736019 | 269.7268361 | 381.7115868 | Hectd2os |
| 1.023599543 | 6.25E-15 | 2.24E-13 | 802.0080083 | 664.6074787 | 770.7028415 | 1373.329111 | 1574.115605 | 1601.825409 | Abtb2 |
| 1.647763507 | 6.50E-15 | 2.32E-13 | 24117.39269 | 13556.53389 | 23932.7941 | 57582.31354 | 62280.52206 | 73179.5642 | Vgf |
| 1.245524543 | 6.94E-15 | 2.46E-13 | 4556.267458 | 3331.242424 | 4657.863012 | 9197.750971 | 9287.282068 | 11261.85507 | Gng3 |
| 2.770390624 | 7.47E-15 | 2.63E-13 | 37.47700973 | 27.35010201 | 29.44021768 | 125.759401 | 316.5248676 | 200.398583 | Cbln1 |
| 1.440869647 | 9.22E-15 | 3.21E-13 | 276.3929468 | 235.2108773 | 326.9967036 | 622.4179048 | 779.3999427 | 875.2101382 | Mrm1 |
| 3.372848846 | 9.30E-15 | 3.23E-13 | 7.495401947 | 10.9400408 | 17.87441788 | 71.08140055 | 144.648461 | 158.1376574 | D5Ertd615e |
| 2.680348009 | 9.47E-15 | 3.28E-13 | 20.61235535 | 13.675051 | 13.6686725 | 92.04130071 | 91.04344309 | 126.782777 | Sowahb |
| 1.208543372 | 1.02E-14 | 3.49E-13 | 292.3206759 | 260.7376391 | 294.4021768 | 654.3134051 | 565.8307444 | 741.6110828 | Shc4 |
| 1.579584104 | 1.03E-14 | 3.52E-13 | 370.0854711 | 273.5010201 | 405.8544295 | 878.4932068 | 953.8289692 | 1305.998929 | Lrrc55 |
| 1.266530736 | 1.09E-14 | 3.71E-13 | 7949.81069 | 6607.784645 | 8080.288318 | 14323.81351 | 21142.49976 | 18995.60446 | Herc3 |
| 1.106151914 | 1.26E-14 | 4.21E-13 | 9355.198555 | 7116.496542 | 9163.267754 | 17709.29304 | 16969.81709 | 20507.455 | Arpp21 |
| 2.497077047 | 1.34E-14 | 4.49E-13 | 28.1077573 | 31.90845234 | 36.80027211 | 112.0899009 | 217.8235648 | 216.7576511 | A930003O13Rik |
| 1.387551282 | 1.57E-14 | 5.21E-13 | 1738.933252 | 1189.729437 | 1659.166554 | 3695.321529 | 3536.229434 | 4774.121346 | Fabp3 |
| 1.621139918 | 1.73E-14 | 5.68E-13 | 76.82786996 | 61.08189448 | 77.80628959 | 230.5589018 | 194.8499857 | 238.5697417 | Cntn3 |
| 1.216725065 | 1.81E-14 | 5.93E-13 | 2856.685067 | 3860.011063 | 2755.814663 | 6713.547152 | 7140.528733 | 8167.264701 | Cep170 |
| 5.757612193 | 1.87E-14 | 6.11E-13 | 0.936925243 | 1.823340134 | 4.205745383 | 37.36330029 | 188.0429993 | 143.141845 | Slc1a7 |
| 1.297151863 | 1.89E-14 | 6.18E-13 | 2127.757228 | 1928.182191 | 2159.650254 | 3991.494031 | 6275.190587 | 5005.874809 | Megf11 |
| 1.52978201 | 2.08E-14 | 6.74E-13 | 1212.381265 | 1663.797872 | 1162.888599 | 3202.308225 | 3520.913715 | 4945.89156 | Snhg11 |
| 1.886533824 | 2.09E-14 | 6.77E-13 | 250.15904 | 140.3971903 | 207.1329601 | 819.2587063 | 534.3484323 | 857.4878145 | Gimap6 |
| 1.518827365 | 2.42E-14 | 7.76E-13 | 90.88174861 | 101.1953774 | 78.85772594 | 229.6476018 | 261.2181031 | 289.0102014 | Phex |
| 1.11454785 | 2.71E-14 | 8.58E-13 | 3357.940072 | 2571.821259 | 3444.505469 | 6102.064847 | 6858.88967 | 7337.042 | Galnt14 |
| 1.836296064 | 3.01E-14 | 9.51E-13 | 572.4613237 | 459.4817137 | 588.8043537 | 1909.173515 | 1246.529384 | 2635.173204 | Phf21b |
| 1.40750281 | 3.05E-14 | 9.60E-13 | 544.3535664 | 727.5127134 | 484.7121554 | 1705.042313 | 1293.327416 | 1665.898425 | B230209E15Rik |
| 1.113218064 | 3.37E-14 | 1.05E-12 | 2643.066112 | 1991.999096 | 2593.893465 | 4801.639737 | 5045.678668 | 5792.473329 | Rapgef5 |
| 1.615143617 | 3.82E-14 | 1.19E-12 | 111.494104 | 107.5770679 | 121.9666161 | 262.454402 | 395.6560844 | 387.1646094 | Gm11549 |
| 1.040231797 | 4.01E-14 | 1.24E-12 | 7676.228519 | 6038.902523 | 7640.787925 | 13191.0676 | 16000.6724 | 14725.88771 | Tagln3 |
| 1.411170448 | 4.55E-14 | 1.40E-12 | 1525.314296 | 1006.483754 | 1495.142484 | 2975.394523 | 3725.123307 | 4009.334917 | Sik1 |
| 1.396387298 | 4.76E-14 | 1.46E-12 | 6370.15473 | 4075.165199 | 6047.861861 | 12245.13819 | 16124.04903 | 15046.2528 | Stmn4 |
| 1.688558017 | 4.84E-14 | 1.49E-12 | 234.2313108 | 184.1573535 | 232.3674324 | 484.8116037 | 771.742083 | 842.4920022 | Cdyl2 |
| 2.479558746 | 4.89E-14 | 1.50E-12 | 128.3587583 | 54.70020401 | 135.6352886 | 378.1895029 | 695.163486 | 702.0766685 | Lipg |
| 1.529390413 | 5.21E-14 | 1.59E-12 | 175.2050205 | 169.5706324 | 203.9786511 | 476.6099037 | 437.3488761 | 673.4482995 | Col22a1 |
| 3.236642783 | 5.87E-14 | 1.79E-12 | 7.495401947 | 5.470020401 | 10.51436346 | 66.52490051 | 66.36811739 | 87.24836269 | Wnt9b |
| 2.029501701 | 6.06E-14 | 1.84E-12 | 234.2313108 | 137.6621801 | 229.2131234 | 531.2879041 | 1015.091847 | 906.5650186 | Kcnk3 |
| 1.644299327 | 6.37E-14 | 1.93E-12 | 163.0249923 | 122.163789 | 159.8183246 | 344.4714027 | 530.0940658 | 516.6738978 | Trim66 |
| 1.150176144 | 7.70E-14 | 2.28E-12 | 4535.655103 | 3340.359125 | 4778.778192 | 8845.989168 | 9109.449549 | 10130.35286 | Tmem35a |
| 1.213927615 | 8.45E-14 | 2.49E-12 | 3465.686475 | 3585.598373 | 3391.933652 | 6971.445054 | 7064.801009 | 10195.78913 | Akap13 |
| 2.427704508 | 9.24E-14 | 2.71E-12 | 66.52169228 | 81.13863595 | 58.88043537 | 385.479903 | 199.9552255 | 530.3064545 | D330045A20Rik |
| 1.577498482 | 1.03E-13 | 2.99E-12 | 3968.815331 | 3663.090329 | 3818.816808 | 13357.8355 | 7315.808632 | 13504.41064 | Tfrc |
| 1.06087738 | 1.39E-13 | 3.99E-12 | 3751.448674 | 4504.5618 | 3416.116688 | 8662.817867 | 7133.721746 | 8558.519077 | Pgap1 |
| 1.207347222 | 1.52E-13 | 4.34E-12 | 1143.985722 | 908.9350567 | 1014.636074 | 1947.448115 | 2787.46093 | 2347.526259 | Gpsm1 |
| 1.091727557 | 1.74E-13 | 4.88E-12 | 4307.982269 | 3586.510043 | 4518.021978 | 8311.056064 | 7840.797458 | 10306.21284 | Brinp1 |
| 1.141083738 | 1.83E-13 | 5.10E-12 | 458.156444 | 380.1664179 | 479.4549737 | 860.2672066 | 947.8728561 | 1100.147323 | Sema4c |
| 1.125959576 | 1.88E-13 | 5.22E-12 | 2632.759934 | 3457.052894 | 2519.241485 | 5999.999246 | 5821.675118 | 6973.052737 | Armcx3 |
| 2.588186628 | 1.93E-13 | 5.37E-12 | 27.17083206 | 45.58350334 | 28.38878134 | 145.8080011 | 161.665927 | 305.3692694 | A230004M16Rik |
| 1.422878872 | 2.10E-13 | 5.79E-12 | 1030.617768 | 1117.707502 | 973.6300563 | 2872.417622 | 2007.210114 | 3496.750786 | Crem |
| 1.007848569 | 2.21E-13 | 6.08E-12 | 8846.448148 | 7489.369599 | 8864.659832 | 14490.58141 | 17218.2721 | 18969.70261 | Gramd1b |
| 1.873692391 | 2.25E-13 | 6.19E-12 | 34.666234 | 41.02515301 | 53.62325364 | 169.5018013 | 163.3676736 | 137.6888224 | Gm43951 |
| 1.898586547 | 2.26E-13 | 6.20E-12 | 2630.886083 | 4539.205263 | 2416.200723 | 11589.00219 | 8641.469234 | 15517.93926 | Dmxl1 |
| 4.364413153 | 2.72E-13 | 7.36E-12 | 5.62155146 | 1.823340134 | 2.102872692 | 45.56500035 | 74.02597709 | 79.06882869 | Tnni3k |
| 2.425273493 | 2.78E-13 | 7.52E-12 | 55.27858936 | 34.64346254 | 28.38878134 | 143.0741011 | 282.4899356 | 211.3046284 | Akr1c18 |
| 2.148592537 | 2.84E-13 | 7.63E-12 | 107.746403 | 79.31529582 | 100.9378892 | 258.809202 | 602.4182963 | 414.4297228 | Lamb3 |
| 1.624745345 | 3.71E-13 | 9.81E-12 | 93.69252434 | 92.99034682 | 85.16634402 | 221.4459017 | 270.5777094 | 350.3567064 | Hrh2 |
| 1.861976855 | 4.09E-13 | 1.08E-11 | 37.47700973 | 36.46680267 | 31.54309038 | 121.2029009 | 122.5257552 | 141.7785894 | Tekt5 |
| 1.262878832 | 4.11E-13 | 1.08E-11 | 168.6465438 | 145.8672107 | 161.9211973 | 350.8505027 | 438.1997494 | 351.7199621 | Rspo2 |
| 1.199182068 | 4.25E-13 | 1.11E-11 | 1462.540305 | 1182.436077 | 1469.908012 | 2959.902423 | 2675.145655 | 3817.115868 | Pmvk |
| 1.016763843 | 4.61E-13 | 1.19E-11 | 6662.475406 | 5735.316391 | 6899.525302 | 12251.51729 | 11628.03452 | 15170.30906 | Srxn1 |
| 1.667143123 | 4.90E-13 | 1.27E-11 | 478.7687994 | 337.3179247 | 494.1750826 | 944.1068073 | 1619.21189 | 1597.735642 | Cgref1 |
| 1.261913512 | 5.09E-13 | 1.31E-11 | 13306.21231 | 8981.773499 | 13615.04924 | 26684.68681 | 26996.50806 | 32419.58302 | Lingo1 |
| 1.305621524 | 5.70E-13 | 1.45E-11 | 4784.877218 | 3208.166965 | 4728.309247 | 8869.682968 | 10667.39856 | 11909.40151 | Rab15 |
| 1.162041338 | 5.73E-13 | 1.46E-11 | 3679.305431 | 2642.019854 | 3609.580975 | 7498.176458 | 6476.847559 | 8249.060041 | Slco1c1 |
| 1.079749486 | 6.01E-13 | 1.52E-11 | 1813.887271 | 1358.3884 | 1789.544661 | 3268.833125 | 3896.999713 | 3318.164293 | Lzts3 |
| 1.390846863 | 6.46E-13 | 1.63E-11 | 328.8607604 | 501.4185368 | 350.1283032 | 976.9136075 | 922.3466571 | 1201.028243 | Cntnap5a |
| 1.875310066 | 8.58E-13 | 2.14E-11 | 267.0236944 | 209.6841154 | 239.7274869 | 897.6305069 | 533.497559 | 1201.028243 | Cubn |
| 2.251937182 | 9.38E-13 | 2.31E-11 | 111.494104 | 41.93682308 | 107.2465073 | 438.3353034 | 327.5862205 | 474.4129721 | Trim72 |
| 2.159062382 | 1.01E-12 | 2.48E-11 | 35.60315925 | 43.76016321 | 31.54309038 | 144.8967011 | 128.4818683 | 226.3004407 | Ccdc110 |
| 1.819569081 | 1.03E-12 | 2.53E-11 | 62.77399131 | 110.3120781 | 62.03474441 | 267.0109021 | 251.8584968 | 314.9120591 | Gm43031 |
| 1.340944867 | 1.04E-12 | 2.55E-11 | 1232.99362 | 1766.81659 | 1242.797761 | 3461.117427 | 2995.074016 | 4296.981862 | Rasa2 |
| 1.854025794 | 1.05E-12 | 2.55E-11 | 222.9882079 | 289.9110813 | 227.1102507 | 902.187007 | 556.4711381 | 1221.477078 | B230216N24Rik |
| 1.288903888 | 1.08E-12 | 2.60E-11 | 1216.128966 | 858.793203 | 1313.243996 | 2375.759118 | 2795.969663 | 3106.859665 | Rell2 |
| 1.397897782 | 1.10E-12 | 2.67E-11 | 342.9146391 | 461.3050538 | 262.8590865 | 946.8407073 | 935.9606299 | 932.4668762 | Gm16485 |
| 2.580234664 | 1.18E-12 | 2.85E-11 | 11.24310292 | 13.675051 | 24.18303596 | 90.2187007 | 85.93820329 | 115.8767317 | Uox |
| 1.602705449 | 1.41E-12 | 3.37E-11 | 64.64784179 | 87.52032642 | 97.78358017 | 215.9781017 | 289.296922 | 252.2022984 | Gm42433 |
| 2.527011647 | 1.47E-12 | 3.51E-11 | 13.11695341 | 14.58672107 | 22.08016326 | 73.81530057 | 100.4030494 | 111.7869647 | Oxgr1 |
| 1.950623612 | 1.83E-12 | 4.28E-11 | 75.89094471 | 82.96197608 | 87.26921671 | 194.1069015 | 372.6825053 | 385.8013538 | Plekhg4 |
| 1.34467234 | 1.97E-12 | 4.56E-11 | 1452.234127 | 1804.195062 | 1508.811156 | 3577.763828 | 3262.248232 | 5268.983153 | Unc13b |
| 1.022301634 | 2.21E-12 | 5.06E-11 | 284.825274 | 282.6177207 | 281.7849407 | 507.5941039 | 631.3479885 | 586.1999368 | Moap1 |
| 2.879606254 | 2.43E-12 | 5.50E-11 | 15.92772914 | 9.116700669 | 19.97729057 | 74.72660058 | 99.55217609 | 156.7744017 | Pde6b |
| 1.063538482 | 2.59E-12 | 5.84E-11 | 18133.25116 | 13221.03931 | 18267.65507 | 32697.44425 | 32944.11242 | 38071.64101 | Nnat |
| 1.020125133 | 2.91E-12 | 6.51E-11 | 22164.84048 | 16928.80147 | 22373.514 | 36924.96499 | 45307.30147 | 42427.24287 | Stx1b |
| 1.546467756 | 3.51E-12 | 7.76E-11 | 549.9751179 | 467.6867443 | 556.209827 | 1122.721609 | 1363.099026 | 2115.772795 | Mirg |
| 1.096392308 | 3.58E-12 | 7.90E-11 | 256.7175167 | 258.914299 | 197.670033 | 517.618404 | 515.6292197 | 493.4985515 | Pcdhb2 |
| 1.016838054 | 4.00E-12 | 8.76E-11 | 1039.98702 | 881.5849547 | 992.5559105 | 1699.574513 | 1917.868418 | 2283.453242 | Creb3l1 |
| 1.718599982 | 4.02E-12 | 8.81E-11 | 2203.648172 | 4118.925362 | 1937.797185 | 8591.736466 | 8377.698511 | 10221.69099 | Gabrb2 |
| 1.188968862 | 4.32E-12 | 9.40E-11 | 538.7320149 | 672.8125093 | 493.1236462 | 1523.693612 | 1138.468475 | 1225.566845 | Sema3a |
| 1.82328629 | 4.34E-12 | 9.43E-11 | 61.83706606 | 39.20181288 | 69.39479883 | 167.6792013 | 228.8849177 | 204.4883501 | Neu2 |
| 1.042628619 | 4.50E-12 | 9.71E-11 | 394.4455275 | 380.1664179 | 397.4429387 | 672.5394052 | 878.9521188 | 864.3040929 | Armcx6 |
| 1.209866764 | 4.54E-12 | 9.79E-11 | 20808.17273 | 14904.89392 | 21648.02293 | 37455.34159 | 44084.59654 | 51146.62611 | Hpcal4 |
| 1.015252218 | 5.16E-12 | 1.11E-10 | 583.7044266 | 640.904057 | 627.7074985 | 1078.979208 | 1209.941832 | 1460.046819 | Idnk |
| 1.025994609 | 5.51E-12 | 1.18E-10 | 461.904145 | 346.4346254 | 419.523102 | 793.7423061 | 820.2418611 | 887.4794392 | Clvs2 |
| 1.304840585 | 5.66E-12 | 1.21E-10 | 141.4757117 | 175.9523229 | 126.1723615 | 342.6488026 | 342.0510666 | 415.7929784 | Far2 |
| 3.564474976 | 6.58E-12 | 1.39E-10 | 7.495401947 | 4.558350334 | 7.360054421 | 41.91980032 | 79.13121689 | 109.0604534 | Ccdc36 |
| 1.032045144 | 7.06E-12 | 1.48E-10 | 870.4035511 | 716.5726726 | 938.9326569 | 1564.702112 | 1651.545075 | 1950.818859 | Maml3 |
| 1.483673783 | 7.20E-12 | 1.51E-10 | 668.9646238 | 437.6016321 | 659.2505889 | 1231.16631 | 1966.368196 | 1739.514231 | Hcn3 |
| 2.908531308 | 7.76E-12 | 1.61E-10 | 7.495401947 | 11.85171087 | 6.308618075 | 60.14580046 | 61.26287759 | 73.61580602 | Pbp2 |
| 1.524059547 | 8.11E-12 | 1.68E-10 | 308.2484051 | 472.2450946 | 301.7622313 | 1023.389908 | 818.5401145 | 1276.007304 | 5330434G04Rik |
| 2.760015524 | 8.15E-12 | 1.68E-10 | 10.30617768 | 10.02837074 | 15.77154519 | 62.87970049 | 74.02597709 | 107.6971977 | Mcub |
| 2.05613321 | 9.43E-12 | 1.93E-10 | 68.39554277 | 30.08511221 | 54.67468999 | 178.6148014 | 199.9552255 | 259.0185767 | Calcr |
| 1.006512302 | 1.00E-11 | 2.04E-10 | 11006.06083 | 8060.075061 | 10993.81843 | 18800.11915 | 19878.10203 | 21713.93626 | Dner |
| 1.351351486 | 1.01E-11 | 2.06E-10 | 154.5926652 | 144.9555406 | 161.9211973 | 331.7132026 | 357.366786 | 492.1352958 | Gpr150 |
| 1.488719015 | 1.04E-11 | 2.10E-10 | 1228.308994 | 796.7996384 | 1253.312124 | 2862.393322 | 2424.988905 | 3915.270276 | Grasp |
| 1.688461681 | 1.04E-11 | 2.11E-10 | 6207.129737 | 11371.26074 | 5610.464342 | 24236.93499 | 20799.59782 | 29709.43075 | Rock2 |
| 2.151870259 | 1.16E-11 | 2.32E-10 | 19.67543011 | 30.99678227 | 22.08016326 | 91.1300007 | 103.8065426 | 130.872544 | Rhcg |
| 2.710964233 | 1.27E-11 | 2.53E-10 | 13.11695341 | 20.96841154 | 12.61723615 | 74.72660058 | 145.4993343 | 85.88510702 | Prom2 |
| 1.182527242 | 1.29E-11 | 2.57E-10 | 311.0591808 | 387.4597784 | 284.9392497 | 707.1688055 | 663.6811739 | 867.0306042 | Kbtbd3 |
| 1.118666297 | 1.31E-11 | 2.61E-10 | 476.8949489 | 465.8634042 | 488.9179008 | 876.6706068 | 964.0394488 | 1273.280793 | Pnoc |
| 1.36467534 | 1.38E-11 | 2.72E-10 | 243.6005633 | 244.3275779 | 199.7729057 | 604.1919047 | 450.9628489 | 721.1622478 | Nmbr |
| 2.136428794 | 1.42E-11 | 2.81E-10 | 29.04468254 | 19.1450714 | 28.38878134 | 102.0656008 | 95.29780959 | 140.4153337 | Gimap4 |
| 1.409337209 | 1.47E-11 | 2.90E-10 | 109.6202535 | 82.96197608 | 103.0407619 | 218.7120017 | 280.788189 | 286.2836901 | Dmp1 |
| 1.283243959 | 1.52E-11 | 2.99E-10 | 30005.96784 | 18696.52973 | 29963.83298 | 55310.44263 | 66923.73766 | 69227.48603 | Slc6a17 |
| 1.022743646 | 1.53E-11 | 3.00E-10 | 25467.50197 | 20060.38815 | 26011.48376 | 44003.03214 | 44865.69823 | 56486.49856 | Pam |
| 1.202106235 | 1.64E-11 | 3.21E-10 | 328.8607604 | 443.9833226 | 284.9392497 | 778.250206 | 827.0488475 | 831.5859569 | Chml |
| 1.086689583 | 1.76E-11 | 3.44E-10 | 2816.397282 | 3376.825928 | 2871.472661 | 6619.683251 | 5117.152025 | 7521.081515 | Epha7 |
| 1.744345886 | 1.99E-11 | 3.85E-10 | 40.28778546 | 49.23018361 | 56.77756268 | 135.783701 | 156.5606872 | 199.0353274 | Atp8b1 |
| 1.677010251 | 2.02E-11 | 3.90E-10 | 74.01709423 | 51.05352374 | 70.44623517 | 170.4131013 | 252.7093701 | 200.398583 | Adgrd1 |
| 2.205978575 | 2.14E-11 | 4.11E-10 | 30.91853303 | 44.67183328 | 21.02872692 | 139.4289011 | 117.4205154 | 193.5823047 | Sytl5 |
| 1.347201303 | 2.38E-11 | 4.56E-10 | 2014.389273 | 3350.387496 | 1986.163257 | 6096.597047 | 5850.60481 | 6759.021597 | Atp11b |
| 1.073955293 | 2.51E-11 | 4.77E-10 | 3397.290932 | 4074.253529 | 3249.989745 | 7729.64666 | 6100.76156 | 8746.648359 | Cnr1 |
| 1.086866463 | 2.58E-11 | 4.88E-10 | 12303.7023 | 8556.023577 | 12065.23207 | 20611.78356 | 24163.09997 | 25161.60985 | Lynx1 |
| 2.902180959 | 2.68E-11 | 5.07E-10 | 11.24310292 | 6.381690468 | 10.51436346 | 50.12150039 | 75.72772369 | 84.52185135 | 1700023F02Rik |
| 1.051303737 | 2.74E-11 | 5.16E-10 | 5524.111235 | 4056.931798 | 5655.676104 | 9229.646471 | 11288.53607 | 11057.36672 | Rap1gap |
| 1.112066891 | 2.76E-11 | 5.18E-10 | 534.0473887 | 414.8098804 | 541.4897181 | 914.9452071 | 1221.003185 | 1083.788255 | Hsd11b1 |
| 1.028686891 | 3.05E-11 | 5.69E-10 | 344.7884896 | 410.2515301 | 358.5397939 | 672.5394052 | 731.7510379 | 872.4836269 | Zfp382 |
| 3.946695138 | 3.29E-11 | 6.09E-10 | 1.873850487 | 6.381690468 | 5.257181729 | 55.58930043 | 42.54366499 | 111.7869647 | Fam124b |
| 1.444665177 | 3.34E-11 | 6.18E-10 | 512.4981081 | 393.8414689 | 501.535137 | 877.5819068 | 1554.545519 | 1400.06357 | Slc9a5 |
| 2.309952584 | 3.42E-11 | 6.30E-10 | 84.3232719 | 50.14185368 | 69.39479883 | 172.2357013 | 475.6381746 | 362.6260074 | Klhl40 |
| 1.118532172 | 3.49E-11 | 6.41E-10 | 2501.5904 | 1733.084797 | 2419.355032 | 4175.576632 | 4927.40728 | 5345.32547 | Pdgfb |
| 1.899361143 | 3.63E-11 | 6.65E-10 | 59.96321558 | 103.0187176 | 58.88043537 | 287.9708022 | 201.6569721 | 342.1771724 | Ndst4 |
| 1.082668841 | 3.65E-11 | 6.69E-10 | 4604.050646 | 3336.712445 | 4443.369998 | 8179.828863 | 7842.499205 | 10209.42169 | Ttbk1 |
| 1.175639836 | 3.86E-11 | 7.03E-10 | 274.5190963 | 252.5326085 | 322.7909582 | 527.6427041 | 736.0054044 | 654.3627202 | Grip2 |
| 1.353196642 | 3.98E-11 | 7.23E-10 | 85.26019715 | 93.90201689 | 84.11490767 | 202.3086016 | 209.3148318 | 264.4715994 | Prlr |
| 3.854168616 | 4.20E-11 | 7.58E-10 | 1.873850487 | 6.381690468 | 6.308618075 | 35.54070027 | 78.28034359 | 96.79115236 | 9230020A06Rik |
| 3.394283215 | 4.62E-11 | 8.28E-10 | 2.81077573 | 8.205030602 | 5.257181729 | 47.38760037 | 51.90327129 | 73.61580602 | Muc16 |
| 1.201855919 | 5.06E-11 | 9.01E-10 | 4446.647205 | 2830.735558 | 4320.351945 | 8181.651463 | 8433.005275 | 10064.91659 | Gpr26 |
| 8.381263026 | 6.08E-11 | 1.07E-09 | 0 | 0 | 0 | 27.33900021 | 75.72772369 | 74.97906169 | Drd4 |
| 1.094256785 | 6.55E-11 | 1.15E-09 | 8856.754326 | 6035.255843 | 8525.045892 | 14798.60081 | 16615.8538 | 18582.538 | Syt7 |
| 1.333078664 | 6.65E-11 | 1.16E-09 | 83.38634666 | 82.05030602 | 101.9893255 | 195.9295015 | 242.4988905 | 234.4799747 | Gm32391 |
| 1.301887398 | 6.77E-11 | 1.18E-09 | 872.2774016 | 1172.407706 | 761.2399144 | 2178.918317 | 1915.315798 | 2830.118765 | Fam135b |
| 1.143666549 | 6.85E-11 | 1.19E-09 | 979.0868793 | 691.0459107 | 941.0355296 | 1609.355812 | 1998.701381 | 2162.123488 | Syt12 |
| 1.170435701 | 7.83E-11 | 1.35E-09 | 1102.761011 | 732.9827338 | 1063.002146 | 1945.625515 | 2094.850064 | 2485.215081 | Rasl11b |
| 1.046641522 | 8.05E-11 | 1.39E-09 | 1600.268316 | 2216.269933 | 1538.251374 | 3827.46003 | 3400.089706 | 3837.564703 | Micu3 |
| 1.21294725 | 8.44E-11 | 1.45E-09 | 342.9146391 | 409.33986 | 323.8423945 | 759.1129059 | 702.8213457 | 1038.800818 | Cntn5 |
| 1.355728605 | 8.81E-11 | 1.51E-09 | 300.7530031 | 166.8356222 | 228.1616871 | 599.6354046 | 610.076156 | 569.8408688 | Gm26833 |
| 2.085130528 | 8.81E-11 | 1.51E-09 | 55.27858936 | 28.26177207 | 41.00601749 | 120.2916009 | 201.6569721 | 207.2148614 | Adamts13 |
| 2.363903065 | 9.01E-11 | 1.54E-09 | 28.1077573 | 28.26177207 | 34.69739941 | 88.39610068 | 223.7796779 | 155.411146 | Nlrp10 |
| 1.573998184 | 1.03E-10 | 1.74E-09 | 103.998702 | 120.3404488 | 121.9666161 | 284.3256022 | 279.0864424 | 471.6864608 | Nrarp |
| 1.548166774 | 1.03E-10 | 1.75E-09 | 194.8804506 | 110.3120781 | 168.2298153 | 380.9234029 | 440.7523693 | 564.3878461 | Rhov |
| 1.870792023 | 1.05E-10 | 1.77E-09 | 132.1064593 | 77.49195568 | 100.9378892 | 241.4945019 | 404.1648174 | 492.1352958 | Ecel1 |
| 2.140105397 | 1.14E-10 | 1.91E-09 | 29.04468254 | 15.49839114 | 28.38878134 | 85.66220066 | 114.0170222 | 121.3297544 | Cd5 |
| 1.136615607 | 1.20E-10 | 2.00E-09 | 5484.760375 | 7709.082085 | 5051.100206 | 12534.9316 | 12357.23293 | 15227.5658 | Casc4 |
| 2.358145211 | 1.26E-10 | 2.11E-09 | 21.5492806 | 24.61509181 | 26.28590865 | 71.08140055 | 154.8589406 | 145.8683564 | Gm32200 |
| 1.347544673 | 1.27E-10 | 2.13E-09 | 106.8094777 | 75.66861555 | 111.4522527 | 238.7606018 | 236.5427774 | 272.6511334 | Dbh |
| 1.994189799 | 1.29E-10 | 2.16E-09 | 53.40473887 | 30.08511221 | 36.80027211 | 115.7351009 | 167.6220401 | 197.6720717 | Stac3 |
| 1.055284858 | 1.93E-10 | 3.14E-09 | 1892.588992 | 1842.485205 | 1789.544661 | 4181.955732 | 2901.477953 | 4401.952549 | Trpc4 |
| 1.231031247 | 2.23E-10 | 3.60E-09 | 3897.609012 | 2466.979201 | 4042.77275 | 7815.30886 | 7279.221081 | 9335.574808 | Crtac1 |
| 6.916129602 | 2.25E-10 | 3.63E-09 | 0.936925243 | 0 | 1.051436346 | 59.23450046 | 63.81549749 | 111.7869647 | Lce1f |
| 1.20890488 | 2.29E-10 | 3.69E-09 | 337.2930876 | 230.6525269 | 297.5564859 | 557.7156043 | 760.6807301 | 681.6278335 | Nov |
| 1.174515929 | 2.41E-10 | 3.86E-09 | 1518.755819 | 1971.942355 | 1359.507195 | 3729.950929 | 2932.109391 | 4291.52884 | Rnf217 |
| 1.939742727 | 2.64E-10 | 4.21E-09 | 34.666234 | 47.40684348 | 28.38878134 | 113.9125009 | 128.4818683 | 185.4027707 | Gm6634 |
| 1.163721173 | 2.65E-10 | 4.22E-09 | 8902.663662 | 5724.37635 | 9159.062009 | 16146.41352 | 17448.85876 | 19693.59137 | Ptpn5 |
| 1.849251278 | 2.88E-10 | 4.57E-09 | 38.41393498 | 31.90845234 | 34.69739941 | 94.77520073 | 154.0080673 | 129.5092884 | 4930539E08Rik |
| 1.101201951 | 3.08E-10 | 4.88E-09 | 988.4561318 | 690.1342406 | 937.8812205 | 1574.726412 | 1953.605097 | 2085.781171 | Kif17 |
| 2.174206961 | 3.59E-10 | 5.63E-09 | 42.16163595 | 46.49517341 | 46.26319922 | 224.1798017 | 106.3591625 | 280.8306674 | C4bp |
| 1.342273376 | 3.71E-10 | 5.80E-09 | 2241.125182 | 3426.967781 | 2107.078437 | 7116.341755 | 5049.933035 | 7552.436395 | Rps6ka3 |
| 2.405507466 | 3.93E-10 | 6.11E-09 | 21.5492806 | 13.675051 | 22.08016326 | 63.79100049 | 108.0609091 | 132.2357997 | Pou4f3 |
| 2.034797512 | 4.29E-10 | 6.64E-09 | 191.1327496 | 328.2012241 | 119.8637434 | 945.9294073 | 583.6990837 | 1094.694301 | Kif18a |
| 2.48449516 | 4.57E-10 | 7.05E-09 | 10.30617768 | 14.58672107 | 14.72010884 | 51.9441004 | 81.68383679 | 88.61161836 | Pip5kl1 |
| 1.249771543 | 4.75E-10 | 7.30E-09 | 1073.716329 | 1709.381375 | 1002.018838 | 3068.347124 | 2739.812026 | 3196.834539 | Lnpk |
| 1.007121505 | 4.92E-10 | 7.57E-09 | 965.9699259 | 829.6197608 | 1008.327456 | 1537.363112 | 1894.043966 | 2207.110925 | Fhod3 |
| 1.36272282 | 4.96E-10 | 7.60E-09 | 275.4560216 | 327.289554 | 315.4309038 | 728.1287056 | 582.8482104 | 1055.159886 | Abcb4 |
| 1.843588331 | 5.42E-10 | 8.26E-09 | 28.1077573 | 39.20181288 | 26.28590865 | 96.59780075 | 107.2100358 | 134.962311 | Gm32014 |
| 1.172060369 | 5.83E-10 | 8.84E-09 | 152.7188147 | 114.8704284 | 145.0982157 | 275.2126021 | 312.2705011 | 343.5404281 | Spag4 |
| 1.02889504 | 5.87E-10 | 8.90E-09 | 2123.072601 | 1543.457423 | 2238.50798 | 3879.40413 | 3639.185104 | 4531.461837 | Cyb5r1 |
| 1.127534983 | 5.98E-10 | 9.06E-09 | 1003.446936 | 732.0710637 | 1017.790383 | 1623.936613 | 2319.480615 | 2070.785358 | Kcnh2 |
| 1.377536136 | 6.20E-10 | 9.36E-09 | 606.1906325 | 331.8479043 | 574.0842448 | 1190.157809 | 1215.047072 | 1524.119836 | 1700016P03Rik |
| 2.047351222 | 6.51E-10 | 9.80E-09 | 22.48620584 | 23.70342174 | 25.2344723 | 92.04130071 | 75.72772369 | 129.5092884 | Klk8 |
| 3.952501661 | 6.63E-10 | 9.96E-09 | 1.873850487 | 3.646680267 | 3.154309038 | 30.98420024 | 46.79803149 | 57.25673801 | Far2os1 |
| 3.460376233 | 6.87E-10 | 1.03E-08 | 6.558476704 | 3.646680267 | 3.154309038 | 33.71810026 | 58.71025769 | 55.89348235 | 4930546K05Rik |
| 1.179975925 | 6.89E-10 | 1.03E-08 | 1312.632266 | 836.9131214 | 1360.558632 | 2331.105418 | 2825.750229 | 2794.674117 | Camk1g |
| 1.614490413 | 7.83E-10 | 1.16E-08 | 305.4376293 | 577.9988224 | 328.0481399 | 1231.16631 | 924.899277 | 1558.201227 | Gucy1a2 |
| 1.377825204 | 8.05E-10 | 1.19E-08 | 246.411339 | 417.5448906 | 235.5217415 | 708.0801055 | 774.2947029 | 858.8510702 | Rab27b |
| 1.218675787 | 8.13E-10 | 1.21E-08 | 30898.8576 | 18483.19894 | 30997.39491 | 63926.78419 | 54652.44292 | 68489.96471 | Rgs4 |
| 2.046367197 | 8.24E-10 | 1.22E-08 | 29.98160779 | 26.43843194 | 28.38878134 | 76.54920059 | 147.2010809 | 126.782777 | Akap3 |
| 1.090395568 | 8.58E-10 | 1.27E-08 | 771.0894753 | 592.5855435 | 755.9827327 | 1185.601309 | 1643.887215 | 1684.984004 | Scrt2 |
| 3.575695911 | 8.72E-10 | 1.29E-08 | 5.62155146 | 8.205030602 | 14.72010884 | 34.62940027 | 180.3851396 | 122.69301 | Krt16 |
| 1.020818399 | 9.01E-10 | 1.33E-08 | 137.7280108 | 128.5454794 | 128.2752342 | 256.075302 | 266.3233429 | 279.4674117 | Arsj |
| 1.05399738 | 9.12E-10 | 1.34E-08 | 3412.281736 | 2561.792888 | 3435.042542 | 5731.165744 | 5909.315068 | 7898.703335 | Rps6ka2 |
| 1.516138228 | 9.25E-10 | 1.36E-08 | 84.3232719 | 90.25533662 | 83.06347132 | 173.1470013 | 261.2181031 | 305.3692694 | Myo3b |
| 1.18390615 | 9.48E-10 | 1.39E-08 | 241.7267128 | 175.9523229 | 231.3159961 | 439.2466034 | 447.5593557 | 590.2897038 | Cyp4x1 |
| 1.71954421 | 9.57E-10 | 1.40E-08 | 48.72011266 | 55.61187408 | 35.74883576 | 117.5577009 | 161.665927 | 185.4027707 | Srpk3 |
| 1.132939731 | 9.74E-10 | 1.42E-08 | 214.5558807 | 216.0658058 | 181.8984878 | 387.302503 | 416.0770436 | 545.3022668 | C78859 |
| 3.089669243 | 9.81E-10 | 1.43E-08 | 17.80157962 | 22.79175167 | 7.360054421 | 56.50060044 | 212.718325 | 141.7785894 | S100a3 |
| 2.45654382 | 1.11E-09 | 1.61E-08 | 12.18002816 | 13.675051 | 17.87441788 | 55.58930043 | 74.87685039 | 110.423709 | C030034L19Rik |
| 2.259459402 | 1.20E-09 | 1.73E-08 | 26.23390681 | 17.32173127 | 17.87441788 | 63.79100049 | 120.8240086 | 110.423709 | Gm26868 |
| 7.72050765 | 1.21E-09 | 1.74E-08 | 0 | 0 | 0 | 27.33900021 | 41.69279169 | 43.62418134 | Gm29536 |
| 1.174810505 | 1.25E-09 | 1.79E-08 | 1145.859573 | 1724.879767 | 1046.179164 | 3087.484424 | 2613.031904 | 3146.394079 | NA |
| 1.075414929 | 1.30E-09 | 1.85E-08 | 293.2576012 | 233.3875371 | 331.2024489 | 522.174904 | 634.7514817 | 650.2729532 | Chrm4 |
| 2.338393523 | 1.32E-09 | 1.89E-08 | 20.61235535 | 9.116700669 | 15.77154519 | 66.52490051 | 71.47335719 | 92.70138536 | Slamf1 |
| 1.233024321 | 1.49E-09 | 2.11E-08 | 213.6189555 | 173.2173127 | 205.0300874 | 366.3426028 | 573.4886041 | 449.8743701 | Nog |
| 2.814109933 | 1.50E-09 | 2.12E-08 | 14.05387865 | 10.02837074 | 10.51436346 | 47.38760037 | 71.47335719 | 126.782777 | 4930417O13Rik |
| 1.021091386 | 1.55E-09 | 2.20E-08 | 4384.810139 | 3153.466761 | 4631.577104 | 7122.720855 | 9023.511345 | 8550.339543 | Hpcal1 |
| 1.151290432 | 1.58E-09 | 2.23E-08 | 179.8896467 | 130.3688196 | 179.7956151 | 368.1652028 | 321.6301074 | 399.4339104 | Lhfpl1 |
| 1.002442077 | 1.67E-09 | 2.35E-08 | 1723.005523 | 1344.713349 | 2002.986239 | 3239.671525 | 3109.091038 | 3810.299589 | Rtn2 |
| 1.243511664 | 1.70E-09 | 2.39E-08 | 1091.517909 | 1719.409746 | 1051.436346 | 3085.661824 | 2561.979506 | 3502.203809 | Ints6l |
| 1.393226909 | 1.84E-09 | 2.57E-08 | 103.998702 | 139.4855202 | 132.4809796 | 242.4058019 | 350.5597996 | 396.7073991 | Sfmbt2 |
| 1.081351732 | 1.89E-09 | 2.62E-08 | 3131.204163 | 4425.246505 | 2869.369788 | 8131.529963 | 6390.058482 | 7542.893606 | Serpini1 |
| 1.28491558 | 2.16E-09 | 2.97E-08 | 81.51249617 | 86.60865635 | 113.5551254 | 196.8408015 | 240.7971439 | 248.1125314 | Hmga1b |
| 2.849264254 | 2.84E-09 | 3.86E-08 | 9.369252434 | 6.381690468 | 4.205745383 | 44.65370034 | 47.64890479 | 53.16697101 | Gm44171 |
| 1.624112552 | 2.85E-09 | 3.86E-08 | 68.39554277 | 72.93360535 | 49.41750826 | 180.4374014 | 153.157194 | 259.0185767 | Gm42608 |
| 1.845454557 | 2.85E-09 | 3.86E-08 | 34.666234 | 43.76016321 | 29.44021768 | 123.936801 | 96.99955619 | 170.4069584 | 4930447F24Rik |
| 2.697992864 | 2.87E-09 | 3.89E-08 | 12.18002816 | 11.85171087 | 9.462927113 | 45.56500035 | 106.3591625 | 65.43627202 | Egfros |
| 3.053777123 | 2.97E-09 | 4.01E-08 | 13.11695341 | 7.293360535 | 10.51436346 | 35.54070027 | 104.6574159 | 117.2399874 | Gm34425 |
| 2.030190519 | 3.01E-09 | 4.06E-08 | 28.1077573 | 28.26177207 | 18.92585423 | 114.8238009 | 71.47335719 | 124.0562657 | Gm44898 |
| 1.951921348 | 3.21E-09 | 4.30E-08 | 40.28778546 | 35.55513261 | 19.97729057 | 92.95260072 | 131.0344882 | 149.9581234 | Nectin4 |
| 2.290301505 | 3.29E-09 | 4.41E-08 | 56.2155146 | 22.79175167 | 38.9031448 | 215.9781017 | 107.2100358 | 254.9288097 | Gbp8 |
| 1.010098769 | 3.68E-09 | 4.88E-08 | 3829.21347 | 2708.571769 | 3849.308462 | 6591.432951 | 6220.734695 | 8110.007963 | Fras1 |
| 1.387758699 | 3.69E-09 | 4.89E-08 | 111.494104 | 70.19859515 | 91.47496209 | 206.8651016 | 226.3322978 | 283.5571787 | 4932441J04Rik |
| 1.124372125 | 4.05E-09 | 5.35E-08 | 406.6255556 | 377.4314077 | 396.3915024 | 865.7350067 | 644.9619613 | 1067.429187 | Mylk |
| 2.814554884 | 4.23E-09 | 5.54E-08 | 9.369252434 | 10.9400408 | 12.61723615 | 55.58930043 | 51.05239799 | 126.782777 | Dbhos |
| 1.093556041 | 4.47E-09 | 5.83E-08 | 4507.547346 | 3073.239795 | 4604.239759 | 7069.865455 | 9747.604523 | 9184.253429 | Cdh13 |
| 1.965856156 | 4.88E-09 | 6.31E-08 | 37.47700973 | 17.32173127 | 28.38878134 | 91.1300007 | 103.8065426 | 130.872544 | Pebp4 |
| 1.263248023 | 5.22E-09 | 6.72E-08 | 205.1866283 | 201.4790848 | 200.8243421 | 482.9890037 | 353.9632928 | 625.7343512 | Plb1 |
| 1.215314991 | 5.66E-09 | 7.26E-08 | 175.2050205 | 172.3056426 | 169.2812517 | 293.4386023 | 434.7962562 | 474.4129721 | Slc17a6 |
| 1.46886063 | 5.88E-09 | 7.52E-08 | 143.3495622 | 134.0154998 | 107.2465073 | 237.8493018 | 437.3488761 | 391.2543764 | Plekhn1 |
| 1.295162873 | 6.28E-09 | 8.00E-08 | 57.15243985 | 61.99356455 | 57.82899902 | 132.138501 | 154.0080673 | 148.5948677 | Fam186b |
| 1.372240818 | 6.52E-09 | 8.29E-08 | 2075.289414 | 3033.126312 | 1946.208676 | 6574.118251 | 4098.656686 | 7594.697321 | Luzp2 |
| 1.450979025 | 6.91E-09 | 8.75E-08 | 84.3232719 | 65.64024481 | 89.3720894 | 161.3001012 | 232.2884109 | 261.7450881 | Ccdc155 |
| 1.068470544 | 7.58E-09 | 9.52E-08 | 894.7636074 | 743.9227746 | 931.5726024 | 1460.813911 | 2272.682584 | 1653.629124 | Col6a1 |
| 1.075510974 | 8.54E-09 | 1.06E-07 | 259.5282924 | 257.0909589 | 241.8303596 | 407.3511031 | 587.1025769 | 606.6487718 | Pnpla3 |
| 1.046036943 | 8.81E-09 | 1.09E-07 | 14378.05478 | 9498.690427 | 14464.60981 | 22545.56217 | 29166.23497 | 27455.96913 | Sez6l2 |
| 1.584597865 | 9.23E-09 | 1.14E-07 | 47.78318741 | 53.78853394 | 77.80628959 | 135.783701 | 190.5956192 | 211.3046284 | Nnmt |
| 1.848084697 | 9.78E-09 | 1.20E-07 | 69.33246801 | 30.08511221 | 48.36607191 | 171.3244013 | 138.6923479 | 223.5739294 | Resp18 |
| 2.451006631 | 1.02E-08 | 1.25E-07 | 12.18002816 | 14.58672107 | 14.72010884 | 43.74240034 | 98.70130279 | 84.52185135 | Cacna1s |
| 2.783928603 | 1.02E-08 | 1.25E-07 | 9.369252434 | 5.470020401 | 10.51436346 | 38.2746003 | 56.15763779 | 80.43208435 | Sprr1a |
| 2.438100346 | 1.02E-08 | 1.25E-07 | 11.24310292 | 20.05674147 | 10.51436346 | 57.41190044 | 64.66637079 | 107.6971977 | Yipf7 |
| 1.254424183 | 1.05E-08 | 1.28E-07 | 1473.783408 | 2517.121055 | 1450.982157 | 4763.365137 | 3613.658905 | 4609.16741 | Slitrk4 |
| 1.726903303 | 1.12E-08 | 1.36E-07 | 71.2063185 | 34.64346254 | 50.4689446 | 134.872401 | 175.2798998 | 208.5781171 | Krt12 |
| 1.547682188 | 1.16E-08 | 1.40E-07 | 82.44942142 | 82.96197608 | 78.85772594 | 213.2442016 | 171.0255333 | 333.9976384 | Gm45869 |
| 1.674292852 | 1.27E-08 | 1.52E-07 | 39.35086022 | 29.17344214 | 35.74883576 | 86.57350067 | 130.1836149 | 115.8767317 | Foxf2 |
| 1.052681385 | 1.33E-08 | 1.58E-07 | 1985.344591 | 1327.391617 | 2007.191984 | 3193.195225 | 4254.366499 | 3585.362404 | Necab3 |
| 5.640081634 | 1.42E-08 | 1.69E-07 | 2.81077573 | 0 | 0 | 24.60510019 | 72.32423049 | 46.35069268 | Sp7 |
| 1.068867714 | 1.42E-08 | 1.69E-07 | 12780.59724 | 7855.860966 | 12793.87746 | 22226.60717 | 22193.32828 | 25709.63862 | Pcdh8 |
| 1.464878369 | 1.43E-08 | 1.70E-07 | 2851.063516 | 5330.534881 | 2706.397154 | 11432.25859 | 7428.974781 | 11197.78205 | Fam126b |
| 2.693139283 | 1.44E-08 | 1.71E-07 | 8.43232719 | 6.381690468 | 8.411490767 | 40.09720031 | 44.24541159 | 66.79952768 | Gm4889 |
| 1.468758113 | 1.45E-08 | 1.71E-07 | 90.88174861 | 53.78853394 | 76.75485325 | 180.4374014 | 182.0868862 | 252.2022984 | Mir132 |
| 1.056778514 | 1.47E-08 | 1.73E-07 | 2074.352489 | 1370.24011 | 2070.278165 | 3203.219525 | 4219.480694 | 4048.869331 | Rims4 |
| 2.601871359 | 1.68E-08 | 1.97E-07 | 9.369252434 | 25.52676187 | 18.92585423 | 57.41190044 | 108.9117824 | 162.2274244 | Mmp10 |
| 5.655621165 | 1.72E-08 | 2.00E-07 | 0 | 1.823340134 | 1.051436346 | 19.13730015 | 70.62248389 | 55.89348235 | Vtcn1 |
| 1.568293366 | 1.76E-08 | 2.04E-07 | 60.90014082 | 54.70020401 | 43.10889018 | 117.5577009 | 157.4115605 | 199.0353274 | Mir5125 |
| 1.223724325 | 1.95E-08 | 2.25E-07 | 789.8279802 | 1124.089192 | 691.8451156 | 1845.382514 | 1671.115161 | 2575.189955 | 9330162G02Rik |
| 1.037695958 | 1.99E-08 | 2.28E-07 | 298.8791526 | 262.5609793 | 303.865104 | 537.6670042 | 502.8661202 | 740.2478272 | Mettl15 |
| 1.018420907 | 2.11E-08 | 2.41E-07 | 352.2838915 | 266.2076595 | 370.1055937 | 592.3450046 | 629.6462419 | 782.5087529 | Gpr176 |
| 1.845821382 | 2.17E-08 | 2.47E-07 | 31.85545827 | 20.96841154 | 25.2344723 | 76.54920059 | 85.93820329 | 119.9664987 | Gm13630 |
| 1.811297408 | 2.35E-08 | 2.66E-07 | 50.59396314 | 20.05674147 | 47.31463556 | 134.872401 | 141.2449678 | 136.3255667 | Htr5a |
| 2.244207639 | 2.37E-08 | 2.68E-07 | 15.92772914 | 16.4100612 | 17.87441788 | 51.9441004 | 111.4644023 | 73.61580602 | Liph |
| 2.054432194 | 2.46E-08 | 2.76E-07 | 26.23390681 | 20.05674147 | 29.44021768 | 72.90400056 | 149.7537008 | 89.97487402 | Sult5a1 |
| 2.614372159 | 2.74E-08 | 3.06E-07 | 39.35086022 | 29.17344214 | 27.33734499 | 72.90400056 | 348.858053 | 164.9539357 | Sytl1 |
| 1.698651706 | 2.79E-08 | 3.10E-07 | 118.0525807 | 48.31851354 | 88.32065305 | 215.0668017 | 285.0425555 | 327.1813601 | Cd164l2 |
| 3.653417336 | 3.11E-08 | 3.42E-07 | 8.43232719 | 0.911670067 | 1.051436346 | 41.91980032 | 39.14017179 | 51.80371535 | Gzmc |
| 1.010587837 | 3.24E-08 | 3.56E-07 | 5806.125733 | 8418.361397 | 5426.462981 | 14835.96411 | 11548.05243 | 13209.94741 | Lin7a |
| 1.3846765 | 3.28E-08 | 3.60E-07 | 86.19712239 | 108.488738 | 91.47496209 | 177.7035014 | 312.2705011 | 257.6553211 | Syt6 |
| 1.355936976 | 3.32E-08 | 3.64E-07 | 2223.323603 | 3991.291553 | 1949.362985 | 7385.175257 | 5758.710494 | 7756.924745 | Ncam2 |
| 1.860825193 | 3.47E-08 | 3.78E-07 | 60.90014082 | 22.79175167 | 43.10889018 | 131.227201 | 144.648461 | 185.4027707 | Iqcf5 |
| 1.17120835 | 3.85E-08 | 4.18E-07 | 249.2221147 | 238.8575575 | 201.8757784 | 569.5625044 | 373.5333786 | 614.8283058 | Tnfaip8l3 |
| 2.062174307 | 3.95E-08 | 4.28E-07 | 18.73850487 | 16.4100612 | 26.28590865 | 57.41190044 | 90.19256979 | 109.0604534 | Pabpc1l |
| 1.900493737 | 4.08E-08 | 4.41E-07 | 46.84626217 | 24.61509181 | 19.97729057 | 101.1543008 | 106.3591625 | 136.3255667 | Gm31373 |
| 1.179959436 | 4.28E-08 | 4.60E-07 | 2854.811217 | 4818.176303 | 2672.751191 | 7695.928559 | 7033.318697 | 8715.293479 | Myo9a |
| 3.00691164 | 4.39E-08 | 4.70E-07 | 5.62155146 | 20.96841154 | 6.308618075 | 42.83110033 | 116.5696421 | 107.6971977 | Mei1 |
| 1.999064941 | 4.42E-08 | 4.72E-07 | 21.5492806 | 14.58672107 | 29.44021768 | 86.57350067 | 66.36811739 | 109.0604534 | Magel2 |
| 1.005598363 | 4.43E-08 | 4.73E-07 | 249.2221147 | 185.0690236 | 251.2932867 | 439.2466034 | 523.2870794 | 410.3399558 | Xkr7 |
| 1.06210367 | 4.45E-08 | 4.75E-07 | 148.9711137 | 130.3688196 | 130.3781069 | 318.0437025 | 233.9901575 | 305.3692694 | Otogl |
| 1.450131828 | 5.15E-08 | 5.43E-07 | 69.33246801 | 62.90523461 | 52.57181729 | 126.670701 | 167.6220401 | 214.0311397 | Erich6 |
| 1.387713275 | 5.28E-08 | 5.55E-07 | 250.15904 | 156.8072515 | 251.2932867 | 408.2624032 | 738.5580243 | 573.9306358 | Gls2 |
| 1.203960627 | 5.54E-08 | 5.81E-07 | 146.160338 | 198.7440746 | 125.1209252 | 313.4872024 | 340.34932 | 433.5153021 | A230103L15Rik |
| 1.66829247 | 5.93E-08 | 6.18E-07 | 41.22471071 | 29.17344214 | 30.49165403 | 87.48480068 | 134.4379814 | 98.15440802 | Cd1d1 |
| 1.73425585 | 6.34E-08 | 6.59E-07 | 74.01709423 | 89.34366655 | 55.72612633 | 163.1227013 | 359.9194059 | 205.8516057 | Lingo4 |
| 1.498937817 | 6.80E-08 | 7.00E-07 | 41.22471071 | 73.84527542 | 52.57181729 | 128.493301 | 173.5781532 | 173.1334697 | Itga2 |
| 2.106258051 | 7.93E-08 | 8.05E-07 | 17.80157962 | 20.05674147 | 28.38878134 | 55.58930043 | 122.5257552 | 106.333942 | Fcer2a |
| 1.665071829 | 8.20E-08 | 8.30E-07 | 28.1077573 | 20.05674147 | 32.59452672 | 83.83960065 | 79.13121689 | 92.70138536 | D830036C21Rik |
| 1.086415373 | 8.82E-08 | 8.89E-07 | 107.746403 | 137.6621801 | 131.4295432 | 222.3572017 | 303.7617681 | 274.0143891 | Nemp2 |
| 1.504821958 | 9.37E-08 | 9.41E-07 | 106.8094777 | 71.11026522 | 83.06347132 | 160.3888012 | 285.8934288 | 295.8264797 | Hhatl |
| 1.705861359 | 1.10E-07 | 1.09E-06 | 177.078871 | 165.9239522 | 145.0982157 | 683.4750053 | 250.1567502 | 661.1789985 | Hcrtr2 |
| 1.292316202 | 1.20E-07 | 1.18E-06 | 359.7792935 | 186.8923637 | 378.5170845 | 741.7982057 | 682.4003865 | 841.1287465 | Nxph3 |
| 1.442568844 | 1.28E-07 | 1.26E-06 | 49.6570379 | 49.23018361 | 56.77756268 | 111.1786009 | 130.1836149 | 184.039515 | Sgpp2 |
| 1.68036703 | 1.30E-07 | 1.28E-06 | 26.23390681 | 28.26177207 | 22.08016326 | 72.90400056 | 74.02597709 | 100.8809194 | Tdrd12 |
| 1.507111362 | 1.34E-07 | 1.32E-06 | 51.53088839 | 99.37203729 | 67.29192614 | 227.8250018 | 157.4115605 | 237.2064861 | A330093E20Rik |
| 1.142408314 | 1.42E-07 | 1.39E-06 | 154.5926652 | 207.8607752 | 134.5838523 | 313.4872024 | 350.5597996 | 437.6050691 | Nsun7 |
| 1.67170403 | 1.45E-07 | 1.41E-06 | 94.62944958 | 273.5010201 | 139.841034 | 480.2551037 | 512.2257265 | 628.4608625 | Plcxd3 |
| 1.699408631 | 1.61E-07 | 1.56E-06 | 27.17083206 | 25.52676187 | 39.95458114 | 78.3718006 | 96.14868289 | 126.782777 | Gm13713 |
| 1.605767065 | 1.68E-07 | 1.62E-06 | 193.0066001 | 102.1070475 | 247.0875413 | 492.1020038 | 419.4805368 | 738.8845715 | Mir212 |
| 1.564079749 | 1.72E-07 | 1.65E-06 | 40.28778546 | 30.08511221 | 29.44021768 | 79.28310061 | 102.9556693 | 114.513476 | Itgb7 |
| 1.197701973 | 1.77E-07 | 1.69E-06 | 170.5203943 | 107.5770679 | 177.6927425 | 289.7934022 | 393.1034645 | 361.2627518 | 1500035N22Rik |
| 1.283848226 | 1.89E-07 | 1.79E-06 | 176.1419458 | 299.939452 | 171.3841244 | 552.2478043 | 418.6296635 | 609.3752832 | Cep85l |
| 1.60625916 | 2.07E-07 | 1.95E-06 | 28.1077573 | 44.67183328 | 33.64596307 | 89.30740069 | 99.55217609 | 137.6888224 | C130023A14Rik |
| 3.40704861 | 2.08E-07 | 1.95E-06 | 6.558476704 | 2.735010201 | 2.102872692 | 33.71810026 | 25.526199 | 64.07301635 | Cartpt |
| 2.995134895 | 2.08E-07 | 1.96E-06 | 3.747700973 | 9.116700669 | 7.360054421 | 24.60510019 | 61.26287759 | 76.34231735 | Wincr1 |
| 1.101359056 | 2.10E-07 | 1.97E-06 | 196.7543011 | 133.1038298 | 209.2358328 | 328.0680025 | 396.5069577 | 432.1520464 | Gpr153 |
| 1.009844076 | 2.52E-07 | 2.32E-06 | 81.51249617 | 92.07867675 | 84.11490767 | 170.4131013 | 174.4290265 | 174.4967254 | Gm12709 |
| 1.363788565 | 2.53E-07 | 2.33E-06 | 65.58476704 | 97.54869715 | 88.32065305 | 154.0097012 | 257.8146099 | 235.8432304 | Ctnna3 |
| 1.228459471 | 2.53E-07 | 2.33E-06 | 51.53088839 | 52.87686388 | 57.82899902 | 113.9125009 | 124.2275018 | 143.141845 | Crb1 |
| 1.091911694 | 2.60E-07 | 2.38E-06 | 153.6557399 | 137.6621801 | 170.332688 | 296.1725023 | 273.9812026 | 417.1562341 | Cpm |
| 1.484161784 | 2.80E-07 | 2.56E-06 | 58.08936509 | 47.40684348 | 85.16634402 | 158.5662012 | 146.3502076 | 229.0269521 | Stab2 |
| 1.152327597 | 3.63E-07 | 3.25E-06 | 845.1065695 | 1438.615366 | 815.9146044 | 2348.420118 | 1896.596585 | 2648.805761 | Ythdc2 |
| 1.109430202 | 3.88E-07 | 3.45E-06 | 4905.740574 | 3042.243013 | 4817.681337 | 9034.62827 | 7069.055375 | 11441.80481 | Trnp1 |
| 1.08381296 | 3.93E-07 | 3.49E-06 | 1409.135566 | 1944.592253 | 1344.787086 | 3587.788128 | 2372.23476 | 4003.881894 | Scn1a |
| 5.057379285 | 3.96E-07 | 3.51E-06 | 0.936925243 | 0 | 2.102872692 | 19.13730015 | 47.64890479 | 29.99162467 | Gm31406 |
| 1.020894344 | 4.16E-07 | 3.67E-06 | 505.0027062 | 614.4656251 | 477.352101 | 974.1797075 | 865.338146 | 1406.879848 | Rorb |
| 2.531548314 | 4.19E-07 | 3.70E-06 | 6.558476704 | 10.02837074 | 4.205745383 | 35.54070027 | 45.09628489 | 40.89767001 | 5830473C10Rik |
| 2.073130327 | 4.24E-07 | 3.73E-06 | 12.18002816 | 10.02837074 | 25.2344723 | 57.41190044 | 73.17510379 | 66.79952768 | Cavin4 |
| 1.769082712 | 4.46E-07 | 3.91E-06 | 82.44942142 | 51.96519381 | 78.85772594 | 132.138501 | 359.0685326 | 234.4799747 | Notch4 |
| 2.997128092 | 4.48E-07 | 3.92E-06 | 6.558476704 | 7.293360535 | 4.205745383 | 20.95990016 | 67.21899069 | 57.25673801 | 3300005D01Rik |
| 4.135380761 | 4.53E-07 | 3.96E-06 | 6.558476704 | 3.646680267 | 1.051436346 | 13.66950011 | 139.5432212 | 46.35069268 | Gjb4 |
| 1.393541025 | 4.58E-07 | 4.01E-06 | 44.03548644 | 41.93682308 | 42.05745383 | 87.48480068 | 115.7187688 | 134.962311 | Tdrd6 |
| 1.678593673 | 4.83E-07 | 4.20E-06 | 27.17083206 | 21.8800816 | 23.13159961 | 59.23450046 | 81.68383679 | 91.33812969 | Gm7972 |
| 1.060022387 | 5.05E-07 | 4.37E-06 | 123.6741321 | 115.7820985 | 101.9893255 | 223.2685017 | 202.5078454 | 290.3734571 | Prdm9 |
| 1.222703962 | 5.69E-07 | 4.88E-06 | 2253.30521 | 4293.966015 | 2155.444509 | 7017.010054 | 5788.491059 | 7508.812214 | Far1 |
| 1.798958702 | 5.75E-07 | 4.92E-06 | 29.98160779 | 24.61509181 | 22.08016326 | 77.4605006 | 65.51724409 | 126.782777 | Gm23600 |
| 4.702399149 | 6.99E-07 | 5.88E-06 | 2.81077573 | 0.911670067 | 0 | 19.13730015 | 22.9735791 | 58.61999368 | Olfr683 |
| 1.041546111 | 7.07E-07 | 5.93E-06 | 1697.708541 | 2631.991483 | 1494.091047 | 4215.673833 | 3393.28272 | 4382.866969 | Lmbrd2 |
| 1.630593369 | 7.48E-07 | 6.26E-06 | 57.15243985 | 27.35010201 | 44.16032653 | 104.7995008 | 171.0255333 | 121.3297544 | Muc3a |
| 1.730815314 | 7.50E-07 | 6.27E-06 | 45.90933693 | 62.90523461 | 36.80027211 | 103.8882008 | 236.5427774 | 143.141845 | Ovgp1 |
| 1.273537729 | 7.70E-07 | 6.42E-06 | 110.5571787 | 131.2804896 | 105.1436346 | 186.8165014 | 353.1124195 | 299.9162467 | Lat2 |
| 4.110515015 | 7.91E-07 | 6.58E-06 | 2.81077573 | 1.823340134 | 1.051436346 | 12.7582001 | 43.39453829 | 43.62418134 | Nts |
| 1.009669979 | 8.34E-07 | 6.92E-06 | 198.6281516 | 274.4126901 | 234.4703051 | 371.8104029 | 490.1030207 | 565.7511018 | Tnfrsf23 |
| 1.539231583 | 8.38E-07 | 6.94E-06 | 50.59396314 | 69.28692508 | 33.64596307 | 111.1786009 | 160.8150537 | 177.2232367 | Col10a1 |
| 1.179898903 | 8.39E-07 | 6.95E-06 | 63.71091655 | 87.52032642 | 91.47496209 | 179.5261014 | 154.0080673 | 218.1209067 | Gm12404 |
| 2.038425348 | 8.47E-07 | 7.00E-06 | 31.85545827 | 10.02837074 | 26.28590865 | 66.52490051 | 110.613529 | 102.244175 | Gm44275 |
| 1.347672352 | 8.85E-07 | 7.28E-06 | 665.2169228 | 1414.000274 | 621.3988804 | 2317.435918 | 2016.569721 | 2542.471819 | Rgs17 |
| 1.383019914 | 9.90E-07 | 8.07E-06 | 41.22471071 | 50.14185368 | 31.54309038 | 104.7995008 | 94.44693629 | 124.0562657 | Gm26767 |
| 1.967244872 | 1.00E-06 | 8.16E-06 | 12.18002816 | 12.76338094 | 14.72010884 | 41.00850032 | 54.45589119 | 59.98324935 | Lmntd1 |
| 1.798756202 | 1.01E-06 | 8.22E-06 | 38.41393498 | 64.72857475 | 29.44021768 | 108.4447008 | 131.8853615 | 224.9371851 | Gm47135 |
| 1.704371709 | 1.03E-06 | 8.35E-06 | 40.28778546 | 72.02193528 | 33.64596307 | 134.872401 | 122.5257552 | 222.2106737 | A230006K03Rik |
| 1.286680756 | 1.04E-06 | 8.42E-06 | 183.6373477 | 94.81368695 | 173.4869971 | 308.0194024 | 447.5593557 | 344.9036838 | Cacng6 |
| 1.336239047 | 1.04E-06 | 8.42E-06 | 40.28778546 | 32.82012241 | 47.31463556 | 96.59780075 | 94.44693629 | 113.1502204 | Gpr6 |
| 1.513350799 | 1.07E-06 | 8.68E-06 | 83.38634666 | 141.3088604 | 71.49767152 | 246.0510019 | 212.718325 | 391.2543764 | Lancl3 |
| 1.422086165 | 1.08E-06 | 8.71E-06 | 46.84626217 | 31.90845234 | 45.21176287 | 86.57350067 | 126.7801217 | 118.603243 | Mustn1 |
| 1.915287882 | 1.09E-06 | 8.82E-06 | 17.80157962 | 19.1450714 | 24.18303596 | 49.21020038 | 74.87685039 | 107.6971977 | Cacna1f |
| 1.246183027 | 1.12E-06 | 9.02E-06 | 75.89094471 | 54.70020401 | 55.72612633 | 118.4690009 | 162.5168003 | 162.2274244 | A630023P12Rik |
| 1.686064769 | 1.23E-06 | 9.82E-06 | 26.23390681 | 72.02193528 | 39.95458114 | 140.3402011 | 129.3327416 | 177.2232367 | Zfp804b |
| 1.706304426 | 1.26E-06 | 1.00E-05 | 28.1077573 | 31.90845234 | 13.6686725 | 81.10570063 | 73.17510379 | 88.61161836 | Samsn1 |
| 1.545980759 | 1.33E-06 | 1.05E-05 | 25.29698157 | 26.43843194 | 27.33734499 | 64.7023005 | 72.32423049 | 95.42789669 | Ppef1 |
| 2.586823024 | 1.38E-06 | 1.09E-05 | 7.495401947 | 9.116700669 | 9.462927113 | 55.58930043 | 24.6753257 | 77.70557302 | A530021J07Rik |
| 1.480302525 | 1.41E-06 | 1.11E-05 | 27.17083206 | 30.99678227 | 24.18303596 | 69.25880053 | 74.87685039 | 87.24836269 | Gm27000 |
| 1.880785758 | 1.47E-06 | 1.16E-05 | 19.67543011 | 30.08511221 | 17.87441788 | 61.96840048 | 68.92073729 | 121.3297544 | Gm45201 |
| 1.714255378 | 1.50E-06 | 1.17E-05 | 16.86465438 | 20.96841154 | 19.97729057 | 52.85540041 | 60.41200429 | 77.70557302 | Gm8407 |
| 2.308530317 | 1.62E-06 | 1.26E-05 | 14.05387865 | 11.85171087 | 4.205745383 | 39.1859003 | 48.49977809 | 64.07301635 | Cxcr6 |
| 1.162509531 | 1.76E-06 | 1.36E-05 | 119.9264312 | 81.13863595 | 154.5611428 | 250.6075019 | 250.1567502 | 294.4632241 | Sdcbp2 |
| 1.528302309 | 1.76E-06 | 1.36E-05 | 25.29698157 | 24.61509181 | 36.80027211 | 97.50910075 | 80.83296349 | 69.52603902 | Wnt10a |
| 1.096171803 | 1.81E-06 | 1.40E-05 | 100.251001 | 100.2837074 | 89.3720894 | 171.3244013 | 188.0429993 | 264.4715994 | D030047H15Rik |
| 2.790902217 | 1.86E-06 | 1.43E-05 | 4.684626217 | 7.293360535 | 3.154309038 | 22.78250018 | 39.99104509 | 43.62418134 | Gm45163 |
| 2.976864604 | 1.89E-06 | 1.45E-05 | 4.684626217 | 3.646680267 | 4.205745383 | 19.13730015 | 36.58755189 | 43.62418134 | Lyzl4os |
| 4.910437692 | 2.03E-06 | 1.55E-05 | 0.936925243 | 2.735010201 | 0 | 8.201700063 | 68.92073729 | 36.80790301 | Cym |
| 1.507643548 | 2.05E-06 | 1.56E-05 | 231.4205351 | 557.0304109 | 221.853069 | 903.098307 | 788.759549 | 1184.669175 | Zfp948 |
| 2.382255441 | 2.24E-06 | 1.69E-05 | 9.369252434 | 6.381690468 | 6.308618075 | 28.25030022 | 42.54366499 | 44.98743701 | Ptgs2os |
| 2.519560429 | 2.31E-06 | 1.74E-05 | 8.43232719 | 8.205030602 | 11.5657998 | 23.69380018 | 67.21899069 | 70.88929468 | Loxhd1 |
| 1.246239088 | 2.40E-06 | 1.81E-05 | 249.2221147 | 394.753139 | 256.5504684 | 737.2417057 | 472.2346814 | 931.1036206 | 2900052N01Rik |
| 1.41937023 | 2.49E-06 | 1.87E-05 | 118.0525807 | 125.8104692 | 83.06347132 | 330.8019026 | 171.0255333 | 376.2585641 | Htr2a |
| 1.978661018 | 2.74E-06 | 2.04E-05 | 23.42313108 | 11.85171087 | 11.5657998 | 44.65370034 | 68.92073729 | 72.25255035 | Cpa2 |
| 1.010238991 | 2.79E-06 | 2.07E-05 | 159.2772914 | 230.6525269 | 157.7154519 | 408.2624032 | 299.5074016 | 398.0706548 | Epha3 |
| 1.578536604 | 3.21E-06 | 2.36E-05 | 37.47700973 | 24.61509181 | 37.85170845 | 67.43620052 | 121.6748819 | 109.0604534 | Barx2 |
| 1.550914364 | 3.28E-06 | 2.41E-05 | 20.61235535 | 21.8800816 | 32.59452672 | 64.7023005 | 70.62248389 | 84.52185135 | Gm4926 |
| 1.010151256 | 3.34E-06 | 2.45E-05 | 343.8515643 | 350.0813057 | 292.2993042 | 480.2551037 | 644.111088 | 867.0306042 | Sh3bp2 |
| 1.191313408 | 3.35E-06 | 2.45E-05 | 105.8725525 | 96.63702709 | 115.657998 | 166.7679013 | 312.2705011 | 246.7492757 | Lamc2 |
| 1.137167531 | 3.37E-06 | 2.46E-05 | 62.77399131 | 75.66861555 | 52.57181729 | 121.2029009 | 155.7098139 | 144.5051007 | Glp2r |
| 1.885015286 | 3.43E-06 | 2.51E-05 | 12.18002816 | 25.52676187 | 21.02872692 | 50.12150039 | 71.47335719 | 96.79115236 | Gm28809 |
| 2.004368927 | 3.44E-06 | 2.51E-05 | 18.73850487 | 12.76338094 | 17.87441788 | 41.91980032 | 59.56113099 | 98.15440802 | Enpp3 |
| 1.165177018 | 3.49E-06 | 2.54E-05 | 1483.15266 | 2809.767146 | 1420.490503 | 4573.814735 | 3422.212412 | 4820.472039 | Fut9 |
| 1.61585843 | 3.50E-06 | 2.55E-05 | 22.48620584 | 20.05674147 | 23.13159961 | 55.58930043 | 61.26287759 | 85.88510702 | Gm16796 |
| 1.057798275 | 3.54E-06 | 2.57E-05 | 167.7096186 | 121.2521189 | 174.5384334 | 244.2284019 | 372.6825053 | 347.6301951 | Lemd1 |
| 2.759101699 | 3.61E-06 | 2.61E-05 | 7.495401947 | 5.470020401 | 5.257181729 | 19.13730015 | 59.56113099 | 44.98743701 | Spink2 |
| 1.460409002 | 3.67E-06 | 2.66E-05 | 32.79238352 | 29.17344214 | 29.44021768 | 62.87970049 | 97.85042949 | 91.33812969 | Prss57 |
| 1.677861519 | 3.68E-06 | 2.66E-05 | 56.2155146 | 47.40684348 | 51.52038095 | 88.39610068 | 251.8584968 | 155.411146 | Hkdc1 |
| 1.632166294 | 3.70E-06 | 2.67E-05 | 24.36005633 | 25.52676187 | 22.08016326 | 56.50060044 | 68.06986399 | 100.8809194 | 4933428G20Rik |
| 1.21219036 | 3.76E-06 | 2.71E-05 | 171.4573195 | 246.1509181 | 179.7956151 | 306.1968024 | 613.4796492 | 464.8701824 | Qpct |
| 1.64443133 | 3.81E-06 | 2.75E-05 | 41.22471071 | 40.11348294 | 32.59452672 | 114.8238009 | 71.47335719 | 173.1334697 | Gm16062 |
| 2.142542664 | 3.82E-06 | 2.75E-05 | 14.99080389 | 13.675051 | 11.5657998 | 32.80680025 | 59.56113099 | 87.24836269 | Btn2a2 |
| 4.367792131 | 3.91E-06 | 2.81E-05 | 3.747700973 | 0.911670067 | 0 | 10.02430008 | 47.64890479 | 40.89767001 | 5730419F03Rik |
| 1.110827883 | 4.05E-06 | 2.90E-05 | 2261.737537 | 1518.842331 | 2195.39909 | 2880.619322 | 5768.0701 | 4256.084192 | Cabp7 |
| 1.079092218 | 4.13E-06 | 2.95E-05 | 2762.055617 | 4860.113126 | 2429.869395 | 7506.378158 | 6184.147144 | 7549.709884 | Cacnb4 |
| 1.66889898 | 4.24E-06 | 3.03E-05 | 26.23390681 | 55.61187408 | 23.13159961 | 101.1543008 | 97.85042949 | 137.6888224 | Ddx4 |
| 4.31410085 | 4.38E-06 | 3.11E-05 | 1.873850487 | 1.823340134 | 0 | 17.31470013 | 18.7192126 | 40.89767001 | Gm37269 |
| 3.626841713 | 4.44E-06 | 3.15E-05 | 1.873850487 | 3.646680267 | 1.051436346 | 20.04860015 | 18.7192126 | 44.98743701 | Ros1 |
| 1.040905078 | 4.69E-06 | 3.31E-05 | 182.7004225 | 123.075459 | 176.6413061 | 255.164002 | 373.5333786 | 363.9892631 | Rasd2 |
| 2.147571628 | 5.01E-06 | 3.50E-05 | 12.18002816 | 24.61509181 | 23.13159961 | 51.03280039 | 146.3502076 | 66.79952768 | Nhlh1 |
| 1.795629821 | 5.10E-06 | 3.56E-05 | 10.30617768 | 18.23340134 | 16.82298153 | 46.47630036 | 52.75414459 | 58.61999368 | Gm15704 |
| 1.348152062 | 5.31E-06 | 3.70E-05 | 70.26939325 | 148.6022209 | 70.44623517 | 248.7849019 | 220.3761847 | 269.9246221 | A230057D06Rik |
| 2.73536542 | 5.32E-06 | 3.70E-05 | 3.747700973 | 5.470020401 | 4.205745383 | 30.98420024 | 21.2718325 | 38.17115868 | Fscn3 |
| 1.072808292 | 5.37E-06 | 3.73E-05 | 59.96321558 | 61.99356455 | 69.39479883 | 113.9125009 | 136.9906013 | 152.6846347 | Hspa12b |
| 1.423101903 | 5.41E-06 | 3.76E-05 | 29.04468254 | 49.23018361 | 38.9031448 | 79.28310061 | 112.3152756 | 124.0562657 | Tmem88b |
| 1.299792646 | 5.54E-06 | 3.84E-05 | 231.4205351 | 478.6267851 | 220.8016326 | 821.0813063 | 597.3130565 | 876.5733939 | Zfp711 |
| 1.383298206 | 5.58E-06 | 3.86E-05 | 44.97241168 | 81.13863595 | 50.4689446 | 129.404601 | 132.7362348 | 201.7618387 | Gm44829 |
| 1.017026528 | 5.63E-06 | 3.90E-05 | 111.494104 | 134.0154998 | 125.1209252 | 256.075302 | 190.5956192 | 306.7325251 | Gm31305 |
| 6.145814611 | 5.66E-06 | 3.92E-05 | 0.936925243 | 0 | 0 | 16.40340013 | 14.4648461 | 43.62418134 | 1700125G02Rik |
| 1.877277296 | 5.87E-06 | 4.04E-05 | 13.11695341 | 14.58672107 | 9.462927113 | 47.38760037 | 39.99104509 | 50.44045968 | Gm45346 |
| 1.805506918 | 5.92E-06 | 4.07E-05 | 27.17083206 | 42.84849314 | 16.82298153 | 84.75090065 | 76.57859699 | 145.8683564 | A930036I15Rik |
| 1.641280647 | 5.93E-06 | 4.07E-05 | 14.99080389 | 22.79175167 | 16.82298153 | 59.23450046 | 51.90327129 | 59.98324935 | Gm26507 |
| 1.111492019 | 6.14E-06 | 4.20E-05 | 89.94482336 | 97.54869715 | 128.2752342 | 170.4131013 | 272.279456 | 238.5697417 | Flywch2 |
| 1.342504647 | 6.23E-06 | 4.26E-05 | 48.72011266 | 35.55513261 | 62.03474441 | 97.50910075 | 122.5257552 | 151.321379 | Krt90 |
| 2.381948953 | 6.54E-06 | 4.46E-05 | 5.62155146 | 17.32173127 | 12.61723615 | 51.9441004 | 35.7366786 | 99.51766369 | Gm14824 |
| 1.58771941 | 7.15E-06 | 4.84E-05 | 27.17083206 | 25.52676187 | 21.02872692 | 51.03280039 | 85.93820329 | 85.88510702 | E230014E18Rik |
| 2.833125647 | 7.42E-06 | 5.00E-05 | 5.62155146 | 3.646680267 | 2.102872692 | 30.07290023 | 22.1227058 | 29.99162467 | Gm5912 |
| 5.936437367 | 7.76E-06 | 5.21E-05 | 0.936925243 | 0 | 0 | 13.66950011 | 22.9735791 | 27.26511334 | Spag11b |
| 1.749283923 | 7.79E-06 | 5.23E-05 | 19.67543011 | 10.02837074 | 26.28590865 | 61.05710047 | 62.96462419 | 62.70976068 | Col6a4 |
| 1.004381112 | 7.81E-06 | 5.23E-05 | 2640.255336 | 4363.25294 | 2364.680342 | 7023.389154 | 5177.56403 | 6595.430917 | Rasa1 |
| 1.401149172 | 7.92E-06 | 5.30E-05 | 57.15243985 | 123.075459 | 62.03474441 | 203.2199016 | 175.2798998 | 264.4715994 | Gm14827 |
| 1.231503641 | 8.05E-06 | 5.37E-05 | 36.54008449 | 41.02515301 | 36.80027211 | 79.28310061 | 88.49082319 | 102.244175 | Oscar |
| 1.714733527 | 8.16E-06 | 5.44E-05 | 39.35086022 | 51.96519381 | 32.59452672 | 120.2916009 | 77.42947029 | 212.6678841 | Gm26945 |
| 1.766267325 | 8.64E-06 | 5.72E-05 | 17.80157962 | 19.1450714 | 16.82298153 | 42.83110033 | 82.53471009 | 57.25673801 | Glp1r |
| 1.194349596 | 8.69E-06 | 5.75E-05 | 135.8541603 | 103.0187176 | 131.4295432 | 338.0923026 | 179.5342663 | 331.2711271 | Ptger3 |
| 3.24402883 | 8.86E-06 | 5.85E-05 | 2.81077573 | 3.646680267 | 1.051436346 | 18.22600014 | 24.6753257 | 29.99162467 | Gm26902 |
| 1.49110939 | 8.98E-06 | 5.92E-05 | 42.16163595 | 45.58350334 | 45.21176287 | 124.848101 | 75.72772369 | 175.859981 | Gm10827 |
| 1.289035006 | 8.99E-06 | 5.92E-05 | 48.72011266 | 39.20181288 | 30.49165403 | 87.48480068 | 91.04344309 | 113.1502204 | Gm45053 |
| 1.337574331 | 9.00E-06 | 5.92E-05 | 40.28778546 | 66.55191488 | 47.31463556 | 117.5577009 | 104.6574159 | 170.4069584 | Gm44560 |
| 1.023958568 | 9.39E-06 | 6.16E-05 | 289.5099002 | 473.1567647 | 260.7562138 | 795.5649061 | 605.8217895 | 681.6278335 | St8sia4 |
| 1.245981878 | 9.52E-06 | 6.23E-05 | 1088.707133 | 2319.28865 | 937.8812205 | 3353.584026 | 3182.266142 | 3774.854942 | Fsd1l |
| 2.814921627 | 1.00E-05 | 6.54E-05 | 11.24310292 | 7.293360535 | 0 | 32.80680025 | 56.15763779 | 43.62418134 | Slc6a16 |
| 1.036049561 | 1.03E-05 | 6.73E-05 | 132.1064593 | 196.9207344 | 130.3781069 | 376.3669029 | 251.0076235 | 316.2753147 | Spink10 |
| 2.295830492 | 1.09E-05 | 7.02E-05 | 8.43232719 | 9.116700669 | 9.462927113 | 22.78250018 | 59.56113099 | 50.44045968 | Fer1l6 |
| 1.507028495 | 1.09E-05 | 7.02E-05 | 29.98160779 | 30.99678227 | 39.95458114 | 64.7023005 | 92.74518969 | 130.872544 | Pdlim3 |
| 4.079925688 | 1.15E-05 | 7.41E-05 | 1.873850487 | 1.823340134 | 0 | 24.60510019 | 14.4648461 | 25.90185767 | Cd163 |
| 2.460348154 | 1.16E-05 | 7.49E-05 | 10.30617768 | 5.470020401 | 3.154309038 | 25.5164002 | 39.14017179 | 40.89767001 | Atp2c2 |
| 2.332671127 | 1.21E-05 | 7.75E-05 | 8.43232719 | 5.470020401 | 8.411490767 | 22.78250018 | 45.94715819 | 43.62418134 | Gm20638 |
| 2.289743096 | 1.23E-05 | 7.90E-05 | 19.67543011 | 8.205030602 | 9.462927113 | 33.71810026 | 52.75414459 | 98.15440802 | Angptl1 |
| 1.845333567 | 1.24E-05 | 7.93E-05 | 18.73850487 | 12.76338094 | 16.82298153 | 41.00850032 | 51.05239799 | 83.15859569 | Slc23a3 |
| 2.138328943 | 1.24E-05 | 7.95E-05 | 16.86465438 | 8.205030602 | 8.411490767 | 32.80680025 | 46.79803149 | 69.52603902 | Gm31518 |
| 1.535977557 | 1.24E-05 | 7.96E-05 | 28.1077573 | 21.8800816 | 25.2344723 | 51.9441004 | 74.87685039 | 92.70138536 | Gm15477 |
| 1.05326381 | 1.28E-05 | 8.16E-05 | 116.1787302 | 93.90201689 | 108.2979436 | 190.4617015 | 182.0868862 | 291.7367127 | Gucy2g |
| 1.251980686 | 1.30E-05 | 8.28E-05 | 67.45861752 | 70.19859515 | 56.77756268 | 131.227201 | 119.122262 | 216.7576511 | Cbln2 |
| 3.222268352 | 1.36E-05 | 8.57E-05 | 3.747700973 | 1.823340134 | 2.102872692 | 15.49210012 | 25.526199 | 31.35488034 | Cd200r2 |
| 2.978591187 | 1.38E-05 | 8.74E-05 | 3.747700973 | 2.735010201 | 3.154309038 | 16.40340013 | 32.3331854 | 27.26511334 | Gm11605 |
| 1.866646543 | 1.39E-05 | 8.79E-05 | 14.05387865 | 12.76338094 | 8.411490767 | 48.29890037 | 37.43842519 | 43.62418134 | Gm13502 |
| 1.16816761 | 1.42E-05 | 8.92E-05 | 55.27858936 | 80.22696588 | 73.60054421 | 131.227201 | 136.9906013 | 204.4883501 | Srrm4os |
| 1.104634755 | 1.42E-05 | 8.92E-05 | 1721.131672 | 3540.92654 | 1686.503899 | 5334.750241 | 4583.654466 | 5026.323644 | Scai |
| 1.257681948 | 1.48E-05 | 9.24E-05 | 43.09856119 | 33.73179247 | 33.64596307 | 101.1543008 | 74.87685039 | 88.61161836 | B4galnt3 |
| 3.729422339 | 1.49E-05 | 9.34E-05 | 1.873850487 | 0.911670067 | 2.102872692 | 14.58080011 | 28.9296922 | 20.44883501 | Csrp3 |
| 4.055893785 | 1.50E-05 | 9.37E-05 | 2.81077573 | 0.911670067 | 0 | 15.49210012 | 21.2718325 | 27.26511334 | Krt33a |
| 1.823769096 | 1.57E-05 | 9.71E-05 | 19.67543011 | 8.205030602 | 13.6686725 | 51.03280039 | 46.79803149 | 49.07720401 | Tarm1 |
| 1.586078557 | 1.57E-05 | 9.72E-05 | 22.48620584 | 23.70342174 | 22.08016326 | 45.56500035 | 74.87685039 | 85.88510702 | Chrm2 |
| 2.641997863 | 1.68E-05 | 0.000103585 | 5.62155146 | 5.470020401 | 3.154309038 | 30.98420024 | 18.7192126 | 40.89767001 | Il27 |
| 2.447190585 | 1.71E-05 | 0.000104946 | 17.80157962 | 2.735010201 | 6.308618075 | 53.76670042 | 37.43842519 | 55.89348235 | Pdcd1lg2 |
| 1.143321141 | 1.76E-05 | 0.000107591 | 1671.474634 | 3243.722098 | 1383.690231 | 4499.088135 | 4207.568468 | 5210.363159 | Trip11 |
| 6.032708698 | 1.98E-05 | 0.000119455 | 0 | 0 | 1.051436346 | 8.201700063 | 44.24541159 | 14.99581234 | Ano7 |
| 1.581777134 | 2.02E-05 | 0.000121405 | 20.61235535 | 23.70342174 | 13.6686725 | 60.14580046 | 48.49977809 | 66.79952768 | Gm20125 |
| 2.21771369 | 2.06E-05 | 0.000124049 | 10.30617768 | 10.9400408 | 4.205745383 | 36.45200028 | 53.60501789 | 28.62836901 | Mcemp1 |
| 1.278403394 | 2.08E-05 | 0.000124758 | 32.79238352 | 34.64346254 | 25.2344723 | 81.10570063 | 73.17510379 | 70.88929468 | Gm3985 |
| 3.250559327 | 2.08E-05 | 0.000124776 | 2.81077573 | 5.470020401 | 2.102872692 | 40.09720031 | 10.2104796 | 50.44045968 | Gm18486 |
| 1.291530214 | 2.15E-05 | 0.000128608 | 27.17083206 | 28.26177207 | 31.54309038 | 63.79100049 | 76.57859699 | 72.25255035 | Actl10 |
| 1.89084923 | 2.18E-05 | 0.000130452 | 14.05387865 | 15.49839114 | 11.5657998 | 49.21020038 | 33.1840587 | 72.25255035 | Ptprq |
| 1.510030603 | 2.19E-05 | 0.000130875 | 101.1879263 | 125.8104692 | 140.8924703 | 231.4702018 | 221.227058 | 598.4692378 | Rtl1 |
| 1.263996533 | 2.21E-05 | 0.000131916 | 43.09856119 | 37.37847274 | 36.80027211 | 71.99270056 | 94.44693629 | 117.2399874 | Ccl17 |
| 4.629324812 | 2.21E-05 | 0.000132057 | 0 | 10.02837074 | 9.462927113 | 83.83960065 | 213.5691983 | 184.039515 | Gpr101 |
| 1.279007015 | 2.26E-05 | 0.0001346 | 37.47700973 | 41.02515301 | 34.69739941 | 78.3718006 | 79.13121689 | 119.9664987 | Gm42853 |
| 1.702791248 | 2.26E-05 | 0.000134732 | 11.24310292 | 17.32173127 | 15.77154519 | 49.21020038 | 39.99104509 | 55.89348235 | Cstl1 |
| 1.831636465 | 2.26E-05 | 0.000134732 | 14.99080389 | 10.9400408 | 12.61723615 | 33.71810026 | 57.00851109 | 46.35069268 | Gm32585 |
| 3.639544503 | 2.29E-05 | 0.000135885 | 2.81077573 | 0 | 3.154309038 | 15.49210012 | 18.7192126 | 39.53441434 | Gm43016 |
| 1.772845882 | 2.44E-05 | 0.000144343 | 24.36005633 | 14.58672107 | 18.92585423 | 40.09720031 | 89.34169649 | 68.16278335 | Gm29595 |
| 3.425563314 | 2.67E-05 | 0.000156231 | 1.873850487 | 6.381690468 | 1.051436346 | 14.58080011 | 22.9735791 | 65.43627202 | Gm43606 |
| 1.009943995 | 2.88E-05 | 0.000167591 | 95.56637482 | 57.43521421 | 89.3720894 | 159.4775012 | 164.2185469 | 163.59068 | CK137956 |
| 1.845119562 | 2.98E-05 | 0.000172951 | 11.24310292 | 18.23340134 | 17.87441788 | 34.62940027 | 76.57859699 | 58.61999368 | Gm12108 |
| 1.719359934 | 3.04E-05 | 0.000176501 | 27.17083206 | 17.32173127 | 48.36607191 | 63.79100049 | 123.3766285 | 117.2399874 | BC024139 |
| 2.536460443 | 3.14E-05 | 0.000181538 | 4.684626217 | 2.735010201 | 8.411490767 | 20.04860015 | 36.58755189 | 34.08139168 | 5830405F06Rik |
| 2.5270875 | 3.15E-05 | 0.000181764 | 8.43232719 | 6.381690468 | 5.257181729 | 17.31470013 | 60.41200429 | 38.17115868 | R3hdml |
| 1.508555098 | 3.17E-05 | 0.000182982 | 25.29698157 | 18.23340134 | 15.77154519 | 50.12150039 | 57.00851109 | 62.70976068 | Gm15775 |
| 1.144713104 | 3.19E-05 | 0.000183755 | 55.27858936 | 47.40684348 | 52.57181729 | 101.1543008 | 96.14868289 | 148.5948677 | Ccdc188 |
| 2.22024157 | 3.20E-05 | 0.000184256 | 8.43232719 | 8.205030602 | 13.6686725 | 21.87120017 | 60.41200429 | 58.61999368 | Gm44386 |
| 2.319687536 | 3.28E-05 | 0.000188234 | 6.558476704 | 7.293360535 | 9.462927113 | 20.95990016 | 57.85938439 | 36.80790301 | Ppm1j |
| 1.08128737 | 3.30E-05 | 0.000189443 | 124.6110574 | 85.69698628 | 95.68070747 | 159.4775012 | 212.718325 | 278.1041561 | Cmbl |
| 1.10175331 | 3.66E-05 | 0.000207978 | 213.6189555 | 183.2456834 | 214.4930146 | 275.2126021 | 611.7779026 | 423.9725124 | Adssl1 |
| 1.319912863 | 3.66E-05 | 0.000208263 | 37.47700973 | 40.11348294 | 36.80027211 | 63.79100049 | 117.4205154 | 104.9706864 | Oit3 |
| 1.103745943 | 3.70E-05 | 0.000210129 | 141.4757117 | 134.0154998 | 132.4809796 | 184.9939014 | 393.1034645 | 298.5529911 | Sphk1 |
| 1.142753258 | 3.73E-05 | 0.00021193 | 44.03548644 | 58.34688428 | 52.57181729 | 88.39610068 | 116.5696421 | 139.052078 | 4930590J08Rik |
| 1.146476667 | 3.74E-05 | 0.000212286 | 902.2590094 | 1831.545164 | 854.8177492 | 2965.370223 | 1977.429549 | 3004.61549 | Tbc1d8b |
| 1.10082428 | 3.82E-05 | 0.000216174 | 915.3759628 | 1258.104692 | 917.9039299 | 1509.112812 | 1796.193536 | 3330.433594 | Kcnq1ot1 |
| 1.688474806 | 3.87E-05 | 0.000219032 | 18.73850487 | 27.35010201 | 19.97729057 | 40.09720031 | 81.68383679 | 92.70138536 | Gm14221 |
| 1.692113336 | 3.94E-05 | 0.000222532 | 16.86465438 | 13.675051 | 11.5657998 | 53.76670042 | 45.09628489 | 36.80790301 | Avpr1b |
| 1.017488206 | 3.96E-05 | 0.000223119 | 240.7897875 | 198.7440746 | 267.0648319 | 486.6342038 | 318.2266142 | 628.4608625 | Csf2rb2 |
| 1.064536211 | 3.98E-05 | 0.000224367 | 1217.065891 | 2460.59751 | 1111.368218 | 3470.230427 | 3138.871603 | 3409.502423 | Gm45884 |
| 1.070156152 | 3.98E-05 | 0.000224406 | 154.5926652 | 131.2804896 | 133.5324159 | 247.8736019 | 218.6744381 | 418.5194898 | Kcnab3 |
| 3.088352683 | 4.03E-05 | 0.000226871 | 0.936925243 | 5.470020401 | 2.102872692 | 15.49210012 | 25.526199 | 32.71813601 | 9430037O13Rik |
| 1.01891136 | 4.05E-05 | 0.00022781 | 742.0447927 | 1402.148563 | 657.1477162 | 1922.843015 | 1851.500301 | 1904.468167 | Igip |
| 1.870497999 | 4.20E-05 | 0.000235188 | 6.558476704 | 27.35010201 | 17.87441788 | 57.41190044 | 57.85938439 | 74.97906169 | Gm44830 |
| 1.493939119 | 4.26E-05 | 0.000238604 | 18.73850487 | 37.37847274 | 34.69739941 | 60.14580046 | 102.9556693 | 92.70138536 | Rad54b |
| 5.185770614 | 4.35E-05 | 0.000242976 | 0 | 0 | 2.102872692 | 7.290400056 | 40.84191839 | 23.17534634 | Tcp11x2 |
| 1.056593418 | 4.46E-05 | 0.000248351 | 61.83706606 | 45.58350334 | 54.67468999 | 96.59780075 | 112.3152756 | 129.5092884 | Lyve1 |
| 1.253904275 | 4.58E-05 | 0.000253892 | 29.04468254 | 36.46680267 | 31.54309038 | 63.79100049 | 76.57859699 | 92.70138536 | Efcab8 |
| 1.082118543 | 4.58E-05 | 0.000253922 | 332.6084614 | 218.800816 | 362.7455393 | 426.4884033 | 842.3645669 | 665.2687655 | Egr2 |
| 1.477836983 | 4.78E-05 | 0.000263612 | 18.73850487 | 20.05674147 | 25.2344723 | 52.85540041 | 50.20152469 | 76.34231735 | Gm6225 |
| 1.037280724 | 4.79E-05 | 0.000263972 | 653.9738199 | 1279.073104 | 631.9132439 | 1733.292613 | 1515.405347 | 2018.981643 | Uba6 |
| 2.519182662 | 4.82E-05 | 0.000265394 | 2.81077573 | 7.293360535 | 9.462927113 | 18.22600014 | 53.60501789 | 39.53441434 | Retn |
| 3.372629514 | 5.07E-05 | 0.000277871 | 1.873850487 | 0.911670067 | 3.154309038 | 20.95990016 | 12.7630995 | 27.26511334 | Serpinb12 |
| 3.433189276 | 5.13E-05 | 0.000280742 | 11.24310292 | 21.8800816 | 15.77154519 | 38.2746003 | 318.2266142 | 171.770214 | Xirp1 |
| 2.956819153 | 5.60E-05 | 0.000303538 | 2.81077573 | 3.646680267 | 13.6686725 | 15.49210012 | 91.89431639 | 46.35069268 | Pkp1 |
| 1.488514278 | 5.69E-05 | 0.00030843 | 71.2063185 | 209.6841154 | 79.90916229 | 333.5358026 | 257.8146099 | 423.9725124 | Hdx |
| 1.652884228 | 5.70E-05 | 0.000308727 | 47.78318741 | 20.05674147 | 15.77154519 | 73.81530057 | 88.49082319 | 102.244175 | Pecam1 |
| 2.087296528 | 5.74E-05 | 0.000311011 | 7.495401947 | 4.558350334 | 10.51436346 | 28.25030022 | 34.034932 | 32.71813601 | Gm31592 |
| 1.267542866 | 6.06E-05 | 0.000326119 | 35.60315925 | 38.29014281 | 27.33734499 | 92.04130071 | 62.11375089 | 91.33812969 | Anks4b |
| 3.184677126 | 6.29E-05 | 0.000338068 | 3.747700973 | 0 | 3.154309038 | 20.04860015 | 21.2718325 | 20.44883501 | Glod5 |
| 1.203016046 | 6.42E-05 | 0.000344084 | 120.8633564 | 246.1509181 | 116.7094344 | 381.8347029 | 282.4899356 | 452.6008814 | C330027C09Rik |
| 1.206451501 | 6.76E-05 | 0.000360465 | 32.79238352 | 67.46358495 | 44.16032653 | 102.9769008 | 116.5696421 | 114.513476 | Ano5 |
| 1.660125198 | 6.80E-05 | 0.000362187 | 17.80157962 | 28.26177207 | 15.77154519 | 61.96840048 | 43.39453829 | 92.70138536 | E530001F21Rik |
| 3.45643479 | 6.80E-05 | 0.000362187 | 0.936925243 | 2.735010201 | 2.102872692 | 20.04860015 | 10.2104796 | 34.08139168 | 1700073E17Rik |
| 1.651637492 | 7.04E-05 | 0.000372878 | 18.73850487 | 23.70342174 | 15.77154519 | 41.91980032 | 87.63994989 | 53.16697101 | Gm5432 |
| 2.227255362 | 7.29E-05 | 0.000385462 | 5.62155146 | 7.293360535 | 16.82298153 | 26.4277002 | 41.69279169 | 70.88929468 | Gm45205 |
| 1.162692952 | 7.40E-05 | 0.000390396 | 66.52169228 | 67.46358495 | 48.36607191 | 98.42040076 | 131.8853615 | 181.3130037 | Arhgef37 |
| 1.695003461 | 7.45E-05 | 0.000392773 | 16.86465438 | 14.58672107 | 29.44021768 | 46.47630036 | 92.74518969 | 55.89348235 | Gm16184 |
| 1.727005696 | 7.52E-05 | 0.000395916 | 17.80157962 | 16.4100612 | 8.411490767 | 36.45200028 | 57.85938439 | 47.71394835 | Cmah |
| 2.389409806 | 7.63E-05 | 0.000401627 | 12.18002816 | 0.911670067 | 8.411490767 | 35.54070027 | 38.28929849 | 38.17115868 | Defb20 |
| 1.016864835 | 7.81E-05 | 0.000409802 | 1008.131562 | 1980.147385 | 898.9780757 | 2841.433422 | 2474.339556 | 2552.014609 | Hook1 |
| 2.307911836 | 7.83E-05 | 0.000410749 | 6.558476704 | 5.470020401 | 6.308618075 | 28.25030022 | 18.7192126 | 44.98743701 | Gm26984 |
| 1.788924823 | 8.03E-05 | 0.000420262 | 11.24310292 | 10.9400408 | 16.82298153 | 29.16160023 | 51.05239799 | 54.53022668 | Epcam |
| 1.322304947 | 8.28E-05 | 0.000432276 | 32.79238352 | 28.26177207 | 34.69739941 | 95.68650074 | 54.45589119 | 89.97487402 | Gm15577 |
| 2.050256194 | 8.34E-05 | 0.000435261 | 7.495401947 | 7.293360535 | 6.308618075 | 26.4277002 | 26.3770723 | 35.44464734 | Gm6999 |
| 1.496869639 | 8.37E-05 | 0.000436592 | 22.48620584 | 13.675051 | 30.49165403 | 52.85540041 | 61.26287759 | 73.61580602 | Gm43172 |
| 3.593653136 | 8.64E-05 | 0.000449284 | 2.81077573 | 1.823340134 | 0 | 12.7582001 | 28.0788189 | 16.359068 | 4933402N03Rik |
| 1.58035661 | 8.86E-05 | 0.000458546 | 19.67543011 | 12.76338094 | 14.72010884 | 37.36330029 | 51.05239799 | 53.16697101 | Gm11915 |
| 2.941812065 | 9.04E-05 | 0.000466974 | 2.81077573 | 1.823340134 | 4.205745383 | 24.60510019 | 11.9122262 | 31.35488034 | Gm21954 |
| 2.663973269 | 9.05E-05 | 0.000467107 | 2.81077573 | 7.293360535 | 9.462927113 | 13.66950011 | 67.21899069 | 42.26092568 | Slc28a1 |
| 1.281718158 | 9.18E-05 | 0.000472872 | 35.60315925 | 58.34688428 | 47.31463556 | 74.72660058 | 136.139728 | 133.5990554 | Srpx2 |
| 1.270052023 | 9.45E-05 | 0.000485021 | 22.48620584 | 33.73179247 | 25.2344723 | 61.05710047 | 71.47335719 | 64.07301635 | Cdsn |
| 1.573814882 | 9.52E-05 | 0.000488125 | 20.61235535 | 24.61509181 | 14.72010884 | 39.1859003 | 74.87685039 | 65.43627202 | Trim15 |
| 1.179316558 | 9.61E-05 | 0.00049217 | 59.96321558 | 61.99356455 | 46.26319922 | 106.6221008 | 99.55217609 | 178.5864924 | Gm26703 |
| 2.322138945 | 9.62E-05 | 0.000492575 | 3.747700973 | 7.293360535 | 8.411490767 | 17.31470013 | 36.58755189 | 43.62418134 | Gm33968 |
| 1.087773259 | 0.000100475 | 0.000512369 | 50.59396314 | 70.19859515 | 47.31463556 | 133.049801 | 92.74518969 | 133.5990554 | Gm44562 |
| 1.074152303 | 0.000102159 | 0.000520266 | 51.53088839 | 41.02515301 | 44.16032653 | 84.75090065 | 114.8678955 | 87.24836269 | Gm20036 |
| 1.040301337 | 0.000105575 | 0.000536956 | 684.8923529 | 1427.675325 | 645.5819164 | 1924.665615 | 1681.325641 | 2069.422103 | Smarca1 |
| 1.39193311 | 0.000108118 | 0.000548442 | 27.17083206 | 47.40684348 | 22.08016326 | 80.19440062 | 69.77161059 | 106.333942 | Impg2 |
| 2.868302946 | 0.000110329 | 0.00055804 | 2.81077573 | 3.646680267 | 3.154309038 | 11.84690009 | 21.2718325 | 38.17115868 | Gm15997 |
| 1.031989344 | 0.00012207 | 0.000610542 | 59.96321558 | 92.99034682 | 50.4689446 | 135.783701 | 141.2449678 | 140.4153337 | A230057D06Rik |
| 1.830982776 | 0.00012214 | 0.000610731 | 26.23390681 | 9.116700669 | 12.61723615 | 65.61360051 | 36.58755189 | 69.52603902 | Ppp1r1c |
| 1.826359145 | 0.000127181 | 0.000633639 | 21.5492806 | 24.61509181 | 21.02872692 | 59.23450046 | 39.99104509 | 141.7785894 | Gm37140 |
| 1.011231078 | 0.00013702 | 0.000676374 | 1752.98713 | 3225.488697 | 1554.022919 | 4974.786738 | 3240.125526 | 4955.43435 | Gpr22 |
| 1.498130353 | 0.000138593 | 0.000682904 | 14.99080389 | 17.32173127 | 15.77154519 | 48.29890037 | 36.58755189 | 51.80371535 | Hhip |
| 1.036830212 | 0.000139083 | 0.000684621 | 169.583469 | 130.3688196 | 136.686725 | 258.809202 | 211.8674517 | 429.4255351 | Fbn2 |
| 2.273887269 | 0.000139721 | 0.000687585 | 3.747700973 | 7.293360535 | 8.411490767 | 24.60510019 | 21.2718325 | 49.07720401 | Gm37959 |
| 1.377776887 | 0.000142412 | 0.000699581 | 30.91853303 | 20.96841154 | 16.82298153 | 51.9441004 | 67.21899069 | 59.98324935 | 1700016A09Rik |
| 1.04527129 | 0.000143076 | 0.000702124 | 155.5295904 | 93.90201689 | 152.4582702 | 191.3730015 | 340.34932 | 297.1897354 | Bean1 |
| 1.338124341 | 0.000144409 | 0.000707768 | 77.7647952 | 72.02193528 | 99.88645286 | 247.8736019 | 101.2539227 | 283.5571787 | Tacr3 |
| 1.107677297 | 0.000146677 | 0.000718125 | 60.90014082 | 53.78853394 | 66.24048979 | 114.8238009 | 97.85042949 | 179.949748 | Dnah17 |
| 1.197839562 | 0.000146678 | 0.000718125 | 35.60315925 | 41.02515301 | 35.74883576 | 76.54920059 | 68.06986399 | 115.8767317 | Msantd1 |
| 1.227142147 | 0.000147537 | 0.000721448 | 73.08016898 | 49.23018361 | 62.03474441 | 99.33170077 | 206.7622119 | 124.0562657 | 4931415C17Rik |
| 1.212420987 | 0.000151434 | 0.000738253 | 128.3587583 | 121.2521189 | 139.841034 | 219.6233017 | 199.9552255 | 486.6822731 | Ttn |
| 2.167743352 | 0.000153173 | 0.000745604 | 10.30617768 | 15.49839114 | 3.154309038 | 65.61360051 | 28.9296922 | 36.80790301 | Gm6117 |
| 1.659922172 | 0.00016589 | 0.000801231 | 11.24310292 | 21.8800816 | 9.462927113 | 46.47630036 | 39.14017179 | 50.44045968 | Rad51ap2 |
| 1.421724576 | 0.000166148 | 0.000802079 | 36.54008449 | 28.26177207 | 21.02872692 | 76.54920059 | 51.05239799 | 104.9706864 | Zfp92 |
| 1.636784381 | 0.000173624 | 0.000832539 | 9.369252434 | 19.1450714 | 14.72010884 | 34.62940027 | 53.60501789 | 46.35069268 | Adgrf4 |
| 1.333339022 | 0.000175109 | 0.000838618 | 17.80157962 | 21.8800816 | 23.13159961 | 60.14580046 | 48.49977809 | 49.07720401 | Gm15337 |
| 1.973370326 | 0.000177869 | 0.000849513 | 13.11695341 | 5.470020401 | 8.411490767 | 34.62940027 | 24.6753257 | 47.71394835 | Pax1 |
| 1.599309412 | 0.000182555 | 0.000869527 | 22.48620584 | 12.76338094 | 25.2344723 | 58.32320045 | 39.99104509 | 85.88510702 | Wif1 |
| 1.977713469 | 0.000183425 | 0.000873097 | 16.86465438 | 10.02837074 | 8.411490767 | 22.78250018 | 66.36811739 | 50.44045968 | Drd2 |
| 3.323531037 | 0.000184685 | 0.000878804 | 3.747700973 | 0.911670067 | 1.051436346 | 9.11300007 | 25.526199 | 23.17534634 | Fam19a3 |
| 2.816009602 | 0.000184951 | 0.000879852 | 4.684626217 | 1.823340134 | 2.102872692 | 16.40340013 | 15.3157194 | 29.99162467 | Gm20730 |
| 1.643731828 | 0.000188939 | 0.000896831 | 15.92772914 | 27.35010201 | 18.92585423 | 34.62940027 | 86.78907659 | 73.61580602 | Arhgef15 |
| 1.69151765 | 0.000189626 | 0.000899512 | 9.369252434 | 18.23340134 | 12.61723615 | 32.80680025 | 56.15763779 | 40.89767001 | Gm18445 |
| 1.043297881 | 0.000193758 | 0.000916996 | 88.07097288 | 93.90201689 | 79.90916229 | 113.9125009 | 218.6744381 | 208.5781171 | Slc22a21 |
| 2.576040324 | 0.000202344 | 0.000953421 | 4.684626217 | 4.558350334 | 1.051436346 | 18.22600014 | 21.2718325 | 23.17534634 | P2rx1 |
| 1.694553891 | 0.000214065 | 0.001003988 | 16.86465438 | 8.205030602 | 14.72010884 | 39.1859003 | 33.1840587 | 57.25673801 | Gm6505 |
| 1.212216526 | 0.00021528 | 0.001008458 | 29.98160779 | 21.8800816 | 35.74883576 | 69.25880053 | 58.71025769 | 74.97906169 | Noxred1 |
| 1.024505585 | 0.000223793 | 0.001043773 | 432.8594624 | 852.4115125 | 350.1283032 | 1120.899009 | 1018.49534 | 1190.122197 | Zfp37 |
| 1.560611674 | 0.000224512 | 0.001046618 | 21.5492806 | 31.90845234 | 12.61723615 | 43.74240034 | 81.68383679 | 70.88929468 | Rec114 |
| 1.188804681 | 0.00023202 | 0.001077969 | 74.95401947 | 37.37847274 | 71.49767152 | 111.1786009 | 125.0783751 | 184.039515 | Obscn |
| 4.732490263 | 0.000242838 | 0.001120135 | 2.81077573 | 0 | 0 | 61.05710047 | 5.105239799 | 9.542789669 | Ccl24 |
| 2.688674291 | 0.000249914 | 0.001148926 | 0.936925243 | 5.470020401 | 3.154309038 | 17.31470013 | 17.8683393 | 27.26511334 | Gm44542 |
| 2.622465721 | 0.000251789 | 0.00115644 | 2.81077573 | 2.735010201 | 4.205745383 | 22.78250018 | 12.7630995 | 24.53860201 | Gm14637 |
| 1.235376987 | 0.000254425 | 0.001166601 | 21.5492806 | 34.64346254 | 37.85170845 | 63.79100049 | 67.21899069 | 91.33812969 | Gm43654 |
| 2.076310701 | 0.000260162 | 0.001190079 | 8.43232719 | 8.205030602 | 3.154309038 | 23.69380018 | 24.6753257 | 36.80790301 | Gm43686 |
| 1.23770663 | 0.000262225 | 0.001198948 | 22.48620584 | 29.17344214 | 26.28590865 | 52.85540041 | 56.15763779 | 76.34231735 | Mog |
| 1.268510633 | 0.000265342 | 0.001211474 | 25.29698157 | 34.64346254 | 21.02872692 | 52.85540041 | 77.42947029 | 65.43627202 | Gm6545 |
| 2.972955686 | 0.000270555 | 0.001232357 | 5.62155146 | 5.470020401 | 0 | 10.02430008 | 43.39453829 | 35.44464734 | Odf3l1 |
| 2.552821981 | 0.000283486 | 0.001283678 | 0.936925243 | 8.205030602 | 3.154309038 | 23.69380018 | 19.5700859 | 29.99162467 | 4930470B04Rik |
| 1.473611926 | 0.000290091 | 0.00130928 | 25.29698157 | 33.73179247 | 18.92585423 | 54.67800042 | 54.45589119 | 110.423709 | Gm44678 |
| 1.2115708 | 0.00029617 | 0.001331109 | 88.07097288 | 174.1289828 | 63.08618075 | 294.3499023 | 203.3587187 | 257.6553211 | Zfp850 |
| 1.550342853 | 0.000301966 | 0.001354317 | 24.36005633 | 36.46680267 | 30.49165403 | 68.34750053 | 51.90327129 | 149.9581234 | B830012L14Rik |
| 1.221092058 | 0.000318806 | 0.001423883 | 27.17083206 | 30.08511221 | 53.62325364 | 100.2430008 | 72.32423049 | 84.52185135 | Prkg2 |
| 1.042605477 | 0.000326544 | 0.00145339 | 84.3232719 | 44.67183328 | 66.24048979 | 138.5176011 | 108.0609091 | 156.7744017 | 4930413G21Rik |
| 1.008598047 | 0.000331143 | 0.001471143 | 59.02629033 | 48.31851354 | 56.77756268 | 105.7108008 | 85.08732999 | 141.7785894 | Fam69c |
| 2.272053936 | 0.000334737 | 0.001485742 | 11.24310292 | 10.9400408 | 7.360054421 | 18.22600014 | 92.74518969 | 31.35488034 | Myo1a |
| 1.305554406 | 0.000335784 | 0.001490045 | 15.92772914 | 25.52676187 | 25.2344723 | 51.03280039 | 63.81549749 | 49.07720401 | Gm26633 |
| 1.961930765 | 0.000336516 | 0.001492282 | 6.558476704 | 9.116700669 | 7.360054421 | 18.22600014 | 36.58755189 | 35.44464734 | Rnf183 |
| 1.400217241 | 0.00034442 | 0.001524861 | 20.61235535 | 52.87686388 | 28.38878134 | 95.68650074 | 64.66637079 | 110.423709 | Gm16054 |
| 1.964693694 | 0.000346395 | 0.001532901 | 11.24310292 | 10.9400408 | 21.02872692 | 20.95990016 | 64.66637079 | 83.15859569 | Slc12a3 |
| 1.38466605 | 0.000366037 | 0.001610946 | 19.67543011 | 13.675051 | 22.08016326 | 40.09720031 | 47.64890479 | 57.25673801 | Gm48677 |
| 2.674540867 | 0.000369318 | 0.001623532 | 0 | 7.293360535 | 8.411490767 | 18.22600014 | 51.05239799 | 29.99162467 | Gm11885 |
| 2.406388908 | 0.000374832 | 0.001644772 | 5.62155146 | 0.911670067 | 7.360054421 | 18.22600014 | 27.2279456 | 27.26511334 | C8b |
| 1.748398209 | 0.000375248 | 0.001646225 | 11.24310292 | 6.381690468 | 16.82298153 | 34.62940027 | 30.6314388 | 50.44045968 | Gm42797 |
| 2.343792865 | 0.000380387 | 0.001666115 | 1.873850487 | 5.470020401 | 5.257181729 | 20.95990016 | 24.6753257 | 17.72232367 | Als2cr12 |
| 1.924953248 | 0.000384899 | 0.001682437 | 6.558476704 | 11.85171087 | 17.87441788 | 21.87120017 | 54.45589119 | 61.34650502 | Bpifc |
| 1.448261073 | 0.000398146 | 0.001734055 | 25.29698157 | 79.31529582 | 29.44021768 | 113.0012009 | 106.3591625 | 148.5948677 | Far1os |
| 1.431705919 | 0.000404442 | 0.001760279 | 14.05387865 | 18.23340134 | 15.77154519 | 51.03280039 | 34.034932 | 44.98743701 | 4930471C04Rik |
| 1.428612675 | 0.000407813 | 0.001771752 | 22.48620584 | 13.675051 | 18.92585423 | 54.67800042 | 35.7366786 | 58.61999368 | Gm36937 |
| 1.094004658 | 0.000417318 | 0.001808042 | 36.54008449 | 58.34688428 | 56.77756268 | 133.049801 | 79.13121689 | 111.7869647 | 4732496C06Rik |
| 1.917620609 | 0.000420812 | 0.001821661 | 6.558476704 | 10.9400408 | 5.257181729 | 25.5164002 | 23.8244524 | 38.17115868 | Eqtn |
| 1.248599404 | 0.00043038 | 0.001855165 | 20.61235535 | 28.26177207 | 34.69739941 | 82.92830064 | 62.11375089 | 51.80371535 | Gm15432 |
| 1.749404279 | 0.000440509 | 0.001894167 | 10.30617768 | 10.02837074 | 12.61723615 | 25.5164002 | 30.6314388 | 55.89348235 | Gm14377 |
| 1.326386455 | 0.000448085 | 0.001922457 | 18.73850487 | 18.23340134 | 19.97729057 | 37.36330029 | 50.20152469 | 55.89348235 | Ripply2 |
| 1.838834547 | 0.000450695 | 0.001932795 | 6.558476704 | 14.58672107 | 6.308618075 | 25.5164002 | 33.1840587 | 40.89767001 | Hao1 |
| 1.516784297 | 0.000466236 | 0.00199191 | 13.11695341 | 18.23340134 | 10.51436346 | 47.38760037 | 32.3331854 | 40.89767001 | Gm37166 |
| 1.52023921 | 0.000467405 | 0.001996465 | 25.29698157 | 11.85171087 | 13.6686725 | 48.29890037 | 37.43842519 | 61.34650502 | Gm29521 |
| 1.846660991 | 0.000470385 | 0.002006969 | 12.18002816 | 13.675051 | 11.5657998 | 22.78250018 | 37.43842519 | 76.34231735 | Mir453 |
| 1.428040994 | 0.000482214 | 0.002053209 | 37.47700973 | 18.23340134 | 33.64596307 | 50.12150039 | 75.72772369 | 115.8767317 | Pax5 |
| 1.450279317 | 0.00049178 | 0.002089468 | 101.1879263 | 41.93682308 | 100.9378892 | 262.454402 | 101.2539227 | 304.0060137 | Spaar |
| 1.469689866 | 0.000496748 | 0.00210779 | 63.71091655 | 64.72857475 | 72.54910787 | 84.75090065 | 332.6914603 | 137.6888224 | Npr1 |
| 1.526513473 | 0.000503276 | 0.002131735 | 11.24310292 | 19.1450714 | 11.5657998 | 48.29890037 | 32.3331854 | 40.89767001 | Gm44723 |
| 1.717297301 | 0.000504003 | 0.00213388 | 6.558476704 | 14.58672107 | 11.5657998 | 33.71810026 | 27.2279456 | 47.71394835 | Gm13423 |
| 1.919291363 | 0.000507196 | 0.002145512 | 7.495401947 | 3.646680267 | 12.61723615 | 25.5164002 | 33.1840587 | 29.99162467 | Gm15457 |
| 1.068189589 | 0.000508157 | 0.002147691 | 69.33246801 | 48.31851354 | 56.77756268 | 80.19440062 | 149.7537008 | 136.3255667 | Hes7 |
| 2.587451784 | 0.000508111 | 0.002147691 | 1.873850487 | 8.205030602 | 1.051436346 | 19.13730015 | 19.5700859 | 29.99162467 | Gm4734 |
| 3.08009465 | 0.000530074 | 0.002227143 | 1.873850487 | 3.646680267 | 2.102872692 | 5.467800042 | 37.43842519 | 21.81209067 | Shbg |
| 2.215768844 | 0.000548148 | 0.002292592 | 11.24310292 | 8.205030602 | 2.102872692 | 21.87120017 | 24.6753257 | 55.89348235 | Gm5177 |
| 2.51389201 | 0.000564923 | 0.002351529 | 2.81077573 | 3.646680267 | 4.205745383 | 10.02430008 | 26.3770723 | 24.53860201 | 1700110K17Rik |
| 1.550285141 | 0.000577049 | 0.002396314 | 9.369252434 | 14.58672107 | 15.77154519 | 30.07290023 | 36.58755189 | 50.44045968 | Nppa |
| 2.664144647 | 0.000594739 | 0.002460764 | 1.873850487 | 3.646680267 | 6.308618075 | 8.201700063 | 37.43842519 | 28.62836901 | Gm10706 |
| 1.150682263 | 0.000609116 | 0.00251324 | 25.29698157 | 24.61509181 | 30.49165403 | 50.12150039 | 57.00851109 | 72.25255035 | Gm16287 |
| 1.6851873 | 0.000613044 | 0.002527823 | 9.369252434 | 17.32173127 | 38.9031448 | 51.9441004 | 60.41200429 | 98.15440802 | Sycp2 |
| 2.247757752 | 0.00062118 | 0.00255809 | 2.81077573 | 6.381690468 | 4.205745383 | 16.40340013 | 28.0788189 | 19.08557934 | Gm19299 |
| 2.047139302 | 0.000623775 | 0.002567679 | 9.369252434 | 7.293360535 | 6.308618075 | 16.40340013 | 51.05239799 | 27.26511334 | Prss16 |
| 1.982054026 | 0.000637361 | 0.00261913 | 4.684626217 | 12.76338094 | 6.308618075 | 35.54070027 | 18.7192126 | 40.89767001 | Gm38451 |
| 1.233557099 | 0.000646432 | 0.002654142 | 35.60315925 | 59.25855435 | 33.64596307 | 97.50910075 | 67.21899069 | 140.4153337 | Gm44667 |
| 1.875200903 | 0.000654343 | 0.002684338 | 24.36005633 | 15.49839114 | 6.308618075 | 31.89550025 | 45.94715819 | 94.06464102 | Hrc |
| 1.275570184 | 0.000655842 | 0.00268877 | 21.5492806 | 25.52676187 | 22.08016326 | 38.2746003 | 62.11375089 | 68.16278335 | Gm42890 |
| 1.929918066 | 0.000668408 | 0.002733895 | 10.30617768 | 9.116700669 | 4.205745383 | 43.74240034 | 24.6753257 | 21.81209067 | Gm12374 |
| 1.252288598 | 0.00068657 | 0.002799863 | 22.48620584 | 25.52676187 | 18.92585423 | 61.96840048 | 39.99104509 | 58.61999368 | Gm37181 |
| 1.110593385 | 0.000693649 | 0.00282634 | 34.666234 | 51.05352374 | 55.72612633 | 71.99270056 | 98.70130279 | 136.3255667 | Gm42456 |
| 1.099818402 | 0.000698001 | 0.002843472 | 29.04468254 | 30.08511221 | 41.00601749 | 55.58930043 | 74.87685039 | 84.52185135 | Gm28424 |
| 2.308403502 | 0.00069849 | 0.002844655 | 4.684626217 | 1.823340134 | 10.51436346 | 37.36330029 | 29.7805655 | 14.99581234 | Gm3248 |
| 2.506176384 | 0.000698586 | 0.002844655 | 1.873850487 | 6.381690468 | 2.102872692 | 15.49210012 | 16.1665927 | 28.62836901 | Gm37342 |
| 2.6185498 | 0.0007075 | 0.002876699 | 3.747700973 | 0.911670067 | 4.205745383 | 19.13730015 | 11.9122262 | 23.17534634 | 4930470P17Rik |
| 1.203489858 | 0.000708956 | 0.002881404 | 18.73850487 | 36.46680267 | 26.28590865 | 69.25880053 | 53.60501789 | 65.43627202 | Gm38080 |
| 2.129083153 | 0.000725884 | 0.002941522 | 7.495401947 | 2.735010201 | 11.5657998 | 24.60510019 | 20.4209592 | 50.44045968 | Gjd4 |
| 1.226521413 | 0.000733356 | 0.002964949 | 22.48620584 | 19.1450714 | 31.54309038 | 57.41190044 | 45.94715819 | 68.16278335 | Gm45151 |
| 1.604799235 | 0.000733872 | 0.002965791 | 10.30617768 | 11.85171087 | 11.5657998 | 23.69380018 | 43.39453829 | 35.44464734 | Gm37359 |
| 1.478587993 | 0.000742727 | 0.002999062 | 45.90933693 | 64.72857475 | 55.72612633 | 96.59780075 | 82.53471009 | 287.6469457 | 9530059O14Rik |
| 1.043888243 | 0.000783843 | 0.003151225 | 36.54008449 | 31.90845234 | 35.74883576 | 55.58930043 | 75.72772369 | 84.52185135 | Gm42738 |
| 2.075854385 | 0.000806044 | 0.003231048 | 4.684626217 | 5.470020401 | 10.51436346 | 31.89550025 | 14.4648461 | 40.89767001 | Mup5 |
| 1.858238881 | 0.000814685 | 0.003260938 | 13.11695341 | 10.9400408 | 4.205745383 | 40.09720031 | 20.4209592 | 43.62418134 | Prss52 |
| 1.271798641 | 0.000849577 | 0.003384386 | 19.67543011 | 19.1450714 | 26.28590865 | 41.91980032 | 46.79803149 | 69.52603902 | Skap1 |
| 1.017406837 | 0.000892046 | 0.003535364 | 118.0525807 | 263.4726493 | 116.7094344 | 350.8505027 | 282.4899356 | 377.6218198 | Gm10033 |
| 1.314731893 | 0.000905926 | 0.003583185 | 14.05387865 | 22.79175167 | 17.87441788 | 54.67800042 | 39.14017179 | 42.26092568 | Gm9403 |
| 1.229409867 | 0.000941981 | 0.003706601 | 28.1077573 | 24.61509181 | 19.97729057 | 47.38760037 | 47.64890479 | 77.70557302 | Gm16701 |
| 1.058075502 | 0.00094477 | 0.003715933 | 27.17083206 | 42.84849314 | 36.80027211 | 59.23450046 | 78.28034359 | 85.88510702 | Gm15764 |
| 1.553301838 | 0.00095168 | 0.003739421 | 14.99080389 | 20.05674147 | 11.5657998 | 42.83110033 | 28.0788189 | 68.16278335 | Pcsk2os2 |
| 1.217387777 | 0.000984986 | 0.003861633 | 29.98160779 | 37.37847274 | 31.54309038 | 55.58930043 | 61.26287759 | 115.8767317 | Gm43486 |
| 1.872298553 | 0.000999939 | 0.003910716 | 29.04468254 | 6.381690468 | 9.462927113 | 82.01700063 | 37.43842519 | 44.98743701 | F10 |
| 1.001495081 | 0.00100594 | 0.003932588 | 350.410041 | 609.9072747 | 262.8590865 | 852.9768066 | 551.3658983 | 1048.343608 | Acvr1c |
| 2.854547239 | 0.001006907 | 0.003934773 | 1.873850487 | 3.646680267 | 3.154309038 | 11.84690009 | 8.508732999 | 43.62418134 | Gm26854 |
| 1.549208429 | 0.001008194 | 0.003939006 | 13.11695341 | 16.4100612 | 15.77154519 | 28.25030022 | 36.58755189 | 69.52603902 | 4930412M03Rik |
| 1.21992887 | 0.001014676 | 0.003959514 | 36.54008449 | 15.49839114 | 35.74883576 | 66.52490051 | 74.02597709 | 62.70976068 | Gm37168 |
| 2.138230977 | 0.001015813 | 0.00396315 | 3.747700973 | 4.558350334 | 4.205745383 | 18.22600014 | 17.8683393 | 19.08557934 | Gm17751 |
| 1.216728098 | 0.00103018 | 0.004009469 | 16.86465438 | 29.17344214 | 19.97729057 | 48.29890037 | 47.64890479 | 58.61999368 | Gm27010 |
| 1.260614315 | 0.001033995 | 0.004021881 | 20.61235535 | 18.23340134 | 16.82298153 | 35.54070027 | 49.35065139 | 49.07720401 | Gm15801 |
| 2.38242536 | 0.001048646 | 0.004072294 | 4.684626217 | 3.646680267 | 3.154309038 | 10.02430008 | 19.5700859 | 31.35488034 | Gm13912 |
| 1.08359886 | 0.00105938 | 0.004110665 | 89.00789812 | 213.3307956 | 88.32065305 | 259.720502 | 238.244524 | 332.6343827 | A330076H08Rik |
| 1.143299792 | 0.001062889 | 0.004122783 | 96.50330007 | 42.84849314 | 64.1376171 | 114.8238009 | 124.2275018 | 212.6678841 | Baiap2l2 |
| 1.343835869 | 0.001065784 | 0.004132189 | 11.24310292 | 19.1450714 | 19.97729057 | 44.65370034 | 35.7366786 | 47.71394835 | Cyp24a1 |
| 1.241065233 | 0.001070672 | 0.004147804 | 75.89094471 | 35.55513261 | 26.28590865 | 122.1142009 | 90.19256979 | 114.513476 | Gbp4 |
| 1.499428244 | 0.001086794 | 0.004200978 | 31.85545827 | 16.4100612 | 11.5657998 | 56.50060044 | 38.28929849 | 76.34231735 | Gimap8 |
| 1.142738333 | 0.00109972 | 0.004239891 | 37.47700973 | 70.19859515 | 37.85170845 | 139.4289011 | 76.57859699 | 106.333942 | Gm43300 |
| 3.4854066 | 0.001103653 | 0.004253355 | 0 | 5.470020401 | 1.051436346 | 6.379100049 | 12.7630995 | 55.89348235 | Mir493 |
| 1.90178828 | 0.001114297 | 0.004283619 | 15.92772914 | 15.49839114 | 4.205745383 | 20.04860015 | 58.71025769 | 55.89348235 | Dpys |
| 2.204078822 | 0.001121257 | 0.004303992 | 6.558476704 | 7.293360535 | 1.051436346 | 18.22600014 | 19.5700859 | 32.71813601 | Gm38124 |
| 1.120007038 | 0.001122811 | 0.00430849 | 59.02629033 | 152.2489012 | 69.39479883 | 185.9052014 | 174.4290265 | 252.2022984 | Pabpc5 |
| 1.050980523 | 0.001138226 | 0.004360448 | 41.22471071 | 31.90845234 | 29.44021768 | 71.08140055 | 56.15763779 | 87.24836269 | Gm37941 |
| 1.381469019 | 0.001162401 | 0.004443349 | 18.73850487 | 51.05352374 | 25.2344723 | 77.4605006 | 57.85938439 | 114.513476 | Gm44559 |
| 1.324444921 | 0.001176275 | 0.004489264 | 49.6570379 | 68.37525501 | 35.74883576 | 110.2673009 | 69.77161059 | 208.5781171 | Muc6 |
| 1.438956251 | 0.001180227 | 0.004503454 | 14.05387865 | 21.8800816 | 10.51436346 | 31.89550025 | 42.54366499 | 53.16697101 | Gm45204 |
| 1.311364129 | 0.001200998 | 0.004571854 | 25.29698157 | 40.11348294 | 51.52038095 | 72.90400056 | 66.36811739 | 152.6846347 | B230206I08Rik |
| 1.368700347 | 0.001232085 | 0.004678188 | 20.61235535 | 16.4100612 | 9.462927113 | 38.2746003 | 41.69279169 | 40.89767001 | Pate2 |
| 1.934099595 | 0.001243269 | 0.004712299 | 5.62155146 | 4.558350334 | 6.308618075 | 17.31470013 | 21.2718325 | 24.53860201 | Serpini2 |
| 1.399397449 | 0.001262956 | 0.004778463 | 11.24310292 | 11.85171087 | 18.92585423 | 37.36330029 | 32.3331854 | 40.89767001 | Gm13199 |
| 1.838116922 | 0.001266825 | 0.004791224 | 5.62155146 | 9.116700669 | 22.08016326 | 32.80680025 | 30.6314388 | 68.16278335 | Gm38048 |
| 1.420642872 | 0.001279742 | 0.004833999 | 25.29698157 | 19.1450714 | 11.5657998 | 39.1859003 | 40.84191839 | 72.25255035 | Mir1188 |
| 1.513175419 | 0.00128051 | 0.004835391 | 15.92772914 | 10.9400408 | 11.5657998 | 23.69380018 | 44.24541159 | 42.26092568 | Rasgrp4 |
| 1.36964742 | 0.001281085 | 0.004836618 | 15.92772914 | 12.76338094 | 14.72010884 | 30.07290023 | 36.58755189 | 46.35069268 | Gm19385 |
| 3.130000103 | 0.001312254 | 0.004934965 | 0 | 5.470020401 | 1.051436346 | 10.02430008 | 34.034932 | 13.63255667 | Rp1l1 |
| 1.55541342 | 0.001353554 | 0.005069517 | 11.24310292 | 9.116700669 | 17.87441788 | 28.25030022 | 31.4823121 | 53.16697101 | Gm24564 |
| 1.133816023 | 0.001368363 | 0.005121998 | 27.17083206 | 52.87686388 | 35.74883576 | 74.72660058 | 67.21899069 | 114.513476 | Gm42549 |
| 1.054393745 | 0.001392529 | 0.005199333 | 25.29698157 | 31.90845234 | 26.28590865 | 55.58930043 | 51.05239799 | 68.16278335 | 2900037B21Rik |
| 1.107116388 | 0.001417375 | 0.005275755 | 29.98160779 | 22.79175167 | 22.08016326 | 45.56500035 | 55.30676449 | 61.34650502 | Alpk3 |
| 1.11361354 | 0.00143936 | 0.005346234 | 54.34166412 | 47.40684348 | 42.05745383 | 86.57350067 | 69.77161059 | 158.1376574 | Gm37677 |
| 1.780423659 | 0.001456365 | 0.005403149 | 4.684626217 | 12.76338094 | 10.51436346 | 19.13730015 | 34.034932 | 43.62418134 | Mir341 |
| 1.823186147 | 0.001458658 | 0.005408536 | 10.30617768 | 4.558350334 | 6.308618075 | 20.04860015 | 31.4823121 | 23.17534634 | Gm6652 |
| 1.075333529 | 0.001466396 | 0.005433047 | 118.9895059 | 262.5609793 | 89.3720894 | 350.8505027 | 282.4899356 | 361.2627518 | Gm16185 |
| 1.505727362 | 0.00149177 | 0.005513286 | 6.558476704 | 17.32173127 | 16.82298153 | 46.47630036 | 33.1840587 | 35.44464734 | Foxr2 |
| 2.279278456 | 0.001565341 | 0.005749922 | 5.62155146 | 4.558350334 | 5.257181729 | 13.66950011 | 13.6139728 | 49.07720401 | 5730408A14Rik |
| 2.62858852 | 0.001661369 | 0.006057649 | 1.873850487 | 4.558350334 | 3.154309038 | 10.02430008 | 11.0613529 | 39.53441434 | Acot10 |
| 2.365104397 | 0.001663037 | 0.006062584 | 1.873850487 | 7.293360535 | 3.154309038 | 11.84690009 | 34.034932 | 17.72232367 | Tmem114 |
| 1.291842674 | 0.001673729 | 0.006094651 | 31.85545827 | 26.43843194 | 18.92585423 | 47.38760037 | 47.64890479 | 96.79115236 | Gm14372 |
| 2.487300216 | 0.001698104 | 0.006164778 | 0.936925243 | 3.646680267 | 6.308618075 | 9.11300007 | 28.0788189 | 23.17534634 | Mrgprh |
| 1.303456676 | 0.001727747 | 0.006255903 | 60.90014082 | 34.64346254 | 17.87441788 | 76.54920059 | 81.68383679 | 124.0562657 | Ifi209 |
| 1.529340098 | 0.001781982 | 0.006419729 | 18.73850487 | 12.76338094 | 23.13159961 | 25.5164002 | 78.28034359 | 53.16697101 | Gm11762 |
| 1.554364211 | 0.001792887 | 0.006454194 | 15.92772914 | 13.675051 | 8.411490767 | 22.78250018 | 37.43842519 | 53.16697101 | Gm12976 |
| 1.107269646 | 0.001800494 | 0.006476738 | 62.77399131 | 107.5770679 | 59.93187171 | 151.2758012 | 102.9556693 | 245.3860201 | Gm25047 |
| 1.23247097 | 0.001850307 | 0.006632415 | 23.42313108 | 45.58350334 | 27.33734499 | 76.54920059 | 48.49977809 | 103.6074307 | Gm15478 |
| 1.511765666 | 0.001851204 | 0.006634399 | 9.369252434 | 24.61509181 | 16.82298153 | 36.45200028 | 36.58755189 | 73.61580602 | Gm43485 |
| 1.604851944 | 0.001863651 | 0.006671567 | 5.62155146 | 14.58672107 | 9.462927113 | 34.62940027 | 24.6753257 | 31.35488034 | 5930409G06Rik |
| 1.870149985 | 0.001885269 | 0.006730212 | 7.495401947 | 3.646680267 | 8.411490767 | 15.49210012 | 27.2279456 | 28.62836901 | Alpk2 |
| 1.818348285 | 0.00189397 | 0.006751274 | 6.558476704 | 5.470020401 | 7.360054421 | 27.33900021 | 15.3157194 | 25.90185767 | 9330175M20Rik |
| 1.360496141 | 0.001922886 | 0.006839175 | 15.92772914 | 41.93682308 | 15.77154519 | 58.32320045 | 53.60501789 | 79.06882869 | Gm38104 |
| 1.269770588 | 0.001929826 | 0.006860061 | 13.11695341 | 26.43843194 | 15.77154519 | 47.38760037 | 39.14017179 | 47.71394835 | Gm37397 |
| 1.505890213 | 0.001932487 | 0.00686699 | 14.05387865 | 7.293360535 | 22.08016326 | 42.83110033 | 29.7805655 | 50.44045968 | Gabrq |
| 1.007895629 | 0.001942741 | 0.006895598 | 43.09856119 | 41.02515301 | 24.18303596 | 61.96840048 | 75.72772369 | 81.79534002 | 2610020C07Rik |
| 1.243688862 | 0.001943042 | 0.006895598 | 16.86465438 | 14.58672107 | 15.77154519 | 36.45200028 | 34.8858053 | 40.89767001 | Gm19345 |
| 1.829017736 | 0.001980379 | 0.007011327 | 5.62155146 | 9.116700669 | 7.360054421 | 20.04860015 | 39.99104509 | 17.72232367 | 4930461G14Rik |
| 1.40920723 | 0.001993909 | 0.007052755 | 14.05387865 | 13.675051 | 14.72010884 | 27.33900021 | 32.3331854 | 54.53022668 | Itgb1bp2 |
| 2.133883293 | 0.001993693 | 0.007052755 | 5.62155146 | 2.735010201 | 4.205745383 | 11.84690009 | 20.4209592 | 23.17534634 | Gm14009 |
| 2.199778119 | 0.002008496 | 0.00709784 | 3.747700973 | 7.293360535 | 2.102872692 | 20.04860015 | 11.9122262 | 29.99162467 | Gm11750 |
| 1.083340747 | 0.002018526 | 0.007127918 | 131.1695341 | 313.614503 | 114.6065617 | 366.3426028 | 305.4635147 | 516.6738978 | Hgf |
| 2.029331399 | 0.002083946 | 0.00732921 | 3.747700973 | 11.85171087 | 2.102872692 | 24.60510019 | 20.4209592 | 28.62836901 | Gm44043 |
| 1.554383913 | 0.002119414 | 0.00743395 | 10.30617768 | 13.675051 | 9.462927113 | 22.78250018 | 28.0788189 | 49.07720401 | Klf14 |
| 2.365387937 | 0.002133028 | 0.007476261 | 3.747700973 | 3.646680267 | 3.154309038 | 11.84690009 | 11.0613529 | 32.71813601 | Gm42527 |
| 1.14093239 | 0.002146398 | 0.007517658 | 30.91853303 | 49.23018361 | 62.03474441 | 74.72660058 | 85.08732999 | 155.411146 | Gm44167 |
| 1.295644746 | 0.002147104 | 0.007518764 | 21.5492806 | 12.76338094 | 22.08016326 | 36.45200028 | 61.26287759 | 39.53441434 | Gm17733 |
| 1.202497977 | 0.002148661 | 0.007520227 | 33.72930876 | 38.29014281 | 15.77154519 | 54.67800042 | 61.26287759 | 88.61161836 | Rapgef4os3 |
| 1.111228548 | 0.002156591 | 0.007543089 | 21.5492806 | 22.79175167 | 28.38878134 | 42.83110033 | 48.49977809 | 66.79952768 | Mmel1 |
| 1.471445764 | 0.00218358 | 0.007628483 | 9.369252434 | 10.02837074 | 10.51436346 | 26.4277002 | 25.526199 | 31.35488034 | Cpb1 |
| 1.782759326 | 0.002189495 | 0.007643609 | 2.81077573 | 11.85171087 | 9.462927113 | 22.78250018 | 34.034932 | 25.90185767 | Gm15533 |
| 1.025885619 | 0.002319942 | 0.008046557 | 30.91853303 | 65.64024481 | 39.95458114 | 74.72660058 | 96.99955619 | 107.6971977 | Hfm1 |
| 2.220870323 | 0.002346868 | 0.008123872 | 6.558476704 | 16.4100612 | 13.6686725 | 9.11300007 | 114.8678955 | 46.35069268 | Mlph |
| 1.604909996 | 0.002355062 | 0.008145201 | 11.24310292 | 7.293360535 | 8.411490767 | 22.78250018 | 22.1227058 | 38.17115868 | Gm32710 |
| 1.399844531 | 0.002474841 | 0.008511847 | 14.99080389 | 20.96841154 | 24.18303596 | 47.38760037 | 29.7805655 | 83.15859569 | Gm43696 |
| 1.164729132 | 0.002490028 | 0.008559499 | 27.17083206 | 33.73179247 | 21.02872692 | 50.12150039 | 47.64890479 | 88.61161836 | 4931440F15Rik |
| 1.004366111 | 0.002595134 | 0.008862575 | 26.23390681 | 28.26177207 | 45.21176287 | 71.99270056 | 57.85938439 | 69.52603902 | 2310002F09Rik |
| 2.668798525 | 0.002595202 | 0.008862575 | 1.873850487 | 0 | 7.360054421 | 12.7582001 | 16.1665927 | 28.62836901 | Tuba3a |
| 1.214817315 | 0.00260741 | 0.008896385 | 19.67543011 | 18.23340134 | 12.61723615 | 34.62940027 | 39.99104509 | 43.62418134 | Gm47179 |
| 2.631292451 | 0.002623446 | 0.008944767 | 0 | 5.470020401 | 5.257181729 | 32.80680025 | 7.657859699 | 25.90185767 | Theg |
| 1.008236732 | 0.002662245 | 0.009069036 | 24.36005633 | 51.05352374 | 37.85170845 | 82.92830064 | 66.36811739 | 79.06882869 | Gm44616 |
| 2.717894932 | 0.002681425 | 0.009124699 | 3.747700973 | 4.558350334 | 0 | 6.379100049 | 21.2718325 | 28.62836901 | Gm30085 |
| 2.524726097 | 0.002693453 | 0.009160782 | 8.43232719 | 1.823340134 | 2.102872692 | 6.379100049 | 34.034932 | 31.35488034 | Trbc2 |
| 1.869039894 | 0.002703046 | 0.009188545 | 7.495401947 | 7.293360535 | 12.61723615 | 23.69380018 | 16.1665927 | 61.34650502 | Speer4b |
| 1.238062655 | 0.002713592 | 0.009216272 | 13.11695341 | 14.58672107 | 21.02872692 | 36.45200028 | 34.8858053 | 43.62418134 | Spx |
| 1.860058617 | 0.002726622 | 0.009255632 | 8.43232719 | 9.116700669 | 2.102872692 | 23.69380018 | 17.8683393 | 31.35488034 | Insl5 |
| 1.900584808 | 0.002770974 | 0.009381134 | 3.747700973 | 5.470020401 | 6.308618075 | 15.49210012 | 17.017466 | 25.90185767 | Gabre |
| 1.402264747 | 0.00278805 | 0.009430945 | 112.4310292 | 39.20181288 | 21.02872692 | 174.9696014 | 128.4818683 | 154.0478904 | Ccr5 |
| 2.252674111 | 0.002789836 | 0.009435334 | 3.747700973 | 11.85171087 | 6.308618075 | 10.02430008 | 23.8244524 | 72.25255035 | Gm43169 |
| 2.089992523 | 0.002857413 | 0.009638521 | 3.747700973 | 3.646680267 | 5.257181729 | 11.84690009 | 15.3157194 | 27.26511334 | Gm6012 |
| 1.781907754 | 0.002892951 | 0.009749866 | 3.747700973 | 7.293360535 | 8.411490767 | 16.40340013 | 22.1227058 | 28.62836901 | Spdef |
| 2.105147075 | 0.00293256 | 0.009864392 | 5.62155146 | 4.558350334 | 2.102872692 | 15.49210012 | 12.7630995 | 25.90185767 | Gm26682 |
| 1.29682915 | 0.003022992 | 0.010129703 | 19.67543011 | 10.9400408 | 13.6686725 | 31.89550025 | 42.54366499 | 34.08139168 | Gm15867 |
| 1.948647682 | 0.003037843 | 0.010171968 | 3.747700973 | 10.02837074 | 4.205745383 | 12.7582001 | 26.3770723 | 31.35488034 | Dgat2l6 |
| 1.149854744 | 0.003161039 | 0.010519157 | 15.92772914 | 20.96841154 | 23.13159961 | 34.62940027 | 45.94715819 | 53.16697101 | Gm13054 |
| 1.393448213 | 0.003171925 | 0.010546282 | 1551.548203 | 3434.261142 | 1398.41034 | 5755.770844 | 4428.795526 | 6588.614639 | Sacs |
| 2.098879482 | 0.00317174 | 0.010546282 | 9.369252434 | 0.911670067 | 5.257181729 | 17.31470013 | 19.5700859 | 29.99162467 | Cldn23 |
| 1.137737906 | 0.003251906 | 0.010767647 | 23.42313108 | 39.20181288 | 47.31463556 | 61.05710047 | 63.81549749 | 118.603243 | Ubap1l |
| 1.007368879 | 0.003288039 | 0.010877947 | 29.98160779 | 48.31851354 | 47.31463556 | 69.25880053 | 68.06986399 | 117.2399874 | Gm15594 |
| 1.695463481 | 0.00330604 | 0.010926253 | 12.18002816 | 12.76338094 | 7.360054421 | 15.49210012 | 55.30676449 | 34.08139168 | Crhr2 |
| 1.220526399 | 0.003306888 | 0.010927181 | 18.73850487 | 17.32173127 | 22.08016326 | 29.16160023 | 48.49977809 | 58.61999368 | Adgrf2 |
| 1.141287222 | 0.003310343 | 0.010934851 | 14.99080389 | 22.79175167 | 21.02872692 | 35.54070027 | 46.79803149 | 47.71394835 | Ankrd55 |
| 1.26908572 | 0.00340699 | 0.01121601 | 15.92772914 | 48.31851354 | 19.97729057 | 69.25880053 | 56.15763779 | 79.06882869 | Gm45221 |
| 1.274755882 | 0.003421252 | 0.011260695 | 14.99080389 | 15.49839114 | 11.5657998 | 30.98420024 | 29.7805655 | 42.26092568 | Gm37891 |
| 1.997534828 | 0.003441202 | 0.011318631 | 3.747700973 | 3.646680267 | 5.257181729 | 12.7582001 | 16.1665927 | 21.81209067 | Gm44169 |
| 1.254583379 | 0.003442127 | 0.011319744 | 17.80157962 | 11.85171087 | 19.97729057 | 40.09720031 | 29.7805655 | 49.07720401 | Gm42884 |
| 1.223527858 | 0.003509064 | 0.011506507 | 11.24310292 | 17.32173127 | 16.82298153 | 35.54070027 | 34.8858053 | 35.44464734 | G630030J09Rik |
| 1.399737112 | 0.003619029 | 0.011818851 | 14.05387865 | 36.46680267 | 11.5657998 | 58.32320045 | 39.14017179 | 68.16278335 | Gm24966 |
| 1.807154056 | 0.003645242 | 0.011890358 | 5.62155146 | 3.646680267 | 9.462927113 | 14.58080011 | 24.6753257 | 25.90185767 | Gm43240 |
| 1.671665745 | 0.003670779 | 0.011961517 | 15.92772914 | 5.470020401 | 5.257181729 | 21.87120017 | 29.7805655 | 34.08139168 | Gm2694 |
| 1.716807582 | 0.003739553 | 0.012140481 | 14.99080389 | 8.205030602 | 4.205745383 | 17.31470013 | 33.1840587 | 40.89767001 | Thbs4 |
| 1.258389757 | 0.003779774 | 0.012252491 | 13.11695341 | 31.90845234 | 17.87441788 | 42.83110033 | 42.54366499 | 66.79952768 | Gm37233 |
| 1.473806881 | 0.003943364 | 0.012716148 | 8.43232719 | 18.23340134 | 8.411490767 | 32.80680025 | 23.8244524 | 42.26092568 | Gm37446 |
| 2.040741757 | 0.003952565 | 0.012739786 | 0.936925243 | 6.381690468 | 6.308618075 | 15.49210012 | 18.7192126 | 21.81209067 | Cd300lg |
| 1.361144595 | 0.003993247 | 0.012855867 | 8.43232719 | 15.49839114 | 12.61723615 | 35.54070027 | 24.6753257 | 34.08139168 | Gm37786 |
| 1.901661738 | 0.004117214 | 0.013200582 | 7.495401947 | 7.293360535 | 2.102872692 | 12.7582001 | 25.526199 | 25.90185767 | Npsr1 |
| 2.778862435 | 0.004216071 | 0.013454164 | 6.558476704 | 4.558350334 | 7.360054421 | 9.11300007 | 4.254366499 | 114.513476 | 9930022D16Rik |
| 1.235936825 | 0.00422565 | 0.013480271 | 19.67543011 | 20.05674147 | 11.5657998 | 41.00850032 | 29.7805655 | 51.80371535 | Gm26824 |
| 1.631630391 | 0.004358483 | 0.013837626 | 15.92772914 | 6.381690468 | 6.308618075 | 18.22600014 | 33.1840587 | 38.17115868 | Gm9844 |
| 1.425555854 | 0.004363446 | 0.013848946 | 14.05387865 | 18.23340134 | 11.5657998 | 41.91980032 | 20.4209592 | 57.25673801 | Dpp4 |
| 1.821251808 | 0.004458694 | 0.01411627 | 6.558476704 | 5.470020401 | 3.154309038 | 20.04860015 | 18.7192126 | 14.99581234 | Fcrlb |
| 1.462011509 | 0.004496362 | 0.014223849 | 13.11695341 | 10.02837074 | 10.51436346 | 21.87120017 | 24.6753257 | 47.71394835 | Gm42639 |
| 1.946709436 | 0.004516294 | 0.014277531 | 2.81077573 | 6.381690468 | 6.308618075 | 17.31470013 | 11.9122262 | 31.35488034 | Olfr70 |
| 1.123387129 | 0.004538002 | 0.014341455 | 21.5492806 | 19.1450714 | 34.69739941 | 41.91980032 | 49.35065139 | 73.61580602 | Evpl |
| 1.53530315 | 0.004608987 | 0.014548202 | 3.747700973 | 17.32173127 | 12.61723615 | 36.45200028 | 29.7805655 | 31.35488034 | Slco1a1 |
| 1.952015434 | 0.004612645 | 0.014555876 | 2.81077573 | 7.293360535 | 4.205745383 | 16.40340013 | 12.7630995 | 27.26511334 | Gm17235 |
| 1.345260284 | 0.004615039 | 0.014561048 | 17.80157962 | 47.40684348 | 12.61723615 | 56.50060044 | 58.71025769 | 84.52185135 | Gm3257 |
| 1.439100509 | 0.00462657 | 0.014588186 | 14.05387865 | 10.9400408 | 7.360054421 | 20.04860015 | 33.1840587 | 35.44464734 | Gm43327 |
| 4.323645933 | 0.004683765 | 0.014746517 | 14.05387865 | 1.823340134 | 1.051436346 | 294.3499023 | 15.3157194 | 29.99162467 | Chil3 |
| 1.681981188 | 0.004791156 | 0.015035527 | 74.95401947 | 12.76338094 | 9.462927113 | 97.50910075 | 102.104796 | 113.1502204 | Plbd1 |
| 1.856525162 | 0.004861411 | 0.015231211 | 3.747700973 | 8.205030602 | 4.205745383 | 13.66950011 | 27.2279456 | 17.72232367 | Plet1 |
| 1.133124091 | 0.004922258 | 0.015384349 | 17.80157962 | 29.17344214 | 37.85170845 | 40.09720031 | 74.87685039 | 70.88929468 | Malrd1 |
| 1.616667276 | 0.004938127 | 0.015416455 | 9.369252434 | 17.32173127 | 7.360054421 | 22.78250018 | 23.8244524 | 59.98324935 | Has1 |
| 1.003813458 | 0.00499087 | 0.015558442 | 39.35086022 | 27.35010201 | 35.74883576 | 65.61360051 | 47.64890479 | 94.06464102 | Gm45790 |
| 1.61521848 | 0.005075769 | 0.015789917 | 9.369252434 | 17.32173127 | 10.51436346 | 21.87120017 | 24.6753257 | 69.52603902 | Gm37949 |
| 1.757161268 | 0.005125939 | 0.015920301 | 7.495401947 | 9.116700669 | 3.154309038 | 24.60510019 | 13.6139728 | 29.99162467 | Tpo |
| 1.073233456 | 0.005232954 | 0.016223925 | 17.80157962 | 20.05674147 | 32.59452672 | 50.12150039 | 40.84191839 | 57.25673801 | Gm45169 |
| 1.54354005 | 0.005264432 | 0.016305786 | 5.62155146 | 20.96841154 | 15.77154519 | 37.36330029 | 24.6753257 | 62.70976068 | Gm42548 |
| 2.126183397 | 0.005270973 | 0.016319408 | 6.558476704 | 6.381690468 | 1.051436346 | 16.40340013 | 11.0613529 | 35.44464734 | Olfr441 |
| 1.46239123 | 0.005377043 | 0.016593272 | 33.72930876 | 10.9400408 | 7.360054421 | 47.38760037 | 45.09628489 | 51.80371535 | Slamf7 |
| 1.561289765 | 0.00545054 | 0.016795885 | 10.30617768 | 5.470020401 | 13.6686725 | 24.60510019 | 41.69279169 | 19.08557934 | Gm11716 |
| 1.050371088 | 0.0054769 | 0.016869028 | 33.72930876 | 56.52354415 | 50.4689446 | 56.50060044 | 140.3940945 | 94.06464102 | 1700040D17Rik |
| 1.520494423 | 0.005489061 | 0.016903782 | 9.369252434 | 5.470020401 | 9.462927113 | 18.22600014 | 25.526199 | 25.90185767 | Rhd |
| 1.745820666 | 0.00550811 | 0.016948911 | 10.30617768 | 9.116700669 | 6.308618075 | 14.58080011 | 20.4209592 | 53.16697101 | Gm37211 |
| 1.497530736 | 0.005677913 | 0.017400289 | 9.369252434 | 25.52676187 | 8.411490767 | 39.1859003 | 26.3770723 | 58.61999368 | 1700126G02Rik |
| 2.722541155 | 0.005728426 | 0.017537279 | 3.747700973 | 1.823340134 | 4.205745383 | 4.556500035 | 7.657859699 | 53.16697101 | Gm20491 |
| 1.603194818 | 0.005741995 | 0.017562087 | 10.30617768 | 5.470020401 | 5.257181729 | 19.13730015 | 19.5700859 | 25.90185767 | 9330171B17Rik |
| 1.710856251 | 0.005753176 | 0.017585129 | 9.369252434 | 5.470020401 | 9.462927113 | 12.7582001 | 41.69279169 | 24.53860201 | Gm39244 |
| 1.83388154 | 0.005839399 | 0.017786644 | 5.62155146 | 7.293360535 | 3.154309038 | 15.49210012 | 28.0788189 | 13.63255667 | B930018H19Rik |
| 1.803605419 | 0.005876557 | 0.017877233 | 4.684626217 | 12.76338094 | 5.257181729 | 23.69380018 | 13.6139728 | 43.62418134 | Rapgef4os1 |
| 1.759526114 | 0.005880941 | 0.017887747 | 6.558476704 | 10.9400408 | 9.462927113 | 17.31470013 | 17.017466 | 58.61999368 | Gm43281 |
| 1.064310188 | 0.005917955 | 0.017986142 | 59.02629033 | 151.3372311 | 71.49767152 | 164.9453013 | 134.4379814 | 293.0999684 | Gm9801 |
| 2.001808078 | 0.005969642 | 0.018134658 | 11.24310292 | 7.293360535 | 11.5657998 | 9.11300007 | 25.526199 | 87.24836269 | Gm45176 |
| 1.030057887 | 0.006022863 | 0.018276175 | 30.91853303 | 55.61187408 | 30.49165403 | 71.99270056 | 57.85938439 | 111.7869647 | 5830432E09Rik |
| 1.577068065 | 0.006074543 | 0.018424299 | 9.369252434 | 4.558350334 | 14.72010884 | 20.04860015 | 24.6753257 | 40.89767001 | Gm30694 |
| 1.568338091 | 0.006077444 | 0.018430199 | 15.92772914 | 5.470020401 | 8.411490767 | 31.89550025 | 17.8683393 | 39.53441434 | Gm10564 |
| 1.739627391 | 0.006095578 | 0.018470664 | 11.24310292 | 7.293360535 | 4.205745383 | 11.84690009 | 29.7805655 | 35.44464734 | Foxn4 |
| 1.034442281 | 0.006137136 | 0.018584909 | 18.73850487 | 22.79175167 | 27.33734499 | 53.76670042 | 35.7366786 | 51.80371535 | Pcdha8 |
| 1.851897732 | 0.006166429 | 0.018667754 | 5.62155146 | 7.293360535 | 3.154309038 | 14.58080011 | 13.6139728 | 31.35488034 | Gm37983 |
| 1.653611189 | 0.006175347 | 0.018683017 | 14.99080389 | 7.293360535 | 5.257181729 | 14.58080011 | 34.8858053 | 38.17115868 | Fam83c |
| 1.283953094 | 0.006188764 | 0.018717736 | 12.18002816 | 19.1450714 | 9.462927113 | 35.54070027 | 25.526199 | 39.53441434 | Gm6565 |
| 1.100548306 | 0.006192025 | 0.018720769 | 29.98160779 | 20.96841154 | 14.72010884 | 57.41190044 | 45.09628489 | 38.17115868 | Gm12504 |
| 1.059027494 | 0.006198664 | 0.018730048 | 21.5492806 | 18.23340134 | 16.82298153 | 34.62940027 | 39.14017179 | 44.98743701 | Mss51 |
| 1.075951147 | 0.006257878 | 0.018876431 | 26.23390681 | 53.78853394 | 34.69739941 | 90.2187007 | 48.49977809 | 104.9706864 | Gm45853 |
| 1.730249534 | 0.006322933 | 0.019043629 | 7.495401947 | 23.70342174 | 25.2344723 | 44.65370034 | 21.2718325 | 122.69301 | Gm15425 |
| 2.065812056 | 0.006323184 | 0.019043629 | 3.747700973 | 6.381690468 | 2.102872692 | 8.201700063 | 19.5700859 | 24.53860201 | Pla2g4d |
| 1.009954315 | 0.006390816 | 0.019205319 | 34.666234 | 26.43843194 | 26.28590865 | 41.91980032 | 79.13121689 | 54.53022668 | Gm11734 |
| 1.051141724 | 0.006412173 | 0.019263495 | 43.09856119 | 30.99678227 | 38.9031448 | 71.08140055 | 46.79803149 | 118.603243 | Dapk2 |
| 1.66151411 | 0.006482774 | 0.019457403 | 5.62155146 | 8.205030602 | 8.411490767 | 14.58080011 | 18.7192126 | 38.17115868 | Gm37247 |
| 1.675784298 | 0.006481853 | 0.019457403 | 6.558476704 | 10.9400408 | 6.308618075 | 14.58080011 | 40.84191839 | 20.44883501 | Cabcoco1 |
| 1.302797873 | 0.00652584 | 0.01957143 | 12.18002816 | 11.85171087 | 11.5657998 | 27.33900021 | 22.1227058 | 39.53441434 | Adam32 |
| 1.195834151 | 0.006556056 | 0.019655936 | 2376.042417 | 5214.752782 | 2287.925489 | 8148.844663 | 6211.375089 | 8270.872132 | Dzip3 |
| 1.042650168 | 0.006625991 | 0.019837846 | 25.29698157 | 19.1450714 | 21.02872692 | 40.09720031 | 57.00851109 | 36.80790301 | Prss22 |
| 1.919506666 | 0.006658026 | 0.019918294 | 5.62155146 | 4.558350334 | 8.411490767 | 11.84690009 | 14.4648461 | 44.98743701 | Gm17087 |
| 1.220604298 | 0.006664255 | 0.01991981 | 22.48620584 | 12.76338094 | 14.72010884 | 32.80680025 | 30.6314388 | 54.53022668 | Hspb7 |
| 1.513789489 | 0.006716981 | 0.020060427 | 9.369252434 | 4.558350334 | 12.61723615 | 21.87120017 | 21.2718325 | 32.71813601 | 1700029H14Rik |
| 1.696597308 | 0.006784174 | 0.020229765 | 4.684626217 | 6.381690468 | 8.411490767 | 11.84690009 | 22.9735791 | 28.62836901 | Tex35 |
| 1.799064681 | 0.006807783 | 0.020297026 | 4.684626217 | 8.205030602 | 3.154309038 | 14.58080011 | 15.3157194 | 27.26511334 | Gm45698 |
| 1.952868917 | 0.006852996 | 0.020409732 | 1.873850487 | 7.293360535 | 3.154309038 | 17.31470013 | 14.4648461 | 16.359068 | Gm26875 |
| 1.017037249 | 0.006856049 | 0.020412519 | 49.6570379 | 36.46680267 | 31.54309038 | 89.30740069 | 45.94715819 | 104.9706864 | Hes5 |
| 1.482620478 | 0.006929042 | 0.020594861 | 18.73850487 | 20.96841154 | 4.205745383 | 35.54070027 | 30.6314388 | 58.61999368 | Slco1b2 |
| 1.925698966 | 0.00713862 | 0.021139586 | 4.684626217 | 5.470020401 | 7.360054421 | 10.02430008 | 15.3157194 | 42.26092568 | 1700080N15Rik |
| 1.517216414 | 0.007237257 | 0.021369303 | 3.747700973 | 9.116700669 | 15.77154519 | 23.69380018 | 28.9296922 | 28.62836901 | Htr1b |
| 1.12835535 | 0.007267107 | 0.021450871 | 18.73850487 | 42.84849314 | 15.77154519 | 52.85540041 | 58.71025769 | 58.61999368 | Gm13416 |
| 1.171445005 | 0.007537844 | 0.022155056 | 20.61235535 | 20.96841154 | 11.5657998 | 29.16160023 | 39.14017179 | 53.16697101 | Gpr165 |
| 1.551407895 | 0.007546816 | 0.022174665 | 34.666234 | 18.23340134 | 22.08016326 | 150.3645012 | 33.1840587 | 35.44464734 | Nos2 |
| 1.695090011 | 0.007593671 | 0.022285172 | 3.747700973 | 11.85171087 | 12.61723615 | 13.66950011 | 30.6314388 | 47.71394835 | Gm48314 |
| 1.104789761 | 0.007702274 | 0.02255925 | 18.73850487 | 18.23340134 | 16.82298153 | 28.25030022 | 38.28929849 | 50.44045968 | Gm7807 |
| 1.369779329 | 0.008127201 | 0.023599482 | 20.61235535 | 8.205030602 | 15.77154519 | 38.2746003 | 22.1227058 | 55.89348235 | 1700080G11Rik |
| 1.703112172 | 0.00815619 | 0.023665838 | 6.558476704 | 6.381690468 | 3.154309038 | 20.95990016 | 17.8683393 | 13.63255667 | Trat1 |
| 1.856870598 | 0.008177882 | 0.023718071 | 6.558476704 | 1.823340134 | 12.61723615 | 16.40340013 | 18.7192126 | 40.89767001 | Ahrr |
| 1.005477931 | 0.008224677 | 0.023828701 | 29.04468254 | 31.90845234 | 24.18303596 | 64.7023005 | 35.7366786 | 72.25255035 | Gm17096 |
| 1.483980018 | 0.008233013 | 0.023842104 | 5.62155146 | 11.85171087 | 6.308618075 | 20.95990016 | 20.4209592 | 25.90185767 | Nuggc |
| 1.763170149 | 0.008330119 | 0.024101595 | 6.558476704 | 10.9400408 | 2.102872692 | 13.66950011 | 22.9735791 | 31.35488034 | Gm45425 |
| 1.66430204 | 0.00842434 | 0.024352278 | 5.62155146 | 8.205030602 | 8.411490767 | 16.40340013 | 14.4648461 | 40.89767001 | Gm10719 |
| 1.41139573 | 0.008570134 | 0.024696333 | 14.99080389 | 11.85171087 | 5.257181729 | 22.78250018 | 24.6753257 | 39.53441434 | Cyp4f18 |
| 1.500829167 | 0.008660406 | 0.02492258 | 12.18002816 | 4.558350334 | 8.411490767 | 19.13730015 | 21.2718325 | 31.35488034 | Gm45906 |
| 2.204130428 | 0.008784065 | 0.025221974 | 8.43232719 | 0.911670067 | 4.205745383 | 6.379100049 | 26.3770723 | 29.99162467 | 5430421F17Rik |
| 1.363667221 | 0.008791096 | 0.025238403 | 15.92772914 | 9.116700669 | 11.5657998 | 22.78250018 | 23.8244524 | 49.07720401 | Gm10571 |
| 1.004346817 | 0.008793849 | 0.025242549 | 21.5492806 | 30.99678227 | 17.87441788 | 40.09720031 | 44.24541159 | 58.61999368 | Il12rb2 |
| 1.421273088 | 0.008835054 | 0.025349504 | 17.80157962 | 12.76338094 | 30.49165403 | 33.71810026 | 31.4823121 | 99.51766369 | 5330406M23Rik |
| 1.062039961 | 0.008838922 | 0.025356829 | 18.73850487 | 32.82012241 | 26.28590865 | 34.62940027 | 57.00851109 | 72.25255035 | 1110002J07Rik |
| 1.053727045 | 0.008842205 | 0.025358701 | 23.42313108 | 23.70342174 | 23.13159961 | 31.89550025 | 67.21899069 | 46.35069268 | Siglec5 |
| 1.009054223 | 0.008847321 | 0.025368405 | 28.1077573 | 30.99678227 | 21.02872692 | 47.38760037 | 39.99104509 | 76.34231735 | Gm42735 |
| 1.367122546 | 0.0090328 | 0.025820819 | 8.43232719 | 16.4100612 | 10.51436346 | 27.33900021 | 20.4209592 | 44.98743701 | Tek |
| 1.33842176 | 0.009037365 | 0.025826208 | 9.369252434 | 10.9400408 | 9.462927113 | 21.87120017 | 20.4209592 | 34.08139168 | Scn11a |
| 1.445355481 | 0.009124868 | 0.026053099 | 14.05387865 | 11.85171087 | 9.462927113 | 14.58080011 | 34.034932 | 49.07720401 | Prss54 |
| 1.069628627 | 0.00922547 | 0.026292652 | 21.5492806 | 30.99678227 | 26.28590865 | 32.80680025 | 55.30676449 | 79.06882869 | 2900022M07Rik |
| 1.373420876 | 0.009316634 | 0.026506421 | 14.99080389 | 10.9400408 | 7.360054421 | 20.04860015 | 25.526199 | 42.26092568 | Gm26615 |
| 1.225612758 | 0.009344024 | 0.026576506 | 7.495401947 | 15.49839114 | 16.82298153 | 28.25030022 | 33.1840587 | 31.35488034 | Fendrr |
| 1.787306301 | 0.009436677 | 0.026800496 | 14.99080389 | 10.02837074 | 10.51436346 | 20.95990016 | 13.6139728 | 89.97487402 | Gm43175 |
| 1.625705247 | 0.009441998 | 0.02680771 | 14.05387865 | 11.85171087 | 2.102872692 | 25.5164002 | 20.4209592 | 42.26092568 | Gm30292 |
| 1.373468671 | 0.009489325 | 0.026892096 | 11.24310292 | 10.02837074 | 10.51436346 | 15.49210012 | 35.7366786 | 31.35488034 | Mir335 |
| 1.5888245 | 0.009939804 | 0.027907526 | 29.04468254 | 9.116700669 | 3.154309038 | 44.65370034 | 43.39453829 | 36.80790301 | Clec4a3 |
| 1.393782768 | 0.010105333 | 0.028311672 | 10.30617768 | 5.470020401 | 11.5657998 | 24.60510019 | 18.7192126 | 28.62836901 | A330069K06Rik |
| 1.520458504 | 0.010227301 | 0.028586921 | 5.62155146 | 8.205030602 | 13.6686725 | 13.66950011 | 32.3331854 | 32.71813601 | Myrfl |
| 1.482224115 | 0.010295874 | 0.02874942 | 4.684626217 | 10.9400408 | 8.411490767 | 20.95990016 | 17.017466 | 29.99162467 | Srrm3os |
| 1.844331624 | 0.010396538 | 0.028992717 | 5.62155146 | 4.558350334 | 4.205745383 | 15.49210012 | 27.2279456 | 8.179534002 | 1700123K08Rik |
| 1.262701321 | 0.010616171 | 0.029541117 | 11.24310292 | 26.43843194 | 10.51436346 | 31.89550025 | 32.3331854 | 53.16697101 | Gm37776 |
| 1.209762214 | 0.010715938 | 0.029780051 | 25.29698157 | 15.49839114 | 13.6686725 | 32.80680025 | 29.7805655 | 65.43627202 | Slc7a10 |
| 1.047575096 | 0.010774289 | 0.029904814 | 17.80157962 | 21.8800816 | 12.61723615 | 34.62940027 | 34.8858053 | 39.53441434 | Gm16105 |
| 1.063736463 | 0.01083175 | 0.03004128 | 16.86465438 | 33.73179247 | 15.77154519 | 38.2746003 | 48.49977809 | 53.16697101 | Trim43a |
| 1.020323002 | 0.0109684 | 0.030350418 | 59.96321558 | 190.539044 | 58.88043537 | 201.3973016 | 188.0429993 | 239.9329974 | Gm42664 |
| 1.925504258 | 0.011000002 | 0.030424765 | 3.747700973 | 4.558350334 | 6.308618075 | 9.11300007 | 11.9122262 | 35.44464734 | Gm37436 |
| 1.239826976 | 0.01100338 | 0.030429743 | 24.36005633 | 16.4100612 | 19.97729057 | 22.78250018 | 45.94715819 | 76.34231735 | AC117245.2 |
| 1.462960306 | 0.011041023 | 0.03050759 | 6.558476704 | 14.58672107 | 12.61723615 | 34.62940027 | 14.4648461 | 44.98743701 | Gm15379 |
| 1.677222694 | 0.01108329 | 0.030598068 | 3.747700973 | 3.646680267 | 9.462927113 | 17.31470013 | 14.4648461 | 21.81209067 | Gm14041 |
| 1.057701427 | 0.011146831 | 0.030733883 | 20.61235535 | 27.35010201 | 11.5657998 | 45.56500035 | 38.28929849 | 40.89767001 | Gm28800 |
| 1.809704188 | 0.011145847 | 0.030733883 | 4.684626217 | 8.205030602 | 6.308618075 | 27.33900021 | 6.806986399 | 34.08139168 | Sis |
| 1.852462873 | 0.011184622 | 0.03082045 | 8.43232719 | 10.9400408 | 3.154309038 | 10.93560008 | 17.8683393 | 54.53022668 | Gm21846 |
| 1.204762694 | 0.011202847 | 0.030861852 | 14.05387865 | 12.76338094 | 9.462927113 | 23.69380018 | 34.034932 | 25.90185767 | Gm42572 |
| 1.576759762 | 0.011255153 | 0.030970545 | 11.24310292 | 27.35010201 | 15.77154519 | 22.78250018 | 29.7805655 | 111.7869647 | Gm42992 |
| 1.517700198 | 0.011353218 | 0.031200312 | 11.24310292 | 12.76338094 | 16.82298153 | 27.33900021 | 16.1665927 | 74.97906169 | Gm37407 |
| 1.066245158 | 0.011481869 | 0.031517927 | 15.92772914 | 23.70342174 | 15.77154519 | 42.83110033 | 45.09628489 | 27.26511334 | Gm26752 |
| 1.346280825 | 0.011517657 | 0.031593677 | 20.61235535 | 9.116700669 | 6.308618075 | 33.71810026 | 24.6753257 | 34.08139168 | Mefv |
| 1.418652439 | 0.011522894 | 0.031603546 | 9.369252434 | 10.02837074 | 9.462927113 | 13.66950011 | 26.3770723 | 38.17115868 | Fth-ps2 |
| 1.125666152 | 0.01177319 | 0.032180164 | 14.99080389 | 20.05674147 | 13.6686725 | 23.69380018 | 39.99104509 | 43.62418134 | Cda |
| 1.462391211 | 0.011806398 | 0.032252644 | 20.61235535 | 32.82012241 | 41.00601749 | 51.03280039 | 29.7805655 | 181.3130037 | Gm10722 |
| 1.71623798 | 0.011942165 | 0.032565384 | 4.684626217 | 5.470020401 | 14.72010884 | 10.93560008 | 27.2279456 | 43.62418134 | Erg |
| 1.424342189 | 0.012041208 | 0.032791882 | 9.369252434 | 17.32173127 | 10.51436346 | 22.78250018 | 20.4209592 | 58.61999368 | Gm45263 |
| 1.423529311 | 0.01206857 | 0.032852478 | 14.99080389 | 8.205030602 | 14.72010884 | 21.87120017 | 21.2718325 | 59.98324935 | Tex14 |
| 1.051471175 | 0.012470688 | 0.033770693 | 36.54008449 | 24.61509181 | 13.6686725 | 57.41190044 | 39.14017179 | 59.98324935 | Npc1l1 |
| 1.250427372 | 0.012491073 | 0.033797415 | 10.30617768 | 15.49839114 | 7.360054421 | 30.07290023 | 24.6753257 | 24.53860201 | Gm20405 |
| 1.974861845 | 0.012613558 | 0.034076224 | 13.11695341 | 0.911670067 | 6.308618075 | 10.93560008 | 24.6753257 | 44.98743701 | Gm21984 |
| 1.606647157 | 0.012631639 | 0.034108773 | 4.684626217 | 6.381690468 | 5.257181729 | 11.84690009 | 17.8683393 | 20.44883501 | Tmprss13 |
| 1.110568371 | 0.0126799 | 0.034212308 | 15.92772914 | 42.84849314 | 16.82298153 | 61.05710047 | 43.39453829 | 59.98324935 | Gm43328 |
| 1.815750168 | 0.012741505 | 0.034330485 | 1.873850487 | 10.9400408 | 3.154309038 | 18.22600014 | 14.4648461 | 24.53860201 | Spaca5 |
| 1.021530162 | 0.012864478 | 0.034594143 | 19.67543011 | 20.05674147 | 42.05745383 | 45.56500035 | 69.77161059 | 49.07720401 | D430040D24Rik |
| 1.654330043 | 0.013108608 | 0.035152582 | 6.558476704 | 7.293360535 | 2.102872692 | 14.58080011 | 16.1665927 | 20.44883501 | 4930408O17Rik |
| 1.164795277 | 0.013583474 | 0.036199442 | 12.18002816 | 9.116700669 | 23.13159961 | 31.89550025 | 34.034932 | 32.71813601 | Rcvrn |
| 1.360491093 | 0.013647873 | 0.036340926 | 6.558476704 | 18.23340134 | 7.360054421 | 22.78250018 | 25.526199 | 35.44464734 | 4930447C04Rik |
| 1.270692114 | 0.013667096 | 0.036379874 | 27.17083206 | 11.85171087 | 26.28590865 | 33.71810026 | 34.034932 | 91.33812969 | Gm37529 |
| 1.862361467 | 0.013834773 | 0.03676244 | 5.62155146 | 20.96841154 | 7.360054421 | 13.66950011 | 20.4209592 | 91.33812969 | Gm44553 |
| 1.057109078 | 0.013973321 | 0.037064191 | 15.92772914 | 16.4100612 | 13.6686725 | 30.07290023 | 27.2279456 | 39.53441434 | Gm14025 |
| 1.438919952 | 0.014026649 | 0.03718518 | 6.558476704 | 18.23340134 | 5.257181729 | 20.95990016 | 26.3770723 | 35.44464734 | Gm26789 |
| 1.772221808 | 0.014126321 | 0.037387728 | 6.558476704 | 5.470020401 | 5.257181729 | 8.201700063 | 15.3157194 | 36.80790301 | Gm37331 |
| 1.246604544 | 0.014227617 | 0.037609361 | 9.369252434 | 12.76338094 | 14.72010884 | 23.69380018 | 21.2718325 | 43.62418134 | Gm3086 |
| 1.525969506 | 0.014236543 | 0.03762264 | 15.92772914 | 10.02837074 | 14.72010884 | 10.93560008 | 66.36811739 | 39.53441434 | Cryba2 |
| 1.196940941 | 0.014386699 | 0.037977009 | 18.73850487 | 12.76338094 | 18.92585423 | 59.23450046 | 33.1840587 | 21.81209067 | F7 |
| 1.757252459 | 0.014388364 | 0.037977009 | 7.495401947 | 7.293360535 | 1.051436346 | 14.58080011 | 14.4648461 | 25.90185767 | Gfy |
| 1.453138009 | 0.014414469 | 0.038025089 | 4.684626217 | 8.205030602 | 8.411490767 | 14.58080011 | 19.5700859 | 24.53860201 | Gm44027 |
| 1.130050346 | 0.014541474 | 0.038307714 | 14.05387865 | 10.9400408 | 12.61723615 | 25.5164002 | 24.6753257 | 32.71813601 | Fam71d |
| 1.281474339 | 0.01457324 | 0.038370427 | 304.5007041 | 725.6893732 | 255.499032 | 1109.963409 | 725.7949248 | 1291.003117 | BC005561 |
| 1.91066273 | 0.014884253 | 0.039082675 | 2.81077573 | 4.558350334 | 7.360054421 | 30.07290023 | 5.956113099 | 19.08557934 | Gm24265 |
| 1.418205765 | 0.015110833 | 0.039575112 | 10.30617768 | 24.61509181 | 10.51436346 | 37.36330029 | 17.8683393 | 68.16278335 | Alas2 |
| 1.262227167 | 0.015347324 | 0.040117275 | 252.0328905 | 644.5507373 | 222.9045053 | 1021.567308 | 676.4442734 | 988.3603586 | D930016D06Rik |
| 1.479356192 | 0.015563464 | 0.040551235 | 7.495401947 | 3.646680267 | 9.462927113 | 17.31470013 | 22.9735791 | 16.359068 | Gm14061 |
| 1.139008297 | 0.015649397 | 0.040736602 | 14.99080389 | 21.8800816 | 10.51436346 | 27.33900021 | 29.7805655 | 49.07720401 | Lrp8os2 |
| 2.000082502 | 0.016207227 | 0.041956436 | 4.684626217 | 0.911670067 | 8.411490767 | 12.7582001 | 9.359606299 | 34.08139168 | D630014O11Rik |
| 1.669273645 | 0.016250009 | 0.042033327 | 11.24310292 | 13.675051 | 13.6686725 | 11.84690009 | 21.2718325 | 91.33812969 | Gm42993 |
| 2.481512846 | 0.016272437 | 0.042068765 | 8.43232719 | 0 | 2.102872692 | 41.00850032 | 6.806986399 | 10.90604534 | H2-Ea-ps |
| 1.025372047 | 0.016380288 | 0.04231355 | 14.99080389 | 17.32173127 | 14.72010884 | 29.16160023 | 28.0788189 | 39.53441434 | Abca17 |
| 1.324363796 | 0.01644466 | 0.042440033 | 12.18002816 | 9.116700669 | 8.411490767 | 14.58080011 | 34.034932 | 25.90185767 | Mroh5 |
| 1.036693333 | 0.016547144 | 0.042670254 | 18.73850487 | 11.85171087 | 19.97729057 | 27.33900021 | 35.7366786 | 40.89767001 | Gm27002 |
| 1.029287945 | 0.016743429 | 0.04308997 | 15.92772914 | 18.23340134 | 11.5657998 | 30.07290023 | 32.3331854 | 31.35488034 | Nlrp5-ps |
| 1.229501539 | 0.01676036 | 0.043127786 | 7.495401947 | 14.58672107 | 9.462927113 | 21.87120017 | 28.9296922 | 23.17534634 | Gm28988 |
| 1.987638754 | 0.016850951 | 0.043326203 | 7.495401947 | 2.735010201 | 4.205745383 | 9.11300007 | 8.508732999 | 40.89767001 | Mir3070a |
| 1.038729118 | 0.016865066 | 0.043345152 | 2272.98064 | 4790.826201 | 1986.163257 | 6750.910452 | 5485.580164 | 6356.861175 | Pcmtd1 |
| 1.057048228 | 0.01689967 | 0.043416727 | 22.48620584 | 12.76338094 | 27.33734499 | 29.16160023 | 44.24541159 | 57.25673801 | 4930429H19Rik |
| 1.160992314 | 0.016979739 | 0.043610809 | 11.24310292 | 15.49839114 | 22.08016326 | 22.78250018 | 34.034932 | 53.16697101 | Muc5b |
| 1.027246758 | 0.017110159 | 0.043893157 | 13.11695341 | 26.43843194 | 22.08016326 | 43.74240034 | 29.7805655 | 53.16697101 | Gpr21 |
| 1.552562969 | 0.017389817 | 0.044487079 | 10.30617768 | 2.735010201 | 7.360054421 | 27.33900021 | 14.4648461 | 17.72232367 | Myl2 |
| 1.634315743 | 0.017460012 | 0.044621753 | 4.684626217 | 16.4100612 | 12.61723615 | 19.13730015 | 16.1665927 | 70.88929468 | Gm26601 |
| 1.429301469 | 0.017509644 | 0.044715689 | 4.684626217 | 9.116700669 | 6.308618075 | 20.04860015 | 15.3157194 | 19.08557934 | Gm43523 |
| 1.861812188 | 0.017670156 | 0.04505995 | 6.558476704 | 4.558350334 | 9.462927113 | 13.66950011 | 7.657859699 | 54.53022668 | Gm42974 |
| 1.893927664 | 0.017786994 | 0.045286021 | 7.495401947 | 4.558350334 | 2.102872692 | 5.467800042 | 24.6753257 | 23.17534634 | Nox3 |
| 1.122559299 | 0.01779105 | 0.045290367 | 20.61235535 | 10.02837074 | 17.87441788 | 22.78250018 | 40.84191839 | 42.26092568 | Gm31597 |
| 1.59526888 | 0.017908367 | 0.045570968 | 8.43232719 | 3.646680267 | 6.308618075 | 14.58080011 | 11.9122262 | 29.99162467 | Gm37666 |
| 1.158134483 | 0.017928274 | 0.04559857 | 19.67543011 | 12.76338094 | 16.82298153 | 20.04860015 | 36.58755189 | 54.53022668 | 4921507P07Rik |
| 1.042514677 | 0.018010384 | 0.04578223 | 19.67543011 | 16.4100612 | 11.5657998 | 25.5164002 | 36.58755189 | 36.80790301 | Gm37095 |
| 1.659820687 | 0.018220913 | 0.046219894 | 35.60315925 | 6.381690468 | 3.154309038 | 31.89550025 | 45.09628489 | 66.79952768 | Cxcl9 |
| 1.607125759 | 0.018325719 | 0.046473522 | 2.81077573 | 4.558350334 | 11.5657998 | 13.66950011 | 17.8683393 | 25.90185767 | Trhr2 |
| 1.023424379 | 0.01873819 | 0.047332772 | 20.61235535 | 21.8800816 | 31.54309038 | 36.45200028 | 36.58755189 | 79.06882869 | Gm16183 |
| 1.102509622 | 0.018805505 | 0.047477929 | 17.80157962 | 14.58672107 | 15.77154519 | 26.4277002 | 25.526199 | 53.16697101 | 8030445P17Rik |
| 1.68934004 | 0.018912388 | 0.047685334 | 82.44942142 | 107.5770679 | 110.4008163 | 116.6464009 | 205.9113386 | 647.5464418 | Gm37899 |
| 1.503385906 | 0.018916387 | 0.047685792 | 6.558476704 | 4.558350334 | 5.257181729 | 16.40340013 | 16.1665927 | 13.63255667 | Asb15 |
| 1.062532415 | 0.018979501 | 0.04782587 | 19.67543011 | 47.40684348 | 13.6686725 | 60.14580046 | 56.15763779 | 53.16697101 | Olfr1564 |
| 1.510377183 | 0.019080046 | 0.048007616 | 10.30617768 | 2.735010201 | 7.360054421 | 17.31470013 | 15.3157194 | 25.90185767 | A530016L24Rik |
| 1.449828996 | 0.019092985 | 0.048027637 | 2.81077573 | 9.116700669 | 11.5657998 | 16.40340013 | 25.526199 | 21.81209067 | Kbtbd12 |
| 1.738795705 | 0.019133054 | 0.048109601 | 2.81077573 | 14.58672107 | 4.205745383 | 10.93560008 | 22.9735791 | 39.53441434 | 9530003O04Rik |
| 1.224863847 | 0.019463007 | 0.048784162 | 13.11695341 | 10.9400408 | 6.308618075 | 20.04860015 | 28.0788189 | 23.17534634 | Gm29856 |
| 1.010453729 | 0.019497542 | 0.048854075 | 14.05387865 | 28.26177207 | 19.97729057 | 29.16160023 | 44.24541159 | 53.16697101 | Gm44644 |
| 1.117635037 | 0.019735117 | 0.04934675 | 10.30617768 | 14.58672107 | 10.51436346 | 24.60510019 | 22.9735791 | 29.99162467 | Pde6a |
| 1.072876194 | 0.019923983 | 0.049741591 | 13.11695341 | 27.35010201 | 12.61723615 | 28.25030022 | 40.84191839 | 43.62418134 | Gm27616 |
| 1.012739158 | 0.020013797 | 0.049914115 | 14.99080389 | 30.99678227 | 21.02872692 | 33.71810026 | 39.14017179 | 64.07301635 | Gm12224 |

**Table S5.** Differentially expressed genes that are downregulated by rAAV8-sg2 treatment (ranked by adjusted p values)

| **log2FoldChange** | **pvalue** | **padj** | **WT-3** | **WT-2** | **WT-1** | **WT-sg2-3** | **WT-sg2-2** | **WT-sg2-1** | **Gene.name** |
| --- | --- | --- | --- | --- | --- | --- | --- | --- | --- |
| -5.245062405 | 6.91E-165 | 1.33E-160 | 39164.4121 | 60599.62101 | 41323.55127 | 1128.189409 | 1116.345769 | 1479.132399 | Ttr |
| -4.008066708 | 1.39E-92 | 1.34E-88 | 3049.691667 | 4321.316117 | 2975.564859 | 241.4945019 | 170.17466 | 233.1167191 | Ptgds |
| -5.762706206 | 2.80E-90 | 1.80E-86 | 1110.256413 | 1768.63993 | 1145.014181 | 20.95990016 | 17.8683393 | 38.17115868 | Tmem72 |
| -4.497796011 | 4.65E-75 | 2.24E-71 | 772.9633258 | 1231.66626 | 860.0749309 | 42.83110033 | 34.034932 | 51.80371535 | Clic6 |
| -4.245241057 | 2.43E-73 | 9.37E-70 | 915.3759628 | 1226.19624 | 887.4122759 | 38.2746003 | 51.05239799 | 73.61580602 | Mfrp |
| -3.179208235 | 9.60E-59 | 2.31E-55 | 1122.436442 | 1632.80109 | 1129.242635 | 127.582001 | 161.665927 | 139.052078 | Igf2 |
| -4.51288147 | 2.01E-58 | 4.30E-55 | 397.2563032 | 625.4056659 | 436.3460835 | 20.95990016 | 21.2718325 | 21.81209067 | Cldn1 |
| -3.646785183 | 5.59E-55 | 1.08E-51 | 456.2825935 | 497.7718565 | 403.7515568 | 29.16160023 | 47.64890479 | 29.99162467 | Wfdc2 |
| -3.27180904 | 5.37E-51 | 7.40E-48 | 2713.335505 | 4576.583736 | 2598.099211 | 367.2539028 | 342.0510666 | 313.5488034 | Enpp2 |
| -4.788776295 | 1.54E-50 | 1.98E-47 | 300.7530031 | 403.8698396 | 308.0708493 | 17.31470013 | 10.2104796 | 8.179534002 | Krt18 |
| -3.247494197 | 2.24E-45 | 2.16E-42 | 291.3837507 | 331.8479043 | 296.5050495 | 26.4277002 | 36.58755189 | 34.08139168 | Gsta3 |
| -2.169398832 | 7.47E-45 | 6.55E-42 | 3661.503851 | 4593.905467 | 3821.971117 | 972.3571075 | 971.6973085 | 736.1580602 | Vat1l |
| -4.559201922 | 9.23E-43 | 7.12E-40 | 373.8331721 | 430.3082716 | 344.8711214 | 5.467800042 | 25.526199 | 17.72232367 | Krt8 |
| -3.408295577 | 1.25E-41 | 8.93E-39 | 430.0486867 | 708.367642 | 488.9179008 | 48.29890037 | 61.26287759 | 42.26092568 | Cldn2 |
| -1.718831936 | 5.06E-37 | 2.38E-34 | 784.2064287 | 787.6829378 | 798.0401865 | 266.0996021 | 229.735791 | 222.2106737 | Nt5dc2 |
| -1.332156237 | 4.45E-36 | 2.04E-33 | 4396.053242 | 4699.659195 | 4395.003926 | 1742.405613 | 1936.587631 | 1675.441215 | Shroom3 |
| -1.596132668 | 6.19E-36 | 2.78E-33 | 4637.779955 | 4093.3986 | 4665.223067 | 1567.436012 | 1576.668225 | 1281.460327 | Myo5b |
| -2.454602236 | 9.98E-35 | 4.01E-32 | 2071.541713 | 2652.048224 | 2153.341636 | 474.7873037 | 483.2960343 | 291.7367127 | Sfrp1 |
| -2.118947601 | 5.54E-34 | 2.14E-31 | 714.8739607 | 662.7841386 | 645.5819164 | 159.4775012 | 182.0868862 | 119.9664987 | Gm42928 |
| -1.369087095 | 9.91E-32 | 3.19E-29 | 10638.78614 | 10757.70679 | 10574.29533 | 4047.083331 | 4644.917344 | 3679.427045 | Fam107a |
| -1.208737103 | 2.77E-31 | 8.49E-29 | 8952.3207 | 10045.69247 | 8933.003195 | 4143.681132 | 4148.85821 | 3788.487499 | Flnb |
| -4.950016921 | 5.07E-31 | 1.50E-28 | 268.8975448 | 464.9517341 | 225.007378 | 12.7582001 | 3.4034932 | 16.359068 | Slc4a5 |
| -2.205951698 | 3.24E-30 | 8.79E-28 | 2437.879483 | 2219.004943 | 2331.034379 | 530.3766041 | 627.093622 | 351.7199621 | Fbln5 |
| -2.167033979 | 3.65E-30 | 9.77E-28 | 520.9304353 | 558.853751 | 559.364136 | 132.138501 | 141.2449678 | 87.24836269 | Myl9 |
| -1.6179989 | 1.35E-28 | 3.13E-26 | 2091.217143 | 2453.30415 | 2030.323584 | 735.4191057 | 795.5665354 | 606.6487718 | Sulf1 |
| -1.483870293 | 2.30E-28 | 5.29E-26 | 1399.766314 | 1425.851985 | 1347.941395 | 499.3924039 | 559.8746313 | 428.0622794 | Pxdc1 |
| -1.839058473 | 2.62E-28 | 5.94E-26 | 1079.33788 | 1236.224611 | 977.8358017 | 351.7618027 | 313.1213744 | 252.2022984 | Igfbpl1 |
| -2.145648902 | 7.11E-28 | 1.58E-25 | 360.7162187 | 456.7467035 | 353.2826122 | 78.3718006 | 95.29780959 | 91.33812969 | Calml4 |
| -1.430808092 | 7.47E-28 | 1.64E-25 | 241919.7194 | 279672.1147 | 244581.9685 | 99427.38727 | 103624.4557 | 81134.16102 | Gfap |
| -1.615004055 | 1.33E-27 | 2.82E-25 | 2399.465548 | 2642.931524 | 2453.000995 | 810.1457063 | 958.0833357 | 673.4482995 | Ccdc3 |
| -1.566824987 | 1.58E-27 | 3.27E-25 | 4625.599927 | 5378.853394 | 4806.115537 | 1788.881914 | 1836.184581 | 1368.70869 | Pdpn |
| -2.688039506 | 2.03E-27 | 4.11E-25 | 409.4363314 | 636.3457067 | 421.6259747 | 79.28310061 | 90.19256979 | 55.89348235 | Sema3b |
| -1.723332069 | 4.72E-27 | 9.10E-25 | 2588.724447 | 2705.836758 | 2626.487992 | 827.4604064 | 945.3202362 | 620.2813285 | Serping1 |
| -1.83178521 | 4.93E-27 | 9.41E-25 | 601.5060062 | 650.0207577 | 640.3247346 | 203.2199016 | 186.3412527 | 137.6888224 | Pdgfrl |
| -2.741369737 | 6.12E-27 | 1.16E-24 | 207.0604788 | 277.1477003 | 214.4930146 | 40.09720031 | 35.7366786 | 27.26511334 | C1ql1 |
| -1.462164402 | 9.10E-27 | 1.67E-24 | 2090.280218 | 2510.739364 | 2046.095129 | 828.3717064 | 861.0837795 | 719.7989922 | Tnc |
| -1.820753533 | 1.24E-26 | 2.21E-24 | 1400.703239 | 1435.880355 | 1437.313485 | 455.6500035 | 450.1119756 | 298.5529911 | Kank4 |
| -1.510622294 | 2.57E-26 | 4.47E-24 | 2335.754632 | 2375.812194 | 2388.863378 | 864.8237067 | 943.6184896 | 677.5380665 | Islr |
| -2.535863263 | 3.21E-26 | 5.52E-24 | 3851.699675 | 4505.47347 | 4194.179584 | 743.6208057 | 981.0569148 | 434.8785578 | Ace |
| -1.793053592 | 3.35E-26 | 5.67E-24 | 1743.617878 | 1976.500705 | 1751.692952 | 534.0218041 | 632.1988618 | 407.6134444 | Lama5 |
| -2.112432331 | 3.43E-26 | 5.75E-24 | 4073.750958 | 5858.39185 | 4250.957146 | 1231.16631 | 1229.511918 | 815.2268889 | Heg1 |
| -2.447264948 | 4.89E-26 | 8.12E-24 | 295.1314517 | 413.8982104 | 285.9906861 | 64.7023005 | 64.66637079 | 51.80371535 | Otx2 |
| -1.364700376 | 5.11E-26 | 8.42E-24 | 819.8095879 | 851.4998424 | 841.1490767 | 356.3183028 | 318.2266142 | 298.5529911 | Arx |
| -1.488870068 | 6.51E-26 | 1.05E-23 | 783.2695035 | 826.8847506 | 788.5772594 | 269.7448021 | 324.1827273 | 257.6553211 | Ndp |
| -1.354248655 | 8.22E-26 | 1.31E-23 | 5022.85623 | 5353.326633 | 5142.575168 | 2107.836916 | 2243.752892 | 1712.249118 | Cryab |
| -1.980460738 | 1.32E-25 | 2.07E-23 | 487.2011266 | 547.9137102 | 473.1463556 | 134.872401 | 148.0519542 | 95.42789669 | Rbm24 |
| -2.013688242 | 1.48E-25 | 2.28E-23 | 606.1906325 | 787.6829378 | 595.1129718 | 165.8566013 | 191.4464925 | 132.2357997 | Rdh5 |
| -2.308794799 | 1.69E-25 | 2.59E-23 | 688.6400539 | 852.4115125 | 725.4910787 | 162.2114013 | 191.4464925 | 99.51766369 | Folr1 |
| -2.426810728 | 1.90E-25 | 2.88E-23 | 3388.858605 | 4611.227198 | 3351.979071 | 664.3377051 | 958.934209 | 483.9557618 | Id3 |
| -1.825535732 | 2.48E-25 | 3.68E-23 | 449.7241168 | 504.153547 | 459.4776831 | 151.2758012 | 137.8414746 | 106.333942 | Ppp1r1b |
| -1.200323735 | 3.95E-25 | 5.72E-23 | 1334.181547 | 1235.312941 | 1285.906651 | 544.9574042 | 599.8656764 | 530.3064545 | Snx33 |
| -1.585071537 | 4.28E-25 | 6.15E-23 | 37004.79941 | 42018.87338 | 38375.32375 | 14088.69811 | 14754.99389 | 10280.31098 | Prdx6 |
| -1.471797061 | 4.64E-25 | 6.63E-23 | 1689.276214 | 1741.289828 | 1822.139187 | 729.9513056 | 628.7953686 | 530.3064545 | Adrb1 |
| -2.107645579 | 6.13E-25 | 8.69E-23 | 9242.767526 | 14500.11241 | 9265.25708 | 2923.450423 | 2679.400021 | 2053.063035 | A2m |
| -2.073549902 | 6.48E-25 | 9.13E-23 | 283.8883487 | 379.2547478 | 337.511067 | 84.75090065 | 80.83296349 | 70.88929468 | Crybg1 |
| -2.463776531 | 8.65E-25 | 1.21E-22 | 207.997404 | 221.5358262 | 182.9499242 | 33.71810026 | 42.54366499 | 34.08139168 | Dlk1 |
| -3.201780813 | 8.73E-25 | 1.21E-22 | 189.2588992 | 278.0593704 | 164.02407 | 22.78250018 | 19.5700859 | 27.26511334 | St6galnac2 |
| -1.565986479 | 9.29E-25 | 1.27E-22 | 2479.104194 | 2925.549245 | 2500.31563 | 958.6876074 | 983.6095347 | 722.5255035 | Afap1l2 |
| -2.148146203 | 1.11E-24 | 1.50E-22 | 404.7517051 | 588.9388632 | 421.6259747 | 109.3560008 | 115.7187688 | 92.70138536 | Atp10d |
| -1.586428972 | 1.22E-24 | 1.63E-22 | 556.5335946 | 654.579108 | 595.1129718 | 225.0911017 | 187.192126 | 188.129282 | Tcf7 |
| -1.771201722 | 1.61E-24 | 2.10E-22 | 933.1775424 | 1281.808114 | 961.0128201 | 341.7375026 | 313.9722477 | 272.6511334 | P4ha3 |
| -1.828689695 | 1.90E-24 | 2.46E-22 | 312.9330313 | 370.1380471 | 373.2599028 | 95.68650074 | 95.29780959 | 107.6971977 | Fam19a1 |
| -2.140540938 | 2.15E-24 | 2.77E-22 | 445.0394906 | 664.6074787 | 527.8210456 | 123.0255009 | 143.7975877 | 102.244175 | Slc2a12 |
| -1.607076612 | 6.25E-24 | 7.73E-22 | 643.6676422 | 596.2322237 | 558.3126997 | 217.8007017 | 204.209592 | 164.9539357 | Pdgfrb |
| -1.464041855 | 1.71E-23 | 2.06E-21 | 4453.205682 | 5440.846959 | 4310.889018 | 1763.365514 | 1905.105318 | 1476.405887 | Tspan15 |
| -2.149963404 | 1.81E-23 | 2.17E-21 | 604.316782 | 787.6829378 | 620.3474441 | 153.0984012 | 188.8938726 | 107.6971977 | Slco1a5 |
| -1.121584255 | 2.23E-23 | 2.62E-21 | 6210.877438 | 6560.377801 | 6181.394277 | 3195.929125 | 2893.820093 | 2616.087625 | 2810459M11Rik |
| -1.626056589 | 3.15E-23 | 3.60E-21 | 697.0723811 | 879.7616145 | 780.1657686 | 259.720502 | 285.0425555 | 215.3943954 | Glb1l2 |
| -1.8679188 | 3.72E-23 | 4.19E-21 | 2600.904476 | 3147.996741 | 2716.911518 | 898.5418069 | 863.6363994 | 552.1185451 | Lrp2 |
| -1.375131854 | 4.14E-23 | 4.64E-21 | 10922.67449 | 10729.44502 | 11239.85454 | 4511.846335 | 4719.794194 | 3442.220559 | Igfbp3 |
| -1.698378579 | 4.54E-23 | 5.06E-21 | 570.5874732 | 587.1155231 | 529.9239183 | 181.3487014 | 198.2534789 | 136.3255667 | Lcat |
| -1.615309164 | 5.67E-23 | 6.21E-21 | 1567.475932 | 1648.299481 | 1503.553975 | 543.1348042 | 596.4621832 | 395.3441434 | Rab29 |
| -1.949425221 | 7.39E-23 | 8.00E-21 | 4696.806245 | 6517.529308 | 4698.86903 | 1422.539311 | 1657.501188 | 1036.074307 | Padi2 |
| -2.587344459 | 8.51E-23 | 9.07E-21 | 1640.556101 | 3045.889693 | 1658.115117 | 410.0850032 | 392.2525912 | 250.8390427 | Chrdl1 |
| -1.68666445 | 9.42E-23 | 9.98E-21 | 3312.967661 | 4489.975079 | 3294.150072 | 1057.108008 | 1357.142913 | 1030.621284 | Hspb8 |
| -1.653496839 | 1.42E-22 | 1.47E-20 | 1249.858275 | 1610.009338 | 1227.026216 | 465.6743036 | 471.3838081 | 358.5362404 | Stard8 |
| -1.369106774 | 2.60E-22 | 2.64E-20 | 1559.043605 | 1563.514165 | 1633.932081 | 676.1846052 | 655.1724409 | 504.4045968 | Lrrk1 |
| -1.247879653 | 3.05E-22 | 3.08E-20 | 2107.144872 | 2241.796694 | 2047.146565 | 1026.123808 | 834.7067072 | 830.2227012 | Lrp4 |
| -2.288131957 | 4.15E-22 | 4.12E-20 | 3584.675981 | 4494.53343 | 3676.872902 | 904.920907 | 1013.3901 | 483.9557618 | St8sia2 |
| -1.650833998 | 5.22E-22 | 5.11E-20 | 2356.366987 | 2363.048813 | 2352.063106 | 763.6694059 | 915.5396707 | 567.1143575 | Sorbs3 |
| -1.541051962 | 5.75E-22 | 5.60E-20 | 992.2038327 | 1223.46123 | 1058.7964 | 381.8347029 | 427.1383965 | 312.1855477 | Cgnl1 |
| -1.326655589 | 6.24E-22 | 6.02E-20 | 7179.65814 | 8112.040255 | 7222.31626 | 3344.471026 | 3126.108504 | 2500.210893 | Notch2 |
| -2.07299569 | 6.97E-22 | 6.59E-20 | 388.823976 | 361.0213465 | 393.2371934 | 91.1300007 | 114.0170222 | 62.70976068 | Gstm2 |
| -4.580907337 | 7.02E-22 | 6.60E-20 | 100.251001 | 209.6841154 | 121.9666161 | 6.379100049 | 5.105239799 | 6.816278335 | Prr32 |
| -1.078032217 | 7.53E-22 | 7.05E-20 | 1852.301206 | 1976.500705 | 1789.544661 | 896.7192069 | 826.1979742 | 942.0096659 | Metrn |
| -1.46086665 | 9.70E-22 | 8.94E-20 | 978.1499541 | 1060.272288 | 945.2412749 | 367.2539028 | 413.5244237 | 298.5529911 | Gpr146 |
| -2.415716804 | 1.10E-21 | 9.98E-20 | 178.9527215 | 253.4442786 | 212.3901419 | 46.47630036 | 34.8858053 | 39.53441434 | Rasl10a |
| -1.31365814 | 1.11E-21 | 1.01E-19 | 1096.202535 | 1067.565648 | 972.5786199 | 432.8675033 | 459.4715819 | 365.3525188 | Nhsl2 |
| -1.656097691 | 1.30E-21 | 1.18E-19 | 392.571677 | 473.1567647 | 382.7228299 | 143.0741011 | 125.9292484 | 126.782777 | Gm13111 |
| -1.909056079 | 1.35E-21 | 1.21E-19 | 788.8910549 | 1121.354182 | 784.371514 | 242.4058019 | 277.3846958 | 194.9455604 | Prom1 |
| -1.215091384 | 1.40E-21 | 1.25E-19 | 9075.057907 | 10290.93171 | 9489.213022 | 4528.249735 | 4336.050336 | 3559.460547 | Hopx |
| -1.783615899 | 1.50E-21 | 1.33E-19 | 961.2852997 | 1003.748744 | 930.5211661 | 272.4787021 | 353.9632928 | 209.9413727 | Adamtsl4 |
| -2.10795155 | 1.84E-21 | 1.62E-19 | 2442.564109 | 3879.156134 | 2441.435195 | 747.2660058 | 772.5929563 | 509.8576195 | Id4 |
| -2.308516458 | 1.97E-21 | 1.72E-19 | 236.1051613 | 332.7595744 | 229.2131234 | 61.05710047 | 49.35065139 | 50.44045968 | Pcolce2 |
| -2.447521493 | 2.08E-21 | 1.79E-19 | 179.8896467 | 204.214095 | 159.8183246 | 35.54070027 | 27.2279456 | 38.17115868 | Vtn |
| -1.933456171 | 2.08E-21 | 1.79E-19 | 9323.343097 | 11223.57019 | 9666.905764 | 2784.021521 | 3315.85325 | 1804.950503 | 1500015O10Rik |
| -1.460475035 | 2.14E-21 | 1.83E-19 | 825.4311394 | 647.2857475 | 791.7315684 | 291.6160023 | 275.6829492 | 253.5655541 | Lmo1 |
| -1.69523227 | 2.81E-21 | 2.37E-19 | 678.3338762 | 942.6668491 | 673.9706977 | 239.6719019 | 248.4550036 | 219.4841624 | H2-T23 |
| -1.762737082 | 4.05E-21 | 3.35E-19 | 1618.069895 | 1935.475552 | 1631.829209 | 483.9003037 | 645.8128346 | 393.9808878 | Col4a6 |
| -1.245267051 | 5.56E-21 | 4.58E-19 | 2087.469442 | 2338.433721 | 2117.592801 | 961.4215074 | 1005.73224 | 787.9617755 | Tbc1d2b |
| -1.849104469 | 5.74E-21 | 4.71E-19 | 1883.219739 | 2289.203538 | 1957.774476 | 672.5394052 | 628.7953686 | 395.3441434 | Crb2 |
| -1.33022738 | 5.95E-21 | 4.86E-19 | 1455.981828 | 1536.164063 | 1441.51923 | 659.7812051 | 611.7779026 | 486.6822731 | Gas1 |
| -1.628690186 | 6.55E-21 | 5.33E-19 | 4858.894312 | 5381.588405 | 4726.206375 | 1779.768914 | 1861.71078 | 1192.848709 | Palld |
| -1.437324067 | 7.70E-21 | 6.19E-19 | 32134.662 | 38894.58006 | 32746.98499 | 13159.1721 | 14730.31857 | 10424.81609 | Cnn3 |
| -1.276085445 | 9.65E-21 | 7.66E-19 | 3144.321117 | 3869.127764 | 3089.119984 | 1438.942711 | 1477.966922 | 1251.468702 | Tle3 |
| -1.239052045 | 1.13E-20 | 8.90E-19 | 11554.1621 | 12511.76 | 11687.76642 | 5248.176741 | 5662.561811 | 4230.182335 | Plxnb1 |
| -1.277131957 | 1.15E-20 | 9.02E-19 | 1456.918753 | 1565.337505 | 1662.320863 | 657.0473051 | 717.2861918 | 553.4818008 | Tmem98 |
| -1.370181032 | 2.10E-20 | 1.61E-18 | 649.2891937 | 742.0994344 | 639.2732983 | 290.7047022 | 254.4111167 | 238.5697417 | Frem2 |
| -1.799896429 | 2.52E-20 | 1.92E-18 | 3185.545827 | 3722.348883 | 3081.75993 | 1163.730109 | 1027.004073 | 673.4482995 | Rassf4 |
| -1.29034552 | 4.41E-20 | 3.33E-18 | 46163.24367 | 37390.32445 | 47294.65827 | 19310.44715 | 18721.76522 | 15460.68252 | Igfbp2 |
| -2.483028191 | 7.08E-20 | 5.21E-18 | 186.4481234 | 289.9110813 | 236.5731778 | 52.85540041 | 41.69279169 | 31.35488034 | Calca |
| -2.141004342 | 8.80E-20 | 6.41E-18 | 963.1591502 | 1272.691413 | 1058.7964 | 271.5674021 | 315.6739943 | 155.411146 | Id1 |
| -2.394166194 | 1.05E-19 | 7.56E-18 | 1230.182845 | 1549.839114 | 1218.614725 | 351.7618027 | 262.0689764 | 143.141845 | Tcerg1l |
| -1.310014501 | 1.07E-19 | 7.71E-18 | 2978.485349 | 3463.434584 | 2966.101932 | 1278.55391 | 1442.230243 | 1068.792443 | Antxr1 |
| -1.779735639 | 1.17E-19 | 8.32E-18 | 1500.95424 | 1752.229869 | 1491.988175 | 503.0376039 | 552.2167716 | 321.7283374 | Gdpd2 |
| -1.491661612 | 1.49E-19 | 1.06E-17 | 5907.313659 | 7227.72029 | 6004.752971 | 2504.252419 | 2524.541081 | 1772.232367 | Wls |
| -1.549195091 | 1.60E-19 | 1.12E-17 | 1827.94115 | 2652.048224 | 1909.408404 | 803.7666062 | 706.2248389 | 672.0850438 | Ccdc190 |
| -1.822691864 | 1.81E-19 | 1.26E-17 | 718.6216617 | 975.4869715 | 749.6741146 | 267.9222021 | 247.6041303 | 171.770214 | Fam84b |
| -1.764448248 | 1.92E-19 | 1.33E-17 | 1110.256413 | 1072.123999 | 1070.3622 | 356.3183028 | 377.7877451 | 218.1209067 | Nuak2 |
| -1.65452117 | 2.03E-19 | 1.40E-17 | 1533.746623 | 1542.545753 | 1478.319502 | 513.061904 | 581.9973371 | 346.2669394 | Loxl2 |
| -1.879499714 | 2.07E-19 | 1.43E-17 | 902.2590094 | 1086.71072 | 908.4410028 | 292.5273023 | 313.1213744 | 177.2232367 | Cnn2 |
| -1.07363495 | 2.70E-19 | 1.85E-17 | 926.6190657 | 973.6636314 | 927.3668571 | 474.7873037 | 419.4805368 | 449.8743701 | Mid1 |
| -1.454598171 | 3.26E-19 | 2.21E-17 | 1576.845185 | 1417.646954 | 1518.274083 | 555.8930043 | 651.7689477 | 433.5153021 | Dbp |
| -1.057670151 | 3.81E-19 | 2.55E-17 | 1745.491728 | 1847.955226 | 1719.098426 | 857.5333066 | 924.0484037 | 766.1496849 | Irf9 |
| -1.091791822 | 5.61E-19 | 3.70E-17 | 2210.206649 | 2107.781195 | 2183.83329 | 1116.342509 | 1041.468919 | 887.4794392 | Raver2 |
| -1.597011625 | 5.97E-19 | 3.91E-17 | 780.4587277 | 964.5469307 | 846.4062584 | 296.1725023 | 335.2440802 | 220.8474181 | Fxyd1 |
| -1.640109522 | 6.33E-19 | 4.12E-17 | 1647.114578 | 1909.03712 | 1612.903355 | 555.8930043 | 683.2512598 | 414.4297228 | Slc14a1 |
| -1.004865342 | 6.54E-19 | 4.24E-17 | 1420.378669 | 1407.618583 | 1317.449741 | 708.0801055 | 716.4353185 | 638.0036522 | Ramp1 |
| -1.97642704 | 7.66E-19 | 4.94E-17 | 293.2576012 | 271.6776799 | 273.3734499 | 80.19440062 | 81.68383679 | 47.71394835 | Sec14l3 |
| -1.166051285 | 8.34E-19 | 5.36E-17 | 38448.60121 | 35867.83544 | 39661.2304 | 18140.33794 | 18468.20497 | 14177.85894 | Gstm1 |
| -1.990912589 | 9.48E-19 | 6.01E-17 | 1276.092181 | 1252.634672 | 1235.437706 | 403.7059031 | 343.7528132 | 194.9455604 | Dsp |
| -1.356281665 | 9.76E-19 | 6.17E-17 | 83551.2455 | 101008.4851 | 87271.31958 | 37290.39629 | 40331.39441 | 28546.57367 | Vim |
| -1.581180178 | 1.17E-18 | 7.34E-17 | 616.4968101 | 793.1529582 | 615.0902623 | 240.5832019 | 247.6041303 | 185.4027707 | Rfx2 |
| -1.62987208 | 1.36E-18 | 8.45E-17 | 1634.93455 | 1341.978338 | 1562.43441 | 585.9659045 | 514.7783464 | 361.2627518 | Cygb |
| -1.626864568 | 1.58E-18 | 9.77E-17 | 415.9948081 | 516.9169279 | 457.3748105 | 172.2357013 | 154.8589406 | 119.9664987 | Fgd3 |
| -1.29652144 | 1.64E-18 | 1.01E-16 | 1437.243323 | 1406.706913 | 1263.826488 | 564.0947044 | 637.3041016 | 466.2334381 | Caskin2 |
| -1.285789247 | 1.69E-18 | 1.04E-16 | 3591.234458 | 3590.156723 | 3717.878919 | 1628.493113 | 1648.141582 | 1187.395686 | Gldc |
| -1.52402691 | 1.71E-18 | 1.05E-16 | 592.1367538 | 624.4939958 | 537.2839727 | 188.6391015 | 245.0515104 | 173.1334697 | Adamtsl1 |
| -2.248708619 | 1.86E-18 | 1.13E-16 | 492.822678 | 766.7145262 | 500.4837006 | 135.783701 | 149.7537008 | 81.79534002 | P3h2 |
| -1.550478737 | 1.90E-18 | 1.14E-16 | 2436.005633 | 2655.694905 | 2519.241485 | 900.364407 | 1051.679399 | 640.7301635 | Mxra8 |
| -1.081024937 | 2.11E-18 | 1.26E-16 | 3341.075418 | 3541.83821 | 3325.693162 | 1596.597612 | 1807.254889 | 1416.422638 | Fam167a |
| -1.073727644 | 2.28E-18 | 1.36E-16 | 9083.490234 | 9558.860651 | 9191.656536 | 4593.863335 | 4843.170823 | 3780.307965 | Hepacam |
| -1.417011612 | 2.30E-18 | 1.37E-16 | 10881.44978 | 12250.11069 | 10964.37821 | 4366.949634 | 5076.310107 | 3319.527549 | Sdc4 |
| -1.386921841 | 2.45E-18 | 1.45E-16 | 1393.207837 | 1430.410335 | 1469.908012 | 610.5710047 | 601.567423 | 423.9725124 | Parm1 |
| -1.528713267 | 2.54E-18 | 1.50E-16 | 1590.899063 | 1638.27111 | 1678.092408 | 715.3705055 | 536.9010522 | 444.4213474 | Pkp2 |
| -2.220849122 | 2.72E-18 | 1.60E-16 | 966.9068512 | 714.7493324 | 914.7496209 | 262.454402 | 172.7272799 | 118.603243 | Foxo6 |
| -2.016799648 | 2.81E-18 | 1.64E-16 | 798.2603073 | 846.9414921 | 786.4743867 | 220.5346017 | 254.4111167 | 121.3297544 | Galnt15 |
| -1.634896244 | 2.89E-18 | 1.68E-16 | 1124.310292 | 1431.322005 | 1156.57998 | 454.7387035 | 439.0506227 | 297.1897354 | Pawr |
| -3.011083853 | 3.58E-18 | 2.07E-16 | 450.6610421 | 930.8151383 | 425.8317201 | 87.48480068 | 93.59606299 | 40.89767001 | Npr3 |
| -1.672445227 | 3.86E-18 | 2.22E-16 | 1016.563889 | 1263.574713 | 969.4243109 | 410.9963032 | 347.1563064 | 257.6553211 | Cnmd |
| -1.446704705 | 3.97E-18 | 2.27E-16 | 1551.548203 | 1744.936508 | 1560.331537 | 629.7083049 | 690.0582462 | 456.6906484 | Gpc6 |
| -1.247654314 | 3.96E-18 | 2.27E-16 | 8222.455936 | 9314.533073 | 8332.633041 | 4084.446632 | 3834.035089 | 2970.534098 | Tns3 |
| -1.414386837 | 4.58E-18 | 2.62E-16 | 2722.704757 | 2745.950241 | 2701.139973 | 1167.375309 | 1118.898389 | 772.9659632 | Cdc42ep4 |
| -1.362872937 | 4.69E-18 | 2.66E-16 | 3437.578718 | 3970.323141 | 3598.015176 | 1510.935412 | 1637.931102 | 1124.685925 | Sft2d2 |
| -1.248705246 | 5.14E-18 | 2.92E-16 | 3002.845405 | 3250.103788 | 3029.188112 | 1424.361911 | 1423.511031 | 1052.433375 | Myl12a |
| -2.233085975 | 5.30E-18 | 3.00E-16 | 5154.962689 | 5635.944353 | 5438.028781 | 1376.974311 | 1454.14247 | 616.1915615 | Ndrg1 |
| -1.131394051 | 5.72E-18 | 3.22E-16 | 1336.992322 | 1544.369093 | 1397.358904 | 627.8857048 | 724.9440515 | 597.1059821 | Pamr1 |
| -1.170741075 | 6.54E-18 | 3.65E-16 | 1902.895169 | 2200.771541 | 1951.465858 | 981.4701076 | 933.40801 | 770.2394519 | Tst |
| -1.567449816 | 8.25E-18 | 4.56E-16 | 3597.792935 | 3478.021305 | 3609.580975 | 1295.86861 | 1444.782863 | 858.8510702 | Atp2b4 |
| -1.087734548 | 8.79E-18 | 4.84E-16 | 2481.91497 | 2734.098531 | 2511.88143 | 1304.07031 | 1279.713443 | 1046.980352 | Ctdsp1 |
| -1.555827131 | 9.23E-18 | 5.07E-16 | 4173.065034 | 4923.930031 | 4314.043327 | 1633.049613 | 1810.658382 | 1112.416624 | Csf1 |
| -1.610993916 | 1.19E-17 | 6.47E-16 | 279.2037225 | 301.7627921 | 250.2418503 | 98.42040076 | 88.49082319 | 84.52185135 | Cdkn2c |
| -1.1223651 | 1.28E-17 | 6.91E-16 | 2844.505039 | 3076.886476 | 3016.570876 | 1413.426311 | 1524.764953 | 1161.493828 | Oplah |
| -1.200003331 | 1.28E-17 | 6.93E-16 | 4806.426498 | 5173.727629 | 4764.058083 | 2540.70442 | 2007.210114 | 1866.297008 | Itpr1 |
| -1.721166318 | 1.29E-17 | 6.93E-16 | 1295.767612 | 1369.32844 | 1385.793104 | 436.5127034 | 508.8222333 | 278.1041561 | Ucma |
| -1.113059431 | 1.36E-17 | 7.29E-16 | 14024.83397 | 14896.68889 | 14661.22841 | 7044.349054 | 7466.413206 | 5631.60916 | Fat1 |
| -1.127327802 | 1.41E-17 | 7.58E-16 | 47865.63683 | 51205.77265 | 48944.3619 | 23835.96298 | 25100.76235 | 18812.9282 | Pla2g7 |
| -2.403244644 | 1.43E-17 | 7.64E-16 | 601.5060062 | 828.7080908 | 575.1356812 | 152.1871012 | 156.5606872 | 66.79952768 | Pgm5 |
| -2.145376143 | 1.64E-17 | 8.66E-16 | 261.4021429 | 342.7879451 | 310.173722 | 68.34750053 | 90.19256979 | 44.98743701 | Car14 |
| -1.093548387 | 1.69E-17 | 8.86E-16 | 3453.506447 | 3961.206441 | 3477.099996 | 1696.840613 | 1894.894839 | 1507.760768 | Arhgef26 |
| -2.53962166 | 1.72E-17 | 8.98E-16 | 341.0407886 | 546.09037 | 354.3340486 | 93.86390072 | 77.42947029 | 39.53441434 | Cntf |
| -1.387272035 | 1.76E-17 | 9.15E-16 | 913.5021123 | 986.4270123 | 1070.3622 | 354.4957027 | 455.2172154 | 321.7283374 | Serpinf1 |
| -1.554687304 | 1.88E-17 | 9.73E-16 | 2293.592996 | 2952.899347 | 2229.045053 | 883.0497068 | 998.9252541 | 658.4524872 | Naaa |
| -1.180531318 | 1.99E-17 | 1.02E-15 | 591.1998286 | 657.3141182 | 636.1189893 | 266.0996021 | 302.9108948 | 260.3818324 | Tmem63a |
| -1.86604663 | 2.49E-17 | 1.27E-15 | 271.7083206 | 347.3462955 | 268.1162682 | 64.7023005 | 94.44693629 | 84.52185135 | Rcsd1 |
| -1.071831271 | 2.67E-17 | 1.35E-15 | 1768.914859 | 1975.589035 | 1855.78515 | 953.2198074 | 931.7062634 | 774.3292189 | Mettl7a1 |
| -1.293092678 | 2.69E-17 | 1.36E-15 | 1947.867581 | 2193.478181 | 1991.420439 | 882.1384068 | 947.0219828 | 667.9952768 | Bace2 |
| -2.106918285 | 2.90E-17 | 1.45E-15 | 188.3219739 | 232.475867 | 193.4642876 | 55.58930043 | 51.90327129 | 32.71813601 | Ctf1 |
| -1.179468439 | 2.89E-17 | 1.45E-15 | 1260.164452 | 1442.262046 | 1332.16985 | 637.9100049 | 635.602355 | 503.0413411 | Kank2 |
| -1.37420854 | 3.16E-17 | 1.58E-15 | 2081.847891 | 2317.46531 | 2157.547382 | 839.3073065 | 1016.793593 | 667.9952768 | Celsr1 |
| -2.704468583 | 3.30E-17 | 1.64E-15 | 145.2234127 | 170.4823025 | 142.995343 | 25.5164002 | 31.4823121 | 10.90604534 | Rorc |
| -1.535813262 | 3.63E-17 | 1.80E-15 | 2671.173869 | 2732.27519 | 2707.448591 | 1006.075208 | 1118.898389 | 666.6320212 | Pik3ip1 |
| -1.118843897 | 4.48E-17 | 2.21E-15 | 1427.874071 | 1498.78559 | 1391.050286 | 681.6524053 | 734.3036578 | 567.1143575 | Rgs12 |
| -1.146407319 | 4.54E-17 | 2.23E-15 | 2705.840103 | 2600.994701 | 2703.242845 | 1216.585509 | 1381.818239 | 1014.262216 | Igf1r |
| -1.617739434 | 5.17E-17 | 2.53E-15 | 472.2103227 | 608.9956047 | 439.5003926 | 185.9052014 | 168.4729134 | 139.052078 | Fam46c |
| -1.408075967 | 5.65E-17 | 2.74E-15 | 606.1906325 | 677.3708597 | 588.8043537 | 254.252702 | 260.3672298 | 186.7660264 | Atp7b |
| -1.609589717 | 5.98E-17 | 2.89E-15 | 3803.916488 | 4766.21111 | 3860.874262 | 1497.265912 | 1597.940057 | 973.3645462 | Prss23 |
| -1.324392166 | 6.89E-17 | 3.31E-15 | 585.5782771 | 598.9672339 | 618.2445714 | 258.809202 | 262.9198497 | 193.5823047 | Cxcl12 |
| -1.035840095 | 7.09E-17 | 3.40E-15 | 1360.415453 | 1455.937097 | 1322.706923 | 717.1931055 | 700.2687258 | 597.1059821 | Bag3 |
| -1.512483214 | 7.42E-17 | 3.53E-15 | 1530.935848 | 1290.013145 | 1395.256031 | 554.0704043 | 557.3220114 | 361.2627518 | Vwa5b1 |
| -1.109587646 | 7.68E-17 | 3.64E-15 | 1684.591588 | 1860.718606 | 1708.584062 | 839.3073065 | 895.1187115 | 695.2603902 | Efemp2 |
| -1.074648712 | 8.66E-17 | 4.07E-15 | 1618.069895 | 1668.356222 | 1622.366282 | 820.1700063 | 842.3645669 | 662.5422542 | Glis2 |
| -1.100830265 | 1.10E-16 | 5.13E-15 | 915.3759628 | 972.7519613 | 902.1323848 | 436.5127034 | 477.3399212 | 383.0748424 | Syde1 |
| -1.297983203 | 1.14E-16 | 5.32E-15 | 1130.868769 | 1304.599866 | 1213.357543 | 501.2150039 | 574.3394774 | 403.5236774 | Igfbp7 |
| -1.836522395 | 1.28E-16 | 5.86E-15 | 376.6439478 | 499.5951966 | 407.9573022 | 138.5176011 | 134.4379814 | 83.15859569 | Smad9 |
| -1.059260339 | 1.30E-16 | 5.93E-15 | 5163.395016 | 5396.175126 | 5074.231805 | 2717.496621 | 2665.786049 | 2113.046284 | Cpt1a |
| -1.186466892 | 1.69E-16 | 7.61E-15 | 2047.181657 | 2320.20032 | 2033.477893 | 947.7520073 | 1070.398611 | 789.3250312 | Arhgef10 |
| -1.17265177 | 1.70E-16 | 7.63E-15 | 569.650548 | 666.4308189 | 635.0675529 | 292.5273023 | 281.6390623 | 253.5655541 | Fhdc1 |
| -1.089381745 | 1.77E-16 | 7.95E-15 | 3753.322525 | 4733.390987 | 3992.303805 | 2100.546516 | 1980.833042 | 1780.411901 | Gpx1 |
| -1.542898115 | 1.79E-16 | 7.99E-15 | 1655.546905 | 1620.037709 | 1749.59008 | 676.1846052 | 638.1549749 | 404.8869331 | St3gal1 |
| -1.252801932 | 1.90E-16 | 8.45E-15 | 1022.185441 | 1259.016362 | 983.0929834 | 494.8359038 | 466.2785683 | 406.2501888 | Prdm16 |
| -1.184801827 | 1.96E-16 | 8.69E-15 | 5139.971885 | 5211.106102 | 5282.416202 | 2432.259719 | 2595.163565 | 1843.121662 | Tmem176b |
| -1.612951487 | 2.03E-16 | 8.98E-15 | 1060.599375 | 1389.385182 | 982.041547 | 364.5200028 | 454.3663421 | 299.9162467 | Nid2 |
| -1.227820332 | 2.18E-16 | 9.60E-15 | 3827.339619 | 5011.450358 | 3829.331172 | 1900.971815 | 1908.508812 | 1596.372386 | Igsf11 |
| -1.290454251 | 2.21E-16 | 9.69E-15 | 1619.006821 | 2074.961072 | 1589.771755 | 769.1372059 | 767.4877165 | 620.2813285 | Adgrg6 |
| -1.74871218 | 2.47E-16 | 1.08E-14 | 614.6229596 | 667.3424889 | 554.1069543 | 183.1713014 | 232.2884109 | 126.782777 | Prodh |
| -1.09306531 | 2.48E-16 | 1.08E-14 | 1403.514015 | 1624.596059 | 1476.21663 | 720.8383056 | 770.0403364 | 616.1915615 | Ifngr1 |
| -1.011611386 | 3.25E-16 | 1.40E-14 | 63581.62087 | 73920.94403 | 63904.19823 | 34220.22656 | 36223.37812 | 29447.68566 | Slc1a3 |
| -2.40459447 | 3.30E-16 | 1.42E-14 | 200.5020021 | 198.7440746 | 171.3841244 | 44.65370034 | 42.54366499 | 17.72232367 | Atoh8 |
| -2.224968449 | 3.31E-16 | 1.42E-14 | 163.9619176 | 148.6022209 | 134.5838523 | 40.09720031 | 29.7805655 | 24.53860201 | Arsi |
| -1.080784563 | 3.88E-16 | 1.64E-14 | 698.0093063 | 790.417948 | 750.725551 | 342.6488026 | 381.1912383 | 332.6343827 | Fam114a1 |
| -1.018892198 | 4.25E-16 | 1.79E-14 | 7141.244205 | 7458.372817 | 7265.42515 | 3669.805128 | 4030.586822 | 3083.684319 | Cotl1 |
| -1.965501711 | 4.36E-16 | 1.83E-14 | 398.1932284 | 386.5481083 | 428.9860291 | 137.6063011 | 105.5082892 | 64.07301635 | Gdf10 |
| -1.811227779 | 4.73E-16 | 1.98E-14 | 1327.62307 | 1365.68176 | 1401.564649 | 434.6901034 | 485.8486542 | 241.2962531 | Pdlim2 |
| -1.010400052 | 5.00E-16 | 2.08E-14 | 3300.787632 | 3920.181288 | 3588.552248 | 1978.432315 | 1739.185025 | 1645.44959 | Myo1e |
| -1.088929599 | 5.72E-16 | 2.37E-14 | 611.8121839 | 590.7622033 | 580.3928629 | 292.5273023 | 289.296922 | 253.5655541 | Cpt2 |
| -1.20390793 | 5.99E-16 | 2.48E-14 | 6273.65143 | 7197.635178 | 6482.105072 | 2944.410323 | 3353.291675 | 2358.432304 | Sox9 |
| -1.608097767 | 6.76E-16 | 2.78E-14 | 340.1038633 | 448.5416729 | 444.7575743 | 128.493301 | 159.1133071 | 114.513476 | Tns2 |
| -1.055073687 | 6.84E-16 | 2.81E-14 | 891.0159064 | 896.1716757 | 772.8057142 | 418.2867032 | 426.2875232 | 385.8013538 | Tax1bp3 |
| -1.130788786 | 7.76E-16 | 3.16E-14 | 734.5493908 | 746.6577848 | 712.8738425 | 370.8991029 | 292.7004152 | 339.4506611 | Lhx6 |
| -1.143236068 | 8.48E-16 | 3.43E-14 | 10967.6469 | 11701.28531 | 11010.64141 | 5311.056441 | 5796.148919 | 4134.754438 | Ezr |
| -1.607523805 | 1.12E-15 | 4.52E-14 | 499.3811547 | 585.2921829 | 457.3748105 | 173.1470013 | 202.5078454 | 126.782777 | Plekhg3 |
| -1.09684195 | 1.36E-15 | 5.46E-14 | 1012.816188 | 1230.75459 | 1112.419654 | 538.5783042 | 558.1728847 | 468.9599494 | Limd1 |
| -1.338124948 | 1.53E-15 | 6.11E-14 | 5282.384522 | 6106.366108 | 5289.776256 | 2179.829617 | 2681.952641 | 1729.971441 | Lgals3bp |
| -1.017658328 | 1.59E-15 | 6.34E-14 | 1433.495622 | 1559.867484 | 1472.010884 | 774.605006 | 787.9086757 | 638.0036522 | Cib1 |
| -1.84641042 | 1.64E-15 | 6.50E-14 | 10158.14349 | 13943.082 | 10026.49699 | 3823.81483 | 3639.185104 | 2023.07141 | Ahnak |
| -1.260579183 | 1.68E-15 | 6.63E-14 | 634.2983898 | 489.5668259 | 605.6273352 | 246.0510019 | 244.2006371 | 230.3902077 | Adamts8 |
| -1.068380064 | 1.69E-15 | 6.65E-14 | 3710.223964 | 4548.321964 | 3546.494795 | 1987.545315 | 1904.254445 | 1735.424464 | Cdk5rap2 |
| -1.00654126 | 1.69E-15 | 6.65E-14 | 1441.92795 | 1510.637301 | 1509.862593 | 754.5564058 | 815.1366213 | 646.1831862 | Spats2l |
| -1.600318507 | 1.89E-15 | 7.41E-14 | 267.9606196 | 245.239248 | 254.4475957 | 92.95260072 | 91.89431639 | 65.43627202 | Icosl |
| -1.211966408 | 1.91E-15 | 7.47E-14 | 997.8253842 | 921.6984376 | 1065.105018 | 460.2065036 | 471.3838081 | 351.7199621 | Sdk2 |
| -1.250495966 | 2.16E-15 | 8.40E-14 | 3496.605008 | 4491.798419 | 3558.060594 | 1744.228213 | 1760.456857 | 1344.170088 | Sox6 |
| -1.663364393 | 2.32E-15 | 9.00E-14 | 2577.481345 | 3102.413238 | 2624.385119 | 1040.704608 | 998.0743808 | 578.0204028 | Kcnk2 |
| -1.22679325 | 2.51E-15 | 9.67E-14 | 914.4390375 | 869.7332438 | 792.7830048 | 353.5844027 | 428.8401431 | 314.9120591 | Zfp423 |
| -2.147190754 | 2.60E-15 | 9.98E-14 | 894.7636074 | 1196.111128 | 1026.201874 | 308.9307024 | 265.4724696 | 125.4195214 | Traf1 |
| -1.66665528 | 2.96E-15 | 1.13E-13 | 237.9790118 | 245.239248 | 229.2131234 | 59.23450046 | 87.63994989 | 77.70557302 | Rab27a |
| -1.230402495 | 3.20E-15 | 1.21E-13 | 1466.288006 | 1535.252393 | 1577.154519 | 616.9501048 | 787.0578024 | 542.5757555 | Pcsk6 |
| -1.123524304 | 3.21E-15 | 1.21E-13 | 1862.607384 | 2071.314392 | 2014.552039 | 855.7107066 | 1071.249485 | 798.8678209 | Rapgef3 |
| -2.585238929 | 3.31E-15 | 1.24E-13 | 101.1879263 | 128.5454794 | 99.88645286 | 20.95990016 | 20.4209592 | 12.269301 | Ntf3 |
| -1.165894114 | 3.38E-15 | 1.27E-13 | 630.5506888 | 679.1941998 | 621.3988804 | 267.9222021 | 328.4370938 | 261.7450881 | Igtp |
| -1.127325132 | 3.39E-15 | 1.27E-13 | 2192.405069 | 2551.764517 | 2163.856 | 1195.625609 | 1060.188132 | 902.4752516 | Rcn1 |
| -1.688881869 | 3.77E-15 | 1.40E-13 | 1039.050095 | 1086.71072 | 1071.413636 | 375.4556029 | 399.9104509 | 211.3046284 | Wnt9a |
| -1.232481032 | 3.79E-15 | 1.40E-13 | 2242.062107 | 2693.073378 | 2300.542725 | 1171.931809 | 1071.249485 | 831.5859569 | Hacd4 |
| -1.021723212 | 3.85E-15 | 1.42E-13 | 2308.5838 | 2458.77417 | 2198.553399 | 1235.72281 | 1209.941832 | 980.1808246 | Frmd8 |
| -1.139541544 | 3.94E-15 | 1.45E-13 | 2564.364391 | 3157.113442 | 2692.728482 | 1386.998611 | 1349.485054 | 1078.335233 | Rdh10 |
| -1.124349445 | 4.14E-15 | 1.51E-13 | 4574.069038 | 4298.524365 | 4684.148921 | 2214.459017 | 2316.927996 | 1680.894237 | Fgfrl1 |
| -1.542240665 | 4.51E-15 | 1.64E-13 | 874.1512521 | 1075.770679 | 871.6407307 | 337.1810026 | 391.4017179 | 235.8432304 | Steap3 |
| -1.871610015 | 5.40E-15 | 1.96E-13 | 901.3220841 | 1086.71072 | 937.8812205 | 316.2211024 | 320.7792341 | 158.1376574 | Ajuba |
| -1.16034124 | 5.87E-15 | 2.12E-13 | 1426.937146 | 1796.901702 | 1396.307467 | 727.2174056 | 722.3914316 | 614.8283058 | Pax6 |
| -1.140434753 | 6.13E-15 | 2.20E-13 | 1013.753113 | 1204.316158 | 1011.481765 | 539.4896042 | 496.9100071 | 425.3357681 | Rai14 |
| -1.350324051 | 6.63E-15 | 2.36E-13 | 791.7018306 | 878.8499445 | 720.2338969 | 328.9793025 | 359.0685326 | 245.3860201 | Vamp8 |
| -1.35198463 | 6.89E-15 | 2.45E-13 | 614.6229596 | 523.2986184 | 602.4730262 | 256.986602 | 183.7886328 | 242.6595087 | Nos1 |
| -1.174784125 | 6.90E-15 | 2.45E-13 | 1024.059291 | 1203.404488 | 1071.413636 | 478.4325037 | 561.5763779 | 417.1562341 | Slc22a4 |
| -1.049448144 | 6.93E-15 | 2.45E-13 | 2197.089696 | 2628.344803 | 2396.223432 | 1242.10191 | 1236.318905 | 1006.082682 | Gsn |
| -1.957226641 | 8.04E-15 | 2.82E-13 | 56356.05339 | 56085.03084 | 56277.07898 | 15442.88992 | 19847.47059 | 8153.632144 | Igfbp5 |
| -1.673991587 | 8.53E-15 | 2.99E-13 | 1147.733423 | 1417.646954 | 1148.16849 | 408.2624032 | 489.2521474 | 261.7450881 | Adamtsl3 |
| -2.207361271 | 8.56E-15 | 3.00E-13 | 266.0867691 | 381.989758 | 258.6533411 | 89.30740069 | 63.81549749 | 40.89767001 | Ccbe1 |
| -1.157910968 | 9.08E-15 | 3.17E-13 | 36624.40776 | 39137.0843 | 37903.22883 | 17823.20554 | 19627.94528 | 13482.59855 | Mlc1 |
| -3.321218815 | 9.10E-15 | 3.17E-13 | 107.746403 | 178.6873331 | 80.96059863 | 8.201700063 | 11.9122262 | 17.72232367 | Trpv4 |
| -1.180646494 | 9.92E-15 | 3.43E-13 | 1602.142166 | 1913.59547 | 1653.909372 | 848.4203065 | 803.2243951 | 624.3710955 | Rftn2 |
| -1.640807565 | 1.01E-14 | 3.49E-13 | 347.5992653 | 313.614503 | 291.2478678 | 129.404601 | 91.04344309 | 83.15859569 | Pth2r |
| -2.186117607 | 1.02E-14 | 3.49E-13 | 1599.33139 | 1440.438706 | 1483.576684 | 446.5370034 | 381.1912383 | 162.2274244 | Lypd1 |
| -1.349345507 | 1.04E-14 | 3.56E-13 | 778.5848772 | 1059.360618 | 784.371514 | 350.8505027 | 376.0859985 | 299.9162467 | Stard13 |
| -1.184297233 | 1.12E-14 | 3.80E-13 | 718.6216617 | 738.4527542 | 678.1764431 | 367.2539028 | 296.9547817 | 272.6511334 | Adra1a |
| -2.273612684 | 1.13E-14 | 3.81E-13 | 140.5387865 | 161.3656018 | 106.1950709 | 25.5164002 | 31.4823121 | 27.26511334 | Gm4524 |
| -1.403724989 | 1.16E-14 | 3.93E-13 | 1146.796498 | 1399.413553 | 1085.082309 | 517.618404 | 500.3135003 | 350.3567064 | Plscr2 |
| -1.237714206 | 1.22E-14 | 4.10E-13 | 564.9659218 | 604.4372543 | 586.701481 | 261.543102 | 278.2355691 | 200.398583 | Copz2 |
| -1.341758826 | 1.22E-14 | 4.12E-13 | 16201.31131 | 19058.46275 | 16308.82916 | 7115.430455 | 8111.375168 | 5113.572007 | S1pr3 |
| -1.21317846 | 1.23E-14 | 4.15E-13 | 1126.184143 | 1266.309723 | 1067.207891 | 520.352304 | 562.4272512 | 404.8869331 | Mdfic |
| -1.256456132 | 1.53E-14 | 5.09E-13 | 557.4705198 | 711.1026522 | 574.0842448 | 236.9380018 | 284.1916822 | 249.4757871 | Angptl2 |
| -1.176535703 | 1.54E-14 | 5.13E-13 | 1797.959542 | 2057.639341 | 1784.287479 | 855.7107066 | 955.5307158 | 678.9013222 | Tor3a |
| -2.994113896 | 1.67E-14 | 5.54E-13 | 84.3232719 | 93.90201689 | 67.29192614 | 10.93560008 | 10.2104796 | 9.542789669 | F2rl1 |
| -1.324033714 | 1.69E-14 | 5.58E-13 | 1400.703239 | 1661.062862 | 1435.210612 | 667.0716051 | 668.7864137 | 455.3273928 | Anxa4 |
| -1.22189811 | 1.69E-14 | 5.58E-13 | 11960.78766 | 14025.13231 | 11717.20664 | 5733.899644 | 6164.577058 | 4260.173959 | Msn |
| -1.438736472 | 1.77E-14 | 5.81E-13 | 7358.610861 | 7678.996973 | 7342.180003 | 2994.531823 | 3327.765476 | 1927.643513 | Lamb2 |
| -1.066577637 | 2.03E-14 | 6.62E-13 | 1326.686145 | 1506.07895 | 1318.501178 | 667.0716051 | 739.4088976 | 571.2041245 | Scube3 |
| -1.177291741 | 2.04E-14 | 6.62E-13 | 13131.00729 | 14607.68948 | 13910.50286 | 6041.007747 | 7389.834609 | 4979.972952 | Mt2 |
| -1.706455799 | 2.13E-14 | 6.85E-13 | 391.6347517 | 459.4817137 | 423.7288474 | 120.2916009 | 172.7272799 | 94.06464102 | Fbln7 |
| -1.229666911 | 2.34E-14 | 7.54E-13 | 4522.53815 | 5421.701888 | 4981.705407 | 2342.952318 | 2361.173407 | 1654.99238 | Anxa2 |
| -1.22310793 | 2.35E-14 | 7.54E-13 | 4122.471071 | 4703.305875 | 4061.698604 | 1918.286515 | 2147.604209 | 1449.140774 | Ifitm3 |
| -1.464311529 | 2.44E-14 | 7.83E-13 | 12841.49739 | 15172.01325 | 13255.45801 | 5275.515741 | 6145.857845 | 3529.468922 | Timp3 |
| -1.164985104 | 2.48E-14 | 7.92E-13 | 1062.473226 | 1368.41677 | 1070.3622 | 520.352304 | 565.8307444 | 473.0497165 | Cd59a |
| -1.474642835 | 2.52E-14 | 8.04E-13 | 1247.984424 | 1372.063451 | 1299.575323 | 480.2551037 | 589.6551968 | 335.3608941 | Rcn3 |
| -1.372588856 | 2.60E-14 | 8.27E-13 | 3716.78244 | 3850.894362 | 3842.999844 | 1512.758012 | 1814.061875 | 1074.245466 | Man2b2 |
| -1.19653031 | 3.10E-14 | 9.73E-13 | 4826.101929 | 5621.357632 | 4928.082153 | 2342.952318 | 2586.654832 | 1773.595623 | Nfe2l2 |
| -1.350528201 | 3.26E-14 | 1.02E-12 | 6088.140231 | 5492.812153 | 6229.760349 | 2635.47962 | 2651.321202 | 1691.800283 | Spon1 |
| -1.059662107 | 3.64E-14 | 1.14E-12 | 18877.1698 | 19683.86841 | 18779.70457 | 9815.612376 | 10246.21628 | 7440.649431 | Ddr1 |
| -1.31768456 | 3.71E-14 | 1.16E-12 | 1005.320786 | 1051.155587 | 971.5271836 | 364.5200028 | 509.6731066 | 336.7241497 | Perp |
| -1.163642531 | 4.00E-14 | 1.24E-12 | 2708.650879 | 2817.060507 | 2764.226153 | 1336.87711 | 1393.730465 | 963.8217566 | Zfp703 |
| -1.66164834 | 4.13E-14 | 1.28E-12 | 893.8266822 | 702.8976216 | 794.8858775 | 327.1567025 | 251.8584968 | 173.1334697 | Rerg |
| -1.212721241 | 4.39E-14 | 1.35E-12 | 967.8437764 | 1054.802267 | 952.6013294 | 462.0291036 | 479.8925411 | 336.7241497 | Fndc1 |
| -2.004274738 | 5.11E-14 | 1.56E-12 | 1105.571787 | 1506.990621 | 1285.906651 | 355.4070027 | 432.2436363 | 179.949748 | Slc24a4 |
| -1.084918197 | 5.12E-14 | 1.56E-12 | 1263.912153 | 1309.158216 | 1234.38627 | 633.3535049 | 665.3829205 | 490.7720401 | Plin3 |
| -1.210239163 | 6.22E-14 | 1.89E-12 | 523.741211 | 546.09037 | 568.8270631 | 254.252702 | 258.6654832 | 190.8557934 | Msx1 |
| -1.759539864 | 6.28E-14 | 1.90E-12 | 1300.452238 | 1899.920419 | 1277.49516 | 503.9489039 | 519.0327129 | 295.8264797 | Bst2 |
| -1.160767143 | 6.76E-14 | 2.03E-12 | 751.4140452 | 861.5282132 | 709.7195335 | 348.1166027 | 388.849098 | 298.5529911 | Car5b |
| -1.12878612 | 7.02E-14 | 2.10E-12 | 2695.533925 | 2696.720058 | 2708.500027 | 1273.99741 | 1438.82675 | 985.6338472 | Cebpd |
| -4.684717182 | 7.04E-14 | 2.11E-12 | 58.08936509 | 87.52032642 | 57.82899902 | 3.645200028 | 2.5526199 | 1.363255667 | Pon1 |
| -1.292061881 | 7.19E-14 | 2.15E-12 | 704.567783 | 890.7016553 | 800.1430592 | 328.0680025 | 380.340365 | 265.8348551 | Usp18 |
| -1.042567093 | 7.44E-14 | 2.22E-12 | 780.4587277 | 773.0962167 | 833.7890223 | 347.2053027 | 436.4980028 | 373.5320528 | Aard |
| -1.189370581 | 7.58E-14 | 2.25E-12 | 5513.805057 | 7455.637807 | 5616.77296 | 2614.51972 | 3098.029685 | 2434.774621 | Slc16a1 |
| -1.221820193 | 7.66E-14 | 2.27E-12 | 3732.71017 | 4804.501252 | 3624.301084 | 2003.037415 | 1738.334152 | 1469.589609 | Slc25a18 |
| -1.235512502 | 8.00E-14 | 2.36E-12 | 773.900251 | 965.4586008 | 877.9493488 | 373.6330029 | 427.1383965 | 306.7325251 | Tsku |
| -1.212282977 | 7.99E-14 | 2.36E-12 | 1311.695341 | 1529.782372 | 1427.850558 | 664.3377051 | 691.7599928 | 481.2292505 | Zic2 |
| -1.732744513 | 9.72E-14 | 2.85E-12 | 909.7544113 | 1300.041515 | 907.3895665 | 340.8262026 | 382.0421116 | 211.3046284 | Col8a1 |
| -1.308720787 | 9.89E-14 | 2.89E-12 | 2626.201457 | 2256.383415 | 2723.220136 | 1091.737408 | 1201.433099 | 771.6027075 | Adra2a |
| -2.039898498 | 1.02E-13 | 2.98E-12 | 2138.063405 | 1857.071926 | 2185.936163 | 707.1688055 | 527.5414459 | 264.4715994 | Hrk |
| -1.755515274 | 1.08E-13 | 3.15E-12 | 1534.683549 | 1198.846138 | 1620.263409 | 534.9331041 | 481.5942877 | 268.5613664 | Cacna1g |
| -1.046310492 | 1.12E-13 | 3.25E-12 | 2020.010825 | 2350.285432 | 2150.187327 | 1155.528409 | 1114.644023 | 882.0264166 | Nde1 |
| -1.300598111 | 1.14E-13 | 3.30E-12 | 579.9567256 | 666.4308189 | 578.2899902 | 280.6804022 | 263.770723 | 192.219049 | Tmem107 |
| -1.021522498 | 1.15E-13 | 3.32E-12 | 7241.495206 | 6784.648638 | 7413.677675 | 3900.36403 | 3754.903872 | 2899.644804 | Crip2 |
| -1.449878672 | 1.23E-13 | 3.54E-12 | 401.0040042 | 407.5165199 | 427.9345928 | 133.961101 | 191.4464925 | 124.0562657 | Mia |
| -1.062022968 | 1.24E-13 | 3.59E-12 | 1234.867471 | 1499.69726 | 1260.672179 | 658.8699051 | 693.4617394 | 557.5715678 | St6gal1 |
| -1.136818028 | 1.28E-13 | 3.67E-12 | 2308.5838 | 2631.079813 | 2236.405108 | 1050.728908 | 1290.774796 | 917.4710639 | Stat1 |
| -1.04766448 | 1.31E-13 | 3.76E-12 | 1952.552207 | 2034.847589 | 1906.254095 | 986.0266076 | 1075.503851 | 783.8720085 | Wasf2 |
| -2.222887359 | 1.51E-13 | 4.33E-12 | 447.8502663 | 466.7750742 | 489.9693372 | 134.872401 | 117.4205154 | 44.98743701 | Capn3 |
| -1.259579119 | 1.56E-13 | 4.45E-12 | 1345.424649 | 1554.397464 | 1431.004867 | 621.5066048 | 717.2861918 | 464.8701824 | Ehd2 |
| -1.23129105 | 1.59E-13 | 4.53E-12 | 1095.265609 | 1173.319376 | 1020.944692 | 503.9489039 | 530.9449391 | 361.2627518 | Parp12 |
| -1.689813644 | 1.61E-13 | 4.57E-12 | 1491.584987 | 1381.180151 | 1579.257392 | 625.1518048 | 453.5154688 | 297.1897354 | Cadps2 |
| -1.214260918 | 1.63E-13 | 4.61E-12 | 7159.045785 | 7522.189722 | 7433.654965 | 3410.995926 | 3714.912827 | 2399.329974 | Fkbp9 |
| -1.714071626 | 1.68E-13 | 4.73E-12 | 2466.924166 | 1756.788219 | 2312.108525 | 842.0412065 | 716.4353185 | 429.4255351 | Fndc5 |
| -1.24915633 | 1.68E-13 | 4.73E-12 | 3436.641793 | 3503.548067 | 3325.693162 | 1461.725211 | 1755.351618 | 1096.057556 | Hspg2 |
| -1.064515689 | 1.68E-13 | 4.73E-12 | 13507.65123 | 17087.43206 | 14332.12883 | 7771.56646 | 7619.5704 | 6085.573298 | Cd63 |
| -1.049961871 | 1.69E-13 | 4.76E-12 | 1398.829388 | 1421.293634 | 1453.08503 | 786.4519061 | 701.1195991 | 571.2041245 | Ptprm |
| -1.535684727 | 1.84E-13 | 5.13E-12 | 1782.968738 | 2039.40594 | 1765.361625 | 636.0874049 | 840.6628203 | 445.7846031 | Gm19935 |
| -1.435022476 | 1.94E-13 | 5.39E-12 | 710.1893345 | 815.9447098 | 768.5999688 | 289.7934022 | 349.7089263 | 204.4883501 | Fbln1 |
| -1.29474143 | 1.98E-13 | 5.48E-12 | 935.9883181 | 1001.013733 | 954.7042021 | 448.3596035 | 434.7962562 | 290.3734571 | Trip6 |
| -1.102641079 | 2.08E-13 | 5.75E-12 | 1112.130264 | 1299.129845 | 1201.791743 | 565.9173044 | 641.5584681 | 470.3232051 | Spag6 |
| -1.267031914 | 2.33E-13 | 6.40E-12 | 490.9488275 | 537.8853394 | 472.0949193 | 210.5103016 | 241.6480172 | 167.680447 | Erbb2 |
| -1.899042038 | 2.51E-13 | 6.86E-12 | 1381.964734 | 1052.067257 | 1297.472451 | 435.6014034 | 372.6825053 | 188.129282 | Grm2 |
| -1.079241922 | 2.54E-13 | 6.93E-12 | 1515.008119 | 1629.154409 | 1669.680917 | 747.2660058 | 889.1625984 | 636.6403965 | Il11ra1 |
| -1.165555149 | 2.54E-13 | 6.94E-12 | 10468.26574 | 12950.2733 | 10842.4116 | 5317.435541 | 5858.26267 | 4092.493512 | Anxa5 |
| -1.631023852 | 2.56E-13 | 6.96E-12 | 1016.563889 | 1471.435488 | 1052.487782 | 386.391203 | 479.0416678 | 274.0143891 | Bicc1 |
| -1.006593641 | 2.57E-13 | 7.00E-12 | 3246.445968 | 4231.97245 | 3254.19549 | 1791.615814 | 1825.123228 | 1724.518419 | Rnf213 |
| -1.693941889 | 2.60E-13 | 7.06E-12 | 1093.391759 | 1563.514165 | 1189.174507 | 440.1579034 | 484.1469076 | 260.3818324 | Car12 |
| -1.475211909 | 2.65E-13 | 7.17E-12 | 635.235315 | 850.5881724 | 591.9586627 | 249.6962019 | 294.4021618 | 200.398583 | Rtp4 |
| -1.404061328 | 2.93E-13 | 7.87E-12 | 631.487614 | 733.8944038 | 552.0040816 | 268.8335021 | 268.0250895 | 184.039515 | Bcar3 |
| -1.073770991 | 3.05E-13 | 8.15E-12 | 757.9725219 | 837.8247914 | 872.6921671 | 364.5200028 | 449.2611023 | 355.8097291 | Scd4 |
| -2.116144218 | 3.25E-13 | 8.66E-12 | 228.6097594 | 279.8827105 | 220.8016326 | 69.25880053 | 67.21899069 | 28.62836901 | Map3k7cl |
| -1.066149667 | 3.57E-13 | 9.47E-12 | 602.4429315 | 719.3076828 | 610.884517 | 303.4629023 | 332.6914603 | 284.9204344 | Pgghg |
| -1.617434421 | 3.68E-13 | 9.75E-12 | 1470.972632 | 1506.990621 | 1483.576684 | 629.7083049 | 513.0765998 | 306.7325251 | Frzb |
| -1.226197658 | 3.79E-13 | 1.00E-11 | 3142.447266 | 4127.130393 | 3221.600964 | 1645.807813 | 1631.974989 | 1202.391498 | Ifi27 |
| -1.134730788 | 3.99E-13 | 1.05E-11 | 3530.334317 | 3925.651308 | 3340.413271 | 1668.590313 | 1922.122784 | 1320.994741 | Akap12 |
| -1.20123679 | 4.12E-13 | 1.08E-11 | 3265.184473 | 3100.589897 | 3234.2182 | 1551.032612 | 1572.413858 | 1045.617097 | Sv2c |
| -1.059892374 | 4.19E-13 | 1.10E-11 | 2016.263124 | 2484.300932 | 1929.385695 | 984.2040076 | 1150.380701 | 947.4626886 | Irgm1 |
| -1.052299675 | 4.23E-13 | 1.10E-11 | 8810.844989 | 9722.961263 | 9190.605099 | 4538.274035 | 5181.818396 | 3642.619142 | Fstl1 |
| -1.347568955 | 4.25E-13 | 1.11E-11 | 1748.302504 | 2023.907548 | 1790.596097 | 803.7666062 | 853.4259198 | 523.4901761 | Kirrel |
| -2.105602433 | 4.96E-13 | 1.28E-11 | 254.8436662 | 309.0561527 | 236.5731778 | 51.9441004 | 91.89431639 | 39.53441434 | Gprc5c |
| -1.474286976 | 5.07E-13 | 1.30E-11 | 853.5388967 | 1053.890597 | 900.0295121 | 366.3426028 | 405.0156907 | 234.4799747 | Fzd6 |
| -1.07490242 | 5.12E-13 | 1.31E-11 | 619.3075859 | 682.8408801 | 733.9025694 | 363.6087028 | 301.2091482 | 299.9162467 | BC064078 |
| -1.832937066 | 5.21E-13 | 1.33E-11 | 6039.420119 | 5948.647186 | 6250.789076 | 1869.076314 | 2298.208783 | 947.4626886 | Ctgf |
| -1.315889426 | 5.43E-13 | 1.39E-11 | 2809.838805 | 3626.623526 | 3028.136676 | 1302.24771 | 1528.168447 | 966.5482679 | Col4a5 |
| -1.120033582 | 5.77E-13 | 1.47E-11 | 1521.566595 | 1938.210562 | 1517.222647 | 842.0412065 | 786.2069291 | 658.4524872 | Jam3 |
| -1.684192054 | 5.92E-13 | 1.50E-11 | 4369.819335 | 4053.285117 | 4257.265764 | 1779.768914 | 1354.590293 | 807.0473549 | Pcdh19 |
| -1.387201457 | 5.97E-13 | 1.51E-11 | 1310.758415 | 1387.561842 | 1312.19256 | 544.0461042 | 625.3918754 | 358.5362404 | Ntn4 |
| -1.661651221 | 6.05E-13 | 1.53E-11 | 640.8568665 | 432.1316117 | 606.6787716 | 213.2442016 | 188.8938726 | 125.4195214 | Fibcd1 |
| -1.014038189 | 6.37E-13 | 1.61E-11 | 815.1249617 | 846.029822 | 852.7148765 | 457.4726035 | 432.2436363 | 350.3567064 | Mavs |
| -1.046052616 | 6.51E-13 | 1.64E-11 | 2016.263124 | 2557.234538 | 2077.638219 | 1202.916009 | 1046.574159 | 969.2747792 | Cybrd1 |
| -1.112418221 | 6.95E-13 | 1.75E-11 | 429.1117615 | 450.365013 | 448.9633197 | 215.9781017 | 220.3761847 | 174.4967254 | Nynrin |
| -1.602858459 | 6.96E-13 | 1.75E-11 | 293.2576012 | 402.0464995 | 274.4248863 | 114.8238009 | 116.5696421 | 85.88510702 | Nek5 |
| -1.134829774 | 7.46E-13 | 1.87E-11 | 2108.081798 | 2440.540769 | 2072.381038 | 1150.971909 | 1055.082892 | 804.3208435 | Rhoc |
| -1.40051928 | 8.14E-13 | 2.03E-11 | 1123.373367 | 1399.413553 | 1005.173147 | 538.5783042 | 450.9628489 | 343.5404281 | Pard3b |
| -1.596260935 | 8.79E-13 | 2.18E-11 | 422.5532848 | 598.9672339 | 360.6426666 | 164.9453013 | 159.9641804 | 130.872544 | Mkx |
| -1.198740917 | 8.85E-13 | 2.19E-11 | 3399.164783 | 3766.109046 | 3624.301084 | 1623.025313 | 1876.175626 | 1195.57522 | Clic1 |
| -1.515215689 | 9.21E-13 | 2.28E-11 | 56169.60527 | 38686.71929 | 55620.9827 | 22287.66427 | 17482.89369 | 12869.1335 | Hpca |
| -1.038780406 | 9.70E-13 | 2.39E-11 | 5505.37273 | 6637.869757 | 5854.397574 | 3061.968024 | 3269.055218 | 2423.868576 | Serpinh1 |
| -1.808732579 | 1.02E-12 | 2.49E-11 | 210.8081798 | 281.7060507 | 214.4930146 | 79.28310061 | 74.87685039 | 44.98743701 | Fam160a1 |
| -1.225406476 | 1.02E-12 | 2.49E-11 | 1307.010714 | 1623.684389 | 1302.729633 | 601.4580046 | 715.5844452 | 489.4087845 | Ggta1 |
| -3.345142115 | 1.06E-12 | 2.57E-11 | 90.88174861 | 171.3939726 | 108.2979436 | 8.201700063 | 5.956113099 | 24.53860201 | Kcnj13 |
| -1.289450346 | 1.08E-12 | 2.60E-11 | 311.0591808 | 343.6996152 | 290.1964315 | 112.0899009 | 143.7975877 | 130.872544 | Sox13 |
| -1.016226427 | 1.10E-12 | 2.65E-11 | 500.31808 | 576.1754823 | 537.2839727 | 271.5674021 | 279.9373157 | 244.0227644 | Dock6 |
| -1.258025384 | 1.14E-12 | 2.75E-11 | 1511.260418 | 1847.043555 | 1389.998849 | 672.5394052 | 778.5490694 | 530.3064545 | Gem |
| -1.196242831 | 1.21E-12 | 2.90E-11 | 13659.43312 | 13541.0355 | 13637.12941 | 6441.06845 | 6961.84534 | 4412.858594 | Wwc1 |
| -1.986389114 | 1.25E-12 | 2.99E-11 | 1962.858385 | 2343.903742 | 1911.511277 | 590.5224046 | 708.7774588 | 265.8348551 | Col12a1 |
| -1.00590049 | 1.36E-12 | 3.26E-11 | 9444.206453 | 10990.18266 | 9554.402075 | 5294.653041 | 5505.15025 | 4127.93816 | Ctnna1 |
| -1.096570701 | 1.50E-12 | 3.56E-11 | 2814.523431 | 2463.332521 | 2920.890169 | 1425.273211 | 1379.265619 | 1023.805006 | Lrtm2 |
| -1.851448916 | 1.56E-12 | 3.69E-11 | 1534.683549 | 1697.529664 | 1570.845901 | 595.0789046 | 491.8047673 | 239.9329974 | Grb14 |
| -1.932801453 | 1.63E-12 | 3.83E-11 | 1283.587583 | 866.0865635 | 1325.861232 | 431.9562033 | 288.4460487 | 186.7660264 | Amigo2 |
| -1.389091142 | 1.66E-12 | 3.91E-11 | 583.7044266 | 774.9195568 | 578.2899902 | 267.9222021 | 277.3846958 | 190.8557934 | Mob3b |
| -1.165478578 | 1.70E-12 | 3.99E-11 | 529.3627625 | 647.2857475 | 516.2552458 | 256.986602 | 275.6829492 | 219.4841624 | Rarb |
| -1.498983572 | 1.74E-12 | 4.09E-11 | 297.0053021 | 338.2295948 | 300.7107949 | 115.7351009 | 132.7362348 | 79.06882869 | Tcea3 |
| -1.1144853 | 1.75E-12 | 4.10E-11 | 1784.842589 | 2193.478181 | 1602.388991 | 909.477407 | 918.0922906 | 747.0641055 | Ucp2 |
| -1.505883288 | 1.80E-12 | 4.23E-11 | 699.8831568 | 668.254159 | 707.6166608 | 260.631802 | 304.6126414 | 160.8641687 | Gcnt2 |
| -1.301227711 | 1.84E-12 | 4.29E-11 | 4010.040042 | 5174.6393 | 4157.379312 | 1728.736113 | 2259.068611 | 1421.875661 | Cd44 |
| -1.608384237 | 1.88E-12 | 4.37E-11 | 1772.66256 | 1369.32844 | 1832.653551 | 678.9185052 | 596.4621832 | 351.7199621 | Kcng2 |
| -1.029386957 | 1.91E-12 | 4.44E-11 | 17837.18278 | 19340.1688 | 17750.34839 | 9442.890673 | 10245.3654 | 7215.712245 | Plpp3 |
| -1.551653117 | 1.94E-12 | 4.49E-11 | 3301.724558 | 3610.213465 | 3301.510126 | 1210.206409 | 1523.063207 | 745.7008499 | Prelp |
| -1.086440643 | 1.93E-12 | 4.49E-11 | 920.9975142 | 1081.240699 | 818.0174771 | 477.5212037 | 451.8137222 | 396.7073991 | Skap2 |
| -1.072080542 | 2.14E-12 | 4.93E-11 | 368.2116206 | 420.2799008 | 393.2371934 | 180.4374014 | 182.9377595 | 200.398583 | Ppm1m |
| -1.736870729 | 2.17E-12 | 4.98E-11 | 1081.211731 | 1704.823025 | 1125.03689 | 353.5844027 | 529.2431925 | 287.6469457 | Ifit1 |
| -1.199074591 | 2.17E-12 | 4.98E-11 | 109973.4743 | 114327.9847 | 111581.5793 | 51626.0567 | 58472.86404 | 36190.34819 | Sparc |
| -1.227904397 | 2.18E-12 | 4.99E-11 | 5708.685508 | 6810.175399 | 5588.384178 | 2794.045822 | 2987.416156 | 1944.002581 | H2-K1 |
| -1.159875345 | 2.27E-12 | 5.18E-11 | 698.0093063 | 856.9698628 | 677.1250067 | 349.0279027 | 365.8755189 | 280.8306674 | Nme5 |
| -1.503804199 | 2.38E-12 | 5.41E-11 | 474.0841731 | 691.0459107 | 458.4262468 | 206.8651016 | 209.3148318 | 154.0478904 | Kif27 |
| -1.127036859 | 2.42E-12 | 5.50E-11 | 1976.912264 | 2554.499527 | 2141.775837 | 1150.971909 | 1061.889878 | 838.4022352 | Gpx8 |
| -1.045858826 | 2.44E-12 | 5.52E-11 | 613.6860344 | 639.9923869 | 612.9873896 | 339.9149026 | 305.4635147 | 254.9288097 | Dmrt3 |
| -1.280818837 | 2.51E-12 | 5.69E-11 | 602.4429315 | 693.7809209 | 571.9813722 | 236.0267018 | 315.6739943 | 214.0311397 | Maob |
| -1.116018418 | 2.54E-12 | 5.75E-11 | 575.2720994 | 650.9324277 | 614.038826 | 315.3098024 | 235.6919041 | 299.9162467 | Gm26694 |
| -1.599425657 | 2.63E-12 | 5.93E-11 | 325.1130594 | 458.5700436 | 369.0541574 | 150.3645012 | 136.9906013 | 89.97487402 | Csrp2 |
| -1.077604908 | 2.78E-12 | 6.24E-11 | 464.7149207 | 504.153547 | 483.6607191 | 251.5188019 | 238.244524 | 194.9455604 | Rbp1 |
| -1.478836509 | 2.87E-12 | 6.42E-11 | 1441.92795 | 2431.424068 | 1446.776412 | 625.1518048 | 695.163486 | 587.5631925 | Ctnnal1 |
| -1.362766725 | 2.95E-12 | 6.59E-11 | 5050.963987 | 5533.837306 | 4711.486266 | 2152.490617 | 2413.076678 | 1376.888224 | Thbs1 |
| -1.147232317 | 2.96E-12 | 6.61E-11 | 394.4455275 | 453.1000232 | 456.3233741 | 169.5018013 | 212.718325 | 207.2148614 | Eef2kmt |
| -1.054771433 | 3.10E-12 | 6.90E-11 | 770.15255 | 824.1497404 | 820.1203498 | 430.1336033 | 410.9718038 | 316.2753147 | Galnt4 |
| -1.5244275 | 3.13E-12 | 6.96E-11 | 691.4508296 | 775.8312269 | 691.8451156 | 290.7047022 | 294.4021618 | 160.8641687 | Glycam1 |
| -1.089446641 | 3.18E-12 | 7.07E-11 | 566.8397722 | 609.9072747 | 588.8043537 | 287.0595022 | 310.5687545 | 227.6636964 | Rhoj |
| -1.23892755 | 3.68E-12 | 8.11E-11 | 887.2682055 | 1098.562431 | 933.6754751 | 415.5528032 | 492.6556406 | 324.4548487 | C1s1 |
| -1.472745454 | 3.74E-12 | 8.23E-11 | 996.8884589 | 1091.26907 | 999.9159649 | 340.8262026 | 500.3135003 | 267.1981107 | Chil1 |
| -1.499068915 | 3.86E-12 | 8.49E-11 | 183.6373477 | 167.7472923 | 195.5671603 | 57.41190044 | 63.81549749 | 73.61580602 | Gm10020 |
| -1.503061978 | 4.40E-12 | 9.54E-11 | 753.2878957 | 821.4147302 | 768.5999688 | 257.897902 | 370.9807587 | 193.5823047 | Rarres2 |
| -1.26614994 | 4.46E-12 | 9.64E-11 | 1232.99362 | 1186.082757 | 1155.528544 | 510.3280039 | 606.6726628 | 363.9892631 | Tpbg |
| -1.351462986 | 4.95E-12 | 1.06E-10 | 5028.477781 | 4740.684348 | 5055.305951 | 2323.815018 | 2162.069055 | 1318.26823 | Foxo1 |
| -1.538739658 | 5.62E-12 | 1.20E-10 | 200.5020021 | 280.7943806 | 209.2358328 | 75.63790058 | 74.87685039 | 88.61161836 | Prtg |
| -1.378549258 | 5.91E-12 | 1.26E-10 | 282.0144983 | 325.4662139 | 237.6246142 | 119.3803009 | 99.55217609 | 106.333942 | Arhgap25 |
| -1.173201816 | 5.91E-12 | 1.26E-10 | 1411.946342 | 1727.614777 | 1426.799121 | 787.3632061 | 700.2687258 | 533.0329658 | Stom |
| -1.045363057 | 5.95E-12 | 1.26E-10 | 2467.861091 | 2963.839387 | 2336.291561 | 1254.86011 | 1429.467144 | 1075.608721 | Dnah9 |
| -1.076155307 | 6.49E-12 | 1.37E-10 | 494.6965285 | 489.5668259 | 463.6834285 | 248.7849019 | 243.3497638 | 190.8557934 | Jag1 |
| -1.044656649 | 6.70E-12 | 1.41E-10 | 50722.3219 | 53743.86211 | 52113.39105 | 23698.35668 | 31051.77021 | 21148.18516 | Clu |
| -1.404063643 | 7.10E-12 | 1.49E-10 | 1164.598078 | 1073.035669 | 1194.431689 | 493.0133038 | 509.6731066 | 289.0102014 | Rhbdl3 |
| -1.254278286 | 7.33E-12 | 1.53E-10 | 496.570379 | 508.7118973 | 515.2038095 | 227.8250018 | 246.753257 | 158.1376574 | Smpdl3b |
| -1.242036743 | 7.49E-12 | 1.56E-10 | 2505.338101 | 2857.17399 | 2474.029722 | 1178.310909 | 1319.704488 | 809.7738662 | Gab1 |
| -1.156118328 | 7.49E-12 | 1.56E-10 | 743.9186432 | 779.4779072 | 806.4516773 | 379.1008029 | 393.9543378 | 267.1981107 | Crtap |
| -1.207737787 | 7.70E-12 | 1.60E-10 | 4031.589322 | 4095.22194 | 4213.105438 | 1777.035014 | 2222.481059 | 1337.353809 | Ltbp3 |
| -2.079402603 | 7.85E-12 | 1.63E-10 | 206.1235535 | 260.7376391 | 181.8984878 | 68.34750053 | 55.30676449 | 27.26511334 | AC166078.1 |
| -1.378069861 | 8.05E-12 | 1.67E-10 | 211.745105 | 233.3875371 | 231.3159961 | 72.90400056 | 98.70130279 | 88.61161836 | Tbc1d2 |
| -2.33402554 | 8.14E-12 | 1.68E-10 | 167.7096186 | 220.6241562 | 170.332688 | 42.83110033 | 50.20152469 | 14.99581234 | Cnn1 |
| -1.401049073 | 8.13E-12 | 1.68E-10 | 3707.413188 | 4765.299439 | 3785.170845 | 1671.324213 | 1889.789599 | 1075.608721 | Anxa3 |
| -1.544884005 | 8.66E-12 | 1.78E-10 | 1010.942338 | 1201.581148 | 991.5044742 | 326.2454025 | 506.2696134 | 261.7450881 | Itih3 |
| -1.066057734 | 9.07E-12 | 1.86E-10 | 698.0093063 | 663.6958087 | 637.1704256 | 337.1810026 | 353.9632928 | 259.0185767 | Smim1 |
| -1.010408866 | 9.32E-12 | 1.91E-10 | 3092.790228 | 3564.629961 | 3136.43462 | 1578.371612 | 1908.508812 | 1370.071945 | Tapbp |
| -1.714207287 | 9.49E-12 | 1.93E-10 | 2019.073899 | 1974.677365 | 2082.895401 | 717.1931055 | 787.9086757 | 342.1771724 | Kif26b |
| -1.41240623 | 9.50E-12 | 1.93E-10 | 558.407445 | 705.6326318 | 541.4897181 | 206.8651016 | 288.4460487 | 179.949748 | Dnajb13 |
| -1.203234702 | 9.50E-12 | 1.93E-10 | 871.3404763 | 1157.820985 | 957.8585111 | 424.6658033 | 510.5239799 | 358.5362404 | Ptpn21 |
| -1.226733551 | 9.65E-12 | 1.96E-10 | 3776.745656 | 3148.908411 | 3798.839518 | 1817.132214 | 1621.76451 | 1138.318482 | Syt17 |
| -1.339982449 | 1.07E-11 | 2.16E-10 | 1546.863577 | 1185.171087 | 1560.331537 | 633.3535049 | 645.8128346 | 411.7032114 | Mycl |
| -1.151071025 | 1.14E-11 | 2.28E-10 | 544.3535664 | 544.2670299 | 473.1463556 | 264.277002 | 245.9023837 | 189.4925377 | Cebpa |
| -1.387639759 | 1.15E-11 | 2.31E-10 | 3605.288336 | 3994.938233 | 3438.196851 | 1601.154112 | 1674.518654 | 937.9198989 | Col5a2 |
| -1.408185083 | 1.23E-11 | 2.46E-10 | 254.8436662 | 350.9929757 | 278.6306317 | 120.2916009 | 118.2713887 | 92.70138536 | Plscr4 |
| -1.179603689 | 1.32E-11 | 2.62E-10 | 413.1840323 | 470.4217545 | 398.4943751 | 210.5103016 | 198.2534789 | 154.0478904 | Manba |
| -1.070833819 | 1.39E-11 | 2.75E-10 | 2226.134378 | 2480.654252 | 2358.371724 | 1191.069109 | 1289.923923 | 876.5733939 | Rnf144a |
| -1.537957433 | 1.66E-11 | 3.25E-10 | 814.1880365 | 1355.653389 | 834.8404586 | 390.947703 | 367.5772655 | 274.0143891 | Ecm2 |
| -1.146340962 | 1.77E-11 | 3.44E-10 | 1980.659964 | 2081.342763 | 2010.346293 | 1019.744708 | 1038.916299 | 678.9013222 | Tcp11l2 |
| -2.186136762 | 1.82E-11 | 3.54E-10 | 90.88174861 | 159.5422617 | 113.5551254 | 26.4277002 | 29.7805655 | 23.17534634 | Acox2 |
| -1.038104428 | 1.83E-11 | 3.54E-10 | 455.3456683 | 540.6203496 | 441.6032653 | 236.9380018 | 242.4988905 | 219.4841624 | Rhod |
| -3.536615472 | 1.96E-11 | 3.78E-10 | 68.39554277 | 128.5454794 | 45.21176287 | 6.379100049 | 7.657859699 | 6.816278335 | Epn3 |
| -1.461354963 | 2.06E-11 | 3.98E-10 | 583.7044266 | 652.7557679 | 614.038826 | 213.2442016 | 297.805655 | 156.7744017 | Il1r1 |
| -1.090140242 | 2.07E-11 | 3.99E-10 | 355.0946672 | 380.1664179 | 348.0254305 | 170.4131013 | 187.192126 | 148.5948677 | Lmcd1 |
| -1.483979383 | 2.08E-11 | 4.00E-10 | 4137.461875 | 3273.80721 | 4095.344567 | 1859.052014 | 1301.836149 | 948.8259442 | Islr2 |
| -1.305251168 | 2.19E-11 | 4.21E-10 | 1890.715141 | 2161.569729 | 1890.48255 | 860.2672066 | 977.6534216 | 561.6613348 | Cdon |
| -1.094931786 | 2.42E-11 | 4.62E-10 | 905.0697851 | 963.6352607 | 951.549893 | 502.1263039 | 472.2346814 | 340.8139168 | Nkain4 |
| -1.800374603 | 2.44E-11 | 4.65E-10 | 1475.657258 | 1284.543124 | 1487.782429 | 499.3924039 | 504.5678668 | 211.3046284 | Ltbp2 |
| -2.335596185 | 2.46E-11 | 4.69E-10 | 80.57557093 | 112.1354182 | 89.3720894 | 24.60510019 | 17.8683393 | 12.269301 | Prcd |
| -1.175988994 | 2.54E-11 | 4.82E-10 | 1351.046201 | 1236.224611 | 1355.30145 | 563.1834043 | 724.0931782 | 452.6008814 | Me3 |
| -1.385397423 | 2.66E-11 | 5.04E-10 | 2929.765236 | 3208.166965 | 2872.524097 | 1202.004709 | 1470.309062 | 771.6027075 | Cavin1 |
| -1.069173247 | 2.74E-11 | 5.16E-10 | 403.8147799 | 420.2799008 | 370.1055937 | 212.3329016 | 184.6395061 | 170.4069584 | Pdk4 |
| -1.136642148 | 2.92E-11 | 5.46E-10 | 3293.29223 | 2804.297126 | 3149.051856 | 1595.686312 | 1547.738532 | 1056.523142 | Pdk2 |
| -1.245399907 | 3.16E-11 | 5.88E-10 | 257.6544419 | 269.8543398 | 226.0588144 | 115.7351009 | 105.5082892 | 95.42789669 | Vsig10 |
| -1.259871928 | 3.25E-11 | 6.02E-10 | 1416.630968 | 1876.216998 | 1307.986814 | 663.4264051 | 744.5141374 | 509.8576195 | Glis3 |
| -1.182342559 | 3.25E-11 | 6.02E-10 | 1195.516611 | 1196.111128 | 1278.546597 | 501.2150039 | 683.2512598 | 428.0622794 | Npnt |
| -1.039061797 | 3.38E-11 | 6.24E-10 | 2104.334097 | 2205.329892 | 2182.781854 | 1118.165109 | 1213.345326 | 822.0431672 | Ifitm2 |
| -1.092479082 | 3.42E-11 | 6.30E-10 | 661.4692218 | 828.7080908 | 591.9586627 | 316.2211024 | 338.6475734 | 321.7283374 | Eva1a |
| -1.560604487 | 3.66E-11 | 6.69E-10 | 1953.489132 | 3120.646639 | 2202.759145 | 806.5005062 | 1072.100358 | 584.8366811 | Ifit3 |
| -1.335924415 | 3.72E-11 | 6.80E-10 | 1232.99362 | 1860.718606 | 1323.758359 | 687.1202053 | 596.4621832 | 463.5069268 | Synpo2 |
| -1.084012838 | 3.80E-11 | 6.92E-10 | 1419.441744 | 1639.18278 | 1368.970122 | 739.9756057 | 793.0139155 | 550.7552895 | Cpq |
| -1.045933003 | 4.00E-11 | 7.26E-10 | 416.9317333 | 488.6551558 | 474.197792 | 243.3171019 | 227.1831711 | 194.9455604 | Aifm2 |
| -1.204778684 | 4.08E-11 | 7.40E-10 | 371.9593216 | 411.1632002 | 376.4142118 | 185.9052014 | 183.7886328 | 129.5092884 | Mfsd9 |
| -1.518435984 | 4.17E-11 | 7.55E-10 | 416.9317333 | 562.5004313 | 365.8998484 | 159.4775012 | 188.8938726 | 118.603243 | Bdh2 |
| -1.129444909 | 4.19E-11 | 7.56E-10 | 1255.479826 | 1663.797872 | 1289.06096 | 639.7326049 | 741.9615175 | 538.4859885 | Ddx58 |
| -1.433089327 | 4.26E-11 | 7.68E-10 | 4163.695782 | 4266.615913 | 4197.333893 | 1664.945113 | 2009.762734 | 996.5398926 | Ass1 |
| -1.115321874 | 4.59E-11 | 8.23E-10 | 2486.599596 | 2403.162296 | 2489.801267 | 1292.22341 | 1264.397724 | 843.8552579 | F730043M19Rik |
| -1.111984863 | 4.67E-11 | 8.34E-10 | 795.4495316 | 960.9002505 | 839.046204 | 380.9234029 | 480.7434144 | 335.3608941 | Hacl1 |
| -1.129570521 | 5.16E-11 | 9.18E-10 | 2549.373587 | 3524.516478 | 2628.590865 | 1397.934211 | 1474.563429 | 1101.510579 | Vwa5a |
| -1.380316232 | 5.37E-11 | 9.53E-10 | 956.6006735 | 1300.041515 | 992.5559105 | 462.0291036 | 487.5504008 | 294.4632241 | Trem2 |
| -1.106920191 | 5.41E-11 | 9.58E-10 | 351.3469663 | 403.8698396 | 350.1283032 | 173.1470013 | 188.8938726 | 148.5948677 | Tram2 |
| -1.171871931 | 5.50E-11 | 9.74E-10 | 389.7609012 | 474.0684348 | 387.9800116 | 209.5990016 | 188.0429993 | 155.411146 | Akna |
| -1.758774958 | 5.62E-11 | 9.91E-10 | 464.7149207 | 634.5223665 | 476.3006647 | 146.7193011 | 218.6744381 | 96.79115236 | Zfp185 |
| -1.273568944 | 5.87E-11 | 1.03E-09 | 2571.859793 | 2242.708364 | 2505.572812 | 1198.359509 | 1124.854502 | 699.3501572 | B3galt5 |
| -1.517822251 | 6.09E-11 | 1.07E-09 | 602.4429315 | 594.4088836 | 534.1296637 | 164.0340013 | 283.3408089 | 154.0478904 | C1ra |
| -1.02479496 | 6.77E-11 | 1.18E-09 | 1859.796608 | 2425.042378 | 1998.780494 | 1170.109209 | 1035.512806 | 879.2999052 | Lipa |
| -1.049310797 | 6.84E-11 | 1.19E-09 | 1623.691447 | 1377.533471 | 1580.308828 | 726.3061056 | 877.2503722 | 605.2855161 | Tspan4 |
| -2.824715516 | 7.08E-11 | 1.23E-09 | 65.58476704 | 84.78531622 | 80.96059863 | 14.58080011 | 13.6139728 | 2.726511334 | Cldn9 |
| -1.415867549 | 7.18E-11 | 1.24E-09 | 777.647952 | 995.543713 | 758.0856054 | 359.0522028 | 370.1298854 | 215.3943954 | Aif1l |
| -1.059411634 | 7.40E-11 | 1.28E-09 | 2036.875479 | 1834.280175 | 2107.078437 | 976.0023075 | 1131.661489 | 755.2436395 | Tmem176a |
| -1.254534697 | 8.08E-11 | 1.39E-09 | 397.2563032 | 486.8318157 | 417.4202293 | 183.1713014 | 216.9726915 | 141.7785894 | Aifm3 |
| -1.179169411 | 8.71E-11 | 1.50E-09 | 1255.479826 | 1284.543124 | 1221.769034 | 531.2879041 | 701.1195991 | 423.9725124 | Arpin |
| -1.194880627 | 8.79E-11 | 1.51E-09 | 825.4311394 | 1147.792614 | 783.3200777 | 377.2782029 | 453.5154688 | 372.1687971 | Parp9 |
| -1.00036024 | 8.85E-11 | 1.51E-09 | 3063.745546 | 3178.081853 | 3118.560202 | 1737.849113 | 1720.465812 | 1214.660799 | Wwc2 |
| -1.2261372 | 9.08E-11 | 1.55E-09 | 2600.904476 | 2597.34802 | 2711.654336 | 1169.197909 | 1404.791818 | 801.5943322 | Amotl2 |
| -1.158768491 | 9.17E-11 | 1.56E-09 | 288.572975 | 293.5577615 | 249.190414 | 135.783701 | 119.9731353 | 115.8767317 | Ccdc102a |
| -1.198742382 | 9.20E-11 | 1.57E-09 | 513.4350334 | 538.7970095 | 489.9693372 | 242.4058019 | 258.6654832 | 166.3171914 | Ephb4 |
| -1.415628976 | 9.92E-11 | 1.68E-09 | 3370.1201 | 4520.971862 | 3296.252944 | 1510.024112 | 1728.974545 | 950.1891999 | Cyp1b1 |
| -1.66032206 | 1.04E-10 | 1.76E-09 | 728.9278393 | 682.8408801 | 766.4970961 | 300.7290023 | 256.1128633 | 128.1460327 | Kirrel2 |
| -1.298450849 | 1.08E-10 | 1.82E-09 | 2326.385379 | 2681.221667 | 2415.149286 | 1002.430008 | 1300.134402 | 710.2562025 | S100a11 |
| -1.899065814 | 1.17E-10 | 1.96E-09 | 132.1064593 | 170.4823025 | 153.5097065 | 34.62940027 | 57.00851109 | 28.62836901 | Slc39a4 |
| -1.250894525 | 1.21E-10 | 2.02E-09 | 909.7544113 | 1146.880944 | 1094.545236 | 510.3280039 | 490.1030207 | 319.0018261 | Oaf |
| -1.295682897 | 1.21E-10 | 2.03E-09 | 364.4639197 | 461.3050538 | 361.694103 | 153.0984012 | 195.700859 | 132.2357997 | Ttc25 |
| -1.177196598 | 1.30E-10 | 2.16E-09 | 546.2274169 | 688.3109005 | 512.0495004 | 282.5030022 | 275.6829492 | 211.3046284 | Cxcr4 |
| -1.077282376 | 1.37E-10 | 2.27E-09 | 505.9396314 | 542.4436898 | 475.2492283 | 242.4058019 | 278.2355691 | 197.6720717 | Parp10 |
| -1.424829141 | 1.47E-10 | 2.43E-09 | 3768.313329 | 4601.198827 | 4002.818169 | 1613.912312 | 1996.999635 | 992.4501256 | Tm4sf1 |
| -1.283558602 | 1.55E-10 | 2.55E-09 | 533.1104635 | 552.4720605 | 579.3414266 | 184.9939014 | 298.6565283 | 197.6720717 | Il3ra |
| -1.738796711 | 1.67E-10 | 2.74E-09 | 156.4665156 | 206.0374351 | 152.4582702 | 58.32320045 | 59.56113099 | 34.08139168 | Osr1 |
| -1.152593935 | 1.73E-10 | 2.85E-09 | 680.2077267 | 536.0619993 | 657.1477162 | 328.9793025 | 230.5866643 | 283.5571787 | H1fx |
| -1.549625571 | 1.82E-10 | 2.98E-09 | 172.3942448 | 233.3875371 | 159.8183246 | 66.52490051 | 57.00851109 | 70.88929468 | Rbm47 |
| -2.33992188 | 1.86E-10 | 3.04E-09 | 139.6018613 | 288.9994112 | 127.2237979 | 42.83110033 | 39.99104509 | 25.90185767 | F5 |
| -1.001542552 | 1.89E-10 | 3.09E-09 | 981.897655 | 1181.524407 | 1054.590655 | 567.7399044 | 597.3130565 | 437.6050691 | Olfml1 |
| -1.458709179 | 1.90E-10 | 3.10E-09 | 1011.879263 | 918.0517573 | 931.5726024 | 309.8420024 | 485.8486542 | 241.2962531 | Hspb1 |
| -1.168800394 | 2.18E-10 | 3.53E-09 | 243.6005633 | 228.8291868 | 234.4703051 | 113.9125009 | 107.2100358 | 91.33812969 | Pole |
| -1.70446163 | 2.22E-10 | 3.59E-09 | 309.1853303 | 360.1096764 | 268.1162682 | 76.54920059 | 137.8414746 | 70.88929468 | Megf6 |
| -1.010820154 | 2.35E-10 | 3.78E-09 | 741.1078675 | 714.7493324 | 743.3654965 | 384.568603 | 412.6735504 | 289.0102014 | Eci1 |
| -1.082534091 | 2.39E-10 | 3.83E-09 | 1372.595482 | 1692.059644 | 1294.318142 | 819.2587063 | 654.3215676 | 582.1101698 | Tcirg1 |
| -1.387722409 | 2.58E-10 | 4.12E-09 | 1479.404959 | 1844.308545 | 1579.257392 | 632.4422049 | 816.8383679 | 419.8827454 | Tppp3 |
| -1.29725746 | 2.65E-10 | 4.22E-09 | 5688.073152 | 7821.217504 | 5797.620011 | 2552.55132 | 3343.081195 | 1956.271882 | Cd109 |
| -1.924328174 | 2.67E-10 | 4.25E-09 | 122.7372069 | 113.9587584 | 119.8637434 | 36.45200028 | 19.5700859 | 39.53441434 | Cdkn1c |
| -1.041292484 | 3.14E-10 | 4.97E-09 | 1424.12637 | 1470.523818 | 1382.638795 | 778.250206 | 768.3385898 | 526.2166875 | St5 |
| -1.017076437 | 3.23E-10 | 5.10E-09 | 453.4718178 | 462.2167239 | 397.4429387 | 196.8408015 | 245.0515104 | 205.8516057 | Foxc1 |
| -1.889042164 | 3.35E-10 | 5.28E-09 | 707.3785587 | 816.8563799 | 758.0856054 | 245.1397019 | 273.1303293 | 94.06464102 | Tagln |
| -1.230295232 | 4.03E-10 | 6.27E-09 | 444.1025654 | 604.4372543 | 510.9980641 | 219.6233017 | 264.6215963 | 177.2232367 | Lrrc23 |
| -3.06430268 | 4.31E-10 | 6.67E-09 | 118.0525807 | 434.8666219 | 135.6352886 | 38.2746003 | 28.0788189 | 14.99581234 | Gmnc |
| -1.610910726 | 4.96E-10 | 7.60E-09 | 1283.587583 | 1092.18074 | 1319.552614 | 544.9574042 | 433.0945096 | 227.6636964 | Apcdd1 |
| -1.340302369 | 4.95E-10 | 7.60E-09 | 512.4981081 | 659.1374583 | 507.8437551 | 205.9538016 | 285.0425555 | 169.0437027 | Ifi35 |
| -1.404987447 | 5.08E-10 | 7.78E-09 | 185.5111982 | 154.0722413 | 171.3841244 | 64.7023005 | 73.17510379 | 53.16697101 | Adamts2 |
| -1.119960053 | 5.29E-10 | 8.08E-09 | 600.569081 | 639.9923869 | 594.0615354 | 297.0838023 | 330.9897137 | 211.3046284 | Tcf7l1 |
| -1.260858875 | 5.32E-10 | 8.11E-09 | 530.2996877 | 660.0491284 | 553.0555179 | 293.4386023 | 254.4111167 | 175.859981 | Cyp39a1 |
| -1.123395245 | 5.82E-10 | 8.83E-09 | 490.9488275 | 571.6171319 | 437.3975199 | 244.2284019 | 254.4111167 | 186.7660264 | Tekt1 |
| -1.328622931 | 6.03E-10 | 9.12E-09 | 207.997404 | 295.3811017 | 264.9619592 | 116.6464009 | 94.44693629 | 94.06464102 | Rlbp1 |
| -1.136518941 | 6.25E-10 | 9.43E-09 | 1996.587694 | 1957.355634 | 1983.008948 | 1080.801808 | 970.8464352 | 643.4566748 | Isoc1 |
| -1.2548185 | 7.44E-10 | 1.11E-08 | 9580.997539 | 14725.29492 | 9530.219039 | 5694.713744 | 4618.540272 | 3863.46656 | Lyz2 |
| -1.137399581 | 8.61E-10 | 1.27E-08 | 533.1104635 | 610.8189448 | 550.9526452 | 273.3900021 | 300.3582749 | 192.219049 | Tep1 |
| -1.445764107 | 8.86E-10 | 1.31E-08 | 153.6557399 | 216.9774759 | 225.007378 | 72.90400056 | 79.13121689 | 65.43627202 | Cdc20 |
| -2.435731559 | 1.02E-09 | 1.48E-08 | 84.3232719 | 117.6054386 | 57.82899902 | 19.13730015 | 12.7630995 | 16.359068 | Tgtp1 |
| -1.796418765 | 1.08E-09 | 1.56E-08 | 825.4311394 | 542.4436898 | 865.3321127 | 294.3499023 | 228.0340444 | 117.2399874 | Hrh3 |
| -1.125735025 | 1.10E-09 | 1.60E-08 | 6483.522684 | 5995.14236 | 6527.316835 | 3652.490428 | 2933.811138 | 2118.499307 | Nr3c2 |
| -1.182720236 | 1.16E-09 | 1.67E-08 | 435.6702382 | 559.7654211 | 453.1690651 | 204.1312016 | 257.8146099 | 173.1334697 | Dnaaf3 |
| -2.00717472 | 1.22E-09 | 1.75E-08 | 118.0525807 | 171.3939726 | 137.7381613 | 41.00850032 | 45.09628489 | 17.72232367 | Cfi |
| -1.254051271 | 1.25E-09 | 1.79E-08 | 654.9107451 | 689.2225705 | 590.9072264 | 284.3256022 | 334.3932069 | 188.129282 | Wee1 |
| -1.26868612 | 1.25E-09 | 1.79E-08 | 6621.250695 | 6942.367559 | 6722.883996 | 2819.562222 | 3701.298854 | 1893.562121 | Pltp |
| -1.473543043 | 1.34E-09 | 1.91E-08 | 1861.670459 | 2556.322867 | 1779.030297 | 666.1603051 | 1039.767172 | 522.1269205 | Oasl2 |
| -1.473362756 | 1.45E-09 | 2.07E-08 | 171.4573195 | 225.1825065 | 218.6987599 | 69.25880053 | 92.74518969 | 57.25673801 | Morn5 |
| -1.099788874 | 1.49E-09 | 2.12E-08 | 815.1249617 | 825.0614105 | 824.3260952 | 429.2223033 | 437.3488761 | 278.1041561 | Loxl3 |
| -1.071383303 | 1.60E-09 | 2.26E-08 | 601.5060062 | 709.279312 | 576.1871175 | 292.5273023 | 356.5159127 | 245.3860201 | Vstm4 |
| -1.321453118 | 1.65E-09 | 2.32E-08 | 332.6084614 | 386.5481083 | 289.1449951 | 142.1628011 | 159.9641804 | 98.15440802 | Nod1 |
| -1.010020749 | 1.66E-09 | 2.34E-08 | 402.8778546 | 456.7467035 | 484.7121554 | 256.075302 | 212.718325 | 196.308816 | Mcm3 |
| -1.251048518 | 1.75E-09 | 2.45E-08 | 673.64925 | 472.2450946 | 673.9706977 | 226.0024017 | 313.9722477 | 222.2106737 | Nppc |
| -1.330447496 | 1.79E-09 | 2.50E-08 | 668.0276985 | 859.7048731 | 740.2111875 | 276.1239021 | 402.4630708 | 219.4841624 | Lgals9 |
| -1.01109077 | 1.81E-09 | 2.53E-08 | 1989.092292 | 2165.216409 | 2002.986239 | 1135.479809 | 1147.828082 | 766.1496849 | H2afv |
| -1.003890545 | 1.81E-09 | 2.53E-08 | 845.1065695 | 995.543713 | 818.0174771 | 497.5698038 | 469.6820615 | 354.4464734 | Plekha2 |
| -1.904280209 | 1.88E-09 | 2.61E-08 | 130.2326088 | 167.7472923 | 139.841034 | 55.58930043 | 37.43842519 | 21.81209067 | Nek2 |
| -4.098866692 | 1.91E-09 | 2.64E-08 | 39.35086022 | 37.37847274 | 43.10889018 | 0.911300007 | 3.4034932 | 2.726511334 | Gm11578 |
| -1.010405667 | 2.08E-09 | 2.88E-08 | 1737.996326 | 1620.949379 | 1818.984878 | 921.3243071 | 992.969141 | 650.2729532 | Fkbp10 |
| -1.138414888 | 2.09E-09 | 2.89E-08 | 502.1919304 | 568.8821217 | 496.2779553 | 264.277002 | 268.8759628 | 174.4967254 | Pstpip1 |
| -1.230459069 | 2.09E-09 | 2.89E-08 | 329.7976857 | 382.9014281 | 352.2311759 | 161.3001012 | 179.5342663 | 109.0604534 | Ackr3 |
| -1.171726988 | 2.13E-09 | 2.94E-08 | 438.4810139 | 542.4436898 | 450.014756 | 225.0911017 | 246.753257 | 159.500913 | Irf7 |
| -1.057991974 | 2.26E-09 | 3.11E-08 | 782.3325782 | 933.5501485 | 778.0628959 | 391.859003 | 481.5942877 | 320.3650817 | Gbp2 |
| -1.251795591 | 2.49E-09 | 3.41E-08 | 2820.144983 | 2673.928306 | 2904.067187 | 1463.547811 | 1289.073049 | 768.8761962 | Sh3bp4 |
| -1.509846529 | 2.50E-09 | 3.43E-08 | 431.9225372 | 605.3489244 | 428.9860291 | 176.7922014 | 222.9288046 | 111.7869647 | Irgm2 |
| -1.146628666 | 2.66E-09 | 3.63E-08 | 10582.57062 | 10668.36312 | 10509.10628 | 4930.133038 | 6057.367022 | 3352.245685 | Fn1 |
| -1.616643667 | 2.95E-09 | 3.99E-08 | 342.9146391 | 318.1728533 | 329.0995763 | 156.7436012 | 97.85042949 | 65.43627202 | Prss12 |
| -1.160197548 | 3.07E-09 | 4.13E-08 | 1419.441744 | 1517.018991 | 1464.65083 | 729.0400056 | 783.6543092 | 451.2376258 | Bgn |
| -1.348717182 | 3.14E-09 | 4.23E-08 | 1227.372069 | 1457.760437 | 1256.466433 | 525.8201041 | 683.2512598 | 333.9976384 | 2410004P03Rik |
| -1.171061468 | 3.16E-09 | 4.25E-08 | 3627.774542 | 4386.044692 | 3933.42337 | 1684.993713 | 2312.673629 | 1303.272418 | Osmr |
| -1.086870214 | 3.18E-09 | 4.27E-08 | 499.3811547 | 600.7905741 | 481.5578464 | 266.0996021 | 279.9373157 | 194.9455604 | Psmb8 |
| -1.476711612 | 3.30E-09 | 4.41E-08 | 147.0972632 | 182.3340134 | 164.02407 | 52.85540041 | 74.87685039 | 47.71394835 | Hdc |
| -1.055319811 | 3.45E-09 | 4.61E-08 | 672.7123247 | 696.5159311 | 753.87986 | 372.7217029 | 388.849098 | 254.9288097 | Scara3 |
| -1.046622359 | 3.47E-09 | 4.62E-08 | 277.329872 | 268.0309997 | 320.6880855 | 141.2515011 | 126.7801217 | 152.6846347 | Scrn2 |
| -1.028908661 | 3.65E-09 | 4.85E-08 | 1380.090883 | 1773.19828 | 1331.118414 | 754.5564058 | 842.3645669 | 597.1059821 | Ddo |
| -1.030454061 | 3.74E-09 | 4.96E-08 | 1238.615172 | 1161.467665 | 1214.408979 | 631.5309049 | 693.4617394 | 438.9683248 | Ccdc74a |
| -1.407529844 | 3.84E-09 | 5.09E-08 | 178.9527215 | 287.1760711 | 221.853069 | 86.57350067 | 95.29780959 | 76.34231735 | Tbxas1 |
| -1.286115498 | 4.12E-09 | 5.43E-08 | 1103.697937 | 1461.407117 | 1130.294072 | 633.3535049 | 531.7958124 | 346.2669394 | Plau |
| -1.025451965 | 4.72E-09 | 6.14E-08 | 1425.063295 | 1721.233086 | 1568.743028 | 776.427606 | 935.1097566 | 599.8324935 | Flnc |
| -1.58845022 | 4.72E-09 | 6.14E-08 | 116.1787302 | 151.3372311 | 110.4008163 | 48.29890037 | 36.58755189 | 40.89767001 | Moxd1 |
| -1.17479004 | 4.90E-09 | 6.33E-08 | 1124.310292 | 1383.915161 | 1247.003506 | 612.3936047 | 660.2776807 | 385.8013538 | Lima1 |
| -1.010501309 | 5.10E-09 | 6.58E-08 | 368.2116206 | 433.0432818 | 378.5170845 | 202.3086016 | 217.8235648 | 162.2274244 | Echdc2 |
| -1.006788439 | 5.12E-09 | 6.60E-08 | 13240.62754 | 19756.80202 | 14016.69793 | 8616.341567 | 7874.83239 | 6903.526698 | Gpnmb |
| -1.688877038 | 5.16E-09 | 6.65E-08 | 162.0880671 | 281.7060507 | 189.2585423 | 79.28310061 | 68.92073729 | 46.35069268 | Sctr |
| -1.006824461 | 5.27E-09 | 6.78E-08 | 374.7700973 | 476.803445 | 378.5170845 | 206.8651016 | 219.5253114 | 184.039515 | Apobec3 |
| -1.241364849 | 5.32E-09 | 6.83E-08 | 205.1866283 | 221.5358262 | 216.5958872 | 70.17010054 | 102.104796 | 100.8809194 | Trp73 |
| -1.102275467 | 5.36E-09 | 6.88E-08 | 569.650548 | 591.6738734 | 602.4730262 | 286.1482022 | 330.1388404 | 200.398583 | Tcaf2 |
| -1.095165649 | 5.40E-09 | 6.94E-08 | 1290.14606 | 1969.207344 | 1434.159176 | 845.6864065 | 642.4093414 | 708.8929468 | Apod |
| -1.472681677 | 5.88E-09 | 7.52E-08 | 252.0328905 | 286.264401 | 211.3387055 | 89.30740069 | 116.5696421 | 61.34650502 | Col8a2 |
| -1.448521616 | 6.41E-09 | 8.16E-08 | 320.4284332 | 486.8318157 | 316.4823401 | 133.049801 | 172.7272799 | 103.6074307 | Cfap126 |
| -1.262296974 | 6.44E-09 | 8.20E-08 | 1057.7886 | 1647.387811 | 1213.357543 | 657.9586051 | 567.532491 | 404.8869331 | Lpar1 |
| -1.385893956 | 6.73E-09 | 8.53E-08 | 705.5047083 | 949.9602097 | 705.5137881 | 321.6889025 | 382.8929849 | 194.9455604 | Drc7 |
| -1.061130599 | 7.08E-09 | 8.95E-08 | 646.4784179 | 848.7648322 | 705.5137881 | 322.6002025 | 427.9892698 | 301.2795024 | Ccdc114 |
| -1.224748195 | 7.23E-09 | 9.13E-08 | 1687.402363 | 1661.974532 | 1769.56737 | 868.4689067 | 844.9171868 | 471.6864608 | Podxl |
| -1.876606754 | 7.27E-09 | 9.17E-08 | 69.33246801 | 94.81368695 | 84.11490767 | 20.04860015 | 25.526199 | 21.81209067 | Bmp4 |
| -1.073110062 | 7.34E-09 | 9.25E-08 | 209.8712545 | 247.9742582 | 234.4703051 | 110.2673009 | 116.5696421 | 100.8809194 | Gm3608 |
| -1.036924744 | 7.42E-09 | 9.33E-08 | 231.4205351 | 259.8259691 | 227.1102507 | 112.0899009 | 114.0170222 | 125.4195214 | C330011M18Rik |
| -1.198845039 | 7.89E-09 | 9.88E-08 | 1296.704537 | 1470.523818 | 1235.437706 | 598.7241046 | 739.4088976 | 400.7971661 | Pmp22 |
| -1.14384426 | 7.95E-09 | 9.94E-08 | 339.1669381 | 393.8414689 | 337.511067 | 165.8566013 | 192.2973658 | 122.69301 | Nek11 |
| -1.34151327 | 8.07E-09 | 1.01E-07 | 609.9383334 | 762.1561759 | 642.4276073 | 259.720502 | 353.9632928 | 177.2232367 | Cd82 |
| -1.087352273 | 8.20E-09 | 1.02E-07 | 1274.218331 | 1866.188627 | 1305.883942 | 739.9756057 | 771.742083 | 578.0204028 | C1qc |
| -1.690962163 | 9.16E-09 | 1.13E-07 | 130.2326088 | 215.1541358 | 124.0694888 | 43.74240034 | 52.75414459 | 49.07720401 | Hc |
| -1.010256548 | 9.26E-09 | 1.14E-07 | 1303.263014 | 1197.934468 | 1314.295432 | 803.7666062 | 559.8746313 | 527.5799431 | Gpr37 |
| -3.584473777 | 9.34E-09 | 1.15E-07 | 41.22471071 | 46.49517341 | 34.69739941 | 4.556500035 | 0.8508733 | 5.453022668 | Wfikkn2 |
| -1.13032533 | 9.46E-09 | 1.17E-07 | 1614.322194 | 1996.557446 | 1536.148501 | 876.6706068 | 907.0309377 | 563.0245905 | H2-Q4 |
| -1.450849427 | 9.53E-09 | 1.17E-07 | 426.3009857 | 661.8724685 | 365.8998484 | 215.0668017 | 173.5781532 | 141.7785894 | Cd36 |
| -1.118409632 | 9.53E-09 | 1.17E-07 | 480.6426498 | 561.5887612 | 473.1463556 | 258.809202 | 264.6215963 | 170.4069584 | Cyp4f14 |
| -1.043003205 | 1.03E-08 | 1.26E-07 | 611.8121839 | 604.4372543 | 483.6607191 | 297.0838023 | 306.314388 | 218.1209067 | Tgfbr3 |
| -1.182362267 | 1.11E-08 | 1.34E-07 | 6608.133741 | 8505.881724 | 8015.099265 | 4143.681132 | 3729.377673 | 2313.444867 | Lgals1 |
| -1.200992389 | 1.13E-08 | 1.37E-07 | 315.743807 | 427.5732614 | 359.5912303 | 146.7193011 | 198.2534789 | 132.2357997 | 4930523C07Rik |
| -2.58973542 | 1.19E-08 | 1.43E-07 | 53.40473887 | 90.25533662 | 60.98330806 | 17.31470013 | 10.2104796 | 5.453022668 | Omd |
| -1.580897695 | 1.21E-08 | 1.45E-07 | 946.2944958 | 731.1593936 | 955.7556384 | 287.0595022 | 422.88403 | 166.3171914 | Wnt4 |
| -1.21026562 | 1.21E-08 | 1.46E-07 | 338.2300129 | 419.3682308 | 334.356758 | 146.7193011 | 198.2534789 | 124.0562657 | Lbp |
| -1.265480136 | 1.22E-08 | 1.47E-07 | 2094.027919 | 1814.223433 | 2193.296217 | 1017.922108 | 979.3551682 | 535.7594771 | 1700017B05Rik |
| -2.419937084 | 1.22E-08 | 1.47E-07 | 77.7647952 | 44.67183328 | 60.98330806 | 10.02430008 | 11.0613529 | 13.63255667 | Gm2564 |
| -1.599740752 | 1.27E-08 | 1.52E-07 | 95.56637482 | 123.9871291 | 98.83501651 | 31.89550025 | 39.99104509 | 32.71813601 | Itih2 |
| -1.032511755 | 1.32E-08 | 1.57E-07 | 329.7976857 | 413.8982104 | 314.3794674 | 180.4374014 | 181.2360129 | 154.0478904 | Trim21 |
| -1.13340714 | 1.33E-08 | 1.59E-07 | 1056.851675 | 1221.63789 | 1035.664801 | 469.3195036 | 656.8741875 | 380.3483311 | Aebp1 |
| -1.322389471 | 1.34E-08 | 1.59E-07 | 319.491508 | 417.5448906 | 378.5170845 | 173.1470013 | 171.0255333 | 98.15440802 | Fzd2 |
| -1.079705437 | 1.36E-08 | 1.62E-07 | 878.8358783 | 810.4746894 | 901.0809484 | 462.0291036 | 467.9803149 | 290.3734571 | Arrdc4 |
| -1.326310494 | 1.43E-08 | 1.69E-07 | 184.5742729 | 177.775663 | 195.5671603 | 58.32320045 | 93.59606299 | 69.52603902 | Lmx1a |
| -2.506410145 | 1.46E-08 | 1.72E-07 | 57.15243985 | 70.19859515 | 41.00601749 | 10.93560008 | 10.2104796 | 8.179534002 | Gm30191 |
| -1.05467083 | 1.57E-08 | 1.84E-07 | 5057.522464 | 6476.504155 | 4970.139607 | 2806.804022 | 3144.827716 | 1988.990018 | Slc7a2 |
| -1.649476362 | 1.61E-08 | 1.89E-07 | 292.3206759 | 346.4346254 | 261.8076501 | 104.7995008 | 128.4818683 | 50.44045968 | Ror1 |
| -1.377187098 | 1.70E-08 | 1.99E-07 | 11113.80724 | 16337.1276 | 10748.83376 | 5171.62754 | 6355.172677 | 3175.022448 | Cp |
| -1.751905255 | 1.71E-08 | 1.99E-07 | 86.19712239 | 96.63702709 | 105.1436346 | 23.69380018 | 37.43842519 | 23.17534634 | Zbtb42 |
| -1.22434154 | 1.75E-08 | 2.03E-07 | 237.9790118 | 230.6525269 | 242.8817959 | 123.936801 | 103.8065426 | 73.61580602 | Selenbp1 |
| -2.111700424 | 1.75E-08 | 2.03E-07 | 63.71091655 | 64.72857475 | 57.82899902 | 17.31470013 | 11.0613529 | 14.99581234 | Slc6a13 |
| -1.334320252 | 1.84E-08 | 2.12E-07 | 1673.348485 | 1414.911944 | 1840.013605 | 846.5977065 | 698.5669792 | 404.8869331 | Lgi3 |
| -1.321314169 | 1.91E-08 | 2.20E-07 | 237.9790118 | 282.6177207 | 292.2993042 | 103.8882008 | 140.3940945 | 77.70557302 | Mmp11 |
| -2.746484415 | 1.91E-08 | 2.21E-07 | 1892.588992 | 2852.615639 | 1905.202659 | 445.6257034 | 393.9543378 | 149.9581234 | Slco1a4 |
| -1.021185807 | 1.99E-08 | 2.28E-07 | 9328.964648 | 7736.432187 | 9394.58375 | 5023.085639 | 4825.302484 | 3183.201982 | D430041D05Rik |
| -1.537848448 | 2.05E-08 | 2.35E-07 | 337.2930876 | 563.4121013 | 337.511067 | 168.5905013 | 159.9641804 | 95.42789669 | Vgll3 |
| -1.131967337 | 2.09E-08 | 2.39E-07 | 206.1235535 | 169.5706324 | 187.1556696 | 90.2187007 | 80.83296349 | 85.88510702 | Kcns3 |
| -1.193343981 | 2.11E-08 | 2.41E-07 | 498.4442295 | 549.7370503 | 493.1236462 | 273.3900021 | 245.0515104 | 151.321379 | Sqor |
| -1.737341512 | 2.17E-08 | 2.47E-07 | 237.0420866 | 208.7724453 | 238.6760505 | 69.25880053 | 98.70130279 | 34.08139168 | Wnt5b |
| -1.354193385 | 2.27E-08 | 2.57E-07 | 387.8870508 | 289.9110813 | 352.2311759 | 164.0340013 | 148.0519542 | 87.24836269 | Plekhd1 |
| -1.297875002 | 2.33E-08 | 2.63E-07 | 411.3101818 | 510.5352374 | 362.7455393 | 158.5662012 | 228.8849177 | 132.2357997 | Armc3 |
| -1.461346199 | 2.40E-08 | 2.70E-07 | 876.0251025 | 1081.240699 | 1041.973419 | 379.1008029 | 498.6117537 | 207.2148614 | Tspo |
| -1.166086862 | 2.60E-08 | 2.91E-07 | 4322.973073 | 5220.222803 | 4376.078072 | 2606.31802 | 2198.656607 | 1393.247292 | Ccdc85a |
| -1.264006137 | 2.61E-08 | 2.92E-07 | 357.905443 | 597.1438938 | 386.9285753 | 178.6148014 | 210.1657051 | 169.0437027 | Sntb1 |
| -1.005649621 | 2.69E-08 | 3.01E-07 | 4308.919194 | 4307.641066 | 4192.076711 | 2305.589018 | 2542.40942 | 1525.483091 | Nipal3 |
| -1.530413695 | 2.74E-08 | 3.06E-07 | 258.5913672 | 309.9678227 | 249.190414 | 79.28310061 | 136.9906013 | 64.07301635 | Pipox |
| -1.016272768 | 2.74E-08 | 3.06E-07 | 1793.274916 | 1948.238933 | 1783.236043 | 914.9452071 | 1135.064982 | 676.1748108 | Mamdc2 |
| -1.498953398 | 2.77E-08 | 3.09E-07 | 172.3942448 | 185.0690236 | 159.8183246 | 45.56500035 | 84.23645669 | 51.80371535 | Smad6 |
| -1.027114189 | 2.79E-08 | 3.10E-07 | 527.488912 | 456.7467035 | 532.026791 | 284.3256022 | 267.1742162 | 188.129282 | Exosc5 |
| -1.468881102 | 2.85E-08 | 3.16E-07 | 159.2772914 | 184.1573535 | 167.178379 | 62.87970049 | 79.13121689 | 39.53441434 | Gm5069 |
| -1.165059574 | 3.12E-08 | 3.43E-07 | 330.7346109 | 281.7060507 | 316.4823401 | 174.0583013 | 131.0344882 | 106.333942 | Gamt |
| -1.17494277 | 3.29E-08 | 3.60E-07 | 505.0027062 | 627.229006 | 544.6440272 | 209.5990016 | 328.4370938 | 201.7618387 | Xaf1 |
| -1.134132974 | 3.59E-08 | 3.91E-07 | 215.492806 | 229.7408568 | 197.670033 | 92.04130071 | 115.7187688 | 83.15859569 | Nudt7 |
| -1.406456322 | 3.77E-08 | 4.10E-07 | 1363.226229 | 1351.095039 | 1351.095704 | 504.8602039 | 725.7949248 | 298.5529911 | Txnip |
| -1.007991472 | 3.87E-08 | 4.21E-07 | 4125.281847 | 3712.320512 | 4230.979856 | 2079.586616 | 2455.620343 | 1460.046819 | Tspan18 |
| -1.314922088 | 4.24E-08 | 4.56E-07 | 207.997404 | 151.3372311 | 181.8984878 | 86.57350067 | 70.62248389 | 58.61999368 | Fbxl7 |
| -1.835910645 | 4.31E-08 | 4.63E-07 | 108.6833282 | 88.43199649 | 93.57783478 | 36.45200028 | 28.0788189 | 14.99581234 | D330025C20Rik |
| -1.136365315 | 4.32E-08 | 4.63E-07 | 1069.968628 | 1408.530253 | 971.5271836 | 595.9902046 | 585.4008303 | 384.4380981 | Cobll1 |
| -1.219538725 | 4.69E-08 | 4.99E-07 | 566.8397722 | 517.828598 | 650.8390981 | 249.6962019 | 320.7792341 | 170.4069584 | Extl1 |
| -1.11242237 | 4.88E-08 | 5.16E-07 | 298.8791526 | 309.0561527 | 309.1222857 | 136.6950011 | 176.1307731 | 107.6971977 | Dok1 |
| -1.515471672 | 4.97E-08 | 5.26E-07 | 306.3745546 | 378.3430777 | 243.9332322 | 113.0012009 | 142.0958411 | 66.79952768 | Etfbkmt |
| -1.14834434 | 5.21E-08 | 5.49E-07 | 1279.839882 | 1644.652801 | 1291.163833 | 673.4507052 | 783.6543092 | 440.3315804 | Tmem123 |
| -1.199456107 | 5.25E-08 | 5.52E-07 | 483.4534256 | 382.9014281 | 471.043483 | 249.6962019 | 188.8938726 | 140.4153337 | Frat2 |
| -1.393772227 | 5.26E-08 | 5.54E-07 | 149.9080389 | 201.4790848 | 168.2298153 | 79.28310061 | 69.77161059 | 46.35069268 | E030013I19Rik |
| -1.208715004 | 5.27E-08 | 5.55E-07 | 609.9383334 | 648.1974175 | 557.2612633 | 220.5346017 | 359.9194059 | 201.7618387 | Tfcp2l1 |
| -1.29542307 | 5.28E-08 | 5.55E-07 | 245.4744138 | 320.9078635 | 290.1964315 | 139.4289011 | 128.4818683 | 77.70557302 | Plscr1 |
| -1.155918966 | 5.59E-08 | 5.86E-07 | 544.3535664 | 580.7338326 | 526.7696093 | 305.2855024 | 265.4724696 | 166.3171914 | Cdk18 |
| -1.130090474 | 5.75E-08 | 6.01E-07 | 267.0236944 | 389.2831185 | 287.0421224 | 161.3001012 | 131.8853615 | 137.6888224 | 4932438H23Rik |
| -1.41327913 | 5.90E-08 | 6.15E-07 | 362.5900692 | 330.0245642 | 364.848412 | 123.0255009 | 187.192126 | 83.15859569 | Sult1a1 |
| -1.233377227 | 6.01E-08 | 6.26E-07 | 237.9790118 | 327.289554 | 242.8817959 | 103.8882008 | 141.2449678 | 96.79115236 | Tshr |
| -1.200455195 | 6.48E-08 | 6.71E-07 | 475.0210984 | 499.5951966 | 401.6486841 | 162.2114013 | 268.0250895 | 166.3171914 | Il17rc |
| -1.005449478 | 6.52E-08 | 6.75E-07 | 680.2077267 | 793.1529582 | 625.6046258 | 387.302503 | 389.6999713 | 264.4715994 | Ocln |
| -1.099983411 | 6.60E-08 | 6.82E-07 | 267.0236944 | 313.614503 | 249.190414 | 129.404601 | 151.4554474 | 103.6074307 | Kctd11 |
| -1.222374126 | 6.65E-08 | 6.87E-07 | 362.5900692 | 470.4217545 | 339.6139397 | 177.7035014 | 201.6569721 | 119.9664987 | Cep128 |
| -1.08367997 | 6.80E-08 | 7.00E-07 | 1424.12637 | 1808.753413 | 1368.970122 | 858.4446066 | 787.9086757 | 520.7636648 | Laptm5 |
| -1.031858946 | 6.87E-08 | 7.07E-07 | 633.3614645 | 751.2161351 | 677.1250067 | 308.0194024 | 427.9892698 | 268.5613664 | Asap3 |
| -1.045244858 | 6.91E-08 | 7.10E-07 | 633.3614645 | 798.6229786 | 588.8043537 | 342.6488026 | 379.4894917 | 253.5655541 | Aga |
| -1.075040359 | 7.19E-08 | 7.36E-07 | 275.4560216 | 319.9961935 | 301.7622313 | 175.8809014 | 129.3327416 | 118.603243 | Plcg2 |
| -1.151628152 | 7.49E-08 | 7.63E-07 | 405.6886304 | 505.065217 | 433.1917745 | 213.2442016 | 245.9023837 | 141.7785894 | Tead3 |
| -1.119725894 | 8.11E-08 | 8.22E-07 | 199.5650768 | 243.4159079 | 195.5671603 | 92.04130071 | 113.1661489 | 87.24836269 | Prr5l |
| -1.051001218 | 8.20E-08 | 8.30E-07 | 1703.330092 | 2397.692276 | 1586.617446 | 993.3170077 | 1019.346213 | 729.3417818 | C1qa |
| -1.69829042 | 8.44E-08 | 8.53E-07 | 92.75559909 | 148.6022209 | 115.657998 | 45.56500035 | 38.28929849 | 24.53860201 | Gm17455 |
| -1.763737125 | 8.55E-08 | 8.64E-07 | 105.8725525 | 186.8923637 | 109.34938 | 41.00850032 | 29.7805655 | 49.07720401 | Sostdc1 |
| -1.092944451 | 9.25E-08 | 9.29E-07 | 1187.084283 | 1798.725042 | 1156.57998 | 724.4835056 | 687.5056263 | 527.5799431 | C3ar1 |
| -1.304538721 | 9.44E-08 | 9.47E-07 | 120.8633564 | 110.3120781 | 131.4295432 | 51.9441004 | 50.20152469 | 43.62418134 | Cdc42ep3 |
| -2.621726351 | 9.47E-08 | 9.50E-07 | 42.16163595 | 51.05352374 | 44.16032653 | 10.02430008 | 8.508732999 | 2.726511334 | Gm37800 |
| -1.736370534 | 9.55E-08 | 9.57E-07 | 82.44942142 | 154.0722413 | 110.4008163 | 34.62940027 | 39.99104509 | 28.62836901 | Col15a1 |
| -1.438840794 | 9.70E-08 | 9.70E-07 | 112.4310292 | 143.1322005 | 124.0694888 | 56.50060044 | 47.64890479 | 34.08139168 | Plpp2 |
| -1.245792255 | 9.80E-08 | 9.79E-07 | 2050.929358 | 1266.309723 | 2030.323584 | 865.7350067 | 855.1276664 | 530.3064545 | Coq8a |
| -1.815600085 | 1.00E-07 | 9.99E-07 | 73.08016898 | 87.52032642 | 63.08618075 | 17.31470013 | 21.2718325 | 25.90185767 | Galnt12 |
| -1.359795131 | 1.04E-07 | 1.03E-06 | 1868.228935 | 1435.880355 | 1871.556696 | 716.2818055 | 901.9256979 | 393.9808878 | Chrna4 |
| -1.038181575 | 1.09E-07 | 1.08E-06 | 2378.853193 | 3064.123095 | 2277.411125 | 1572.903812 | 1219.301439 | 963.8217566 | Plin2 |
| -1.15429107 | 1.20E-07 | 1.19E-06 | 622.1183616 | 922.6101077 | 628.7589348 | 389.125103 | 335.2440802 | 249.4757871 | Tyrobp |
| -1.446594872 | 1.23E-07 | 1.22E-06 | 1780.157962 | 3438.819492 | 1618.160536 | 1013.365608 | 850.0224266 | 643.4566748 | Sema3c |
| -1.039862782 | 1.36E-07 | 1.33E-06 | 182.7004225 | 179.5990032 | 187.1556696 | 86.57350067 | 98.70130279 | 80.43208435 | Gypc |
| -1.047377028 | 1.41E-07 | 1.37E-06 | 4826.101929 | 3948.44306 | 5092.106223 | 2836.876922 | 2242.902018 | 1625.000755 | Kirrel3 |
| -1.607395814 | 1.41E-07 | 1.37E-06 | 73.08016898 | 88.43199649 | 80.96059863 | 23.69380018 | 29.7805655 | 25.90185767 | Tjp3 |
| -1.003622289 | 1.43E-07 | 1.40E-06 | 306.3745546 | 441.2483124 | 368.0027211 | 193.1956015 | 196.5517323 | 164.9539357 | Rrm2 |
| -1.001803839 | 1.51E-07 | 1.46E-06 | 967.8437764 | 1055.713937 | 1039.870546 | 497.5698038 | 648.3654545 | 378.9850754 | Plekhg2 |
| -1.008482026 | 1.54E-07 | 1.50E-06 | 385.076275 | 401.1348294 | 358.5397939 | 228.7363018 | 192.2973658 | 144.5051007 | Zfp503 |
| -3.42469554 | 1.59E-07 | 1.54E-06 | 30.91853303 | 138.5738502 | 39.95458114 | 9.11300007 | 5.956113099 | 4.089767001 | Tc2n |
| -1.192031914 | 1.59E-07 | 1.54E-06 | 756.0986714 | 948.1368695 | 709.7195335 | 384.568603 | 434.7962562 | 233.1167191 | Ccdc40 |
| -1.129046605 | 1.61E-07 | 1.56E-06 | 324.1761342 | 363.7563567 | 284.9392497 | 164.0340013 | 171.0255333 | 106.333942 | Akip1 |
| -1.906955555 | 1.66E-07 | 1.60E-06 | 59.96321558 | 72.93360535 | 53.62325364 | 15.49210012 | 17.8683393 | 16.359068 | Crygs |
| -1.086932524 | 1.69E-07 | 1.62E-06 | 2283.286818 | 2012.055838 | 2229.045053 | 987.8492076 | 1358.84466 | 719.7989922 | Sun2 |
| -2.19105645 | 1.71E-07 | 1.64E-06 | 8395.787106 | 7839.450905 | 8668.041235 | 2264.580517 | 2298.208783 | 888.8426949 | Fmod |
| -1.482185124 | 1.78E-07 | 1.70E-06 | 873.2143268 | 850.5881724 | 976.7843653 | 408.2624032 | 394.8052111 | 159.500913 | Cd34 |
| -1.084058241 | 1.79E-07 | 1.71E-06 | 4680.878516 | 4820.911314 | 4823.989955 | 2268.225718 | 2958.486464 | 1525.483091 | Foxj1 |
| -2.817073149 | 1.81E-07 | 1.72E-06 | 31.85545827 | 37.37847274 | 55.72612633 | 5.467800042 | 7.657859699 | 4.089767001 | Foxl1 |
| -1.274882096 | 1.96E-07 | 1.85E-06 | 171.4573195 | 130.3688196 | 140.8924703 | 69.25880053 | 62.96462419 | 49.07720401 | Htr3a |
| -1.494654762 | 1.99E-07 | 1.88E-06 | 100.251001 | 104.8420577 | 105.1436346 | 39.1859003 | 44.24541159 | 24.53860201 | Cutal |
| -1.152568707 | 2.08E-07 | 1.95E-06 | 906.0067103 | 1471.435488 | 884.2579669 | 540.4009042 | 526.6905726 | 398.0706548 | Dab2 |
| -1.26177881 | 2.09E-07 | 1.96E-06 | 323.239209 | 303.5861323 | 298.6079222 | 173.1470013 | 125.0783751 | 84.52185135 | Ldlrap1 |
| -2.238918106 | 2.29E-07 | 2.13E-06 | 3385.110904 | 3772.490737 | 3424.528179 | 868.4689067 | 1014.240973 | 357.1729848 | Bmp6 |
| -1.307144116 | 2.31E-07 | 2.15E-06 | 1030.617768 | 1626.419399 | 1041.973419 | 535.8444041 | 627.093622 | 328.5446157 | Iqcg |
| -1.095543836 | 2.34E-07 | 2.17E-06 | 1055.914749 | 1042.038886 | 1081.928 | 472.0534036 | 663.6811739 | 347.6301951 | Rflnb |
| -1.039162661 | 2.38E-07 | 2.21E-06 | 700.820082 | 918.0517573 | 712.8738425 | 402.7946031 | 448.410229 | 279.4674117 | Fas |
| -1.278609553 | 2.48E-07 | 2.29E-06 | 135.8541603 | 177.775663 | 126.1723615 | 67.43620052 | 55.30676449 | 58.61999368 | Acat3 |
| -1.097095945 | 2.78E-07 | 2.54E-06 | 221.1143574 | 255.2676187 | 228.1616871 | 111.1786009 | 131.8853615 | 83.15859569 | Zbtb7c |
| -1.240399777 | 2.80E-07 | 2.55E-06 | 273.5821711 | 378.3430777 | 240.7789232 | 146.7193011 | 133.5871081 | 95.42789669 | Uba7 |
| -2.420661845 | 2.82E-07 | 2.57E-06 | 1323.875369 | 1225.28457 | 1422.593376 | 383.657303 | 240.7971439 | 115.8767317 | Slc30a3 |
| -1.157897248 | 2.87E-07 | 2.61E-06 | 177.078871 | 197.8324045 | 202.9272148 | 70.17010054 | 107.2100358 | 80.43208435 | Slc12a8 |
| -1.032117203 | 3.01E-07 | 2.73E-06 | 1568.412857 | 1556.220804 | 1518.274083 | 740.8869057 | 988.7147745 | 535.7594771 | Plekha7 |
| -1.240676618 | 3.04E-07 | 2.75E-06 | 860.0973734 | 852.4115125 | 848.5091311 | 328.9793025 | 514.7783464 | 235.8432304 | Efemp1 |
| -1.208393102 | 3.62E-07 | 3.24E-06 | 596.82138 | 754.8628154 | 493.1236462 | 306.1968024 | 308.8670079 | 179.949748 | Armc2 |
| -1.224254745 | 3.62E-07 | 3.25E-06 | 561.2182208 | 966.3702709 | 566.7241904 | 318.9550025 | 336.9458268 | 238.5697417 | Iqub |
| -1.252387404 | 3.76E-07 | 3.36E-06 | 349.4731158 | 551.5603905 | 329.0995763 | 193.1956015 | 188.8938726 | 132.2357997 | Efcab12 |
| -1.12672954 | 3.97E-07 | 3.52E-06 | 514.3719586 | 731.1593936 | 577.2385539 | 262.454402 | 359.0685326 | 209.9413727 | Cfap52 |
| -2.036375118 | 4.08E-07 | 3.61E-06 | 194.8804506 | 160.4539318 | 179.7956151 | 62.87970049 | 52.75414459 | 12.269301 | Pdzd3 |
| -1.782119797 | 4.12E-07 | 3.64E-06 | 72.14324374 | 73.84527542 | 87.26921671 | 26.4277002 | 27.2279456 | 12.269301 | Trabd2b |
| -1.310877709 | 4.19E-07 | 3.69E-06 | 413.1840323 | 701.0742814 | 377.4656482 | 193.1956015 | 241.6480172 | 164.9539357 | Gulp1 |
| -1.298856026 | 4.27E-07 | 3.76E-06 | 1329.49692 | 2276.440157 | 1331.118414 | 829.2830064 | 713.8826986 | 460.7804154 | Folh1 |
| -1.379446021 | 4.90E-07 | 4.25E-06 | 272.6452458 | 315.4378431 | 240.7789232 | 128.493301 | 126.7801217 | 59.98324935 | Arhgef5 |
| -1.686719933 | 4.91E-07 | 4.26E-06 | 72.14324374 | 82.96197608 | 76.75485325 | 28.25030022 | 16.1665927 | 28.62836901 | Glns-ps1 |
| -1.538815142 | 4.93E-07 | 4.27E-06 | 101.1879263 | 162.2772719 | 103.0407619 | 43.74240034 | 48.49977809 | 32.71813601 | Ankub1 |
| -1.186857628 | 5.05E-07 | 4.37E-06 | 257.6544419 | 416.6332206 | 252.344723 | 140.3402011 | 139.5432212 | 126.782777 | Abcc12 |
| -1.377039077 | 5.29E-07 | 4.56E-06 | 122.7372069 | 123.075459 | 119.8637434 | 54.67800042 | 53.60501789 | 29.99162467 | Six5 |
| -1.500397775 | 5.62E-07 | 4.82E-06 | 1265.786004 | 1386.650172 | 1136.60269 | 677.0959052 | 433.9453829 | 224.9371851 | Rspo3 |
| -3.597390705 | 5.78E-07 | 4.94E-06 | 21.5492806 | 60.17022441 | 26.28590865 | 2.733900021 | 3.4034932 | 2.726511334 | Sult1c1 |
| -2.394735021 | 5.98E-07 | 5.10E-06 | 63.71091655 | 140.3971903 | 76.75485325 | 29.16160023 | 16.1665927 | 6.816278335 | Tmem252 |
| -1.961808427 | 6.05E-07 | 5.16E-06 | 47.78318741 | 62.90523461 | 60.98330806 | 17.31470013 | 16.1665927 | 9.542789669 | Vnn1 |
| -1.203456816 | 6.19E-07 | 5.26E-06 | 120.8633564 | 131.2804896 | 139.841034 | 62.87970049 | 60.41200429 | 44.98743701 | Gpc3 |
| -1.038051077 | 6.20E-07 | 5.27E-06 | 344.7884896 | 489.5668259 | 382.7228299 | 236.0267018 | 194.8499857 | 159.500913 | Renbp |
| -1.003008921 | 6.23E-07 | 5.30E-06 | 277.329872 | 371.9613873 | 293.3507405 | 179.5261014 | 159.1133071 | 129.5092884 | Parvb |
| -1.153156818 | 6.32E-07 | 5.36E-06 | 126.4849079 | 144.9555406 | 119.8637434 | 55.58930043 | 59.56113099 | 61.34650502 | Bcl2l12 |
| -1.158604371 | 6.43E-07 | 5.45E-06 | 215.492806 | 260.7376391 | 212.3901419 | 102.0656008 | 128.4818683 | 74.97906169 | Dynlrb2 |
| -1.39917124 | 6.51E-07 | 5.52E-06 | 2109.018723 | 1461.407117 | 2096.564074 | 917.6791071 | 846.6189334 | 380.3483311 | Arhgef28 |
| -1.12491359 | 6.62E-07 | 5.61E-06 | 228.6097594 | 337.3179247 | 281.7849407 | 124.848101 | 156.5606872 | 104.9706864 | Zmynd10 |
| -1.184779338 | 6.65E-07 | 5.63E-06 | 1914.138272 | 3165.318472 | 2027.169275 | 1376.063011 | 934.2588833 | 813.8636332 | Mmp12 |
| -1.894859435 | 6.72E-07 | 5.68E-06 | 151.7818894 | 130.3688196 | 129.3266705 | 45.56500035 | 50.20152469 | 12.269301 | Rtl3 |
| -1.168555631 | 6.79E-07 | 5.73E-06 | 144.2864875 | 169.5706324 | 133.5324159 | 77.4605006 | 58.71025769 | 62.70976068 | Adamtsl5 |
| -1.445746048 | 7.00E-07 | 5.88E-06 | 95.56637482 | 154.0722413 | 123.0180525 | 54.67800042 | 44.24541159 | 36.80790301 | Gm1110 |
| -2.779214768 | 7.58E-07 | 6.33E-06 | 57.15243985 | 66.55191488 | 55.72612633 | 5.467800042 | 17.8683393 | 1.363255667 | Arhgef16 |
| -1.608168704 | 7.67E-07 | 6.40E-06 | 504.0657809 | 383.8130981 | 535.1811 | 176.7922014 | 216.9726915 | 69.52603902 | Eln |
| -1.584604176 | 7.68E-07 | 6.40E-06 | 73.08016898 | 112.1354182 | 80.96059863 | 33.71810026 | 29.7805655 | 24.53860201 | Klhl6 |
| -4.557415814 | 7.90E-07 | 6.58E-06 | 19.67543011 | 51.96519381 | 24.18303596 | 0 | 1.7017466 | 2.726511334 | Wdr86 |
| -1.342971615 | 7.93E-07 | 6.60E-06 | 476.8949489 | 747.5694548 | 443.706138 | 211.4216016 | 293.5512885 | 149.9581234 | Sytl2 |
| -1.324656426 | 8.80E-07 | 7.23E-06 | 383.2024245 | 438.5133022 | 351.1797395 | 227.8250018 | 136.9906013 | 100.8809194 | Tlr2 |
| -1.578947164 | 9.78E-07 | 7.98E-06 | 164.8988428 | 167.7472923 | 121.9666161 | 43.74240034 | 74.87685039 | 31.35488034 | Rd3 |
| -1.083887766 | 9.98E-07 | 8.14E-06 | 566.8397722 | 533.3269891 | 550.9526452 | 199.5747015 | 357.366786 | 219.4841624 | Slc16a9 |
| -1.22720316 | 1.03E-06 | 8.39E-06 | 269.8344701 | 319.9961935 | 297.5564859 | 168.5905013 | 126.7801217 | 80.43208435 | Trim59 |
| -1.501888222 | 1.08E-06 | 8.73E-06 | 281.077573 | 240.6808977 | 275.4763226 | 86.57350067 | 141.2449678 | 50.44045968 | Rln1 |
| -1.083506777 | 1.12E-06 | 9.03E-06 | 767.3417743 | 985.5153423 | 778.0628959 | 535.8444041 | 362.4720258 | 293.0999684 | Zc3hav1 |
| -1.060143259 | 1.14E-06 | 9.20E-06 | 855.4127472 | 641.8157271 | 908.4410028 | 431.0449033 | 448.410229 | 269.9246221 | Gpr12 |
| -1.293156682 | 1.18E-06 | 9.48E-06 | 369.1485459 | 321.8195336 | 357.4883576 | 170.4131013 | 174.4290265 | 79.06882869 | Pxmp2 |
| -1.220212652 | 1.22E-06 | 9.77E-06 | 140.5387865 | 167.7472923 | 138.7895977 | 79.28310061 | 62.96462419 | 47.71394835 | Gm20383 |
| -1.047581953 | 1.22E-06 | 9.79E-06 | 148.9711137 | 185.9806936 | 151.4068338 | 73.81530057 | 79.13121689 | 83.15859569 | Entpd2 |
| -1.507308367 | 1.24E-06 | 9.90E-06 | 104.9356273 | 103.0187176 | 124.0694888 | 47.38760037 | 45.09628489 | 21.81209067 | Usp43 |
| -1.237553767 | 1.27E-06 | 1.01E-05 | 871.3404763 | 1452.290417 | 894.7723303 | 412.8189032 | 610.076156 | 339.4506611 | Ifit3b |
| -1.197808377 | 1.30E-06 | 1.03E-05 | 128.3587583 | 136.75051 | 103.0407619 | 50.12150039 | 56.15763779 | 54.53022668 | Ugt1a7c |
| -2.367691236 | 1.32E-06 | 1.05E-05 | 37819.92437 | 54127.67521 | 38065.15003 | 8387.605265 | 12991.13354 | 3810.299589 | C3 |
| -1.208263893 | 1.32E-06 | 1.05E-05 | 200.5020021 | 210.5957854 | 164.02407 | 97.50910075 | 92.74518969 | 55.89348235 | Tcf19 |
| -1.28258191 | 1.35E-06 | 1.07E-05 | 104.9356273 | 129.4571495 | 105.1436346 | 40.09720031 | 55.30676449 | 43.62418134 | Gm29157 |
| -2.401235712 | 1.37E-06 | 1.08E-05 | 37.47700973 | 70.19859515 | 34.69739941 | 8.201700063 | 9.359606299 | 9.542789669 | Slc6a20a |
| -1.031889873 | 1.39E-06 | 1.09E-05 | 837.6111676 | 645.4624073 | 871.6407307 | 487.5455038 | 379.4894917 | 280.8306674 | Rcor2 |
| -1.534381876 | 1.39E-06 | 1.10E-05 | 146.160338 | 131.2804896 | 152.4582702 | 33.71810026 | 74.87685039 | 38.17115868 | Slc22a18 |
| -1.174321791 | 1.40E-06 | 1.10E-05 | 738.2970918 | 897.0833458 | 785.4229504 | 502.1263039 | 332.6914603 | 234.4799747 | Rsad2 |
| -1.062705032 | 1.44E-06 | 1.14E-05 | 161.1511419 | 154.9839114 | 169.2812517 | 92.95260072 | 68.92073729 | 69.52603902 | Cdc45 |
| -1.607542709 | 1.51E-06 | 1.19E-05 | 128.3587583 | 183.2456834 | 162.9726336 | 79.28310061 | 32.3331854 | 43.62418134 | Cdk1 |
| -1.062707446 | 1.53E-06 | 1.20E-05 | 341.0407886 | 512.3585776 | 350.1283032 | 227.8250018 | 187.192126 | 159.500913 | Sgk3 |
| -1.02156334 | 1.55E-06 | 1.21E-05 | 686.7662034 | 990.0736926 | 641.376171 | 418.2867032 | 423.7349033 | 297.1897354 | Parp14 |
| -3.35341658 | 1.56E-06 | 1.22E-05 | 698.0093063 | 1260.839702 | 725.4910787 | 160.3888012 | 71.47335719 | 29.99162467 | Aqp1 |
| -1.041250389 | 1.59E-06 | 1.24E-05 | 171.4573195 | 232.475867 | 206.0815238 | 85.66220066 | 112.3152756 | 98.15440802 | Itpripl1 |
| -1.046577443 | 1.67E-06 | 1.30E-05 | 358.8423682 | 584.3805129 | 381.6713936 | 236.0267018 | 198.2534789 | 207.2148614 | Stk17b |
| -1.056622665 | 1.68E-06 | 1.30E-05 | 1028.743917 | 898.9066859 | 1019.893255 | 585.0546045 | 523.2870794 | 304.0060137 | Grm1 |
| -1.014419896 | 1.70E-06 | 1.32E-05 | 229.5466846 | 285.3527309 | 280.7335043 | 118.4690009 | 162.5168003 | 110.423709 | 9530053A07Rik |
| -1.219312559 | 1.79E-06 | 1.38E-05 | 250.15904 | 386.5481083 | 226.0588144 | 123.936801 | 145.4993343 | 99.51766369 | Gm38562 |
| -1.096345034 | 1.79E-06 | 1.38E-05 | 194.8804506 | 172.3056426 | 230.2645597 | 87.48480068 | 113.1661489 | 76.34231735 | Trim7 |
| -1.643843326 | 1.91E-06 | 1.46E-05 | 54.34166412 | 68.37525501 | 60.98330806 | 20.04860015 | 19.5700859 | 19.08557934 | Slco2b1 |
| -1.001136395 | 1.96E-06 | 1.50E-05 | 266.0867691 | 371.0497172 | 308.0708493 | 172.2357013 | 174.4290265 | 122.69301 | Fam151b |
| -1.114404825 | 2.00E-06 | 1.53E-05 | 163.0249923 | 207.8607752 | 164.02407 | 89.30740069 | 92.74518969 | 62.70976068 | 2610035F20Rik |
| -1.112844336 | 2.17E-06 | 1.65E-05 | 204.2497031 | 279.8827105 | 198.7214694 | 123.0255009 | 108.9117824 | 81.79534002 | Pifo |
| -1.034065177 | 2.19E-06 | 1.66E-05 | 437.5440887 | 533.3269891 | 461.5805558 | 268.8335021 | 269.7268361 | 156.7744017 | Ascl1 |
| -1.084585978 | 2.25E-06 | 1.70E-05 | 236.1051613 | 288.0877411 | 266.0133955 | 146.7193011 | 137.8414746 | 84.52185135 | Zc3h12a |
| -1.129410212 | 2.38E-06 | 1.80E-05 | 122.7372069 | 144.9555406 | 128.2752342 | 71.08140055 | 53.60501789 | 55.89348235 | Fzd10 |
| -1.115167298 | 2.40E-06 | 1.81E-05 | 126.4849079 | 123.075459 | 129.3266705 | 53.76670042 | 68.06986399 | 51.80371535 | Gm16576 |
| -1.428084143 | 2.45E-06 | 1.84E-05 | 111.494104 | 178.6873331 | 97.78358017 | 51.9441004 | 45.94715819 | 46.35069268 | C530044C16Rik |
| -1.433667464 | 2.72E-06 | 2.03E-05 | 73.08016898 | 70.19859515 | 84.11490767 | 30.98420024 | 24.6753257 | 28.62836901 | Gm12407 |
| -1.01860492 | 2.76E-06 | 2.05E-05 | 203.3127778 | 199.6557446 | 192.4128513 | 114.8238009 | 103.8065426 | 72.25255035 | Zeb2os |
| -1.365752604 | 2.92E-06 | 2.15E-05 | 131.1695341 | 171.3939726 | 117.7608707 | 71.08140055 | 39.14017179 | 53.16697101 | Phf19 |
| -1.41178158 | 2.92E-06 | 2.16E-05 | 442.2287149 | 591.6738734 | 380.6199572 | 214.1555017 | 222.9288046 | 91.33812969 | Bbox1 |
| -2.177793388 | 3.04E-06 | 2.24E-05 | 35.60315925 | 38.29014281 | 43.10889018 | 7.290400056 | 10.2104796 | 8.179534002 | A730036I17Rik |
| -1.005462244 | 3.19E-06 | 2.34E-05 | 353.2208167 | 433.9549518 | 330.1510126 | 191.3730015 | 225.4814245 | 136.3255667 | Gm11992 |
| -1.031306612 | 3.37E-06 | 2.46E-05 | 1911.327496 | 2702.190078 | 1974.597458 | 1449.878311 | 936.8115032 | 834.3124682 | Lgals3 |
| -1.311935961 | 3.44E-06 | 2.51E-05 | 460.9672197 | 833.2664411 | 555.1583906 | 251.5188019 | 330.9897137 | 159.500913 | Cx3cr1 |
| -1.226767085 | 3.46E-06 | 2.52E-05 | 424.4271352 | 595.3205537 | 397.4429387 | 230.5589018 | 247.6041303 | 124.0562657 | Fbxo32 |
| -1.025032259 | 3.56E-06 | 2.59E-05 | 159.2772914 | 165.0122821 | 196.6185967 | 82.01700063 | 99.55217609 | 72.25255035 | Lypd6 |
| -1.311622541 | 3.64E-06 | 2.64E-05 | 1485.026511 | 1227.10791 | 1478.319502 | 694.4106054 | 708.7774588 | 280.8306674 | Kcng1 |
| -1.043291141 | 3.64E-06 | 2.64E-05 | 185.5111982 | 163.188942 | 179.7956151 | 81.10570063 | 104.6574159 | 68.16278335 | Wisp1 |
| -1.167283303 | 3.69E-06 | 2.67E-05 | 3968.815331 | 5117.204085 | 3629.558266 | 2615.43102 | 1874.47388 | 1168.310107 | Dock10 |
| -1.614380424 | 3.86E-06 | 2.78E-05 | 133.0433846 | 100.2837074 | 139.841034 | 59.23450046 | 39.99104509 | 20.44883501 | 5330429C05Rik |
| -3.145787369 | 3.86E-06 | 2.78E-05 | 1194.579685 | 1248.076322 | 1200.740307 | 241.4945019 | 131.0344882 | 38.17115868 | Htr1a |
| -1.129673747 | 3.88E-06 | 2.79E-05 | 276.3929468 | 357.3746662 | 222.9045053 | 151.2758012 | 141.2449678 | 96.79115236 | Apbb1ip |
| -1.015680149 | 4.01E-06 | 2.88E-05 | 340.1038633 | 327.289554 | 335.4081943 | 181.3487014 | 199.9552255 | 110.423709 | Rftn1 |
| -2.835124101 | 4.18E-06 | 2.98E-05 | 35.60315925 | 41.93682308 | 30.49165403 | 5.467800042 | 8.508732999 | 0 | Chrnb4 |
| -1.185779404 | 4.29E-06 | 3.06E-05 | 147.0972632 | 180.5106732 | 176.6413061 | 74.72660058 | 93.59606299 | 50.44045968 | Dach1 |
| -1.234186899 | 4.54E-06 | 3.21E-05 | 699.8831568 | 568.8821217 | 780.1657686 | 280.6804022 | 411.8226771 | 174.4967254 | Scn5a |
| -1.18940406 | 4.62E-06 | 3.27E-05 | 1020.31159 | 670.9891692 | 892.6694576 | 449.2709035 | 453.5154688 | 226.3004407 | Ypel1 |
| -1.741808201 | 4.76E-06 | 3.35E-05 | 91.81867385 | 57.43521421 | 70.44623517 | 24.60510019 | 27.2279456 | 12.269301 | Gm5345 |
| -1.047405114 | 5.65E-06 | 3.91E-05 | 712.063185 | 514.1819177 | 826.4289679 | 371.8104029 | 380.340365 | 237.2064861 | Mfap4 |
| -1.019672532 | 5.88E-06 | 4.04E-05 | 238.9159371 | 203.3024249 | 203.9786511 | 90.2187007 | 135.2888547 | 91.33812969 | Tmem53 |
| -1.699581295 | 5.97E-06 | 4.10E-05 | 47.78318741 | 54.70020401 | 53.62325364 | 18.22600014 | 13.6139728 | 16.359068 | Gm5860 |
| -1.031048067 | 6.13E-06 | 4.20E-05 | 238.9159371 | 240.6808977 | 233.4188688 | 133.049801 | 134.4379814 | 77.70557302 | 6430584L05Rik |
| -1.331973824 | 7.37E-06 | 4.97E-05 | 123.6741321 | 133.1038298 | 111.4522527 | 65.61360051 | 48.49977809 | 29.99162467 | 4930526F13Rik |
| -1.830723847 | 7.47E-06 | 5.03E-05 | 59.96321558 | 72.02193528 | 53.62325364 | 13.66950011 | 26.3770723 | 10.90604534 | Hspb2 |
| -1.125807516 | 7.49E-06 | 5.04E-05 | 158.3403661 | 175.9523229 | 146.1496521 | 73.81530057 | 91.89431639 | 51.80371535 | Akr1c13 |
| -1.101924574 | 7.70E-06 | 5.17E-05 | 463.7779955 | 577.0871523 | 481.5578464 | 225.0911017 | 325.0336006 | 155.411146 | Dnali1 |
| -1.135416618 | 7.84E-06 | 5.25E-05 | 609.9383334 | 525.1219585 | 732.8511331 | 247.8736019 | 402.4630708 | 196.308816 | Pdyn |
| -1.454065155 | 7.85E-06 | 5.25E-05 | 60.90014082 | 70.19859515 | 87.26921671 | 30.07290023 | 24.6753257 | 24.53860201 | Msx2 |
| -1.331446693 | 7.96E-06 | 5.32E-05 | 180.826572 | 118.5171087 | 206.0815238 | 57.41190044 | 88.49082319 | 53.16697101 | Lgr5 |
| -1.371638807 | 8.05E-06 | 5.37E-05 | 120.8633564 | 190.539044 | 124.0694888 | 74.72660058 | 41.69279169 | 51.80371535 | Cd33 |
| -1.573115782 | 8.06E-06 | 5.38E-05 | 105.8725525 | 126.7221393 | 116.7094344 | 56.50060044 | 40.84191839 | 17.72232367 | Fibin |
| -1.58153325 | 8.07E-06 | 5.39E-05 | 249.2221147 | 347.3462955 | 256.5504684 | 111.1786009 | 132.7362348 | 38.17115868 | Sync |
| -2.344700501 | 8.49E-06 | 5.64E-05 | 37.47700973 | 85.69698628 | 37.85170845 | 10.93560008 | 14.4648461 | 5.453022668 | Inmt |
| -2.113792075 | 8.51E-06 | 5.65E-05 | 61.83706606 | 61.99356455 | 71.49767152 | 20.95990016 | 19.5700859 | 2.726511334 | Spag17os |
| -1.257567404 | 8.71E-06 | 5.77E-05 | 115.2418049 | 84.78531622 | 103.0407619 | 48.29890037 | 44.24541159 | 32.71813601 | Oprd1 |
| -2.150178313 | 8.81E-06 | 5.82E-05 | 50.59396314 | 97.54869715 | 41.00601749 | 20.04860015 | 13.6139728 | 8.179534002 | A730049H05Rik |
| -2.17750024 | 9.06E-06 | 5.96E-05 | 37.47700973 | 51.96519381 | 41.00601749 | 9.11300007 | 14.4648461 | 4.089767001 | Npy2r |
| -1.130016537 | 9.16E-06 | 6.02E-05 | 204.2497031 | 330.0245642 | 203.9786511 | 113.0012009 | 130.1836149 | 92.70138536 | Gm4876 |
| -5.19821864 | 9.25E-06 | 6.08E-05 | 18.73850487 | 34.64346254 | 18.92585423 | 0.911300007 | 0.8508733 | 0 | Ins2 |
| -1.04989925 | 9.27E-06 | 6.08E-05 | 131.1695341 | 169.5706324 | 130.3781069 | 76.54920059 | 70.62248389 | 59.98324935 | Oas1a |
| -1.614944623 | 9.47E-06 | 6.21E-05 | 66.52169228 | 64.72857475 | 51.52038095 | 14.58080011 | 24.6753257 | 20.44883501 | Sec14l4 |
| -1.236445655 | 9.54E-06 | 6.24E-05 | 319.491508 | 567.0587816 | 271.2705772 | 167.6792013 | 186.3412527 | 136.3255667 | Pdgfd |
| -1.178025245 | 9.61E-06 | 6.28E-05 | 163.0249923 | 197.8324045 | 184.0013605 | 98.42040076 | 90.19256979 | 49.07720401 | Rbpms |
| -1.293117993 | 1.04E-05 | 6.74E-05 | 106.8094777 | 188.7157038 | 125.1209252 | 67.43620052 | 54.45589119 | 49.07720401 | Cd37 |
| -3.157504008 | 1.08E-05 | 6.97E-05 | 1792.337991 | 2054.904331 | 1761.155879 | 275.2126021 | 304.6126414 | 47.71394835 | Wisp2 |
| -1.114026532 | 1.13E-05 | 7.29E-05 | 1016.563889 | 1280.896444 | 1100.853854 | 762.7581059 | 462.0242018 | 342.1771724 | Capg |
| -1.170500211 | 1.18E-05 | 7.57E-05 | 263.2759934 | 454.9233634 | 252.344723 | 174.9696014 | 130.1836149 | 125.4195214 | Col4a4 |
| -1.07128569 | 1.25E-05 | 7.99E-05 | 11968.28306 | 9710.197882 | 12001.09445 | 5138.82074 | 7554.053156 | 3330.433594 | Cyr61 |
| -2.120415032 | 1.26E-05 | 8.05E-05 | 49.6570379 | 65.64024481 | 41.00601749 | 9.11300007 | 6.806986399 | 21.81209067 | Slc22a8 |
| -1.044407057 | 1.28E-05 | 8.17E-05 | 407.5624809 | 577.0871523 | 393.2371934 | 226.9137018 | 279.9373157 | 158.1376574 | Zic5 |
| -1.42751615 | 1.31E-05 | 8.30E-05 | 106.8094777 | 175.9523229 | 107.2465073 | 61.96840048 | 33.1840587 | 50.44045968 | Ckap2l |
| -1.404239833 | 1.34E-05 | 8.47E-05 | 67.45861752 | 88.43199649 | 66.24048979 | 22.78250018 | 31.4823121 | 29.99162467 | Deup1 |
| -1.687103737 | 1.36E-05 | 8.56E-05 | 80.57557093 | 78.40362575 | 73.60054421 | 27.33900021 | 33.1840587 | 9.542789669 | Mc4r |
| -1.403748984 | 1.39E-05 | 8.77E-05 | 63.71091655 | 85.69698628 | 80.96059863 | 35.54070027 | 22.9735791 | 28.62836901 | Klf2 |
| -1.279203892 | 1.51E-05 | 9.39E-05 | 384.1393498 | 474.0684348 | 375.3627755 | 216.8894017 | 203.3587187 | 84.52185135 | Plp2 |
| -1.139005013 | 1.54E-05 | 9.61E-05 | 430.0486867 | 386.5481083 | 507.8437551 | 292.5273023 | 165.9202935 | 140.4153337 | Ccnjl |
| -1.926337988 | 1.55E-05 | 9.65E-05 | 3001.90848 | 4881.081538 | 3059.679766 | 930.4373072 | 1403.090072 | 543.9390111 | Steap4 |
| -1.365550281 | 1.56E-05 | 9.66E-05 | 224.8620584 | 275.3243602 | 169.2812517 | 71.08140055 | 129.3327416 | 57.25673801 | Gm13293 |
| -1.809785663 | 1.59E-05 | 9.86E-05 | 61.83706606 | 93.90201689 | 55.72612633 | 18.22600014 | 29.7805655 | 10.90604534 | Prkcq |
| -1.530755028 | 1.61E-05 | 9.94E-05 | 71.2063185 | 80.22696588 | 55.72612633 | 19.13730015 | 31.4823121 | 20.44883501 | Ces1d |
| -1.22510404 | 1.65E-05 | 0.00010178 | 99.3140758 | 104.8420577 | 86.21778036 | 49.21020038 | 43.39453829 | 29.99162467 | Haus4 |
| -1.084476155 | 1.79E-05 | 0.00010923 | 536.8581644 | 939.0201689 | 527.8210456 | 339.9149026 | 361.6211525 | 241.2962531 | Fcrls |
| -1.005516424 | 1.80E-05 | 0.00010975 | 157.4034409 | 240.6808977 | 177.6927425 | 91.1300007 | 106.3591625 | 88.61161836 | Tmtc4 |
| -2.018513097 | 1.80E-05 | 0.00010991 | 39.35086022 | 57.43521421 | 50.4689446 | 15.49210012 | 15.3157194 | 4.089767001 | Ace2 |
| -1.008831766 | 1.83E-05 | 0.00011113 | 225.7989837 | 313.614503 | 233.4188688 | 143.0741011 | 145.4993343 | 92.70138536 | Fblim1 |
| -1.828278566 | 1.86E-05 | 0.00011321 | 46.84626217 | 48.31851354 | 48.36607191 | 20.04860015 | 10.2104796 | 9.542789669 | Mobp |
| -1.121119598 | 1.88E-05 | 0.00011394 | 209.8712545 | 274.4126901 | 165.0755063 | 117.5577009 | 105.5082892 | 73.61580602 | Ly86 |
| -1.656185427 | 2.00E-05 | 0.00012083 | 77.7647952 | 74.75694548 | 48.36607191 | 24.60510019 | 25.526199 | 12.269301 | Gm13301 |
| -1.911434644 | 2.02E-05 | 0.00012157 | 44.97241168 | 32.82012241 | 48.36607191 | 13.66950011 | 11.0613529 | 8.179534002 | Ptx4 |
| -1.180021047 | 2.10E-05 | 0.0001258 | 109.6202535 | 135.83884 | 113.5551254 | 63.79100049 | 57.00851109 | 35.44464734 | Lpxn |
| -1.162386846 | 2.16E-05 | 0.00012917 | 309.1853303 | 596.2322237 | 340.6653761 | 231.4702018 | 167.6220401 | 156.7744017 | Tlr13 |
| -1.516165165 | 2.18E-05 | 0.00013025 | 58.08936509 | 67.46358495 | 57.82899902 | 15.49210012 | 21.2718325 | 28.62836901 | Crygn |
| -1.41518266 | 2.27E-05 | 0.00013518 | 89.94482336 | 113.9587584 | 73.60054421 | 33.71810026 | 24.6753257 | 47.71394835 | Casq1 |
| -1.036799312 | 2.37E-05 | 0.00014027 | 382.2654993 | 581.6455027 | 417.4202293 | 213.2442016 | 293.5512885 | 163.59068 | Pcgf5 |
| -1.227382602 | 2.49E-05 | 0.000147 | 127.4218331 | 177.775663 | 111.4522527 | 45.56500035 | 74.87685039 | 57.25673801 | Mak |
| -1.007206362 | 2.56E-05 | 0.00015096 | 199.5650768 | 213.3307956 | 169.2812517 | 81.10570063 | 125.0783751 | 81.79534002 | 5930430L01Rik |
| -1.126651635 | 2.57E-05 | 0.00015133 | 449.7241168 | 791.329618 | 492.0722099 | 353.5844027 | 199.9552255 | 239.9329974 | Top2a |
| -1.019472773 | 2.60E-05 | 0.00015297 | 182.7004225 | 219.7124861 | 176.6413061 | 79.28310061 | 124.2275018 | 80.43208435 | Hrasls |
| -2.15235886 | 2.68E-05 | 0.00015694 | 30.91853303 | 51.96519381 | 29.44021768 | 6.379100049 | 8.508732999 | 10.90604534 | Cdh3 |
| -2.286121924 | 2.69E-05 | 0.00015727 | 30.91853303 | 27.35010201 | 32.59452672 | 5.467800042 | 8.508732999 | 4.089767001 | Ccna1 |
| -1.036845126 | 3.07E-05 | 0.00017791 | 114.3048797 | 144.0438706 | 111.4522527 | 59.23450046 | 68.06986399 | 51.80371535 | F11r |
| -1.121430844 | 3.33E-05 | 0.00019104 | 89.00789812 | 129.4571495 | 127.2237979 | 57.41190044 | 55.30676449 | 44.98743701 | Fam60a |
| -1.255224929 | 3.42E-05 | 0.00019598 | 83.38634666 | 85.69698628 | 68.34336248 | 30.07290023 | 39.99104509 | 28.62836901 | Fam46b |
| -1.666327206 | 3.53E-05 | 0.00020147 | 65.58476704 | 122.163789 | 67.29192614 | 20.04860015 | 21.2718325 | 40.89767001 | Hist1h4j |
| -2.121396133 | 3.59E-05 | 0.00020445 | 29.98160779 | 47.40684348 | 35.74883576 | 12.7582001 | 5.105239799 | 8.179534002 | Fgfbp1 |
| -1.236914623 | 3.84E-05 | 0.00021723 | 200.5020021 | 228.8291868 | 191.3614149 | 100.2430008 | 114.8678955 | 44.98743701 | Fkbp7 |
| -1.719807522 | 4.00E-05 | 0.00022506 | 56.2155146 | 110.3120781 | 49.41750826 | 20.95990016 | 26.3770723 | 17.72232367 | Gm27544 |
| -1.980002568 | 4.06E-05 | 0.0002279 | 34.666234 | 86.60865635 | 54.67468999 | 9.11300007 | 20.4209592 | 14.99581234 | Crh |
| -1.198416791 | 4.22E-05 | 0.00023664 | 896.6374579 | 1127.735873 | 890.566585 | 671.6281052 | 334.3932069 | 261.7450881 | Ifih1 |
| -1.523166135 | 4.35E-05 | 0.00024275 | 65.58476704 | 51.05352374 | 62.03474441 | 23.69380018 | 24.6753257 | 12.269301 | Nrtn |
| -1.104233084 | 4.55E-05 | 0.00025258 | 207.0604788 | 252.5326085 | 185.0527969 | 92.04130071 | 136.9906013 | 68.16278335 | Mx2 |
| -1.272081767 | 4.55E-05 | 0.00025277 | 224.8620584 | 264.3843194 | 217.6473236 | 82.01700063 | 150.6045741 | 57.25673801 | Clcf1 |
| -1.214822721 | 4.57E-05 | 0.0002534 | 74.01709423 | 80.22696588 | 79.90916229 | 26.4277002 | 39.14017179 | 35.44464734 | Gm12592 |
| -1.116399635 | 5.07E-05 | 0.00027759 | 353.2208167 | 240.6808977 | 390.0828843 | 178.6148014 | 177.8325197 | 94.06464102 | Ntsr1 |
| -1.832214107 | 5.21E-05 | 0.00028503 | 60.90014082 | 60.17022441 | 37.85170845 | 22.78250018 | 12.7630995 | 8.179534002 | Gm17359 |
| -1.102556959 | 5.23E-05 | 0.00028629 | 142.412637 | 179.5990032 | 137.7381613 | 53.76670042 | 95.29780959 | 64.07301635 | Itgb4 |
| -1.180898277 | 5.29E-05 | 0.00028876 | 249.2221147 | 341.8762751 | 253.3961594 | 132.138501 | 167.6220401 | 69.52603902 | Gm867 |
| -1.108926342 | 5.31E-05 | 0.00028975 | 148.9711137 | 216.0658058 | 119.8637434 | 81.10570063 | 65.51724409 | 79.06882869 | Adamts12 |
| -1.092512535 | 5.52E-05 | 0.00030008 | 286.6991245 | 483.1851354 | 298.6079222 | 218.7120017 | 156.5606872 | 124.0562657 | Cd180 |
| -1.376999206 | 5.85E-05 | 0.0003165 | 62.77399131 | 68.37525501 | 64.1376171 | 31.89550025 | 25.526199 | 16.359068 | C78197 |
| -1.524615719 | 6.61E-05 | 0.00035323 | 104.9356273 | 154.9839114 | 113.5551254 | 70.17010054 | 23.8244524 | 35.44464734 | 4930430E12Rik |
| -1.351416683 | 6.71E-05 | 0.00035821 | 90.88174861 | 96.63702709 | 64.1376171 | 38.2746003 | 38.28929849 | 20.44883501 | Gm45650 |
| -1.252380359 | 6.85E-05 | 0.00036438 | 87.13404763 | 113.9587584 | 74.65198056 | 41.91980032 | 45.09628489 | 27.26511334 | Wfdc17 |
| -1.001180727 | 6.90E-05 | 0.00036645 | 721.4324374 | 1287.278134 | 769.6514052 | 450.1822035 | 581.1464638 | 354.4464734 | Trim30a |
| -1.016328983 | 7.64E-05 | 0.00040193 | 445.9764158 | 396.5764791 | 495.2265189 | 245.1397019 | 282.4899356 | 129.5092884 | Adra2c |
| -1.231608056 | 7.85E-05 | 0.00041164 | 115.2418049 | 79.31529582 | 73.60054421 | 43.74240034 | 38.28929849 | 31.35488034 | Gm17322 |
| -1.412141706 | 8.17E-05 | 0.00042728 | 61.83706606 | 72.02193528 | 47.31463556 | 20.04860015 | 27.2279456 | 20.44883501 | Mb |
| -1.723318373 | 8.48E-05 | 0.0004418 | 47.78318741 | 44.67183328 | 41.00601749 | 18.22600014 | 7.657859699 | 14.99581234 | Tnfsf18 |
| -1.085269903 | 8.61E-05 | 0.00044797 | 113.3679544 | 102.1070475 | 116.7094344 | 51.03280039 | 66.36811739 | 36.80790301 | 1700001L05Rik |
| -1.133333796 | 8.80E-05 | 0.00045621 | 208.9343293 | 293.5577615 | 214.4930146 | 158.5662012 | 90.19256979 | 76.34231735 | Glipr1 |
| -1.410007731 | 8.89E-05 | 0.00046014 | 45.90933693 | 51.96519381 | 64.1376171 | 19.13730015 | 21.2718325 | 20.44883501 | D7Ertd443e |
| -1.048912853 | 8.90E-05 | 0.00046034 | 125.5479826 | 150.425561 | 108.2979436 | 75.63790058 | 62.11375089 | 46.35069268 | Ggt5 |
| -1.349846409 | 9.08E-05 | 0.00046886 | 60.90014082 | 109.400408 | 74.65198056 | 34.62940027 | 25.526199 | 36.80790301 | Pimreg |
| -2.201126584 | 9.28E-05 | 0.00047701 | 19.67543011 | 37.37847274 | 30.49165403 | 6.379100049 | 5.956113099 | 6.816278335 | Edar |
| -1.078065719 | 9.41E-05 | 0.00048298 | 292.3206759 | 588.0271931 | 320.6880855 | 191.3730015 | 219.5253114 | 156.7744017 | Ccdc170 |
| -1.13486435 | 9.45E-05 | 0.0004849 | 133.9803098 | 200.5674147 | 165.0755063 | 105.7108008 | 57.00851109 | 64.07301635 | Ccnb1 |
| -1.030187175 | 9.78E-05 | 0.00049946 | 155.5295904 | 118.5171087 | 104.0921982 | 71.08140055 | 61.26287759 | 51.80371535 | Lrig3 |
| -1.013422049 | 0.00010214 | 0.00052027 | 535.9212392 | 877.9382744 | 537.2839727 | 414.6415032 | 325.8844739 | 223.5739294 | 10-Sep |
| -1.526988831 | 0.00010777 | 0.00054713 | 75.89094471 | 182.3340134 | 138.7895977 | 68.34750053 | 38.28929849 | 29.99162467 | Pbk |
| -1.130470658 | 0.00010975 | 0.00055542 | 88.07097288 | 76.58028562 | 69.39479883 | 40.09720031 | 34.8858053 | 31.35488034 | Spc24 |
| -1.810646581 | 0.00011054 | 0.00055879 | 31.85545827 | 37.37847274 | 31.54309038 | 10.93560008 | 9.359606299 | 8.179534002 | Tmem182 |
| -1.485406433 | 0.00011233 | 0.00056698 | 82.44942142 | 136.75051 | 91.47496209 | 58.32320045 | 24.6753257 | 27.26511334 | Arap3 |
| -1.432624958 | 0.00011725 | 0.00058977 | 55.27858936 | 90.25533662 | 62.03474441 | 33.71810026 | 18.7192126 | 24.53860201 | Cntnap3 |
| -1.387281964 | 0.00011908 | 0.00059776 | 66.52169228 | 66.55191488 | 85.16634402 | 22.78250018 | 39.99104509 | 19.08557934 | 2310040G07Rik |
| -1.087957131 | 0.00012717 | 0.00063364 | 74.95401947 | 101.1953774 | 79.90916229 | 40.09720031 | 37.43842519 | 43.62418134 | Prr11 |
| -1.404840537 | 0.00012861 | 0.00064012 | 47.78318741 | 54.70020401 | 51.52038095 | 23.69380018 | 18.7192126 | 14.99581234 | Gm5424 |
| -2.054481066 | 0.00013043 | 0.00064765 | 32.79238352 | 50.14185368 | 23.13159961 | 6.379100049 | 7.657859699 | 12.269301 | Kcne2 |
| -1.029709963 | 0.00013208 | 0.00065483 | 285.7621992 | 400.2231594 | 303.865104 | 173.1470013 | 210.1657051 | 98.15440802 | Mapk15 |
| -1.028218282 | 0.00013252 | 0.00065649 | 103.998702 | 144.9555406 | 136.686725 | 77.4605006 | 62.11375089 | 47.71394835 | Plk1 |
| -1.025412029 | 0.00013392 | 0.00066292 | 142.412637 | 217.889146 | 171.3841244 | 69.25880053 | 113.1661489 | 77.70557302 | Spag8 |
| -1.267036083 | 0.00013726 | 0.0006772 | 137.7280108 | 223.3591664 | 168.2298153 | 99.33170077 | 79.98209019 | 38.17115868 | Tdo2 |
| -1.837725789 | 0.00013866 | 0.00068306 | 29.98160779 | 47.40684348 | 31.54309038 | 8.201700063 | 9.359606299 | 13.63255667 | Gm19412 |
| -2.079211523 | 0.00013882 | 0.00068349 | 42.16163595 | 31.90845234 | 21.02872692 | 7.290400056 | 9.359606299 | 5.453022668 | 4933413G19Rik |
| -1.032344512 | 0.0001427 | 0.00070044 | 178.0157962 | 168.6589624 | 176.6413061 | 76.54920059 | 119.122262 | 57.25673801 | 2900052L18Rik |
| -1.454917651 | 0.00014356 | 0.00070413 | 150.8449642 | 203.3024249 | 193.4642876 | 72.90400056 | 99.55217609 | 24.53860201 | Cfap99 |
| -1.06893997 | 0.00015252 | 0.00074282 | 85.26019715 | 133.1038298 | 99.88645286 | 51.03280039 | 56.15763779 | 43.62418134 | 4921536K21Rik |
| -1.450296418 | 0.0001573 | 0.00076379 | 63.71091655 | 75.66861555 | 67.29192614 | 38.2746003 | 15.3157194 | 21.81209067 | Kif18b |
| -1.058549557 | 0.00015827 | 0.00076787 | 101.1879263 | 144.9555406 | 97.78358017 | 45.56500035 | 63.81549749 | 55.89348235 | Flvcr2 |
| -1.175862149 | 0.00015844 | 0.0007685 | 92.75559909 | 72.93360535 | 66.24048979 | 41.00850032 | 32.3331854 | 28.62836901 | Tfap4 |
| -1.299029765 | 0.00015858 | 0.00076901 | 69.33246801 | 69.28692508 | 65.18905344 | 22.78250018 | 38.28929849 | 20.44883501 | 1600029O15Rik |
| -1.618129423 | 0.00016183 | 0.00078319 | 36.54008449 | 34.64346254 | 52.57181729 | 12.7582001 | 12.7630995 | 14.99581234 | Tmem154 |
| -1.11228301 | 0.00017051 | 0.00081966 | 123.6741321 | 116.6937686 | 76.75485325 | 58.32320045 | 46.79803149 | 40.89767001 | Pik3cg |
| -1.265786483 | 0.00017335 | 0.00083142 | 51.53088839 | 64.72857475 | 62.03474441 | 22.78250018 | 28.9296922 | 21.81209067 | Nhlrc4 |
| -1.207991543 | 0.00017984 | 0.0008581 | 79.63864569 | 94.81368695 | 101.9893255 | 31.89550025 | 56.15763779 | 29.99162467 | Lrrc46 |
| -1.302653597 | 0.0001817 | 0.00086632 | 57.15243985 | 52.87686388 | 50.4689446 | 22.78250018 | 23.8244524 | 17.72232367 | Cpz |
| -1.950303798 | 0.00018203 | 0.00086747 | 33.72930876 | 22.79175167 | 35.74883576 | 10.02430008 | 6.806986399 | 6.816278335 | Sp5 |
| -1.229332269 | 0.00018615 | 0.00088491 | 252.0328905 | 434.8666219 | 239.7274869 | 199.5747015 | 100.4030494 | 94.06464102 | Rasgrp3 |
| -1.184060881 | 0.00019311 | 0.00091414 | 74.01709423 | 94.81368695 | 99.88645286 | 47.38760037 | 44.24541159 | 24.53860201 | Adamts14 |
| -1.102967308 | 0.00020827 | 0.00097894 | 87.13404763 | 132.1921597 | 98.83501651 | 61.96840048 | 46.79803149 | 38.17115868 | Cenpa |
| -1.551237322 | 0.00020864 | 0.00098045 | 39.35086022 | 59.25855435 | 46.26319922 | 19.13730015 | 19.5700859 | 9.542789669 | Gna15 |
| -1.067377257 | 0.00021474 | 0.0010064 | 78.70172044 | 80.22696588 | 66.24048979 | 33.71810026 | 38.28929849 | 35.44464734 | Nt5c1a |
| -1.065513482 | 0.00022149 | 0.00103476 | 75.89094471 | 74.75694548 | 66.24048979 | 35.54070027 | 34.034932 | 34.08139168 | Rin3 |
| -1.173312196 | 0.00023026 | 0.00107136 | 115.2418049 | 182.3340134 | 112.503689 | 51.9441004 | 83.38558339 | 44.98743701 | Daw1 |
| -1.425028279 | 0.0002511 | 0.00115381 | 46.84626217 | 62.90523461 | 51.52038095 | 23.69380018 | 12.7630995 | 24.53860201 | Dlx2 |
| -1.284662228 | 0.00025242 | 0.00115878 | 341.9777138 | 903.4650363 | 316.4823401 | 251.5188019 | 190.5956192 | 199.0353274 | Cfh |
| -1.043477912 | 0.00026731 | 0.00121902 | 115.2418049 | 122.163789 | 121.9666161 | 81.10570063 | 49.35065139 | 42.26092568 | Slc25a13 |
| -1.179095707 | 0.00027623 | 0.00125545 | 67.45861752 | 55.61187408 | 55.72612633 | 25.5164002 | 26.3770723 | 27.26511334 | Baiap3 |
| -1.07784785 | 0.00028432 | 0.00128714 | 80.57557093 | 105.7537278 | 85.16634402 | 42.83110033 | 52.75414459 | 31.35488034 | Ehhadh |
| -1.146957934 | 0.00029852 | 0.00134043 | 69.33246801 | 56.52354415 | 82.01203498 | 34.62940027 | 28.9296922 | 29.99162467 | Myzap |
| -1.154668315 | 0.00032077 | 0.00143168 | 98.37715055 | 120.3404488 | 114.6065617 | 43.74240034 | 72.32423049 | 31.35488034 | Oas1b |
| -1.276118155 | 0.00032367 | 0.00144225 | 94.62944958 | 105.7537278 | 108.2979436 | 36.45200028 | 65.51724409 | 23.17534634 | Stpg1 |
| -1.805875966 | 0.00032952 | 0.00146433 | 32.79238352 | 65.64024481 | 46.26319922 | 9.11300007 | 9.359606299 | 24.53860201 | Wdr72 |
| -1.0529209 | 0.00033201 | 0.0014744 | 530.2996877 | 1042.038886 | 472.0949193 | 414.6415032 | 308.0161346 | 261.7450881 | Cd84 |
| -1.184792309 | 0.00034943 | 0.00154525 | 103.0617768 | 67.46358495 | 83.06347132 | 32.80680025 | 28.9296922 | 51.80371535 | Ryr1 |
| -1.390336081 | 0.00035249 | 0.001557 | 42.16163595 | 41.02515301 | 46.26319922 | 16.40340013 | 18.7192126 | 13.63255667 | Gm42693 |
| -1.01395446 | 0.00035751 | 0.00157737 | 171.4573195 | 231.564197 | 217.6473236 | 137.6063011 | 105.5082892 | 61.34650502 | Tox3 |
| -1.380269734 | 0.00036432 | 0.00160448 | 42.16163595 | 49.23018361 | 50.4689446 | 23.69380018 | 15.3157194 | 14.99581234 | Vegfd |
| -1.141462694 | 0.00036638 | 0.00161172 | 148.9711137 | 182.3340134 | 133.5324159 | 105.7108008 | 59.56113099 | 43.62418134 | Wdfy4 |
| -1.068267119 | 0.00040725 | 0.0017701 | 121.8002816 | 157.7189216 | 121.9666161 | 65.61360051 | 85.08732999 | 38.17115868 | 9930012K11Rik |
| -1.125451269 | 0.00041068 | 0.00178304 | 103.998702 | 72.02193528 | 66.24048979 | 30.98420024 | 37.43842519 | 43.62418134 | Fam83d |
| -1.627223001 | 0.00041738 | 0.00180804 | 38.41393498 | 55.61187408 | 52.57181729 | 23.69380018 | 7.657859699 | 16.359068 | Mal |
| -1.840332084 | 0.00042986 | 0.00185384 | 25.29698157 | 33.73179247 | 35.74883576 | 4.556500035 | 10.2104796 | 12.269301 | Scube2 |
| -1.029841247 | 0.00051234 | 0.00216204 | 83.38634666 | 123.075459 | 77.80628959 | 44.65370034 | 51.90327129 | 42.26092568 | Morn3 |
| -1.258425459 | 0.00052805 | 0.00221961 | 62.77399131 | 123.075459 | 71.49767152 | 33.71810026 | 28.9296922 | 46.35069268 | Ppp1r3b |
| -1.23282378 | 0.00053239 | 0.00223637 | 83.38634666 | 119.4287788 | 60.98330806 | 39.1859003 | 45.94715819 | 25.90185767 | Tnfrsf11b |
| -1.741796889 | 0.00053944 | 0.00226204 | 30.91853303 | 44.67183328 | 23.13159961 | 10.02430008 | 11.0613529 | 8.179534002 | Tmem221 |
| -1.952994806 | 0.0005507 | 0.00230125 | 21.5492806 | 33.73179247 | 24.18303596 | 9.11300007 | 6.806986399 | 4.089767001 | Glt28d2 |
| -1.147769822 | 0.00055235 | 0.00230714 | 84.3232719 | 155.8955814 | 80.96059863 | 41.91980032 | 49.35065139 | 54.53022668 | 4833427G06Rik |
| -1.719151336 | 0.00055488 | 0.00231581 | 79.63864569 | 222.4474963 | 64.1376171 | 45.56500035 | 46.79803149 | 17.72232367 | Cxcl5 |
| -1.30738809 | 0.0005549 | 0.00231581 | 48.72011266 | 49.23018361 | 53.62325364 | 23.69380018 | 23.8244524 | 12.269301 | A630014C17Rik |
| -1.792568049 | 0.00056641 | 0.00235671 | 24.36005633 | 26.43843194 | 28.38878134 | 8.201700063 | 7.657859699 | 6.816278335 | 2810430I11Rik |
| -1.714589965 | 0.00057099 | 0.00237424 | 27.17083206 | 41.02515301 | 28.38878134 | 12.7582001 | 9.359606299 | 6.816278335 | Gm12144 |
| -1.436700631 | 0.00057369 | 0.00238441 | 49.6570379 | 103.9303876 | 46.26319922 | 20.95990016 | 27.2279456 | 25.90185767 | Sap30bpos |
| -1.557065467 | 0.00057857 | 0.00240211 | 31.85545827 | 66.55191488 | 38.9031448 | 15.49210012 | 12.7630995 | 19.08557934 | Pantr2 |
| -1.183070298 | 0.00059217 | 0.00245225 | 63.71091655 | 103.9303876 | 75.7034169 | 46.47630036 | 34.8858053 | 24.53860201 | Steap1 |
| -1.246294102 | 0.00059307 | 0.00245492 | 65.58476704 | 43.76016321 | 53.62325364 | 19.13730015 | 22.9735791 | 27.26511334 | Slc15a1 |
| -1.11704668 | 0.00059785 | 0.00247044 | 90.88174861 | 87.52032642 | 104.0921982 | 61.96840048 | 39.14017179 | 27.26511334 | B3gnt8 |
| -1.173030488 | 0.00061442 | 0.00253294 | 272.6452458 | 443.0716525 | 349.0768668 | 222.3572017 | 176.1307731 | 70.88929468 | Anln |
| -1.134302823 | 0.00068715 | 0.00280164 | 131.1695341 | 211.5074555 | 129.3266705 | 100.2430008 | 70.62248389 | 42.26092568 | Bcl2a1b |
| -1.014046379 | 0.00070129 | 0.00285387 | 85.26019715 | 124.8987992 | 77.80628959 | 40.09720031 | 50.20152469 | 53.16697101 | Gm5089 |
| -1.994219778 | 0.0007844 | 0.00315282 | 21.5492806 | 24.61509181 | 29.44021768 | 10.02430008 | 3.4034932 | 5.453022668 | Cited4 |
| -1.144153764 | 0.00080494 | 0.00322727 | 126.4849079 | 143.1322005 | 132.4809796 | 69.25880053 | 82.53471009 | 27.26511334 | Gm1661 |
| -1.096570114 | 0.00082685 | 0.00330345 | 82.44942142 | 100.2837074 | 78.85772594 | 56.50060044 | 38.28929849 | 25.90185767 | Ifi27l2a |
| -1.543702295 | 0.00083541 | 0.00333279 | 27.17083206 | 34.64346254 | 42.05745383 | 11.84690009 | 9.359606299 | 14.99581234 | A430027H14Rik |
| -1.347610481 | 0.00089267 | 0.00353637 | 64.64784179 | 42.84849314 | 67.29192614 | 19.13730015 | 33.1840587 | 14.99581234 | Pth1r |
| -1.123625956 | 0.00094508 | 0.00371593 | 78.70172044 | 145.8672107 | 79.90916229 | 54.67800042 | 36.58755189 | 49.07720401 | Hmmr |
| -2.451557025 | 0.00102887 | 0.00400599 | 18.73850487 | 12.76338094 | 23.13159961 | 5.467800042 | 1.7017466 | 2.726511334 | Gm13594 |
| -1.049680445 | 0.0010694 | 0.00414456 | 132.1064593 | 181.4223433 | 151.4068338 | 65.61360051 | 111.4644023 | 44.98743701 | Gm10561 |
| -1.001745915 | 0.00108273 | 0.00418778 | 213.6189555 | 213.3307956 | 220.8016326 | 109.3560008 | 154.8589406 | 55.89348235 | Fam26e |
| -1.107782622 | 0.0011013 | 0.00424512 | 46.84626217 | 72.02193528 | 66.24048979 | 29.16160023 | 31.4823121 | 24.53860201 | Lhb |
| -1.147390937 | 0.00111329 | 0.00428193 | 53.40473887 | 62.90523461 | 54.67468999 | 19.13730015 | 33.1840587 | 24.53860201 | Olfml2a |
| -1.091247189 | 0.00111935 | 0.00429753 | 142.412637 | 169.5706324 | 142.995343 | 63.79100049 | 108.9117824 | 38.17115868 | Ankrd66 |
| -1.408325928 | 0.00112389 | 0.00431152 | 44.97241168 | 30.08511221 | 39.95458114 | 17.31470013 | 14.4648461 | 10.90604534 | Gm37711 |
| -1.026415022 | 0.00114656 | 0.00438801 | 106.8094777 | 160.4539318 | 99.88645286 | 73.81530057 | 66.36811739 | 38.17115868 | Hvcn1 |
| -1.306673154 | 0.00120202 | 0.00457395 | 47.78318741 | 44.67183328 | 53.62325364 | 17.31470013 | 28.0788189 | 12.269301 | Sncg |
| -1.275886572 | 0.00125998 | 0.00477 | 40.28778546 | 45.58350334 | 34.69739941 | 17.31470013 | 16.1665927 | 16.359068 | C1qtnf1 |
| -1.072831054 | 0.00129388 | 0.00487347 | 53.40473887 | 59.25855435 | 58.88043537 | 31.89550025 | 28.0788189 | 20.44883501 | Ada |
| -1.116650539 | 0.00138343 | 0.00516935 | 62.77399131 | 61.99356455 | 54.67468999 | 35.54070027 | 28.0788189 | 17.72232367 | A330048O09Rik |
| -1.345015889 | 0.00138596 | 0.00517781 | 33.72930876 | 49.23018361 | 55.72612633 | 13.66950011 | 16.1665927 | 25.90185767 | Ticrr |
| -1.150827994 | 0.00139208 | 0.00519865 | 73.08016898 | 128.5454794 | 89.3720894 | 60.14580046 | 43.39453829 | 25.90185767 | Dydc2 |
| -1.055408388 | 0.00140894 | 0.00525046 | 49.6570379 | 72.02193528 | 57.82899902 | 28.25030022 | 30.6314388 | 27.26511334 | Tpbgl |
| -1.125132358 | 0.00142438 | 0.00529874 | 55.27858936 | 48.31851354 | 60.98330806 | 26.4277002 | 18.7192126 | 31.35488034 | 5930412G12Rik |
| -1.556649801 | 0.00144262 | 0.00535627 | 30.91853303 | 34.64346254 | 28.38878134 | 12.7582001 | 12.7630995 | 5.453022668 | Pax6os1 |
| -1.013645493 | 0.00145858 | 0.00540854 | 78.70172044 | 104.8420577 | 66.24048979 | 30.98420024 | 45.94715819 | 47.71394835 | Bcas1 |
| -1.056022738 | 0.00146237 | 0.00542021 | 172.3942448 | 162.2772719 | 205.0300874 | 138.5176011 | 64.66637079 | 54.53022668 | Cd83 |
| -1.379649259 | 0.00147962 | 0.00547572 | 67.45861752 | 97.54869715 | 33.64596307 | 29.16160023 | 26.3770723 | 20.44883501 | Ctla2b |
| -1.121095344 | 0.0015307 | 0.0056388 | 324.1761342 | 248.8859283 | 273.3734499 | 151.2758012 | 182.9377595 | 51.80371535 | Scarf2 |
| -1.251199828 | 0.00154568 | 0.00568853 | 39.35086022 | 38.29014281 | 57.82899902 | 21.87120017 | 17.017466 | 17.72232367 | Gm6556 |
| -1.455687537 | 0.0015614 | 0.00573821 | 30.91853303 | 33.73179247 | 33.64596307 | 8.201700063 | 15.3157194 | 12.269301 | 2310016G11Rik |
| -2.013718438 | 0.00159042 | 0.00583221 | 239.8528623 | 712.9259923 | 235.5217415 | 133.961101 | 103.8065426 | 55.89348235 | Ogn |
| -1.167649441 | 0.00163723 | 0.00598436 | 50.59396314 | 61.99356455 | 52.57181729 | 31.89550025 | 16.1665927 | 25.90185767 | Chaf1b |
| -1.773359168 | 0.00164202 | 0.00599844 | 18.73850487 | 22.79175167 | 29.44021768 | 7.290400056 | 7.657859699 | 5.453022668 | Plk5 |
| -1.020907577 | 0.00165663 | 0.0060461 | 65.58476704 | 119.4287788 | 78.85772594 | 36.45200028 | 47.64890479 | 46.35069268 | Greb1 |
| -1.132583021 | 0.0016586 | 0.00605086 | 44.97241168 | 43.76016321 | 56.77756268 | 22.78250018 | 23.8244524 | 19.08557934 | Ankdd1a |
| -1.047529382 | 0.00166664 | 0.00607342 | 136.7910855 | 202.3907548 | 105.1436346 | 64.7023005 | 99.55217609 | 49.07720401 | Ttll6 |
| -2.004199641 | 0.00172673 | 0.00625341 | 28.1077573 | 41.02515301 | 19.97729057 | 2.733900021 | 13.6139728 | 5.453022668 | Tnfsf13 |
| -1.077999983 | 0.00176618 | 0.00637234 | 49.6570379 | 63.81690468 | 59.93187171 | 26.4277002 | 34.034932 | 20.44883501 | Gbp10 |
| -1.031158222 | 0.00178186 | 0.00641973 | 75.89094471 | 99.37203729 | 65.18905344 | 30.98420024 | 51.90327129 | 34.08139168 | Efcab11 |
| -1.182594737 | 0.00179067 | 0.00644741 | 55.27858936 | 64.72857475 | 41.00601749 | 24.60510019 | 28.9296922 | 16.359068 | Gm32389 |
| -1.068765023 | 0.00183679 | 0.00658886 | 51.53088839 | 61.99356455 | 49.41750826 | 30.98420024 | 22.1227058 | 24.53860201 | Gm6356 |
| -1.221874492 | 0.00184917 | 0.00662957 | 47.78318741 | 51.96519381 | 39.95458114 | 26.4277002 | 14.4648461 | 19.08557934 | Mir155hg |
| -1.506706804 | 0.00193863 | 0.00688628 | 38.41393498 | 90.25533662 | 36.80027211 | 15.49210012 | 27.2279456 | 14.99581234 | A330033J07Rik |
| -1.065371908 | 0.00194188 | 0.0068956 | 103.998702 | 234.2992072 | 115.657998 | 86.57350067 | 72.32423049 | 57.25673801 | Ifi44 |
| -1.240023183 | 0.00194838 | 0.00690799 | 48.72011266 | 61.99356455 | 54.67468999 | 35.54070027 | 16.1665927 | 17.72232367 | Cep55 |
| -1.114854825 | 0.00194814 | 0.00690799 | 63.71091655 | 79.31529582 | 53.62325364 | 41.00850032 | 21.2718325 | 28.62836901 | Dlgap5 |
| -1.103576434 | 0.00195654 | 0.00693097 | 52.46781363 | 75.66861555 | 50.4689446 | 32.80680025 | 28.9296922 | 20.44883501 | Fbxl8 |
| -1.025580488 | 0.00201272 | 0.00711145 | 127.4218331 | 143.1322005 | 110.4008163 | 92.04130071 | 36.58755189 | 58.61999368 | Bub1b |
| -1.198583648 | 0.00204835 | 0.00721619 | 38.41393498 | 49.23018361 | 49.41750826 | 18.22600014 | 25.526199 | 14.99581234 | Mfsd7a |
| -1.930271078 | 0.00208657 | 0.00733344 | 23.42313108 | 26.43843194 | 16.82298153 | 2.733900021 | 5.956113099 | 9.542789669 | Otx2os1 |
| -1.684303484 | 0.00212101 | 0.00743818 | 29.04468254 | 38.29014281 | 27.33734499 | 4.556500035 | 8.508732999 | 17.72232367 | 9130017K11Rik |
| -1.903557098 | 0.00221809 | 0.00773362 | 15.92772914 | 32.82012241 | 17.87441788 | 5.467800042 | 6.806986399 | 5.453022668 | BC023105 |
| -2.34676379 | 0.00225234 | 0.00784171 | 12.18002816 | 15.49839114 | 22.08016326 | 5.467800042 | 2.5526199 | 1.363255667 | Vpreb3 |
| -2.046216402 | 0.00238554 | 0.00823262 | 22.48620584 | 28.26177207 | 13.6686725 | 2.733900021 | 8.508732999 | 4.089767001 | Rapsn |
| -1.101609845 | 0.00240066 | 0.00827591 | 63.71091655 | 68.37525501 | 48.36607191 | 21.87120017 | 38.28929849 | 23.17534634 | Il21r |
| -1.391221674 | 0.00242828 | 0.00836065 | 26.23390681 | 57.43521421 | 42.05745383 | 15.49210012 | 19.5700859 | 12.269301 | Rassf7 |
| -1.116780046 | 0.00244292 | 0.00840506 | 275.4560216 | 704.7209617 | 281.7849407 | 239.6719019 | 222.0779313 | 118.603243 | Iigp1 |
| -1.041743004 | 0.00247567 | 0.00851319 | 54.34166412 | 60.17022441 | 49.41750826 | 32.80680025 | 25.526199 | 20.44883501 | Depdc7 |
| -1.421757663 | 0.00249887 | 0.00858378 | 45.90933693 | 34.64346254 | 30.49165403 | 18.22600014 | 15.3157194 | 6.816278335 | Gm13110 |
| -1.187494889 | 0.00258229 | 0.0088357 | 63.71091655 | 51.05352374 | 47.31463556 | 30.07290023 | 27.2279456 | 12.269301 | Chrna2 |
| -1.594202121 | 0.00260031 | 0.00887497 | 26.23390681 | 28.26177207 | 27.33734499 | 12.7582001 | 9.359606299 | 4.089767001 | Omp |
| -2.029672761 | 0.00271707 | 0.00922647 | 20.61235535 | 28.26177207 | 16.82298153 | 4.556500035 | 9.359606299 | 1.363255667 | Gli1 |
| -1.835443806 | 0.00273449 | 0.00927667 | 24.36005633 | 30.99678227 | 22.08016326 | 8.201700063 | 11.0613529 | 1.363255667 | Ces1g |
| -1.330212762 | 0.00274903 | 0.00931836 | 56.2155146 | 51.96519381 | 47.31463556 | 12.7582001 | 14.4648461 | 36.80790301 | Ano2 |
| -1.090910702 | 0.00275822 | 0.00934478 | 50.59396314 | 41.93682308 | 44.16032653 | 23.69380018 | 22.1227058 | 17.72232367 | Rnf125 |
| -1.230307551 | 0.00279532 | 0.00945223 | 30.91853303 | 51.96519381 | 47.31463556 | 14.58080011 | 19.5700859 | 21.81209067 | Eps8l2 |
| -1.219196875 | 0.00291997 | 0.00982888 | 41.22471071 | 76.58028562 | 43.10889018 | 26.4277002 | 25.526199 | 16.359068 | Gm43197 |
| -1.015675286 | 0.00296203 | 0.00995658 | 56.2155146 | 60.17022441 | 44.16032653 | 23.69380018 | 29.7805655 | 25.90185767 | 1700113A16Rik |
| -1.052288482 | 0.00301753 | 0.01011492 | 65.58476704 | 90.25533662 | 45.21176287 | 33.71810026 | 33.1840587 | 29.99162467 | Bcl2a1d |
| -1.116103922 | 0.00311569 | 0.01038976 | 39.35086022 | 41.02515301 | 53.62325364 | 19.13730015 | 18.7192126 | 24.53860201 | Dll3 |
| -1.776836902 | 0.00312905 | 0.01042692 | 20.61235535 | 28.26177207 | 19.97729057 | 10.02430008 | 3.4034932 | 6.816278335 | Gm14165 |
| -1.182116055 | 0.00316698 | 0.01053529 | 72.14324374 | 111.2237482 | 103.0407619 | 36.45200028 | 67.21899069 | 20.44883501 | Trim34b |
| -1.177190443 | 0.00331829 | 0.01095922 | 51.53088839 | 56.52354415 | 37.85170845 | 16.40340013 | 29.7805655 | 17.72232367 | Spaca9 |
| -1.164115268 | 0.00347661 | 0.01140783 | 45.90933693 | 78.40362575 | 43.10889018 | 23.69380018 | 19.5700859 | 32.71813601 | Slc13a4 |
| -1.164870346 | 0.00349412 | 0.0114614 | 34.666234 | 47.40684348 | 60.98330806 | 20.95990016 | 25.526199 | 16.359068 | Slc2a4 |
| -1.042523066 | 0.00351577 | 0.01152597 | 164.8988428 | 92.07867675 | 147.2010884 | 65.61360051 | 92.74518969 | 35.44464734 | Epha8 |
| -1.022471418 | 0.00364261 | 0.0118838 | 51.53088839 | 51.05352374 | 71.49767152 | 21.87120017 | 34.8858053 | 28.62836901 | Tcfl5 |
| -1.027454871 | 0.0036913 | 0.01201416 | 120.8633564 | 117.6054386 | 114.6065617 | 38.2746003 | 93.59606299 | 39.53441434 | Llgl2 |
| -1.928597766 | 0.00373561 | 0.01213236 | 1016.563889 | 1174.231046 | 984.1444197 | 322.6002025 | 433.9453829 | 76.34231735 | Acta2 |
| -2.197600049 | 0.00376159 | 0.01220586 | 14.99080389 | 17.32173127 | 19.97729057 | 6.379100049 | 4.254366499 | 0 | Gm9922 |
| -1.171958464 | 0.00377134 | 0.01223242 | 33.72930876 | 39.20181288 | 35.74883576 | 16.40340013 | 14.4648461 | 17.72232367 | Hist1h1b |
| -1.016836978 | 0.00383059 | 0.01240469 | 51.53088839 | 86.60865635 | 75.7034169 | 40.09720031 | 40.84191839 | 23.17534634 | Ercc6l |
| -1.048129217 | 0.0039331 | 0.01269191 | 57.15243985 | 64.72857475 | 58.88043537 | 41.00850032 | 27.2279456 | 17.72232367 | Gm42984 |
| -1.20599688 | 0.00393489 | 0.01269557 | 45.90933693 | 38.29014281 | 28.38878134 | 16.40340013 | 16.1665927 | 16.359068 | 5830408C22Rik |
| -1.103171871 | 0.00399244 | 0.01285542 | 39.35086022 | 48.31851354 | 42.05745383 | 23.69380018 | 15.3157194 | 21.81209067 | Rec8 |
| -1.188499291 | 0.0040953 | 0.01314929 | 176.1419458 | 440.3366423 | 115.657998 | 131.227201 | 106.3591625 | 83.15859569 | Gm4951 |
| -1.327923732 | 0.00416238 | 0.01331145 | 23.42313108 | 50.14185368 | 38.9031448 | 12.7582001 | 17.017466 | 14.99581234 | Col13a1 |
| -1.856516249 | 0.00416749 | 0.01332559 | 20.61235535 | 30.99678227 | 19.97729057 | 6.379100049 | 11.0613529 | 1.363255667 | Gm43703 |
| -2.313553202 | 0.00420755 | 0.01343263 | 24.36005633 | 36.46680267 | 26.28590865 | 0.911300007 | 1.7017466 | 16.359068 | Tmem27 |
| -1.027395829 | 0.00422173 | 0.01346998 | 52.46781363 | 65.64024481 | 57.82899902 | 18.22600014 | 32.3331854 | 36.80790301 | Nsl1 |
| -1.312842356 | 0.00427878 | 0.01363398 | 32.79238352 | 30.08511221 | 35.74883576 | 8.201700063 | 14.4648461 | 17.72232367 | Xkr5 |
| -1.117470034 | 0.00428957 | 0.01366384 | 166.7726933 | 477.715115 | 160.8697609 | 156.7436012 | 113.1661489 | 100.8809194 | Casp12 |
| -1.09581892 | 0.00430852 | 0.01371061 | 39.35086022 | 50.14185368 | 41.00601749 | 20.04860015 | 16.1665927 | 25.90185767 | E2f7 |
| -1.83276305 | 0.0043364 | 0.01378114 | 17.80157962 | 39.20181288 | 17.87441788 | 5.467800042 | 4.254366499 | 12.269301 | Sult1c2 |
| -1.479690531 | 0.00434138 | 0.01379242 | 21.5492806 | 34.64346254 | 27.33734499 | 6.379100049 | 12.7630995 | 10.90604534 | A930005G22Rik |
| -1.394780094 | 0.00441951 | 0.01401292 | 25.29698157 | 55.61187408 | 52.57181729 | 20.95990016 | 20.4209592 | 8.179534002 | Gchfr |
| -1.576778176 | 0.00445092 | 0.01410092 | 583.7044266 | 1052.978927 | 547.7983362 | 378.1895029 | 239.9462706 | 113.1502204 | Kctd4 |
| -1.395018285 | 0.00470671 | 0.01481634 | 48.72011266 | 73.84527542 | 63.08618075 | 30.98420024 | 32.3331854 | 5.453022668 | Apol9a |
| -1.133384233 | 0.00473384 | 0.01488717 | 58.08936509 | 69.28692508 | 42.05745383 | 37.36330029 | 17.8683393 | 21.81209067 | Sucnr1 |
| -1.079934628 | 0.00477976 | 0.01500708 | 112.4310292 | 78.40362575 | 97.78358017 | 46.47630036 | 67.21899069 | 20.44883501 | Gm42864 |
| -1.016116755 | 0.00478938 | 0.0150324 | 39.35086022 | 57.43521421 | 48.36607191 | 26.4277002 | 22.1227058 | 23.17534634 | Gm26896 |
| -1.175481414 | 0.00481123 | 0.01509608 | 44.03548644 | 74.75694548 | 57.82899902 | 25.5164002 | 37.43842519 | 13.63255667 | Insl6 |
| -1.59660883 | 0.00482923 | 0.01514515 | 20.61235535 | 67.46358495 | 26.28590865 | 11.84690009 | 10.2104796 | 16.359068 | Gm27608 |
| -1.448281308 | 0.00485859 | 0.01522567 | 21.5492806 | 41.93682308 | 24.18303596 | 9.11300007 | 11.0613529 | 12.269301 | Hal |
| -1.010879618 | 0.00509449 | 0.01583792 | 97.44022531 | 85.69698628 | 88.32065305 | 70.17010054 | 27.2279456 | 36.80790301 | Slc9a4 |
| -1.388168895 | 0.0052589 | 0.0162939 | 24.36005633 | 25.52676187 | 25.2344723 | 10.02430008 | 10.2104796 | 8.179534002 | Gdnf |
| -1.252377627 | 0.00529791 | 0.01638316 | 41.22471071 | 51.96519381 | 24.18303596 | 14.58080011 | 16.1665927 | 19.08557934 | Gm27582 |
| -1.110512049 | 0.0053072 | 0.01640663 | 44.97241168 | 56.52354415 | 50.4689446 | 31.89550025 | 24.6753257 | 12.269301 | Rubcnl |
| -1.164685727 | 0.00558082 | 0.01714528 | 33.72930876 | 32.82012241 | 35.74883576 | 12.7582001 | 14.4648461 | 19.08557934 | Acnat1 |
| -1.066794321 | 0.00569905 | 0.01745289 | 56.2155146 | 43.76016321 | 33.64596307 | 20.04860015 | 22.1227058 | 21.81209067 | B130034C11Rik |
| -1.270307418 | 0.00574666 | 0.01756971 | 38.41393498 | 58.34688428 | 35.74883576 | 27.33900021 | 17.017466 | 9.542789669 | Cdca5 |
| -2.227374514 | 0.00582306 | 0.0177481 | 17.80157962 | 36.46680267 | 8.411490767 | 8.201700063 | 3.4034932 | 1.363255667 | Tmem184a |
| -1.089945453 | 0.0058643 | 0.0178512 | 94.62944958 | 102.1070475 | 84.11490767 | 44.65370034 | 67.21899069 | 17.72232367 | Car9 |
| -1.014131962 | 0.00609011 | 0.01846571 | 73.08016898 | 61.99356455 | 46.26319922 | 34.62940027 | 19.5700859 | 36.80790301 | Kctd14 |
| -1.08457808 | 0.00616892 | 0.01867235 | 32.79238352 | 44.67183328 | 39.95458114 | 17.31470013 | 21.2718325 | 16.359068 | Gm10825 |
| -1.085104427 | 0.00619398 | 0.01872077 | 85.26019715 | 100.2837074 | 91.47496209 | 36.45200028 | 71.47335719 | 20.44883501 | Sema3f |
| -1.650147344 | 0.0063612 | 0.01913422 | 18.73850487 | 24.61509181 | 15.77154519 | 8.201700063 | 5.105239799 | 5.453022668 | Gm13031 |
| -1.309100025 | 0.00640514 | 0.01924535 | 29.04468254 | 30.99678227 | 31.54309038 | 17.31470013 | 11.9122262 | 6.816278335 | Spink13 |
| -1.452860636 | 0.00641746 | 0.01927638 | 29.04468254 | 24.61509181 | 23.13159961 | 8.201700063 | 13.6139728 | 5.453022668 | A330070K13Rik |
| -1.109297321 | 0.00649891 | 0.01949977 | 36.54008449 | 58.34688428 | 35.74883576 | 21.87120017 | 16.1665927 | 23.17534634 | Nlrp12 |
| -1.078123654 | 0.00657051 | 0.0196962 | 51.53088839 | 60.17022441 | 34.69739941 | 30.07290023 | 18.7192126 | 20.44883501 | Olr1 |
| -1.334836166 | 0.00665045 | 0.01990006 | 42.16163595 | 24.61509181 | 37.85170845 | 11.84690009 | 20.4209592 | 8.179534002 | Cdh15 |
| -1.124993725 | 0.00713185 | 0.021126 | 10575.07522 | 21924.75344 | 10686.79902 | 8224.482563 | 6042.902176 | 5533.454752 | Spp1 |
| -1.071809856 | 0.00714513 | 0.02113959 | 34.666234 | 50.14185368 | 56.77756268 | 20.95990016 | 28.9296922 | 16.359068 | Gad1os |
| -1.654372734 | 0.00720883 | 0.02129842 | 14.05387865 | 23.70342174 | 24.18303596 | 4.556500035 | 9.359606299 | 5.453022668 | BC051019 |
| -1.785249059 | 0.00755978 | 0.022206 | 23.42313108 | 17.32173127 | 12.61723615 | 5.467800042 | 6.806986399 | 2.726511334 | Serpina3m |
| -1.369359103 | 0.007568 | 0.02222338 | 19.67543011 | 53.78853394 | 32.59452672 | 10.93560008 | 15.3157194 | 14.99581234 | Gm27753 |
| -1.669471403 | 0.00757146 | 0.02223014 | 17.80157962 | 16.4100612 | 27.33734499 | 8.201700063 | 7.657859699 | 2.726511334 | Foxc2 |
| -1.059994238 | 0.00767483 | 0.02249282 | 42.16163595 | 55.61187408 | 37.85170845 | 20.95990016 | 28.0788189 | 14.99581234 | Cd59b |
| -1.186352205 | 0.00776239 | 0.02268363 | 38.41393498 | 54.70020401 | 44.16032653 | 14.58080011 | 13.6139728 | 34.08139168 | Sytl3 |
| -1.049659665 | 0.0078249 | 0.02284899 | 59.96321558 | 45.58350334 | 65.18905344 | 34.62940027 | 32.3331854 | 13.63255667 | Derl3 |
| -1.654926139 | 0.00782668 | 0.02285074 | 13.11695341 | 31.90845234 | 28.38878134 | 11.84690009 | 6.806986399 | 4.089767001 | 1700019G24Rik |
| -1.797062742 | 0.00805188 | 0.02340191 | 13.11695341 | 37.37847274 | 18.92585423 | 6.379100049 | 10.2104796 | 2.726511334 | Prss23os |
| -1.102663318 | 0.00818376 | 0.02373155 | 49.6570379 | 65.64024481 | 43.10889018 | 37.36330029 | 20.4209592 | 14.99581234 | Atp2a3 |
| -1.141570059 | 0.00838483 | 0.02425124 | 37.47700973 | 54.70020401 | 37.85170845 | 27.33900021 | 19.5700859 | 10.90604534 | Parpbp |
| -1.032759875 | 0.00846002 | 0.02442977 | 92.75559909 | 143.1322005 | 108.2979436 | 60.14580046 | 82.53471009 | 23.17534634 | Lrrc74b |
| -1.08380987 | 0.00887623 | 0.02542982 | 96.50330007 | 103.9303876 | 73.60054421 | 74.72660058 | 26.3770723 | 27.26511334 | Clec5a |
| -1.380125425 | 0.00892845 | 0.02556526 | 10067.26174 | 11861.73924 | 10383.98535 | 3254.252325 | 7060.546642 | 2098.050472 | Lcn2 |
| -1.161791755 | 0.00921736 | 0.02627964 | 27.17083206 | 41.93682308 | 44.16032653 | 12.7582001 | 14.4648461 | 24.53860201 | Svopl |
| -1.076990379 | 0.00939648 | 0.02670601 | 79.63864569 | 77.49195568 | 106.1950709 | 34.62940027 | 68.92073729 | 19.08557934 | Tekt4 |
| -1.027372282 | 0.00942086 | 0.02676345 | 37.47700973 | 46.49517341 | 42.05745383 | 27.33900021 | 15.3157194 | 19.08557934 | Rrad |
| -2.224166773 | 0.00945427 | 0.02682674 | 22.48620584 | 10.9400408 | 32.59452672 | 10.93560008 | 2.5526199 | 0 | Gm42545 |
| -1.121775518 | 0.00954224 | 0.02700087 | 43.09856119 | 43.76016321 | 39.95458114 | 30.07290023 | 12.7630995 | 14.99581234 | Slfn10-ps |
| -1.165286793 | 0.00955768 | 0.02703265 | 25.29698157 | 61.08189448 | 43.10889018 | 16.40340013 | 18.7192126 | 23.17534634 | Med9os |
| -1.244209259 | 0.00969321 | 0.0273438 | 48.72011266 | 54.70020401 | 62.03474441 | 23.69380018 | 37.43842519 | 6.816278335 | Angpt4 |
| -1.334091759 | 0.00989472 | 0.02781458 | 29.98160779 | 31.90845234 | 19.97729057 | 15.49210012 | 8.508732999 | 8.179534002 | Gm8369 |
| -1.625715086 | 0.01005229 | 0.02817534 | 20.61235535 | 13.675051 | 17.87441788 | 4.556500035 | 6.806986399 | 5.453022668 | Gm20684 |
| -1.010016461 | 0.01018359 | 0.02848539 | 117.1156554 | 158.6305916 | 152.4582702 | 81.10570063 | 102.9556693 | 25.90185767 | Tinagl1 |
| -1.039372729 | 0.01041057 | 0.02902766 | 44.03548644 | 74.75694548 | 52.57181729 | 26.4277002 | 39.14017179 | 16.359068 | Cfap221 |
| -1.07977404 | 0.01074775 | 0.02985126 | 41.22471071 | 77.49195568 | 33.64596307 | 25.5164002 | 24.6753257 | 21.81209067 | Arl11 |
| -1.445114148 | 0.01096601 | 0.03034816 | 28.1077573 | 67.46358495 | 21.02872692 | 8.201700063 | 16.1665927 | 19.08557934 | Adgrg2 |
| -1.042446163 | 0.01098197 | 0.03037924 | 117.1156554 | 121.2521189 | 177.6927425 | 119.3803009 | 41.69279169 | 39.53441434 | Fxyd2 |
| -1.150044144 | 0.011277 | 0.0310218 | 45.90933693 | 60.17022441 | 36.80027211 | 21.87120017 | 31.4823121 | 9.542789669 | Adrb3 |
| -1.330629954 | 0.01156085 | 0.03168512 | 36.54008449 | 31.90845234 | 25.2344723 | 20.95990016 | 7.657859699 | 8.179534002 | Fap |
| -1.209674273 | 0.01179791 | 0.03223403 | 28.1077573 | 34.64346254 | 27.33734499 | 10.02430008 | 18.7192126 | 9.542789669 | Ccdc183 |
| -1.10618772 | 0.01182179 | 0.03229012 | 40.28778546 | 33.73179247 | 25.2344723 | 17.31470013 | 12.7630995 | 16.359068 | Gm26674 |
| -1.272297186 | 0.01203193 | 0.03277125 | 25.29698157 | 22.79175167 | 28.38878134 | 14.58080011 | 8.508732999 | 8.179534002 | 2210011C24Rik |
| -1.624836259 | 0.01219035 | 0.03313253 | 22.48620584 | 18.23340134 | 13.6686725 | 5.467800042 | 3.4034932 | 9.542789669 | Olfr111 |
| -1.343013788 | 0.01225747 | 0.03327742 | 25.29698157 | 28.26177207 | 34.69739941 | 4.556500035 | 14.4648461 | 16.359068 | Opn1sw |
| -1.034066329 | 0.01226333 | 0.03328864 | 34.666234 | 43.76016321 | 32.59452672 | 21.87120017 | 13.6139728 | 19.08557934 | Gm27419 |
| -1.294983162 | 0.01234967 | 0.03348639 | 20.61235535 | 26.43843194 | 25.2344723 | 12.7582001 | 9.359606299 | 6.816278335 | Ecscr |
| -1.29265361 | 0.01238281 | 0.03356101 | 33.72930876 | 24.61509181 | 33.64596307 | 5.467800042 | 16.1665927 | 16.359068 | Ripk4 |
| -1.11343883 | 0.01275732 | 0.03436351 | 43.09856119 | 42.84849314 | 44.16032653 | 9.11300007 | 28.0788189 | 23.17534634 | 4930556M19Rik |
| -1.043731837 | 0.01295807 | 0.03482639 | 31.85545827 | 44.67183328 | 39.95458114 | 16.40340013 | 25.526199 | 13.63255667 | Nyx |
| -1.524707332 | 0.01299123 | 0.03488633 | 20.61235535 | 41.93682308 | 13.6686725 | 11.84690009 | 7.657859699 | 6.816278335 | Gm27366 |
| -1.075900464 | 0.01300313 | 0.03490857 | 39.35086022 | 26.43843194 | 31.54309038 | 16.40340013 | 13.6139728 | 16.359068 | Slc26a9 |
| -1.42737844 | 0.01322614 | 0.03541359 | 15.92772914 | 51.05352374 | 23.13159961 | 12.7582001 | 11.0613529 | 9.542789669 | Hmgcs2 |
| -1.236861157 | 0.01410854 | 0.0373663 | 35.60315925 | 21.8800816 | 23.13159961 | 11.84690009 | 13.6139728 | 8.179534002 | Ip6k3 |
| -1.039276249 | 0.01464134 | 0.03852342 | 40.28778546 | 31.90845234 | 53.62325364 | 25.5164002 | 22.1227058 | 12.269301 | Nkx1-1 |
| -1.442814117 | 0.01465459 | 0.03854777 | 22.48620584 | 20.96841154 | 18.92585423 | 11.84690009 | 4.254366499 | 6.816278335 | Adh6b |
| -1.424715767 | 0.01472095 | 0.03870121 | 15.92772914 | 28.26177207 | 16.82298153 | 7.290400056 | 8.508732999 | 6.816278335 | Susd3 |
| -1.057422542 | 0.01489086 | 0.0390946 | 44.97241168 | 42.84849314 | 36.80027211 | 14.58080011 | 30.6314388 | 13.63255667 | Erich2os |
| -1.507863948 | 0.01509224 | 0.03953715 | 30.91853303 | 27.35010201 | 14.72010884 | 6.379100049 | 14.4648461 | 4.089767001 | Slc6a19 |
| -1.392999138 | 0.01519361 | 0.03976489 | 18.73850487 | 38.29014281 | 18.92585423 | 10.93560008 | 11.9122262 | 5.453022668 | Eldr |
| -1.434595226 | 0.01525873 | 0.03991535 | 21.5492806 | 26.43843194 | 13.6686725 | 10.02430008 | 5.956113099 | 6.816278335 | Gm12107 |
| -1.597401114 | 0.01537435 | 0.04016162 | 20.61235535 | 19.1450714 | 36.80027211 | 2.733900021 | 15.3157194 | 6.816278335 | 9330121J05Rik |
| -1.015563966 | 0.01580948 | 0.0411109 | 64.64784179 | 48.31851354 | 34.69739941 | 23.69380018 | 33.1840587 | 14.99581234 | Gm26676 |
| -1.295235473 | 0.01585211 | 0.04117821 | 30.91853303 | 18.23340134 | 31.54309038 | 14.58080011 | 11.9122262 | 5.453022668 | Cyp11a1 |
| -1.103282106 | 0.01589545 | 0.04127123 | 40.28778546 | 24.61509181 | 30.49165403 | 10.93560008 | 16.1665927 | 17.72232367 | Gm10710 |
| -1.291335098 | 0.01641801 | 0.04239396 | 23.42313108 | 16.4100612 | 28.38878134 | 10.93560008 | 8.508732999 | 8.179534002 | Atp12a |
| -1.418721833 | 0.01699329 | 0.0436398 | 13.11695341 | 21.8800816 | 21.02872692 | 7.290400056 | 6.806986399 | 6.816278335 | 1700041G16Rik |
| -1.478094243 | 0.01868172 | 0.04720315 | 23.42313108 | 17.32173127 | 18.92585423 | 3.645200028 | 11.9122262 | 5.453022668 | Gm17110 |
| -1.162622826 | 0.01931116 | 0.04845004 | 30.91853303 | 20.05674147 | 24.18303596 | 10.93560008 | 12.7630995 | 9.542789669 | Kcnj5 |
| -1.581036653 | 0.01947302 | 0.04879897 | 17.80157962 | 11.85171087 | 16.82298153 | 5.467800042 | 6.806986399 | 2.726511334 | 8430408G22Rik |
| -1.160802362 | 0.01961314 | 0.04909906 | 31.85545827 | 29.17344214 | 18.92585423 | 9.11300007 | 14.4648461 | 12.269301 | Robo3 |
| -1.018142998 | 0.01987014 | 0.04963286 | 69.33246801 | 64.72857475 | 69.39479883 | 58.32320045 | 17.017466 | 24.53860201 | Oas3 |
| -1.031700186 | 0.02006495 | 0.0499835 | 40.28778546 | 49.23018361 | 27.33734499 | 16.40340013 | 26.3770723 | 13.63255667 | Rasal3 |
